# Integrative Transcriptomic Analyses of Hippocampal–Entorhinal System Subfields Identify Key Regulators in Alzheimer's Disease

**DOI:** 10.1002/advs.202300876

**Published:** 2023-05-26

**Authors:** Dan Luo, Jingying Li, Hanyou Liu, Jiayu Wang, Yu Xia, Wenying Qiu, Naili Wang, Xue Wang, Xia Wang, Chao Ma, Wei Ge

**Affiliations:** ^1^ Department of Immunology State Key Laboratory of Complex Severe and Rare Diseases Institute of Basic Medical Sciences Chinese Academy of Medical Sciences School of Basic Medicine Peking Union Medical College Beijing 100005 China; ^2^ Department of Human Anatomy Histology and Embryology Neuroscience Center National Human Brain Bank for Development and Function Institute of Basic Medical Sciences Chinese Academy of Medical Sciences School of Basic Medicine Peking Union Medical College Beijing 100005 China

**Keywords:** A1 reactive astrocyte, Alzheimer's disease, hippocampal–entorhinal system, PSAP, subfields

## Abstract

The hippocampal–entorhinal system supports cognitive function and is selectively vulnerable to Alzheimer's disease (AD). Little is known about global transcriptomic changes in the hippocampal–entorhinal subfields during AD. Herein, large‐scale transcriptomic analysis is performed in five hippocampal–entorhinal subfields of postmortem brain tissues (262 unique samples). Differentially expressed genes are assessed across subfields and disease states, and integrated genotype data from an AD genome‐wide association study. An integrative gene network analysis of bulk and single‐nucleus RNA sequencing (snRNA‐Seq) data identifies genes with causative roles in AD progression. Using a system‐biology approach, pathology‐specific expression patterns for cell types are demonstrated, notably upregulation of the A1‐reactive astrocyte signature in the entorhinal cortex (EC) during AD. SnRNA‐Seq data show that PSAP signaling is involved in alterations of cell– communications in the EC during AD. Further experiments validate the key role of PSAP in inducing astrogliosis and an A1‐like reactive astrocyte phenotype. In summary, this study reveals subfield‐, cell type‐, and AD pathology‐specific changes and demonstrates PSAP as a potential therapeutic target in AD.

## Graphical abstract

The food pyramid is used to illustrate the overall research strategy. The large‐scale frozen tissues of hippocampal–entorhinal subfields from China Human Brain Bank, are used for deep whole transcriptome sequencing, combined analyses with single‐cell data and transcription‐wide association studies (TWAS). This research discovers the functions of the PSAP protein and three risk genes in Alzheimer's disease.

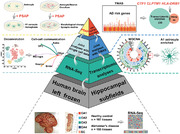

## 1 Introduction

Alzheimer's disease (AD) is the most common form of dementia and is becoming one of the world's most costly, deadly, and debilitating illnesses.^[^ ^1^ ^]^ Unfortunately, existing drugs only address the symptoms of dementia and are not curative, and the mechanisms underlying AD development remain poorly understood. Clinical trials of AD therapies have consistently failed.^[^ ^2^ ^]^ The possible reason is that animal models exhibit different cognitive deficits and pathological progression from human AD, and extrapolating conclusions from one to the other may be misleading.^[^ ^3^ ^]^ Therefore, elucidating molecular changes in the human brain that contribute to AD progression is important for the development of effective therapies.

AD exhibits a well‐characterized progression pattern, with the pathology beginning in the areas of the brain involved in learning, memory, and cognition, such as the entorhinal cortex (EC) and hippocampus, and then spreading throughout the cortex.^[^ ^4^ ^]^ The hippocampal–entorhinal system is composed of distinctive subfields, including the cornu ammonis (CA) subfields 1–4, subiculum, dentate gyrus, and fimbria, which are interconnected, histologically heterogeneous, and functionally specialized.^[^ ^5^ ^]^ Several studies have indicated that the hippocampal subfields play distinct roles in AD pathology.^[^ ^6^, ^7^ ^]^ Several cell types and circuits in the human hippocampal–entorhinal system are selectively involved in AD pathology.^[^ ^8^ ^]^ Therefore, a more detailed molecular and cell profiling of this system will help understand the human brain and AD.

Here, to explore key cell type‐ and subfield‐specific differences in gene expression and AD susceptibility, we performed next‐generation sequencing of five anatomically defined subfields (CA1‐CA4 and the EC) of the hippocampal–entorhinal system of postmortem brains from a large cohort of individuals with AD. Our results revealed extensive transcriptional changes across all five subfields of the brains with AD compared to those in healthy controls. These findings are useful for elucidating the mechanisms underlying AD pathology across the hippocampal–entorhinal system.

Transcription‐wide association studies (TWAS) help identify associations among genotypes, transcripts, and disease states.^[^ ^9^ ^]^ In this study, we conducted TWAS to identify pathogenic genes associated with AD risk using the Genotype‐Tissue Expression (GTEx) database. Because TWAS results may be perturbed by the cellular composition of samples, for each subfield, we deconvoluted bulk tissue transcriptomic data using CIBERSORTx to identify the effects of neuropathology on cellular composition. To determine whether each hippocampal–entorhinal subfield has different effects in AD pathology, and whether such variability preferentially involves specific cell types, we used weighted gene co‐expression network analysis (WGCNA) to identify modules of highly co‐expressed genes enriched with markers for major cell types.

Astrocytes play a critical role in the brain by regulating neuronal activity and providing metabolic support to neurons.^[^ ^10^ ^]^ In response to diseases, astrocytes can be activated into two polarization states: the neurotoxic or pro‐inflammatory phenotype (A1) and the neuroprotective or anti‐inflammatory phenotype (A2).^[^ ^11^ ^]^ Notably, we observed a glia‐associated module which showed that A1‐reactive astrocytes transcriptomic signatures were significantly enriched in the EC. Our data revealed a significant correlation between neurons and astrocytes in the EC. We also used phenotype information from our bulk data to identify neuron and astrocyte subpopulations that drive AD phenotypes in the EC.^[^ ^12^ ^]^ Our data demonstrated that astrocyte–neuron interplay in the EC is critical for AD pathogenesis.

Accumulating evidence has been reviewed that reactive astrocytes are triggered in response to injury or neurodegeneration, such as A1‐reactive astrocytes in the progression of many neurological diseases.^[^ ^13^ ^]^ A1‐reactive astrocytes can amplify inflammatory response and exert neurotoxic effects, which are involved in AD.^[^ ^14^ ^]^ To identify the key signals between neurons and astrocytes, we analyzed cell–cell communication. Our data showed widespread alteration in the cell–cell communication in the EC of patients with AD. Moreover, we found a key mediator, PSAP, that can promote astrogliosis and induce the generation of A1‐reactive astrocytes.

Overall, in this study, we report an extensive transcriptomic analysis of gene expression changes in 262 unique samples of postmortem brains. Our study uncovers novel mechanisms that can be targeted to treat neurodegenerative diseases and offers new insights into AD pathology.

## 2 Results

### 2.1 Transcriptomic Profiling of Hippocampal–Entorhinal System Subfields in AD

**Figure 1:**
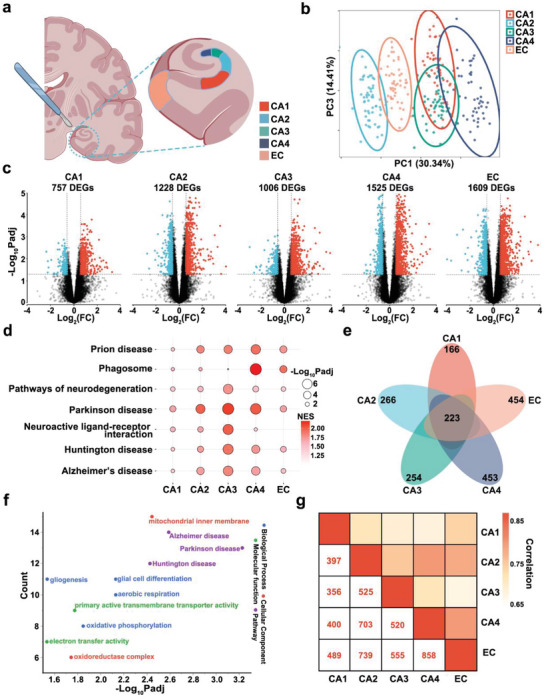
Transcriptomic profiling of hippocampal–entorhinal system subfields from individuals with AD pathology compared with healthy controls (no‐pathology). a) Sketch of the hippocampal–entorhinal system anatomy with manually segmented subfields. b) Principal component analysis plot of hippocampal–entorhinal system subfield RNA‐Seq data; CA1 (*n* = 50), CA2 (*n* = 53), CA3 (*n* = 53), CA4 (*n* = 53), and EC (*n* = 53). Each dot represents a sample, and each color represents a subfield. c) Volcano plots of DEGs for each subfield; blue dots indicate downregulated genes and red dots indicate upregulated genes. d) Gene Set Enrichment Analysis of each subfield. e) Venn diagram for identifying overlapping DEGs. f) GO and KEGG enrichment analyses of overlapping DEGs in the five subfields. g) Pairwise correlation of DEGs from the five subfields. The numbers represent the number of overlapping DEGs. EC, entorhinal cortex; CA1–4, cornu ammonis (CA) subfields 1–4.

We used RNA sequencing (RNA‐Seq) to examine five hippocampal–entorhinal system subfields (CA1–CA4 and the EC) of frozen postmortem brains from individuals with pathological hallmarks of AD and healthy controls (Table S1, Figure S1a, Supporting Information). Initially, 14 µm sections from tissues adjacent to the subfields were obtained and subjected to hematoxylin–eosin to determine the orientation, and then a series of cuts was made to isolate the CA1–CA4 and the EC (**Figure** **1**a). We performed principal component analysis (PCA) (Figure S1b, Supporting Information) for the effects of clinical characteristics including AD pathology score, gender, age, and postmortem interval (PMI), on total variance across all subfields (Figure S1c, Supporting Information). We observed that the AD pathology score had the most robust effect on the variance of the transcriptome (Figure S1d, Supporting Information), indicating that there are significant pathology‐specific differences in the AD transcriptome compared with the healthy control. These clinical characteristics as biological covariates, and RNA integrity number (RIN) and batch as technical covariates were included in our model to normalize the data. We observed marked separation of the CA2, EC, and CA4 based on gene expression variance trends (Figure 1b), suggesting the importance of conducting subfield‐specific analyses. For each subfield, we examined 31 individuals with AD pathology and 22 individuals with no pathology (healthy controls). We identified 757 differentially expressed genes (DEGs) in the CA1, 1228 in CA2, 1006 in CA3, 1525 in CA4, and 1609 in EC in the AD transcriptome compared with healthy control (Figure 1c, Table S2, Supporting Information). Comparisons of the total number of DEGs in each subfield indicated that the EC may be more severely affected in AD.

To elucidate AD‐related pathway changes in each subfield, we performed Gene Set Enrichment Analysis. The AD and neurodegeneration pathway signatures were significantly and positively enriched (Figure 1d), suggesting that neurodegeneration‐related molecular changes occurred in each subfield. In addition to separately identifying DEGs in the subfields, we also identified overlapping DEGs between subfields to assess consistent disease‐relevant expression patterns. Only 223 DEGs were observed in all five regions, hinting the existence of different DEGs in different brain regions (Figure 1e). Gene Ontology (GO) analysis of the 223 overlapping DEGs revealed multiple enriched terms, including gliogenesis and glial cell differentiation, suggesting a role for glial cells in AD pathophysiology. Kyoto Encyclopedia of Genes and Genomes (KEGG) pathway analysis showed that overlapping DEGs were assigned to the term of AD pathway, further validating that AD‐related transcriptional changes occurred in each subfield, despite the differences in vulnerability to AD pathology (Figure 1f). To assess the functional coherence of the subfields, we performed pairwise comparisons of DEGs. The EC and CA4 shared the highest number of DEGs (858 genes), while CA1 to CA3 shared the lowest (356 genes). We also analyzed the correlations of expression fold‐changes across the subfields, finding a high correlation between EC and CA4 (Figure 1g). These findings suggest that AD pathological processes extensively impact the functional interconnectivity among the subfields, particularly EC with CA4.

### 2.2 TWAS Identify AD‐Associated Genes

**Figure 2:**
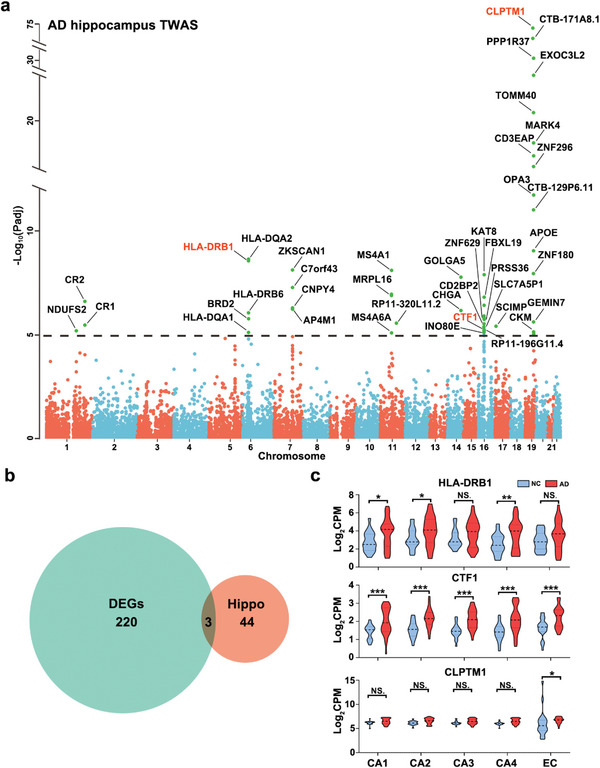
TWAS for identifying AD‐associated genes. a) Manhattan plot of TWAS by FUSION using hippocampal tissue data. Points above the dotted line indicate genes significant for AD (Bonferroni corrected *p‐*values). b) Venn diagram of DEGs shared by the five subfields and TWAS comparisons. c) RNA‐Seq results for *HLA‐DRB1*, *CTF1*, and CLPTM1 in the five subfields. Unpaired two‐tailed Student's *t*‐test, *p < 0.05, **p < 0.01, ***p < 0.001.

We observed vast transcript expression changes across the five subfields. However, it was unclear which changes were driving AD. TWAS can identify the associations among genotypes, transcripts, and disease states.^[^ ^9^ ^]^ Thus, we performed TWAS to identify genes whose mRNA expression was significantly associated with AD. We performed FUSION‐TWAS using the GTEx database of tissue‐specific expression quantitative trait loci (eQTLs) data as the reference panel to reanalyze summary‐level data from recently reported genome‐wide meta‐analyses of AD.^[^ ^15^, ^16^ ^]^ We conducted FUSION‐TWAS on the GTEx database of hippocampal tissue. Forty‐seven genes were identified by FUSION using the hippocampal tissue eQTLs data (**Figure** **2**a, Table S3, Supporting Information). Among the 223 overlapping DEGs obtained from our transcriptomic analysis of subfields CA1–4 and the EC, we found that three, *HLA‐DRB1*, *CTF1*, and *CLPTM1*, were hippocampal TWAS hits (Figure 2b). *CLPTM1* is a known AD locus.^[^ ^17^ ^]^ Notably, among the three genes, *CTF1* showed the most significantly increased expression in each subfield (Figure 2c). To the best of our knowledge, the functional consequences of the risk allele *CTF1* have not yet been reported in AD. In addition, we ran FUSION‐TWAS using GTEx database eQTLs data of the human brain cortex. FUSION identified 32 genes (Figure S2, Table S4, Supporting Information). Among them, *HLA‐DRB1* was also a significant cortical TWAS hit and has been reported to be associated with cognitive ability in individuals with and without dementia.^[^ ^18^ ^]^

### 2.3 Consensus Gene Co‐Expression Network Analysis Across Subfields

To extract additional biological information from the transcriptome dataset, we performed WGCNA. We processed the RNA‐Seq dataset of the 262 samples from CA1‐CA4 and EC regions and identified 49 gene modules (Figure S3a, Supporting Information). Because different network clustering algorithms can produce disparate networks, we tested the robustness of the network generated by the WGCNA algorithm by calculating module preservation statistics. We found that 47 of the 49 WGCNA‐generated modules were preserved (Zsummary > 2) (Figure S3b, Supporting Information). We next performed GO analysis on each module and found that module M07 showed the highest number of enriched categories that were associated with the nervous system (Figure S3c, Supporting Information).

**Figure 3:**
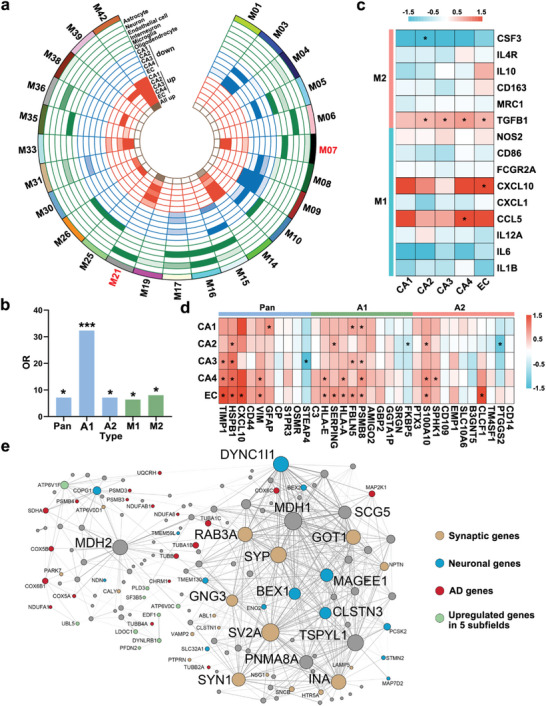
Consensus gene co‐expression network analysis across subfields. a) Degree of enrichment of DEGs and cell‐type‐specific markers in each module. Colors represent corrected Fisher's exact test *p*‐values. b) Module M07 was enriched in transcriptomic signatures associated with pan‐reactive, A1 and A2 astrocytes, and M1 and M2 microglia. Fisher's exact test, *p < 0.05, ***p < 0.001. c,d) Regional expression of selected transcripts associated with microglial polarization (microglial phenotypes M1, M2) and astrocyte activation (pan‐reactive, A1, and A2 phenotypes) in module M07, with upregulation indicated by red and downregulation indicated by blue. Fold change values of genes and adjusted *p‐*values were calculated by using the DEseq2 R package. *Adjusted *p‐*values < 0.05. e) Top WGCNA connections of AD‐associated module M21. Node size represents the number of gene coexpression connections. Tan: synapse‐related genes; blue: neuron‐related genes; red: AD signaling pathway‐related genes; green: genes upregulated in all five subfields.

To determine how the composition of cell types in CA1–CA4 and the EC changes with the progression of AD and quantify the relationships between subfields and disease using DEGs, we plotted the modules with enrichment for cell‐type‐specific markers and had significant numbers of DEGs across each subfield in **Figure** **3**a. Module M07 was significantly enriched for markers of microglia and astrocytes. To further elucidate glial response changes in module M07, we conducted enrichment analyses of known transcriptomic signatures of reactive microglia and astrocytes. The A1‐specific reactive astrocyte signature was the most significantly and positively enriched (Figure 3b). In contrast to observations in the no‐pathology group, in each subfield of the AD‐pathology group, we found increased expression of the A1‐reactive marker, whereas the microglia‐reactive marker did not exhibit consistently elevated expression changes (Figure 3c,d). Notably, the EC was the region with the most noticeable elevation of the A1‐reactive marker. Collectively, these results suggest that an A1‐like reactive astrocyte phenotype may be triggered in each subfield with AD‐pathology, especially in the EC.

KEGG pathway enrichment analysis revealed that module M21 was strongly correlated with multiple neurodegenerative diseases, including AD signaling (Figure S3d, Supporting Information). Module M21 was significantly enriched in markers of neuronal cells and contained DEGs upregulated in all five subfields, revealing a signature of neurons being affected in CA1–CA4 and the EC in AD. Genes with a high degree of connectivity within a module are termed “hub genes” and are expected to be functionally important within the module. Module M21 mostly contained synaptic and neuronal hub genes (Figure 3e). The hub gene, *MDH2*, encodes malate dehydrogenase (MDH). It has been reported that MDH activity was elevated in the brains of AD patients,^[^ ^19^ ^]^ and higher levels of MDH2 protein were observed in AD tissue than in control hippocampal tissue.^[^ ^20^ ^]^ We validated the elevated expression of *MDH2* at the mRNA level in all five subfields (Figure S3e, Supporting Information), suggesting a key functional role of MDH2 in AD.

### 2.4 Region‐Specific Transcriptomic Changes During AD Pathological Progression

In the PCA analysis for clinical characteristics, the AD pathology score had the greatest effect on the variance of the transcriptome (Figure S1b–d, Supporting Information). This indicated highly significant pathological progression‐specific differences in the AD transcriptome compared with healthy controls. According to the “ABC” pathology score, individuals with AD‐pathology were segregated into two subgroups: an “intermediate (I)” score, indicating early pathology, and a “high (H)” score, indicating late pathology. We then respectively quantified gene expression changes in these two subgroups compared with the no‐pathology groups. Significant transcriptomic differences between the two AD pathology subgroups and the no‐pathology group were found in all five subfields (Figure S4a,b, Table S5, Supporting Information). More genes were differentially expressed between the late and no‐pathology groups: 2376 DEGs in CA1, 2310 in CA2, 2378 in CA3, 2616 in CA4, and 3314 in the EC. In contrast, very few DEGs were observed when comparing the early‐ and no‐pathology groups. A few DEGs overlapped between late‐ and early‐pathology groups in each subfield (Figure S4c, Supporting Information). These data indicate that the early‐ and late ‐pathologies of AD have unique, regional transcriptomic profiles in terms of transcript changes and the total number of DEGs.

**Figure 4:**
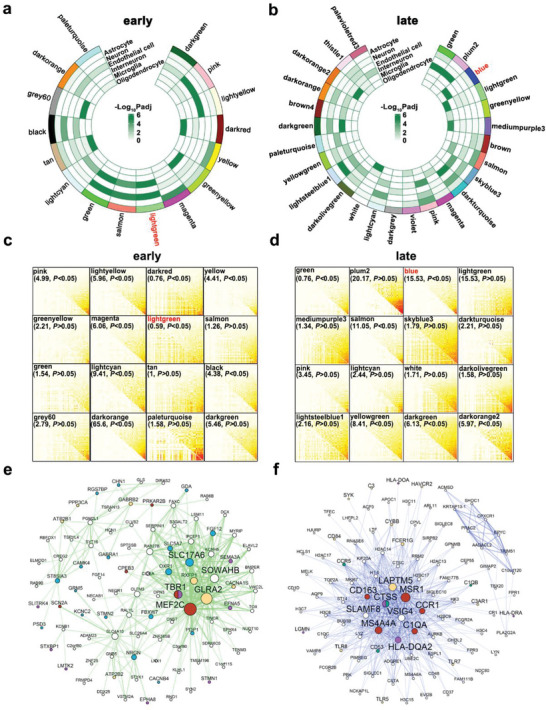
Region‐specific transcriptomic changes during the pathological progression of AD. a,b) Circos plots showing cell‐type‐specific markers for each module in a) early‐pathology and b) late‐pathology. c,d) Differential connection analysis for modules in AD‐pathology versus no‐pathology. c) Early‐pathology versus no‐pathology; d) late‐pathology versus no‐pathology. e) Top WGCNA connections of early‐pathology associated light‐green module (blue, ‐neuronal genes; yellow, ‐genes from neuroactive ligand–receptor interaction GO category; purple, ‐genes from axonogenesis category; red, ‐genes from learning or memory category). f) Top WGCNA connections of late‐pathology associated blue module. (green, ‐microglial genes; yellow, ‐genes from positive regulation of cytokine production category; purple, ‐genes from antigen processing and presentation category; red, ‐AD‐associated genes).

Because few DEGs were found when comparing the early‐ and no‐pathology groups, we hypothesized that their physiological differences may be explained by co‐regulated gene networks. The early‐pathology co‐expression network analysis combining CA1–CA4 and the EC identified 60 modules. While 78 modules were found in the late‐pathology co‐expression network analysis. Of these, 16 early‐pathology modules (**Figure** **4**a) and 26 late‐pathology modules (Figure 4b) were enriched for cell‐type‐specific markers. To detect and quantify network differences across early‐ or late‐ and no‐pathology states, we performed module differential connectivity (MDC) analysis.^[^ ^21^ ^]^ MDC > 1 indicates gain of connectivity (GOC) or enhanced coregulation between genes, whereas MDC < 1 indicates loss of connectivity (LOC) or reduced coregulation between genes. Based on a 5% false‐discovery rate (FDR), in the early‐pathology network, 28 modules showed GOC and two modules exhibited LOC compared with a healthy control group (Figure S4d, Table S6, Supporting Information). We noted that the light‐green module, which was enriched for neurons, showed the most significant LOC (Figure 4c), implying that the corresponding neuron‐related genes in the light‐green module were disrupted in early‐pathology. In the late‐pathology network, 38 GOC modules and two LOC modules were observed compared with the healthy control group (Figure S4e, Table S7, Supporting Information). Notably, the blue module, which was enriched for microglia and astrocyte, showed the greatest GOC (Figure 4d), suggesting that the glial functions were upregulated. The light‐green module, which showed the most significant LOC in early‐pathology, and the blue module, which showed the greatest GOC in late‐pathology, were further examined for key drivers of high network connection. The light‐green module, which mostly consisted of neuronal genes (Figure 4e), was significantly enriched in functional terms, such as learning or memory. *MEF2C*, a hub gene in the light‐green module, has been identified in multiple studies as an AD risk gene.^[^ ^22^, ^23^ ^]^ The blue module was enriched in glial genes (Figure 4f). The hub genes included *MSR1*, which encodes a scavenger receptor that can facilitate amyloid‐*β* phagocytosis and inflammation.^[^ ^24^, ^25^ ^]^ According to these findings, AD pathology perhaps begins with neuronal dysfunction, followed by glial cell‐mediated neuroinflammation in the late stage.

### 2.5 Quantification of Cell‐Type Differences Occurring with Progression of AD Pathology

**Figure 5:**
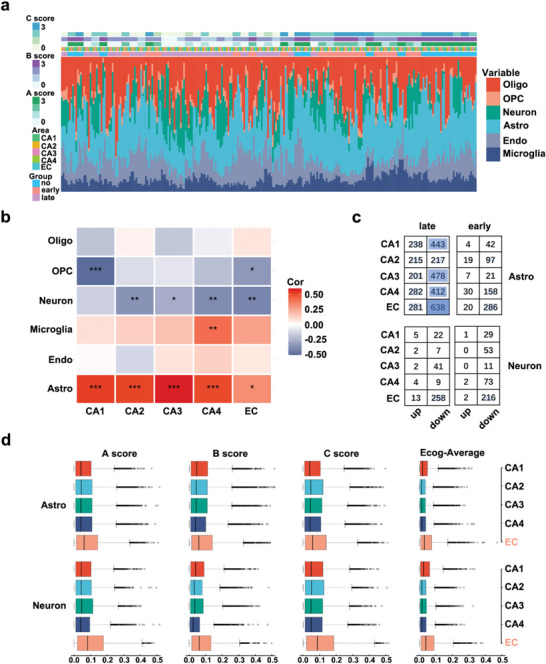
Cell‐type‐specific transcriptomic changes in the five hippocampal–entorhinal system subfields. a) The fractions of six cell types in each sample. b) Correlation heatmap of estimated cell‐type proportions in bulk RNA‐Seq data plotted against AD‐pathology score. The colors represent the coefficient of correlation. The coefficient of correlation and *p*‐values were calculated by using Spearman's correlation. *p < 0.05, **p < 0.01, ***p < 0.001. c) DEG counts in astrocytes and neurons for each subfield. d) Subfield‐level transcriptome‐wide gene–trait correlation analysis in astrocytes and neurons. Boxplots show the distribution of absolute values of the correlation coefficient (Spearman correlation) computed between gene expression profiles for astrocytes and neurons across each subfield and the corresponding pathological traits across individuals.

Because the examination of cell–type marker genes within modules revealed several early‐ or late‐pathology–specific modules that were enriched for cell‐type marker genes, we speculated that the proportions of cell‐types may be altered in hippocampal–entorhinal system subfields of AD patients. Thus, we conducted deconvolution analysis in our RNA‐Seq data to quantify the proportions of cell types using a published human snRNA‐Seq dataset as a reference^[^ ^26^ ^]^ (Figure S5a, Supporting Information). Using the CIBERSORTx method, we first quantified the proportions of central nervous system (CNS) cell–types (**Figure** **5**a). The estimated proportions of astrocytes in CA1–CA4 and the EC were significantly higher in the early‐ and late‐pathology groups than in the no‐pathology group, whereas the proportion of neurons was lower. The estimated proportions of oligodendrocytes showed slight changes in different regions and the proportions of other CNS cell–types were unchanged (Figure S5b–f, Supporting Information). In general terms, the data demonstrated that the changes in the proportions of astrocytes and neurons with AD pathology were mostly consistent across the five subfields. Notably, correlation analysis showed that the relative abundance of astrocytes was significantly positively correlated with AD pathology score, whereas neurons were negatively correlated in most subfields (Figure 5b). Next, we performed further analysis of neurons and astrocytes in all five subfields. We obtained cell‐intrinsic DEGs from the high‐resolution mode of CIBERSORTx for neuronal and astrocytic cell types, respectively (Figure S6, Supporting Information). We observed that neurons and astrocytes in the EC had the most cell‐intrinsic DEGs compared with the no‐pathology group, regardless of the pathology stage examined (early or late) (Figure 5c), indicating that the most significant vulnerability of neurons and astrocytes to AD pathology occurs in the EC. To determine whether astrocytes and neurons exhibit regionally different responses to AD pathology, we computed gene–trait correlations. Consistently, the astrocytes and neurons in the EC showed increased correlations with pathological traits and cognition (Figure 5d). Overall, the above results indicate that neurons and astrocytes in the EC were the most vulnerable to AD pathology.

### 2.6 Alterations of Cell–Cell Communications in the EC of AD Patients

**Figure 6:**
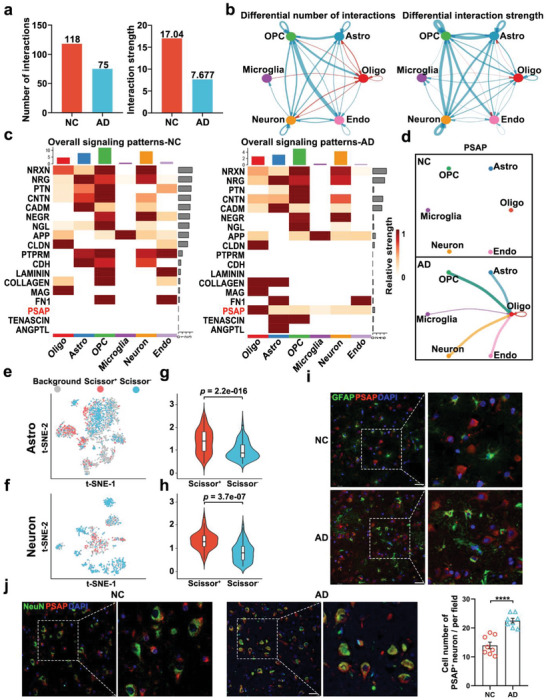
Alterations of cell–cell communications in the EC of patients with AD. a) Decrease in the number of interactions and interaction strength among the cells in the EC of patients with AD. b) Overview of the intercellular communication networks among cells measured by network centrality analysis. Nodes with different colors represent different cell populations, and the red (blue) edge represents an increase (decrease) in the number of interactions or interaction strength in the EC of patients with AD compared with controls. c) Heatmaps of the overall signaling flows for each cell population mediated by individual signaling axes in patients with AD and controls. d) The PSAP signaling pathway network was significantly altered in the EC of patients with AD compared with controls. e,f) t‐distributed stochastic neighbor embedding (t‐SNE) visualizations of Scissor‐selected cells: e) astrocytes and f) neurons. g,h) Violin plots of PSAP expression in Scissor^+^ cells and Scissor^−^ cells; g) astrocytes and h) neurons. i,j) Representative co‐immunofluorescence staining for i) PSAP and GFAP (Glial Fibrillary Acidic Protein), and j) for PSAP and NeuN in the hippocampus. Scale bars, 30 µm. *n* = 8. Data are the mean ± standard error of the mean (S.E.M.). Unpaired two‐tailed Student's *t*‐test. ****p < 0.0001.

As neuronal loss and astrogliosis in the EC significantly correlate with the pathological progression of AD, we investigated whether neuronal–astroglial communication was altered. We conducted a cell–cell communication analysis in the EC. We first compared the global communication atlas between the control and AD samples. We found that the total inferred interactions between cells decreased in the AD samples in terms of both the number and strength of the interactions (**Figure** **6**a), but the number of interactions of astrocytes, neurons, and oligodendrocytes increased (Figure 6b). To dissect this change in communication, we calculated the information flow of each signaling pathway (Figure S7a, Supporting Information). The PTPRM, CDH, and LAMININ signaling communication pathways were switched off, while the ANGPTL, TENASCIN, and PSAP signaling pathways were switched on in the EC of patients with AD. Other pathways such as PTN and CNTN were downregulated. Furthermore, we detected 18 signaling pathways among the six‐cell populations including oligodendrocyte, astrocyte, neuron, microglia, endothelial cell, and oligodendrocyte precursor cell to distinguish the greatly altered signaling pathways in AD (Figure 6c). We observed that the PSAP signaling patterns were obviously altered in oligodendrocytes, astrocytes, and neurons when the EC was compared between AD and healthy control. The hub of PSAP signaling was located in oligodendrocytes, which was generally increased in AD (Figure 6d). To validate the elevation of PSAP signaling in the EC during AD pathology, we performed immunohistochemical staining to evaluate the expression of PSAP. We found that PSAP expression was increased in the hippocampal tissue of patients with AD pathology (Figure S7b, Supporting Information).

To further substantiate the role of PSAP, we applied the Scissor algorithm to astrocytes and neurons from the EC scRNA‐Seq dataset. Scissors can identify biologically and clinically relevant cell subpopulations from single‐cell assays by integrating phenotypic and bulk‐omics datasets.^[^ ^12^ ^]^ We used our EC samples from the no‐pathology and AD‐pathology groups to guide the Scissor analysis. Scissor^+^ cells were associated with AD‐pathology phenotype, while Scissor^−^ cells were associated with the no‐pathology phenotype. In the standard scRNA‐Seq data analysis, we identified 2456 astrocytes and 1022 neurons (Figure S7c, Supporting Information). The astrocytes and neurons were analyzed separately by Scissor. Among astrocytes, 239 Scissor^+^ cells were found to be associated with AD, 470 Scissor^−^ cells were associated with healthy controls (Figure 6e); 162 of the 239 Scissor^+^ cells (67.8%) were from AD samples, and 32.2% of the Scissor^−^ cells were from control samples (Figure S7d, Supporting Information). Among neurons, 89 Scissor^+^ cells and 494 Scissor^−^ cells were identified (Figure 6f), with 98.9% of the Scissor^+^ cells being from AD samples and 66.8% of the Scissor^−^ cells being from control samples (Figure S7d, Supporting Information). These results indicated that Scissor successfully identified AD‐associated cell subpopulations in astrocytes and neurons. Next, we evaluated the expression of PSAP in Scissor^+^ and Scissor^−^ cells. We observed that in both astrocytes and neurons, PSAP expression in Scissor^+^ cells was higher than that in Scissor^−^ cells (Figure 6g,h). We then conducted immunofluorescence staining to investigate the expression of PSAP in astrocytes and neurons. Analysis of the imaging showed that PSAP was highly expressed in neurons and weakly expressed in astrocytes. It is important to note that the expression of PSAP in neurons was significantly higher in samples from the EC of patients with AD pathology than in samples from individuals with no‐pathology (Figure 6i,j). These findings suggest that PSAP could regulate the communication between neurons and astrocytes and their functions in AD.

### 2.7 PSAP Induced the A1‐Like Reactive Astrocyte Phenotype and Astrogliosis

**Figure 7:**
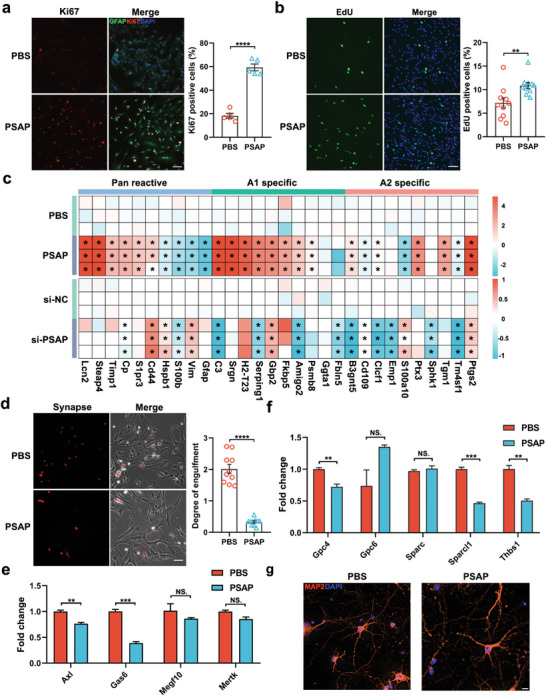
PSAP induced an A1‐like reactive astrocytes phenotype and astrogliosis. a,b) Representative images of astrocyte proliferation measured by a) evaluating Ki67 (*n* = 5 per group) and b) by performing EdU assay (*n* = 10 per group). c) Heat map of pan‐reactive and A1‐ and A2‐specific reactive transcripts in astrocytes 48 h after PSAP treatment or knockdown. d) Phase contrast and fluorescence images of cultured astrocytes engulfing pHrodo‐conjugated synaptosomes (*n* = 10 per group). e) Quantitative PCR analysis of astrocyte‐specific phagocytic receptors. f) Quantitative PCR analysis of astrocyte‐secreted synaptogenic factors (*n* = 3 per group). g) Cropped images of the day in vitro 7 cortical neurons immunostained for 4′,6‐diamidino‐2‐phenylindole (DAPI) and MAP2. Scale bars, 80 µm (a,b), 50 µm (d), and 10 µm (g). Data are the mean ± S.E.M. Unpaired two‐tailed Student's *t*‐test, **p < 0.01, ***p < 0.001, ****p < 0.0001; NS., not significant.

A previous study has defined PSAP as a multifunctional secreted protein.^[^ ^27^ ^]^ To investigate how PSAP signaling impacts the function of neurons and astrocytes, we first generated recombinant rat PSAP protein (Figure S8k, Supporting Information). Because we identified extensive astrogliosis in each subfield (Figure S5, Supporting Information), we first determined whether PSAP had an effect on astrogliosis. We found that treating primary rat astrocytes with PSAP protein led to elevated proliferation, as illustrated by the approximately threefold increase in Ki67^+^ astrocytes that were observed (**Figure** **7**a). Moreover, we detected more proliferating cells (EdU^+^) among PSAP‐treated astrocytes (Figure 7b). Together, these findings suggest that PSAP induces astrogliosis. Having observed that the A1‐specific reactive astrocyte signature was significantly upregulated in each subfield with AD‐pathology (Figure 3d), we next investigated how PSAP influences astrocyte reactivity. We conducted qRT‐PCR assays and assessed which transcripts were upregulated in reactive astrocytes, classified as pan‐reactive, specifically induced by neuroinflammation (A1) or ischemia (A2).^[^ ^14^ ^]^ The results revealed increased expression of most pan‐reactive markers and A1‐reactive markers in PSAP‐treated astrocytes (Figure 7c; Figure S8a, Supporting Information). In contrast, some A1‐reactive markers, such as C3 and Serping1, were substantially decreased in PSAP‐deficient astrocytes (Figure S8b,c, Supporting Information). To further validate the effect of PSAP on astrocyte activation, we treated rat primary astrocytes with different concentrations of PSAP. We rechecked the mRNA expression levels of the most significantly changed Lcn2 (pan reactive marker) and C3 (A1 specific marker). The results showed that PSAP at varied concentrations improved the transcription of Lcn2 and C3 (Figure S8d, Supporting Information). Collectively, these data suggest that PSAP‐treated astrocytes harbor an A1‐like reactive signature. A1‐reactive astrocytes have previously been described to lose phagocytic capacity.^[^ ^14^ ^]^ Therefore, we measured the phagocytic ability of PSAP‐treated astrocytes. We found that PSAP‐treated astrocytes engulfed fewer purified synaptosomes than control astrocytes (Figure 7d). We also performed flow cytometry analysis on primary‐cultured astrocytes to detect the percentage of engulfed synaptosomes. As expected, PSAP treatment significantly decreased synaptosome phagocytosis in astrocytes (Figure S8e, Supporting Information). This phagocytic deficit corresponded with the decrease in mRNA levels of bridging molecules (*Gas6* and *Axl*)^[^ ^28^ ^]^ and phagocytic receptors (*Megf10* and *Mertk*)^[^ ^29^ ^]^ (Figure 7e). Recent studies have shown that A1‐reactive astrocytes are unable to support neuronal function and synapse formation.^[^ ^14^ ^]^ Thus, we also detected the expression levels of synaptogenic factors in PSAP‐treated astrocytes. The qRT‐PCR assays showed decreases in synaptogenic factors including *Gpc4*,^[^ ^30^ ^]^ *Sparcl1*,^[^ ^31^ ^]^ and *Thbs1* ^[^ ^32^ ^]^ (Figure 7f), suggesting that PSAP‐treated astrocytes are unable to support synaptogenesis. We next investigated the effect of PSAP on neuronal morphogenesis in vitro. The morphology of the neurons, including dendrite branch and length did not change significantly (Figure 7g; Figure S8f,g, Supporting Information). The intersection number in the Sholl analysis showed no significant change (Figure S8h, Supporting Information). In terms of synaptogenesis, PSAP had no effect on neurons (Figure S8i,j, Supporting Information). These results suggest that PSAP induced an A1‐like reactive astrocyte phenotype and astrogliosis in vitro, but had no obvious effect on neurons.

### 2.8 PSAP‐Treated Astrocytes are Neurotoxic

**Figure 8:**
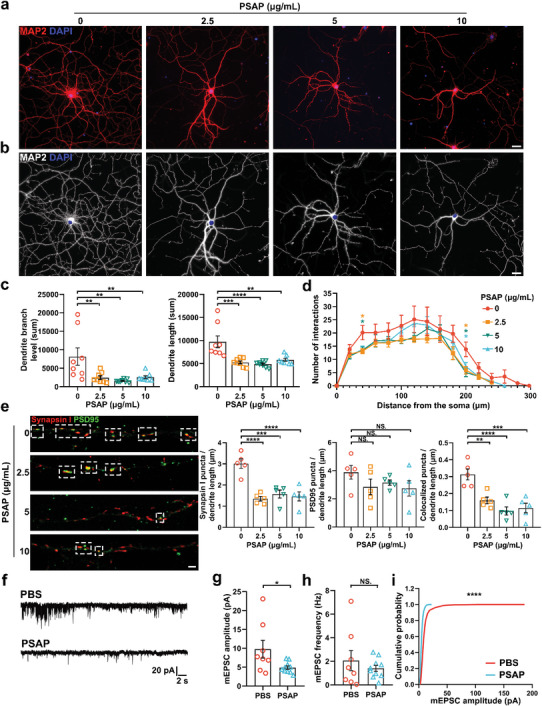
PSAP‐treated astrocytes are neurotoxic. a) Confocal images of immunostaining of MAP2 in co‐cultured neurons following stimulation with different concentrations (0, 2.5, 5, 10 µg mL^−1^) of PSAP in the astrocyte‐neuron co‐culture system. b) Imaris‐rendered neuron morphology immunostained with MAP2 (white) in co‐cultured neurons. c) the Total number of dendrite branch levels and total length of dendrites in co‐cultured neurons following stimulation with different concentrations (0, 2.5, 5, 10 µg mL^−1^) of PSAP in the astrocyte‐neuron co‐culture system (*n* = 8 per group). d) Sholl analysis for quantification of a total of eight co‐cultured neurons in each treatment group. e) Representative images of co‐cultured neurons following stimulation with different concentrations (0, 2.5, 5, 10 µg mL^−1^) of PSAP in the astrocyte‐neuron co‐culture system immunostained with pre‐ and post‐synaptic markers synapsin I (red) and PSD95 (green). Co‐localization (yellow puncta) indicated a structural synapse (*n* = 5 per group). f) Representative recordings of whole‐cell patch clamp mEPSCs. Bar graphs showing g) the amplitude and h) frequency of the mEPSCs, (*n* = 8 in PBS groups and *n* = 10 in PSAP groups). i) Histogram showing the cumulative probability of mEPSC amplitude. Scale bars, 20 µm (a,b), and 2 µm (e). Data are the mean ± S.E.M. *p < 0.05, **p < 0.01, ***p < 0.001, ****p < 0.0001; NS., not significant, as determined by unpaired two‐tailed Student's *t*‐test (for two group comparison), one‐way ANOVA (for multiple group comparison), or multiple *t*‐test (for Sholl analysis).

To ascertain the effects of PSAP‐treated astrocytes on neurons, we co‐cultured astrocytes with primary rat neurons and used a series of different concentrations of purified PSAP protein to treat astrocytes. Altered neuronal morphology accompanies functional changes during aging and disease.^[^ ^33^ ^]^ Thus, we first detected morphological changes in rat cortical neurons co‐cultured with PSAP‐treated astrocytes; MAP2 signals were used to outline neuronal morphology (**Figure** **8**a,b). We found that the dendrite branch number and length were reduced after PSAP protein treatment (Figure 8c). In addition, PSAP treatment reduced the intersection number in the Sholl analysis (Figure 8d). Because we detected decreased expression of synaptogenic factors, such as *Gpc4*, *Sparcl1*, and *Thbs1* (Figure 7f), we assessed whether PSAP‐treated astrocytes had an effect on functional synapses in vitro. Neurons co‐cultured with PSAP‐treated astrocytes had fewer synapses than those cultured with phosphate‐buffered saline (PBS)‐treated astrocytes (Figure 8e). We also performed whole‐cell patch clamp recording on cortical neurons co‐cultured with astrocytes. We observed inhibition of the amplitude of miniature excitatory postsynaptic currents (mEPSCs) (Figure 8f,g) when neurons were co‐cultured with PSAP‐treated astrocytes, but the difference in frequency was not significant (Figure 8h). The neurons co‐cultured with PSAP‐treated astrocytes had significantly more small amplitude mEPSCs than those co‐cultured with PBS‐treated astrocytes in cumulative probability histograms (Figure 8i). These data revealed that PSAP‐treated astrocytes were unable to support neuronal function and synapse formation. This is consistent with observations for A1 astrocytes in rodents.^[^ ^14^ ^]^ Taken together, these results show that PSAP treatment induced A1‐like astrocytes, which were neurotoxic.

## 3 Discussion

In this study, we examined the transcriptomes of the hippocampal–entorhinal system subfields of the postmortem brain samples from individuals with AD and matched healthy controls. This is the first study to comprehensively assess the characteristics of the hippocampal–entorhinal system subfields in Chinese individuals and their involvement in AD pathology.

We found that all five studied subfields were significantly positively enriched in AD signaling pathways, suggesting an AD‐like pathology in each subfield. Our results also clearly showed that the AD‐like pathological vulnerability was different in each subfield; for example, the highest number of DEGs was observed in the EC, followed by CA4. Notably, in terms of expression fold‐changes of genes, the EC and CA4 showed a high correlation even though the anatomical location of CA4 is far from that of the EC, implying that there may be neuronal connectivity between the EC and CA4. Inter‐brain region neuronal connectivity is a scaffolding for neural computation and is organized into topographic maps for relevant stimuli, such as olfactory stimuli.^[^ ^34^ ^]^ There is growing evidence that olfactory dysfunction often precedes cognitive impairment in patients with early‐stage AD.^[^ ^35^ ^]^ Recent evidence showed that the EC is a central region that processes olfactory signaling in the mouse brain.^[^ ^36^ ^]^ However, it is unclear whether CA4 is involved in this processing. In summary, these observations support the notion that the EC is the most vulnerable region to AD pathology, and is closely related to olfactory dysfunction in AD patients.

Given the increasing number of genetic and functional molecules associated with AD, TWAS has become essential for obtaining a better understanding of the mechanisms underlying AD development in the entire brain. The FUSION‐TWAS of hippocampal tissue identified 47 genes with genome‐wide significance using the AD data of Jansen et al.,^[^ ^16^ ^]^ three of which (*HLA‐DRB1*, *CTF1*, and *CLPTM1*) were also differentially expressed when comparing the AD‐pathology group with the healthy control group. HLA‐DRB1 is a human leukocyte antigen class II molecule, which is normally expressed only on the surface of antigen‐presenting cells such as macrophages.^[^ ^37^ ^]^ The expression of *HLA‐DRB1* is low in the homeostatic brain, but it can be rapidly upregulated in activated microglia.^[^ ^38^ ^]^ In AD, microglia can be activated by the accumulation of A*β* plaques.^[^ ^39^ ^]^ The trend of upregulation of *HLA‐DRB1* in the five subfields may imply widespread activation of microglia in the hippocampal–entorhinal system of patients with AD. Although CLPTM1 was previously reported to be associated with AD,^[^ ^40^, ^41^ ^]^ the causal relationship remains unclear. Limited literature indicates that CLPTM1 regulates gamma‐aminobutyric acid (GABA) receptor trafficking from the endoplasmic reticulum to the plasma membrane, suggesting that CLPTM1 may regulate inhibitory neurotransmission.^[^ ^42^, ^43^ ^]^ According to a recent meta‐analysis, patients with AD have decreased levels of GABA, supporting the potential dysregulation of GABAergic signaling in AD.^[^ ^44^ ^]^ CTF1 reportedly acts as a trophic factor for cortical neurons,^[^ ^45^ ^]^ and *CTF1^−^* expressing APPswe/PS1dE9 transgenic mice exhibited improvements in learning and memory.^[^ ^46^ ^]^ However, it remains unknown whether CTF1 mutations are associated with AD. Our data showed that *CTF1* was upregulated in all five subfields, suggesting that its function in human brains with AD may be different from that in AD‐model mice.

Degeneration of the brain is progressive and dynamic. Because of the limitations of current omics techniques, continuous observation and identification of the “initial change” or “driving force” are difficult. Furthermore, our data revealed that the transcriptome of AD exhibits highly significant pathological progression‐specific differences. Thus, we divided individuals with AD pathology into two subgroups, early‐ and late‐pathology, to identify the initial changes. Our results revealed relatively large numbers of DEGs in the five subfields in the late‐AD pathology group, but relatively few in early ‐pathology. Pathology‐specific co‐expression modules can extend the results from independent analysis of single genes to the identification of dysregulated signaling pathways. Thus, we applied MDC analysis to test the connectivity of early‐ or late‐pathology compared with the no‐pathology states. Among the early‐AD pathology‐associated modules, we highlight a light‐green LOC module (Figure 4e). Neuron‐specific gene signatures, axonogenesis, and learning or memory functional terms were enriched in this module. In the largest AD genome‐wide association studies (GWAS) to date,^[^ ^47^ ^]^ *MEF2C* was identified as an AD risk gene. MEF2C is involved in the immune response and synaptic function.^[^ ^48^ ^]^ Overexpression of *MEF2C* in a tauopathy mouse model improved cognitive flexibility.^[^ ^49^ ^]^ The identification of MEF2C as a key driver of the light‐green LOC module in the early‐pathology network may reflect impaired neuronal activities related to cognitive functions. Among the late‐AD pathology‐associated modules, we highlight a blue GOC module (Figure 4f). Microglia‐specific gene signatures and cytokine production functional terms were enriched in this module. Macrophage scavenger receptor 1 (MSR1) was one of the main receptors implicated in A*β* uptake by immune cells.^[^ ^50^ ^]^ In the human brain, MSR1 was predominantly found in microglia.^[^ ^51^ ^]^ Moreover, single‐cell RNA‐seq revealed that *MSR1* was highly expressed in microglia from patients with AD.^[^ ^52^ ^]^ In our results, MSR1 was a key modulator of the blue GOC module in the late‐pathology network. These data indicated the possibility that MSR1 is involved in the process of AD pathology by regulating microglial function.

Our transcriptomic analysis from bulk tissue identified altered networks and key genes in the hippocampal–entorhinal system of patients with AD. However, a knowledge gap remained concerning whether disease‐associated changes in the hippocampal–entorhinal system subfields were due to changes in the cellular composition of the AD samples secondary to disease neuropathology, or to changes in the intrinsic genes in the CNS cells. Thus, by applying CIBERSORTx algorithms, we identified cell proportion changes and cell‐intrinsic transcriptional alterations in the hippocampal–entorhinal system subfields of patients with AD. Our findings showed extensive neuronal loss and astrogliosis in the five subfields, regardless of the AD pathology stage (early or late). The loss of neurons was significantly positively correlated with AD pathology and negatively correlated with astrogliosis, particularly in the EC. A recent study using cortical and hippocampal post‐mortem samples demonstrated intricate interactions between the neuronal and non‐neuronal cells, such as astrocytes.^[^ ^53^ ^]^ It is conceivable that there is a regulatory mechanism that mediates the interaction between neurons and astrocytes, leading to neuronal loss and astrogliosis in the brain of patients with AD. We aimed to elucidate that mechanism by conducting cell–cell communication analyses and observed that PSAP signaling patterns were significantly elevated in the EC of brains from patients with AD compared with healthy controls.

Previous studies have suggested that PSAP plays a critical role in neuroprotection and regulation of lysosomal function.^[^ ^54^ ^]^ In addition, PSAP is a regulator of progranulin, which participates in neuroprotection in AD models.^[^ ^55^ ^]^ However, little attention has been paid to the effect of PSAP on astrocytes. Here, we show that PSAP promoted astrogliosis, and induced an A1‐like reactive astrocytes phenotype. Furthermore, PSAP‐treated astrocytes were neurotoxic and decreased synaptic functions. Taken together, these data indicate that PSAP is a mediator that induces neuronal loss and astrogliosis. Of note, we found that PSAP was mainly expressed and upregulated in neurons from AD brain, with little expression in astrocytes. Accordingly, in the AD brain, PSAP may be secreted by neurons and act on astrocytes, which in turn induce an A1‐like reactive phenotype, leading to further neuronal loss. PSAP is a 527‐amino acid protein that is degraded into four homologous sphingolipid activator proteins (saposins A–D) via proteolysis in the endosome. To date, there are no data on how PSAP functions in astrocytes. It is also unclear how neuronal PSAP contributes to the pathology of AD. Future studies using AD models are needed to elucidate the relevant molecular mechanisms in neurons and astrocytes.

## 4 Conclusion

We conducted a well‐powered transcriptomic study of human hippocampal–entorhinal system subfields in AD. We defined molecular signatures of the five subfields, as well as pathology‐specific transcriptional changes, including differentially expressed key drivers linked to the genetic risk of AD. Our findings provide strong evidence for the role of these and other key drivers in AD and will guide future functional studies in preclinical models. In addition, our results demonstrate that A1‐reactive astrocytes in the EC are involved in AD pathology and that PSAP is a key mediator for the induction of the A1 astrocyte phenotype and astrogliosis. Further studies of these phenomena will lead to the development of improved diagnostic criteria and therapeutic interventions.

## 5 Experimental Section

### Human Postmortem Brain Tissue Collection

Human brain tissue samples were obtained from the National Human Brain Bank for Development and Function, Chinese Academy of Medical Sciences, and Peking Union Medical College, Beijing, China. All donors gave informed consent for using the donated brain tissue for medical research. The brain tissues were collected from 2012 to 2022 following the international standard human brain banking procedure. A tissue cohort for CA1, CA2, CA3, CA4, and the EC was created from 22 control individuals with no pathology and 31 age‐matched individuals with AD pathology. The “ABC” score defined by the National Institute on Aging and Alzheimer's Association guidelines was used for the AD pathology assessment of each sample.^[^ ^56^ ^]^ Basic information for each sample is listed in Table S1 (Supporting Information). The PMI was defined as the number of hours between the time of death and the time the tissue samples were freshly frozen or fixed with 4% paraformaldehyde.

### Hippocampal Dissections

The entire hippocampus was removed from the fresh brain, and tissue blocks were taken at 5 mm intervals and frozen immediately in dry ice. Frozen hippocampal tissue was sectioned at 1 mm, while sectioning adjacent tissue at 14 µm. The 14 µm sections obtained from the adjacent tissue were subjected to hematoxylin–eosin staining to determine the orientation of the hippocampal subfields for dissection. CA1–CA4 and the EC were microdissected as previously described^[^ ^7^ ^]^ and then used for RNA‐Seq.

### RNA Extraction, Library Preparation, and Sequencing

Total RNA was extracted using an RNeasy Mini Kit (74106, Qiagen) following the manufacturer's instructions and RNA integrity was checked by using an Agilent Bioanalyzer 2100. Total RNA sample preparation and sequencing were performed using the VAHTS Total RNA‐Seq Library PrepKit for Illumina (NR603‐02, Vazyme). The reads were first mapped to the human genome using Bowtie2 version 2.1.0 with the reference genome and the annotation GTF file downloaded from ENSEMBL (release 79, GRCH38), and the gene expression level was estimated using RSEM v1.2.15.

### Pre‐Processing of Transcriptome Data

The genes with missing values and with an expression value of 0 in all samples were removed. Then the Quantile Normalization was performed on the matrix to account for any potential batch effects. Subsequently, surrogate variable analysis was used to calculate hidden covariates and removed their effects on gene expression by linearizing them along with the effects of Age, DRI, and RIN, and obtaining the residuals. This was done to minimize the confounding effects of these covariates and any hidden covariates on downstream analyses.

### Differential Gene Expression Analysis

DEGs in bulk RNA samples were identified using the DEseq2 R package. DEGs in deconvoluted single‐cell RNA samples were analyzed using the edgeR package. Both analyses utilized the same criteria to identify DEGs: |log_2_ fold‐change| > 0.58 and *p* < 0.05. The numbers of DEGs in each of the five subfields were displayed in a heatmap drawn using the ggplot2 R package.

### Gene Coexpression Network Analysis

The RNA‐Seq dataset from two hundred and sixty‐two CA1, CA2, CA3, CA4, and EC samples from 53 individuals were analyzed using the WGCNA algorithm. The WGCNA::blockwiseModules() function was used with the following settings for the building network: soft threshold power, 10; minimum module size, 30; deepSplit, 4; mean topological overlap matrix denominator; a signed network with partitioning about medoids respecting the dendrogram; and reassignment threshold, *p* < 0.05, with clustering completed within a single block. The modules were randomly labeled with colors for illustration purposes. Genes that did not fall inside a particular module were colored gray. The WGCNA::modulePreservation() function was used to assess network module robustness. Zsummary preservation scores were obtained using the consensus network as the template versus each other cohort tested, with 200 permutations. The network structures of certain modules were visualized using Gephi software. Key drivers were defined by connectivity with nodes in network graphs.

### GO and Cell‐Type Enrichment Analyses

Cell‐type‐specific markers were obtained from the PanglaoDB database.^[^ ^57^ ^]^ The number of identified module genes was counted for each module and a Fisher's exact test was used to test whether module genes were enriched in a cell‐type term. Enrichment results were presented using the circlize R package. GO and KEGG analyses were performed using the clusterprofiler R package, and results of GO and KEGG analyses were presented by using the ggballoonplot R package and JMP software.

### TWAS

TWAS was performed using the FUSION software.^[^ ^9^ ^]^ Single‐nucleotide polymorphism‐level summary statistics were used to calculate gene‐level associations with AD for each specific tissue with expression eQTLs information, and FUSION combined TWAS results from each tissue. The AD GWAS summary statistics were downloaded from The Psychiatric Genomics Consortium (https://www.med.unc.edu/pgc/). For TWAS analysis using the FUSION SNP‐weights, the GWAS summary statistics were changed to linkage disequilibrium‐score format using LDSC munge_stats.py. SNP weights from the hippocampus and cortex were obtained from the TWAS FUSION website (http://gusevlab.org/projects/fusion/#reference‐functional‐data). Then TWAS was performed in the FUSION software using GWAS and SNP‐weights with default settings and a strict Bonferroni‐corrected *p*‐value threshold. Manhattan plots were used to present risk genes with adjusted *p*‐values < 10^−5^ for AD in the hippocampus and cortex, respectively.

### MDC Analysis

Individuals were separated into three stages of AD—no‐pathology, early‐pathology, and late‐pathology—according to the AD pathology assessment “ABC” score. Co‐expression networks of early‐ and late‐pathology groups were respectively built using the WGCNA algorithm. Differences of networks between no‐ and early‐pathology, and no‐ and late‐pathology, were analyzed by MDC.^[^ ^21^ ^]^ Gene modules with significant gain or loss of overall gene–gene connectivity, compared with those in no‐pathology hippocampal–entorhinal tissue at an overall FDR < 0.05 were retained.

### SnRNA‐Seq Analysis

To analyze cell types in our data, raw counts with accession number GSE186538 were downloaded from the Gene Expression Omnibus (GEO) (https://www.ncbi.nlm.nih.gov/geo/). The count matrix derived from a unique molecular identifier, reflecting the single‐nucleus transcriptomes of 219058 cells from human adult hippocampal‐entorhinal subfields^[^ ^26^ ^]^ was investigated. The authors randomly sampled 8000 cells from the total, and the subsequent data were processed with the Seurat R package.^[^ ^58^ ^]^ Cells with a mitochondrial gene percentage > 10% were discarded. Gene expression was normalized based on the “NormalizeData” function, with the scaling factor equivalent to 10 000. The “FindVariableFeatures” function was further used to identify the top 2000 variable genes. After scaling all gene expression levels, linear dimensional reduction of variable genes was conducted using the “RunPCA” function. Then significant principal components (PCs) were identified using the “JackStrawPlot” function, and PC1–PC15 were used for t‐SNE to cluster the cells using the “FindClusters” function at a resolution of 0.8. Clusters containing astrocytes, endothelial cells, microglia, neuron, oligodendrocyte, and oligodendrocyte precursor cell (OPC) were identified by the expression of known cell‐type markers. To further analyze the difference in EC cell types between patients with AD and healthy people, the count matrix with accession number GSE138852 was downloaded from the GEO.^[^ ^52^ ^]^ All 13214 cells derived from control and AD EC snRNA‐Seq samples were analyzed with the Seurat R package. The analysis was similar to that for the hippocampal–entorhinal subfield data, including gene expression normalization, linear dimensional reduction, t‐SNE dimension reduction, and cluster identification. However, unlike in the above analyses, the “FindVariableFeatures” function was used to characterize variable genes with parameters x.low.cutoff = 0.0125, x.high.cutoff = 3, and y.cutoff = 0.5 as previously described.^[^ ^52^ ^]^ Clusters containing astrocytes, endothelial cells, microglia, neuron, oligodendrocyte, and OPC were identified by the expression of known cell‐type markers.

### Deconvolution with CIBERSORTx

First, the online tool CIBERSORTx^[^ ^59^ ^]^ was used to create a signature matrix from annotated hippocampal–entorhinal subfields snRNA‐seq data. The parameter “Min. Expression” was set to zero and “Replicates” was set to 100.

To estimate cell fractions from our bulk RNA data, the signature matrix and the RNA‐Seq normalized counts per million (CPM) data were subsequently uploaded. S‐mode batch correction and 100 permutations for significance analysis were applied and quantile normalization was disabled. This process led to the generation of a sample‐cell‐type matrix showing the proportion of a given cell‐type in each sample. The samples were separated by the hippocampal–entorhinal subfields. Then ANOVA was used to determine the differences in proportions among no‐, early‐, and late‐pathology samples within each subfield, and *p*‐values were adjusted with the Bonferroni test. The cell‐type fractions were correlated with the AD pathology score using Spearman's rank correlation after the samples N, I, and H were replaced by the numbers 1, 2, and 3 respectively. Then CIBERSORTx high‐resolution mode was used to impute gene expression. The signature matrix, the RNA‐Seq normalized CPM data, and the gene list were uploaded, and quantile normalization was disabled. After calculation, sample‐gene expression matrixes were obtained for each cell‐type. For astrocytes and neurons in each subfield, the gene expression levels were respectively correlated using the ABC score and everyday cognition average score^[^ ^60^ ^]^ using Spearman's rank correlation.

### Scissor Analysis

“Scissor” was a method that identified cell subpopulations that were associated with a given phenotype from single‐cell data. Scissor R package^[^ ^12^ ^]^ was used to associate AD phenotype from this bulk RNA‐seq data with annotated EC snRNA‐seq data. Scissor was run on each sample separately according to the Scissor tutorial using logistic regression. This EC RNA‐Seq normalized CPM data and sample diagnostic information (AD was encoded as 1, controls as 0) were used as phenotypic data, which were combined with snRNA‐Seq data to calculate Scissor with an alpha‐parameter of 0.028. To further explore the Scissor results in specific cell types, astrocytes, and neurons were extracted separately and reanalyzed them using Seurat and Scissor. The procedure was consistent with that used for total cell data. The difference was that the alpha‐parameters of astrocytes and neurons were 0.45 and 0.48, respectively.

### Cell–Cell Communication Analysis

Cell–cell communication analysis based on the expression of known ligand–receptor pairs in different cell types was performed using the CellChat R package.^[^ ^61^ ^]^ To investigate differences in cellular communication between patients with AD and healthy people, annotated EC snRNA‐Seq data were first separated for AD and control groups using the Seurat “SplitObject” function, and then respectively analyzed according to the CellChat workflow. All CellChatDB databases were used for analysis and the RNA raw matrix was used to compute the communication probability and infer cellular communication networks. AD and control CellChat analysis results were merged using the “mergeCellChat” function, and then the differences between AD and controls were visualized according to the standard processes of CellChat, including the interaction numbers, interaction strength, and signaling pathways between cell‐types.

### Primary Cell Culture and Astrocyte–Neuron Coculture

Sprague Dawley rats were from Beijing HFK Bio‐Technology corporation. All animal experiments were performed in accordance with a protocol approved by the Ethics Committee of the Institute of Basic Medical Sciences, China. Cortical astrocytes were prepared from postnatal day 1 rats. Briefly, rat cortexes were enzymatically digested (0.25% trypsin) at 37 °C for 15 min. The supernatant containing dissociated cells was passed through a 70‐µm nylon mesh cell strainer into a 50‐mL conical tube and centrifuged at 200 × g for 5 min to pellet the cells. The supernatant was removed, and the cells were resuspended in Dulbecco's modified Eagle's medium containing 20% fetal bovine serum and 1% penicillin–streptomycin and plated in 10‐cm dishes. Cortical neurons were isolated by microdissection from the rat's cerebral cortex. Tissue was dispersed by trypsin and DNase I digestion for 20 min at 37 °C. After digestion, the tissues were filtered with the cell strainer. Neurons were grown on poly‐D‐lysine‐coated glass coverslips in Neurobasal Medium Plus supplemented with B27, glutamine, and penicillin–streptomycin, where half of the medium was replaced every 2–3 days and cells were used day in vitro 5–7. For neurons co‐culture with astrocytes, neurons were grown on glass coverslips, while astrocytes were plated in wells, but had no contact between them, as established by Jones et al.^[^ ^62^ ^]^

### Prokaryotic Expression and Purification of Rat PSAP Protein

Rat PSAP construct with a C‐terminal His_6_ tag was cloned into the pET28a vector. His_6_‐PSAP protein was overexpressed in *Escherichia coli* and purified using Ni^2+^‐NTA affinity resin (17524802, GE Healthcare) after 0.8 mm Isopropyl *β*‐D‐thiogalactoside induction overnight at 37 °C. The elution buffer contained 500 mm Imidazole, 500 mm NaCl, 200 mm Tris, and 5% glycerol (pH 8.0). After elution, purified PSAP protein was desalted with PBS using PD‐10 Desalting Columns (17085101, GE Healthcare). The eluted and desalted PSAP proteins were detected by an anti‐PSAP antibody (10801‐1‐AP, Proteintech, 1:1000).

### Immunohistochemistry and Immunocytochemistry

Immunohistochemistry of human postmortem brain tissues was completed on 10‐µm thick paraffin sections fixed with 4% paraformaldehyde. The sections were blocked with 2% bovine serum albumin in PBS for 1 h at room temperature before applying the primary antibody overnight at 4 °C. The human brain sections were stained with anti‐NeuN (ab104224, Abcam, 1:200) for neurons, anti‐GFAP (ab4648, Abcam, 1:200) for astrocytes, and anti‐PSAP (10801‐1‐AP, Proteintech, 1:200). For proliferation experiments, sections were stained with anti‐Ki67 (ab15580, Abcam, 1:200) and EdU (C10310‐3, Cell‐light EdU Apollo488, Ribobio). To study neuronal morphology, staining was performed with anti‐MAP2 (ab254143, Abcam, 1:200). To quantify the synaptic count in neurons, cells were stained with anti‐Synapsin I (ab254349, Abcam, 1:200) and anti‐PSD95 (124011, Synaptic Systems, 1:200). Sections were washed three times for 5 min each at room temperature and incubated with DAPI(C0065, Solarbio) for 10 min. The sections were then incubated with the secondary antibodies (A32723 and A11037, ThermoFisher, 1:200) for 1 h at room temperature after incubation with primary antibodies. All sections were mounted on glass slides with fluorescent mounting medium (ZLI‐9556, ZSGB‐BIO).

### Neuronal Morphometry

For dendrites, the total dendrite length was measured, including primary dendrites and all dendritic branches. For neuronal morphology, the number of intersections was assessed by Sholl analysis.^[^ ^63^ ^]^ All measurements and 3D reconstruction of neurons were carried out using the Imaris software.

### Quantitative RT‐PCR

Reverse transcription was performed using the PrimeScript RT Master Mix Kit (Takara, RR036A) with 500 ng of RNA per reaction. PCR was then performed on a Bio‐Rad CFX96 system with 20 ng cDNA per sample in triplicate using TB Green Premix Ex Taq II (Takara, RR820A). After the PCR, the melting curves of amplified products were generated. *β*‐actin was used as the reference gene. Relative gene expression was quantified using the 2^−ΔΔCt^ method. Primer sequences are listed in Table S8 (Supporting Information).

### Synaptosome Engulfment Assay

Synaptosomes were isolated from rat brain cortex as per the protocol of the Synaptic Extraction Reagent (Syn‐PER Synaptic Protein Extraction Reagent, ThermoFisher, 87793) and conjugated with pHrodo Red, succinimidyl ester (ThermoFisher, P36600) at room temperature with gentle agitation. After incubation for 2 h, unbound pHrodo was washed out by centrifugation. Astrocytes were then incubated with 75 µg mL^−1^ pHrodo‐conjugated synaptosomes in a medium with or without PSAP protein for 48 h and imaged using a Leica TCS SP8 gated STED microscope (Leica Microsystems, Germany). For image analysis, 10 images per group were captured using a 20× objective lens from randomly selected areas of the 6‐well plate and plotted the total sum of the objects’ fluorescence intensity in the image. Data were normalized to the degree of engulfment per image.

### Electrophysiology

Whole‐cell patch‐clamp recordings were performed using cultured rat cortical neurons perfused with an isotonic saline solution (in mM: NaCl 125, NaHCO_3_ 25, NaH_2_PO_4_ 1.25, KCl 2.5, MgCl_2_ 1, CaCl_2_ 2, glucose 25) preheated to 37 °C. Patch electrodes with resistances of 2.5–3.5 MΩ were pulled from thick‐walled borosilicate glass capillaries and filled with internal solutions containing (in mm) K^+^gluconate 130, NaCl 4, EGTA 5, CaCl_2_ 0.5, HEPES 10, MgATP 4, and Na_2_GTP 0.5 (pH adjusted to 7.2 with KOH). MEPSCs were recorded in 1 mm tetrodotoxin and 100 mm picrotoxin from a holding potential of −70 mV. Data processing and analysis were performed using pClamp 10.0 (Molecular Devices) and Prism software. *P*‐values for the means plots to compare neurons with PSAP‐treated astrocytes and PBS‐treated astrocytes were calculated using a two‐tailed, unpaired *t*‐test. Kolmogorov–Smirnov test was used to calculate the *p*‐value for the cumulative amplitude plot.

### Statistical Analysis

All the statistical analyses and data presentation were performed using R (version 4.1.0) and GraphPad Prism 7.0. The Shapiro–Wilk test was performed to test the normality of data. All assays were repeated at least three times and the data were presented as mean ± S.E.M. Student's *t*‐test, Fisher's exact test, and Spearman correlation analysis were utilized in this study. Unpaired two‐tailed Student's *t*‐test was used to evaluate the statistical difference between the two groups, while one‐way ANOVA with Turkey's multiple comparisons was used for comparison between multiple groups, and multiple *t*‐test was used in Sholl analysis. *P‐*values of less than 0.05 were considered statistically significant.

## Conflict of Interest

The authors declare no conflict of interest.

## Author Contributions

D.L., J.L., and H.L. contributed equally to this work. W.G. and C.M. conceived the project and provided guidance throughout the study. D.L. designed the study and wrote the manuscript. X.W. revised the manuscript. J.L., H.L., and J.W. performed experiments. J.L. and H.L. analyzed the data. Y.X., W.Q., N.W., and X.W. were responsible for human brain dissection and staining. All authors read and approved the final manuscript.

## Supporting information

Supporting Information

Supplemental Table 1

Supplemental Table 2

Supplemental Table 3

Supplemental Table 4

Supplemental Table 5

Supplemental Table 6

Supplemental Table 7

Supplemental Table 8

## Data Availability

The data that support the findings of this study are available from the corresponding author upon reasonable request.
