# Supplementary material for: Integrative Transcriptomic Analyses of Hippocampal–Entorhinal System Subfields Identify Key Regulators in Alzheimer's Disease
Source: Adv Sci (Weinh). 2023 May 26;10(22):2300876. doi: 10.1002/advs.202300876 (PMC10401097; doi:10.1002/advs.202300876)
Supplement: Supplementary file 1 — Supporting Information [file ADVS-10-2300876-s002.pdf]

ADVANCED SCIENCE
Open Access

Supporting Information

for *Adv. Sci.*, DOI 10.1002/advs.202300876

Integrative Transcriptomic Analyses of Hippocampal–Entorhinal System Subfields Identify Key Regulators in Alzheimer's Disease

Dan Luo, Jingying Li, Hanyou Liu, Jiayu Wang, Yu Xia, Wenying Qiu, Naili Wang, Xue Wang, Xia Wang, Chao Ma* and Wei Ge**

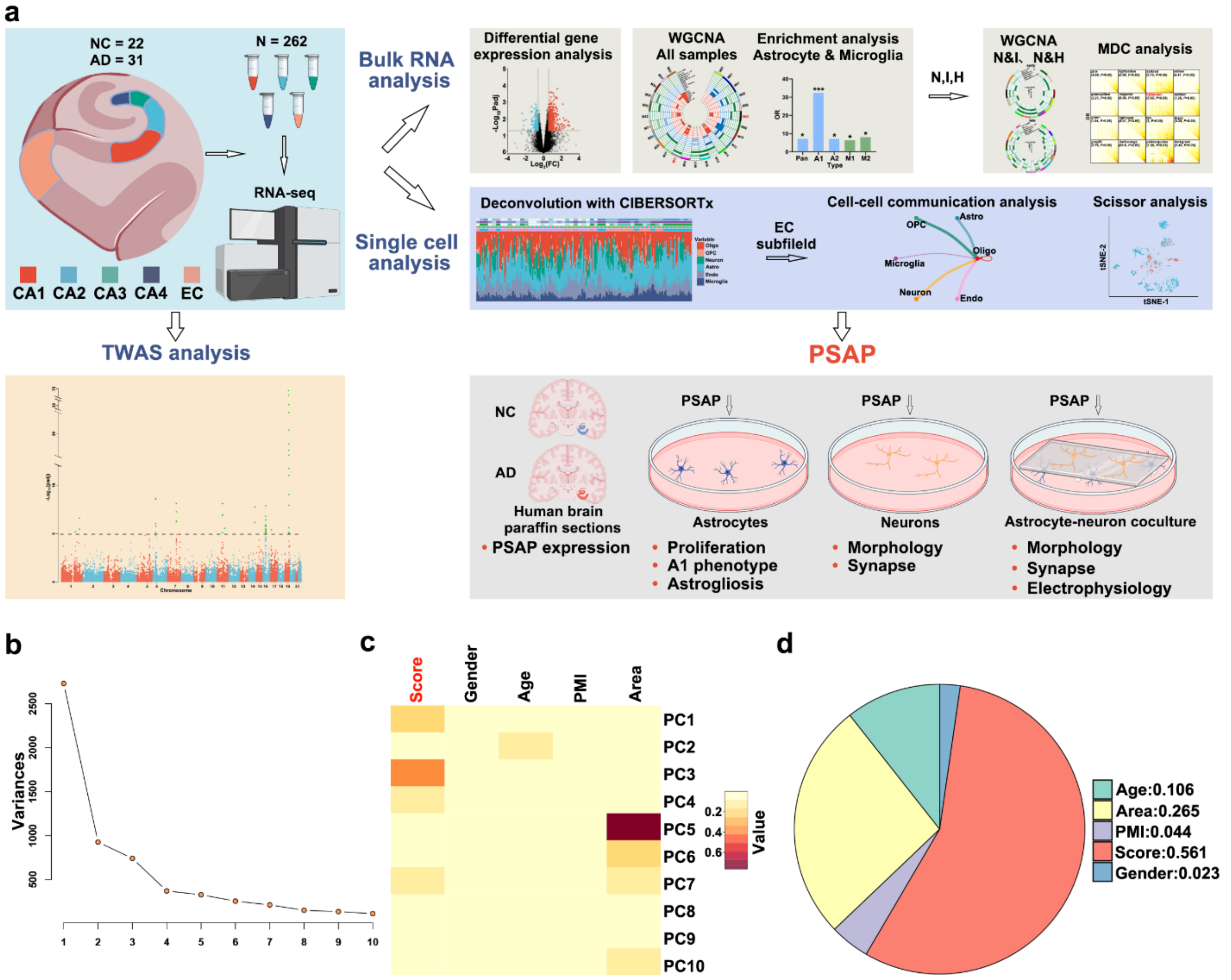

Fig. S1 Transcriptomic profiling of hippocampal–entorhinal system subfields. (a) Flowchart of this study. (b) Scree plot of top 10 principal components. (c) Heat–map of contributed variance of five covariates to the top 10 principal components. Color bar represents contributed variance. (d) Pie chart of the contribution of each covariate to the transcriptomic variance.

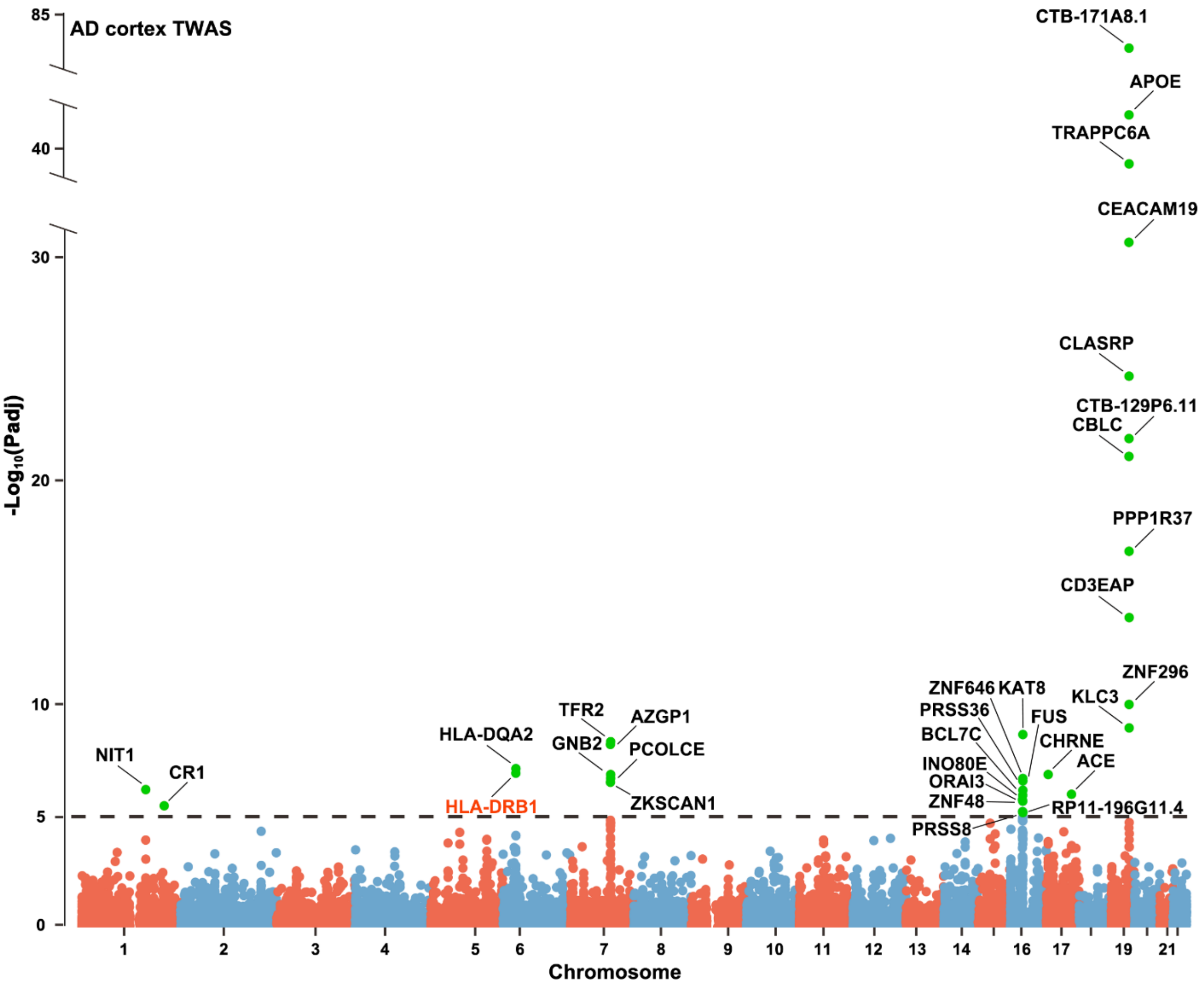

Fig. S2 Manhattan plot of TWAS by FUSION using cortex tissue data.

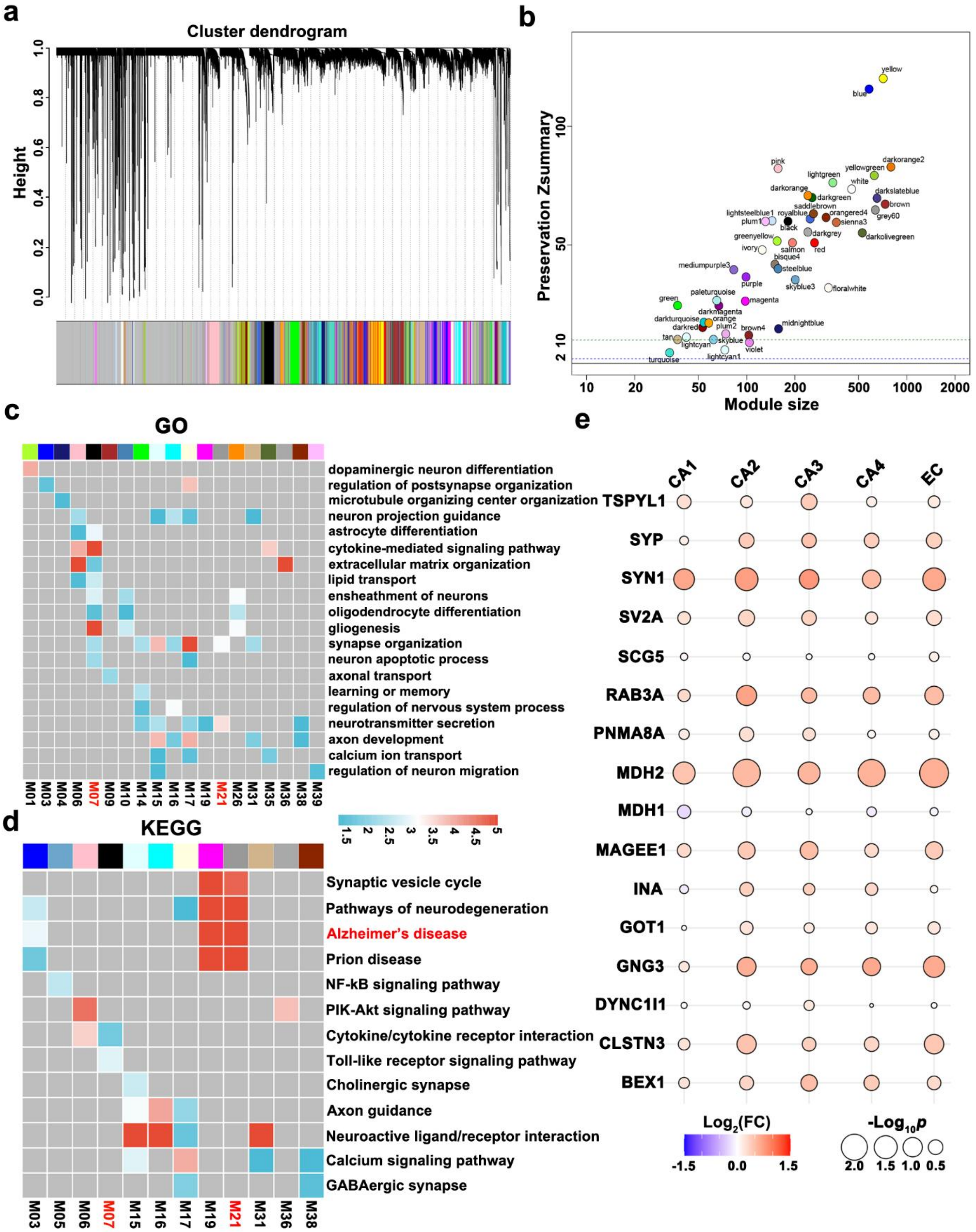

Fig. S3 Weighted gene co-expression network analysis (WGCNA) across subfields and module enrichment analysis. (a) Results of WGCNA and clustering dendrograms.

(b) Illustration of module preservation statistics. (c) Summary of module Gene Ontology (GO) enrichment results. (d) Summary of module Kyoto Encyclopedia of Genes and Genomes (KEGG) enrichment results. (e) Regional expression of hub genes in module M21, with upregulation indicated by red circles and downregulation indicated by blue circles.

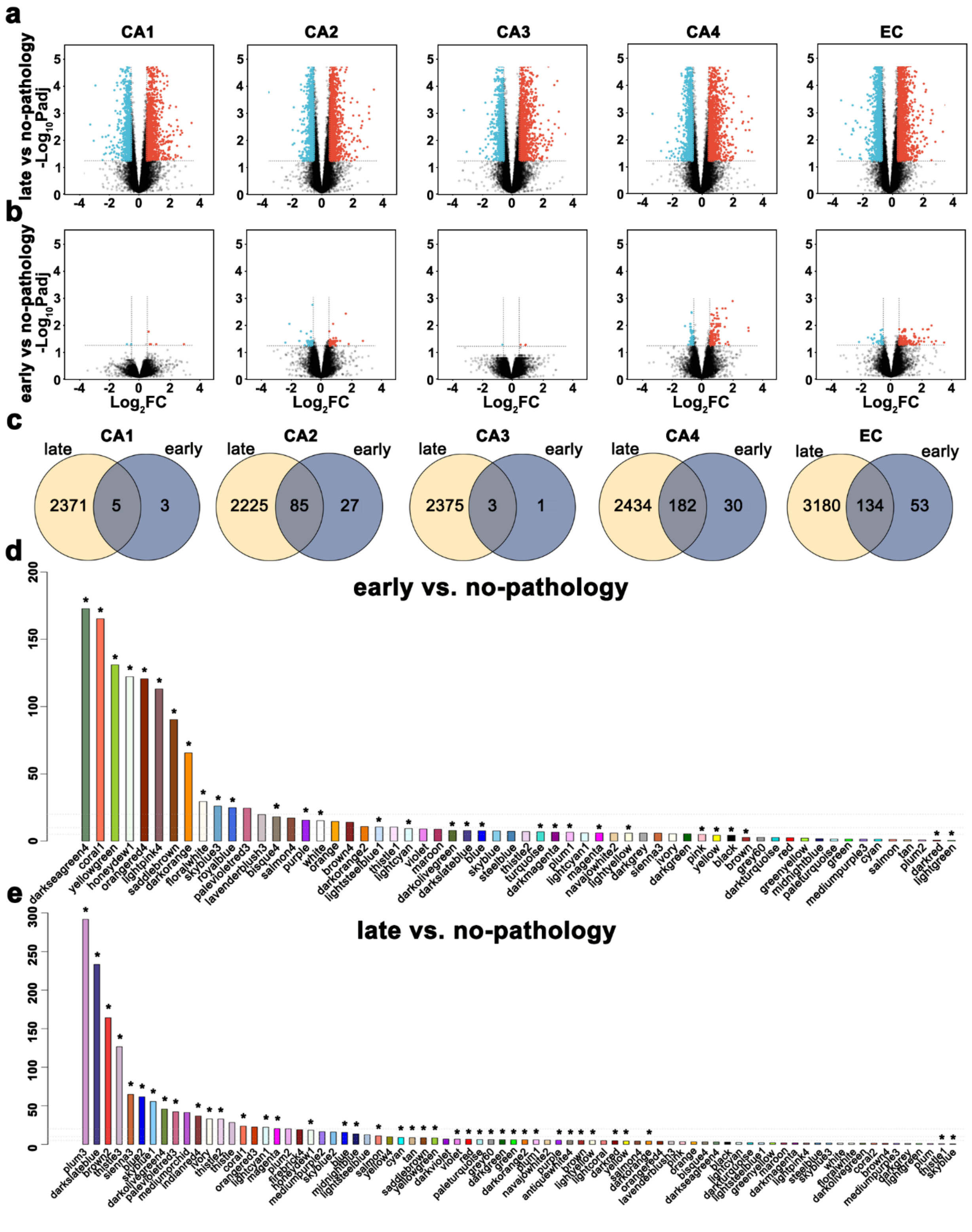

Fig. S4 Region-specific transcriptomic changes during pathological progression of AD. (a, b) Volcano plots of differentially expressed genes (DEGs) in each subfield. (a)

Late-pathology versus no-pathology; (b) early-pathology versus no-pathology. (c) Regional overlap in the number of DEGs between late-pathology (yellow) and early-pathology (grey). (d, e) Analysis of differentially connected modules in AD-pathology versus no-pathology. *FDR < 5% (d) Early-pathology versus no-pathology; (e) late-pathology versus no-pathology.

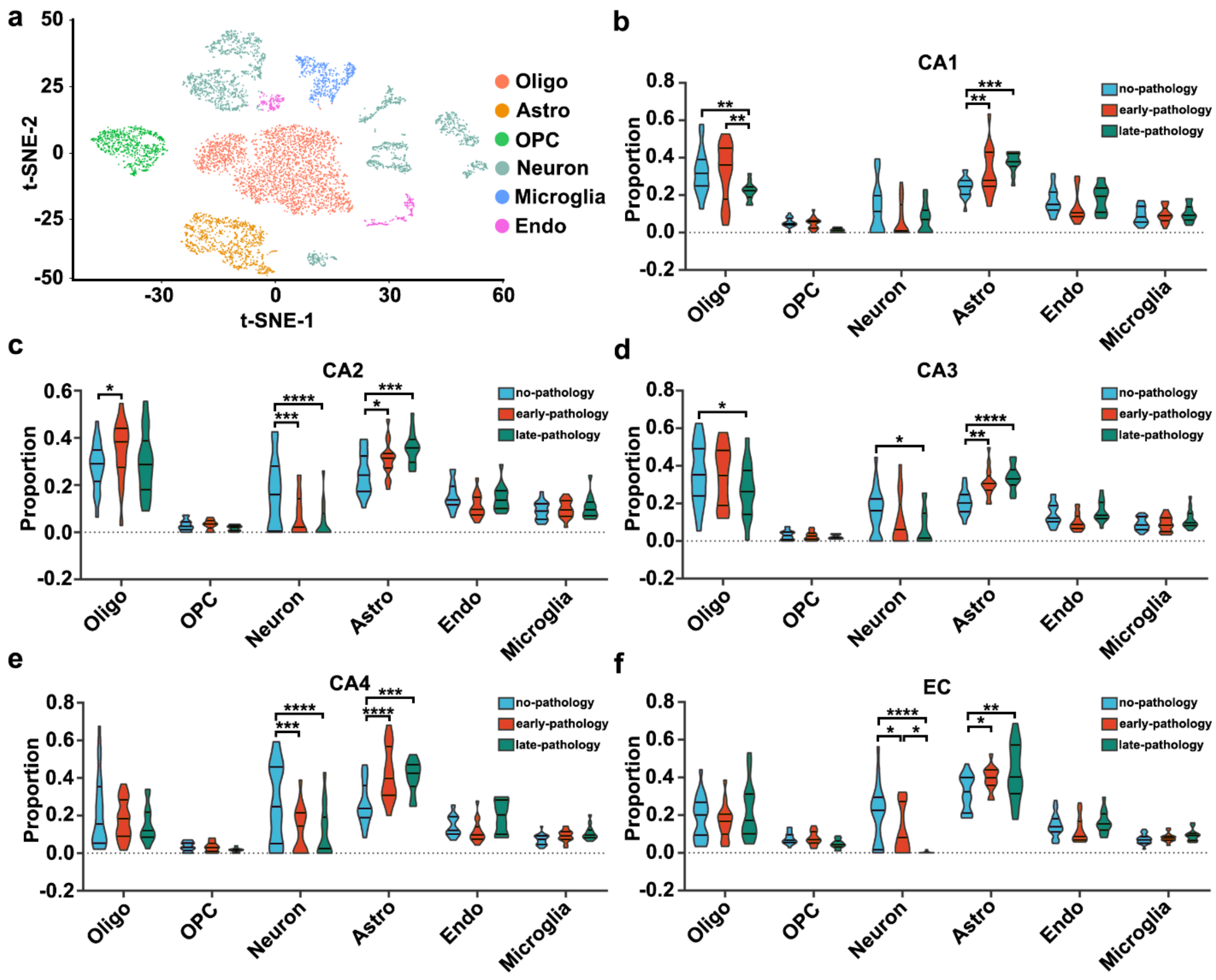

Fig. S5 Deconvolution analysis of bulk single-nucleus RNA-Seq (RNA-Seq) data. (a) *t*-distributed stochastic neighbor embedding (*t*-SNE) visualization showing clustering of single nuclei, colored by cell type. (b–f) Individual cell–type population proportions for no-, early-, and late-pathology in CA1 (b), CA2 (c), CA3 (d), CA4 (e), and the EC (f). *$p < 0.05$, **$p < 0.01$, ***$p < 0.001$, ****$p < 0.0001$, as determined by one-way ANOVA with Turkey's multiple comparisons.

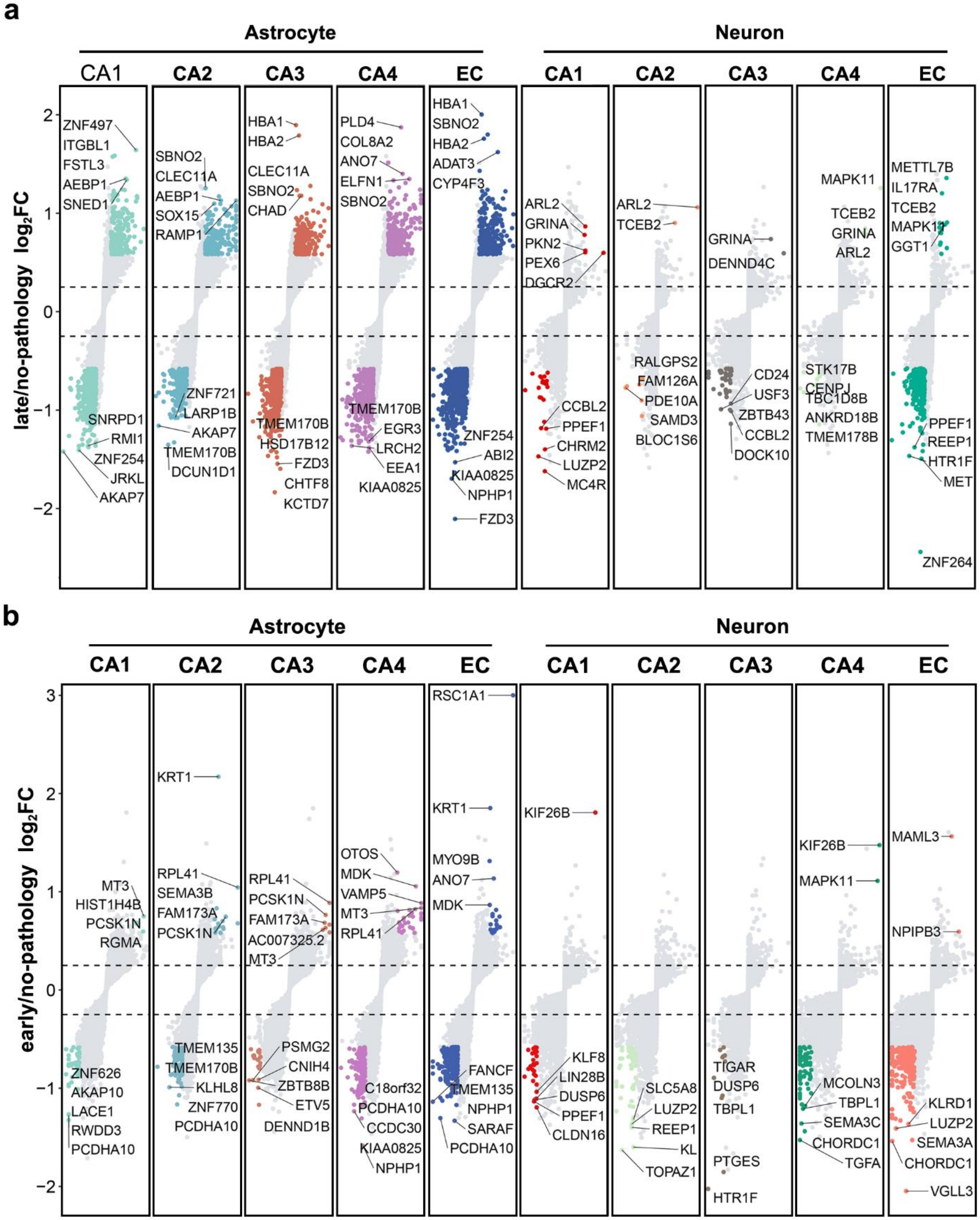

a
Astrocyte
Neuron
CA1
CA2
CA3
CA4
EC
late/no-pathology log$_2$FC
2
1
0
−1
−2
ZNF497
ITGBL1
FSTL3
AEBP1
SNED1
SNRPD1
RMI1
ZNF254
JRKL
AKAP7
SBNO2
CLEC11A
AEBP1
SOX15
RAMP1
ZNF721
LARP1B
AKAP7
TMEM170B
DCUN1D1
HBA1
HBA2
CLEC11A
SBNO2
CHAD
TMEM170B
HSD17B12
FZD3
CHTF8
KCTD7
PLD4
COL8A2
ANO7
ELFN1
SBNO2
TMEM170B
EGR3
LRCH2
EEA1
KIAA0825
HBA1
SBNO2
HBA2
ADAT3
CYP4F3
ZNF254
ABI2
KIAA0825
NPHP1
FZD3
ARL2
GRINA
PKN2
PEX6
DGCR2
CCBL2
PPEF1
CHRM2
LUZP2
MC4R
ARL2
TCEB2
RALGPS2
FAM126A
PDE10A
SAMD3
BLOC1S6
GRINA
DENND4C
CD24
USF3
ZBTB43
CCBL2
DOCK10
MAPK11
TCEB2
GRINA
ARL2
STK17B
CENPJ
TBC1D8B
ANKRD18B
TMEM178B
METTL7B
IL17RA
TCEB2
MAPK11
GGT1
PPEF1
REEP1
HTR1F
MET
ZNF264
b
Astrocyte
Neuron
CA1
CA2
CA3
CA4
EC
early/no-pathology log$_2$FC
3
2
1
0
−1
−2
MT3
HIST1H4B
PCSK1N
RGMA
ZNF626
AKAP10
LACE1
RWDD3
PCDHA10
KRT1
RPL41
SEMA3B
FAM173A
PCSK1N
TMEM135
TMEM170B
KLHL8
ZNF770
PCDHA10
RPL41
PCSK1N
FAM173A
AC007325.2
MT3
PSMG2
CNIH4
ZBTB8B
ETV5
DENND1B
OTOS
MDK
VAMP5
MT3
RPL41
C18orf32
PCDHA10
CCDC30
KIAA0825
NPHP1
RSC1A1
KRT1
MYO9B
ANO7
MDK
FANCF
TMEM135
NPHP1
SARAF
PCDHA10
KIF26B
KLF8
LIN28B
DUSP6
PPEF1
CLDN16
SLC5A8
LUZP2
REEP1
KL
TOPAZ1
TIGAR
DUSP6
TBPL1
PTGES
HTR1F
KIF26B
MAPK11
MCOLN3
TBPL1
SEMA3C
CHORDC1
TGFA
MAML3
NPIPB3
KLRD1
LUZP2
SEMA3A
CHORDC1
VGLL3

Fig. S6 The DEGs of neurons & astrocytes of AD-pathology compared with those of healthy controls. (a) Late-pathology versus no-pathology; (b) early-pathology versus no-pathology.

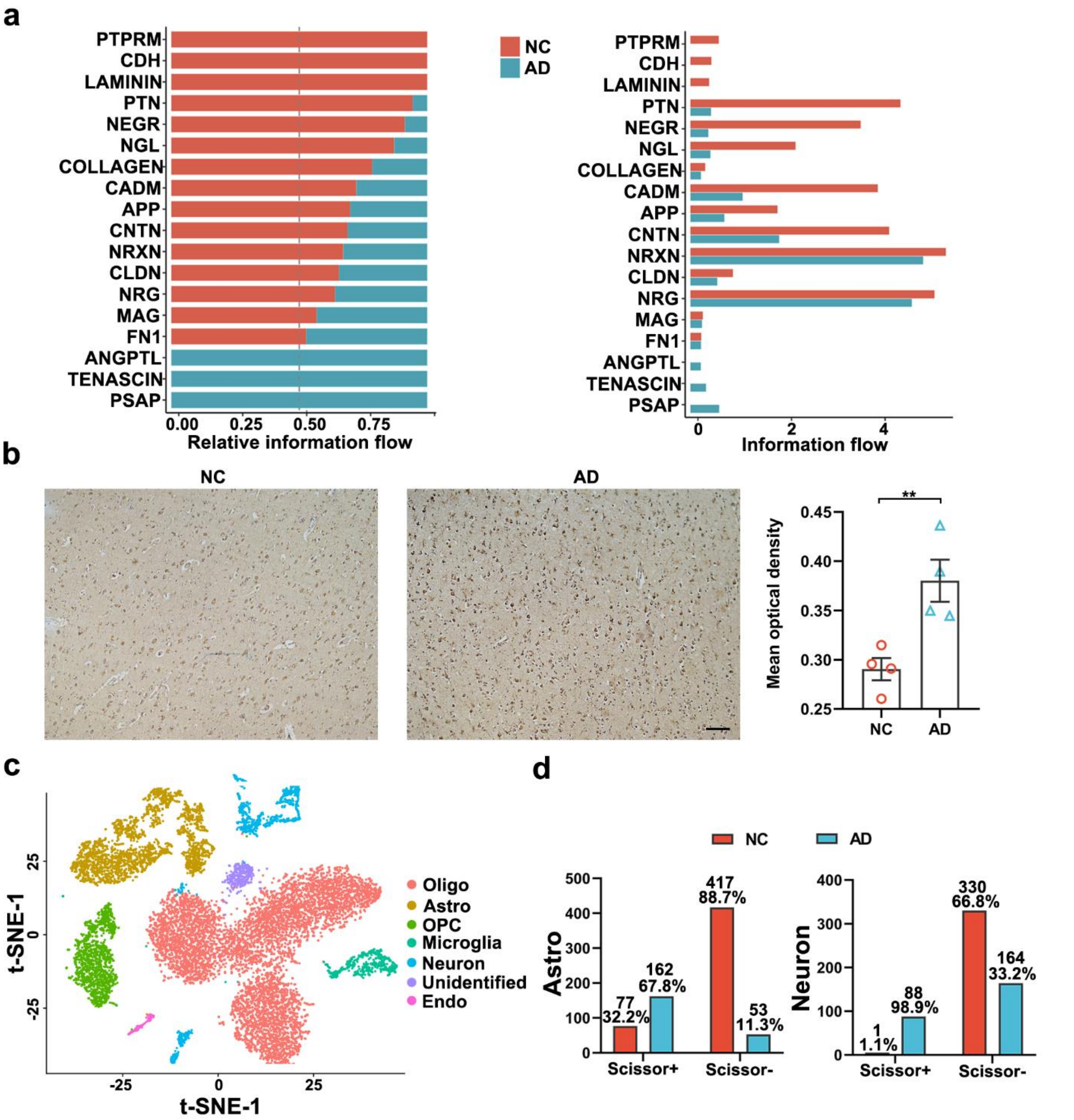

Fig. S7 (a) Bar plots ranking signaling axes by overall information flow in the interaction networks of AD and controls. (b) Representative sections of human brain hippocampus stained for PSAP. Scale bar, 100 μm. *n* = 4. Data are the mean ± S.E.M. Unpaired two-tailed Studnt's *t*-test, ***p* < 0.01 (c) *t*-SNE visualization of RNA-Seq data derived from AD

and control samples. (d) Bar plot showing counts of the phenotypes of astrocytes and neurons.

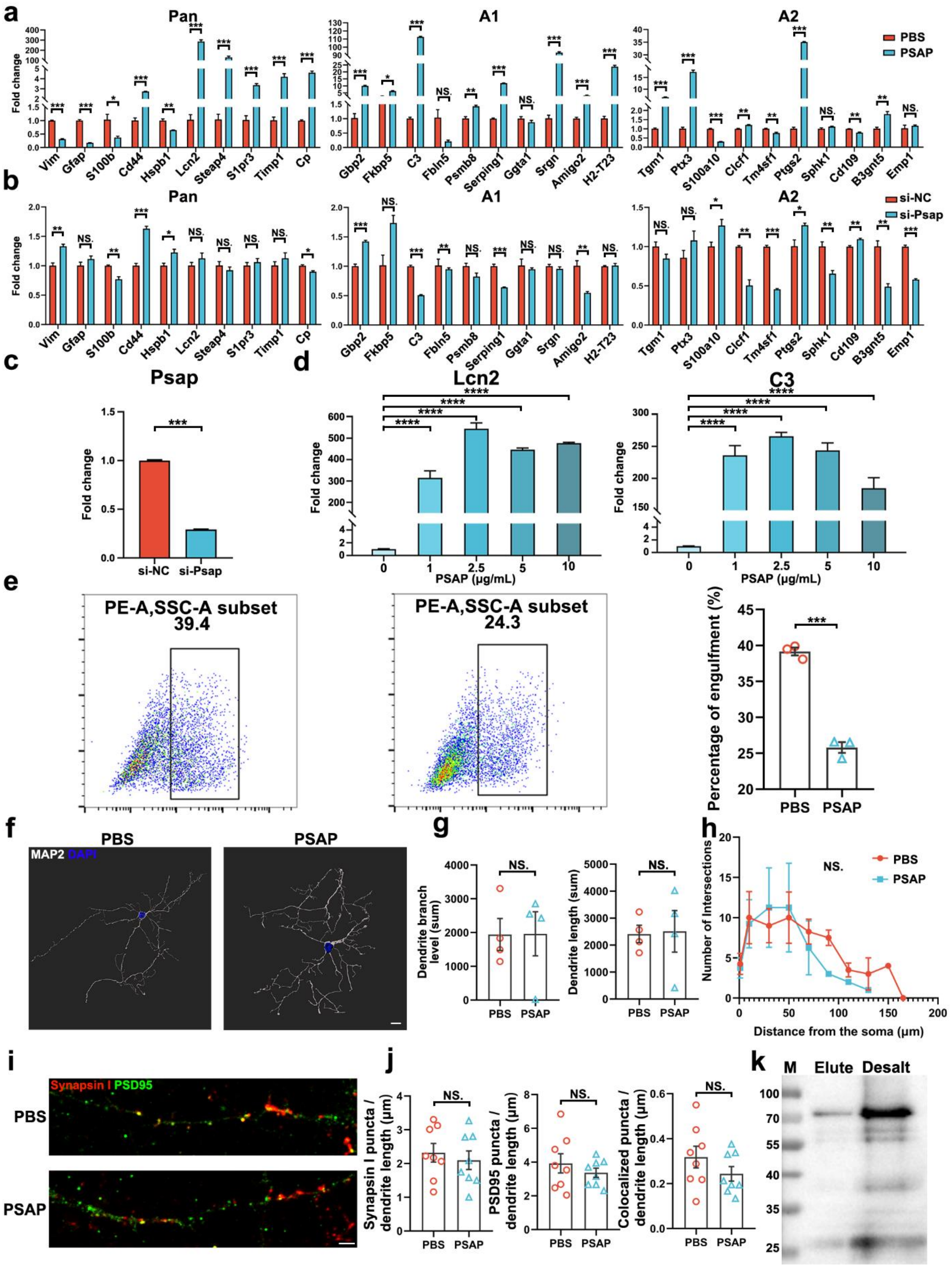

a
Pan
A1
A2
PBS
PSAP
Fold change
Vim Gfap S100b Cd44 Hspb1 Lcn2 Steap4 S1pr3 Timp1 Cp
Gbp2 Fkbp5 C3 Fbln5 Psmb8 Serping1 Ggta1 Srgn Amigo2 H2-T23
Tgm1 Ptx3 S100a10 Clcf1 Tm4sf1 Ptgs2 Sphk1 Cd109 B3gnt5 Emp1
b
si-NC
si-Psap
c
Psap
d
Lcn2
C3
PSAP (μg/mL)
e
PE-A,SSC-A subset
39.4
PE-A,SSC-A subset
24.3
Percentage of engulfment (%)
PBS
PSAP
f
MAP2 DAPI
g
Dendrite branch level (sum)
Dendrite length (sum)
h
Number of Intersections
Distance from the soma (μm)
i
Synapsin I PSD95
j
Synapsin I puncta / dendrite length (μm)
PSD95 puncta / dendrite length (μm)
Colocalize puncta / dendrite length (μm)
k
M
Elute
Desalt

Fig. S8 (a–b) Fold-change data from quantitative PCR analysis for pan- *reactive an*d A1- and A2-specific reactive transcripts in astrocytes 48 h after PSAP treatment (a) or knockdown of *PSAP* (b). (c) Quantitative PCR analysis of PSAP in negative control and si-PSAP transfected astrocytes. (d) Fold-change of Lcn2 and C3 expression in astrocytes stimulated with different concentrations (0, 1, 2.5, 5, 10 μg/mL) of PSAP for 48 hours as measured by quantitative PCR. (e) Flow cytometry analysis of astrocytes to detect the percentage of engulfment synaptosomes, $n = 3$. (f) Imaris-rendered MAP2- labeled PBS-treated or PSAP-treated neurons. (g–h) Total dendrite branches and, length, and Sholl analysis of dendritic processes, $n = 4$. (i–j) Representative images of neurons treated with PBS or PSAP, immunostained with pre- and post-synaptic markers Synapsin I (red) and PSD95 (green). Co-localization (yellow puncta) was counted as a structural synapse. $n = 8$ per group. (k) Western blot of recombinant rat PSAP protein. Scale bars, 10 μm (f, i). Data are the mean ± S.E.M. *$p < 0.05$, **$p < 0.01$, ***$p < 0.001$, ****$p < 0.0001$; NS., not significant, as determined by unpaired two-tailed Studnt's *t*-test (for two group comparison), one-way ANOVA (for multiple group comparison), or multiple *t*-test (for Sholl analysis) .
