# Supplementary material for: Integrative Transcriptomic Analyses of Hippocampal–Entorhinal System Subfields Identify Key Regulators in Alzheimer's Disease
Source: Adv Sci (Weinh). 2023 May 26;10(22):2300876. doi: 10.1002/advs.202300876 (PMC10401097; doi:10.1002/advs.202300876)
Supplement: Supplementary file 2 — Supplemental Table 1 [file ADVS-10-2300876-s005.pdf]

ADVANCED SCIENCE
Open Access

Supporting Information

for *Adv. Sci.*, DOI 10.1002/advs.202300876

Integrative Transcriptomic Analyses of Hippocampal–Entorhinal System Subfields Identify Key Regulators in Alzheimer's Disease

Dan Luo, Jingying Li, Hanyou Liu, Jiayu Wang, Yu Xia, Wenying Qiu, Naili Wang, Xue Wang, Xia Wang, Chao Ma* and Wei Ge**

Tabel S1. The characterization of AD and healthy control brain tissues.

ID	pathologic diagnosis of AD	Score	Gender	Age	PMI	RIN	Area
01	NO	N	M	79	29	5.8	CA2
01	NO	N	M	79	29	6	CA3
01	NO	N	M	79	29	6.2	CA4
01	NO	N	M	79	29	6	EC
02	YES	H	M	87	11	5.8	CA1
02	YES	H	M	87	11	5.6	CA2
02	YES	H	M	87	11	5.8	CA3
02	YES	H	M	87	11	5.8	CA4
02	YES	H	M	87	11	4.5	EC
03	YES	H	F	91	3	4.8	CA1
03	YES	H	F	91	3	5.7	CA2
03	YES	H	F	91	3	4.6	CA3
03	YES	H	F	91	3	5	CA4
03	YES	H	F	91	3	4.3	EC
04	NO	N	M	83	38	5.8	CA1
04	NO	N	M	83	38	6.7	CA2
04	NO	N	M	83	38	6.5	CA3
04	NO	N	M	83	38	5.9	CA4
04	NO	N	M	83	38	6.1	EC
05	NO	N	M	86	37.5	6.3	CA1
05	NO	N	M	86	37.5	6.6	CA2
05	NO	N	M	86	37.5	6.3	CA3
05	NO	N	M	86	37.5	5.8	CA4
05	NO	N	M	86	37.5	6	EC
06	YES	H	M	97	26.5	5.9	CA1
06	YES	H	M	97	26.5	6.1	CA2
06	YES	H	M	97	26.5	5.8	CA3
06	YES	H	M	97	26.5	5.8	CA4
06	YES	H	M	97	26.5	4.3	EC
07	NO	N	F	86	14	4.6	CA1
07	NO	N	F	86	14	6.4	CA2
07	NO	N	F	86	14	5.7	CA3
07	NO	N	F	86	14	4.8	CA4
07	NO	N	F	86	14	4.7	EC
08	NO	N	F	96	32	4.8	CA2
08	NO	N	F	96	32	5.9	CA3
08	NO	N	F	96	32	4.7	CA4
08	NO	N	F	96	32	5.7	EC
09	YES	I	F	86	8	4.1	CA1
09	YES	I	F	86	8	3.6	CA2
09	YES	I	F	86	8	4.2	CA3

09	YES	I	F	86	8	3	CA4
09	YES	I	F	86	8	3.5	EC
10	NO	N	F	86	23	6.5	CA1
10	NO	N	F	86	23	6.7	CA2
10	NO	N	F	86	23	6.5	CA3
10	NO	N	F	86	23	6.3	CA4
10	NO	N	F	86	23	6.4	EC
11	NO	N	M	75	62	2.8	CA1
11	NO	N	M	75	62	2.9	CA2
11	NO	N	M	75	62	2.7	CA3
11	NO	N	M	75	62	2.8	CA4
11	NO	N	M	75	62	2.1	EC
12	YES	I	F	84	4	6.7	CA1
12	YES	I	F	84	4	4.8	CA2
12	YES	I	F	84	4	4.8	CA3
12	YES	I	F	84	4	4.8	CA4
12	YES	I	F	84	4	6.1	EC
13	YES	I	F	86	18	5.6	CA1
13	YES	I	F	86	18	5.9	CA2
13	YES	I	F	86	18	5.8	CA3
13	YES	I	F	86	18	5.1	CA4
13	YES	I	F	86	18	5.1	EC
14	NO	N	M	62	18.5	5.2	CA1
14	NO	N	M	62	18.5	6.6	CA2
14	NO	N	M	62	18.5	6.9	CA3
14	NO	N	M	62	18.5	6.5	CA4
14	NO	N	M	62	18.5	6.2	EC
15	NO	N	F	64	10	6.1	CA1
15	NO	N	F	64	10	6.4	CA2
15	NO	N	F	64	10	5	CA3
15	NO	N	F	64	10	5.4	CA4
15	NO	N	F	64	10	5.6	EC
16	NO	N	M	64	12	5.6	CA1
16	NO	N	M	64	12	5.4	CA2
16	NO	N	M	64	12	4.5	CA3
16	NO	N	M	64	12	5.3	CA4
16	NO	N	M	64	12	6.1	EC
17	NO	N	M	86	10	5.2	CA1
17	NO	N	M	86	10	6.4	CA2
17	NO	N	M	86	10	6.1	CA3
17	NO	N	M	86	10	5.5	CA4
17	NO	N	M	86	10	5.8	EC
18	NO	N	F	89	22.5	4.8	CA1
18	NO	N	F	89	22.5	5.6	CA2

18	NO	N	F	89	22.5	4.8	CA3
18	NO	N	F	89	22.5	5.7	CA4
18	NO	N	F	89	22.5	5.9	EC
19	NO	N	M	86	20.5	6.4	CA1
19	NO	N	M	86	20.5	6.5	CA2
19	NO	N	M	86	20.5	6.1	CA3
19	NO	N	M	86	20.5	5.7	CA4
19	NO	N	M	86	20.5	6.4	EC
20	NO	N	M	86	5	4.6	CA1
20	NO	N	M	86	5	4	CA2
20	NO	N	M	86	5	4.7	CA3
20	NO	N	M	86	5	5.1	CA4
20	NO	N	M	86	5	5.1	EC
21	NO	N	M	95	5	7.6	CA1
21	NO	N	M	95	5	7.3	CA2
21	NO	N	M	95	5	7.8	CA3
21	NO	N	M	95	5	6.7	CA4
21	NO	N	M	95	5	7.3	EC
22	NO	N	M	86	14	6.2	CA1
22	NO	N	M	86	14	6.1	CA2
22	NO	N	M	86	14	5.4	CA3
22	NO	N	M	86	14	5.3	CA4
22	NO	N	M	86	14	5.5	EC
23	YES	I	M	102	46	5.3	CA1
23	YES	I	M	102	46	5.8	CA2
23	YES	I	M	102	46	6.2	CA3
23	YES	I	M	102	46	5	CA4
23	YES	I	M	102	46	4.7	EC
24	NO	N	M	71	10	3.4	CA1
24	NO	N	M	71	10	3.4	CA2
24	NO	N	M	71	10	4.6	CA3
24	NO	N	M	71	10	3.2	CA4
24	NO	N	M	71	10	2.8	EC
25	YES	I	F	91	12	3.3	CA1
25	YES	I	F	91	12	3.9	CA2
25	YES	I	F	91	12	2.6	CA3
25	YES	I	F	91	12	2.7	CA4
25	YES	I	F	91	12	2.7	EC
26	NO	N	F	74	21	6.9	CA1
26	NO	N	F	74	21	5.9	CA2
26	NO	N	F	74	21	6	CA3
26	NO	N	F	74	21	6.2	CA4
26	NO	N	F	74	21	6.7	EC
27	NO	N	F	96	9	5.4	CA1

27	NO	N	F	96	9	4.3	CA2
27	NO	N	F	96	9	5.3	CA3
27	NO	N	F	96	9	4.9	CA4
27	NO	N	F	96	9	4.7	EC
28	NO	N	M	72	39	6.2	CA1
28	NO	N	M	72	39	6.1	CA2
28	NO	N	M	72	39	5.1	CA3
28	NO	N	M	72	39	4.9	CA4
28	NO	N	M	72	39	5.8	EC
29	YES	I	M	91	16.5	3.8	CA1
29	YES	I	M	91	16.5	4.6	CA2
29	YES	I	M	91	16.5	5.2	CA3
29	YES	I	M	91	16.5	2.2	CA4
29	YES	I	M	91	16.5	3	EC
30	NO	N	M	74	28	5.2	CA2
30	NO	N	M	74	28	5.2	CA3
30	NO	N	M	74	28	4.2	CA4
30	NO	N	M	74	28	5.2	EC
31	YES	H	F	81	8	2.8	CA1
31	YES	H	F	81	8	3	CA2
31	YES	H	F	81	8	2.7	CA3
31	YES	H	F	81	8	3.1	CA4
31	YES	H	F	81	8	2.5	EC
32	YES	H	F	90	91.3	3.4	CA1
32	YES	H	F	90	91.3	4.9	CA2
32	YES	H	F	90	91.3	2.9	CA3
32	YES	H	F	90	91.3	2.6	CA4
32	YES	H	F	90	91.3	2.8	EC
33	NO	N	F	89	12.3	5.4	CA1
33	NO	N	F	89	12.3	6.1	CA2
33	NO	N	F	89	12.3	6.2	CA3
33	NO	N	F	89	12.3	6	CA4
33	NO	N	F	89	12.3	6.3	EC
34	YES	I	F	88	7	3.8	CA1
34	YES	I	F	88	7	3.4	CA2
34	YES	I	F	88	7	2.9	CA3
34	YES	I	F	88	7	3.1	CA4
34	YES	I	F	88	7	3.8	EC
35	YES	I	F	89	6.5	2.3	CA1
35	YES	I	F	89	6.5	2.5	CA2
35	YES	I	F	89	6.5	2.6	CA3
35	YES	I	F	89	6.5	2.6	CA4
35	YES	I	F	89	6.5	2.3	EC
36	YES	I	M	77	4	3.4	CA1

36	YES	I	M	77	4	3.8	CA2
36	YES	I	M	77	4	3.4	CA3
36	YES	I	M	77	4	4.2	CA4
36	YES	I	M	77	4	3.7	EC
37	YES	I	F	86	5.5	4.4	CA1
37	YES	I	F	86	5.5	3.5	CA2
37	YES	I	F	86	5.5	3.2	CA3
37	YES	I	F	86	5.5	2.9	CA4
37	YES	I	F	86	5.5	3	EC
38	YES	I	M	89	11	2.4	CA1
38	YES	I	M	89	11	2.5	CA2
38	YES	I	M	89	11	2.5	CA3
38	YES	I	M	89	11	2.3	CA4
38	YES	I	M	89	11	2.3	EC
39	YES	I	M	86	7	2.6	CA1
39	YES	I	M	86	7	3	CA2
39	YES	I	M	86	7	2.8	CA3
39	YES	I	M	86	7	2.6	CA4
39	YES	I	M	86	7	2.9	EC
40	YES	H	M	98	3	2.5	CA1
40	YES	H	M	98	3	2.6	CA2
40	YES	H	M	98	3	2.8	CA3
40	YES	H	M	98	3	3.2	CA4
40	YES	H	M	98	3	3.2	EC
41	YES	I	F	75	8	2.5	CA1
41	YES	I	F	75	8	2.8	CA2
41	YES	I	F	75	8	2.9	CA3
41	YES	I	F	75	8	2.6	CA4
41	YES	I	F	75	8	2.6	EC
42	YES	I	M	94	4.3	2.7	CA1
42	YES	I	M	94	4.3	3.1	CA2
42	YES	I	M	94	4.3	2.8	CA3
42	YES	I	M	94	4.3	2.4	CA4
42	YES	I	M	94	4.3	3	EC
43	YES	I	F	93	7.3	2.6	CA1
43	YES	I	F	93	7.3	2.6	CA2
43	YES	I	F	93	7.3	3	CA3
43	YES	I	F	93	7.3	3.8	CA4
43	YES	I	F	93	7.3	3.5	EC
44	YES	H	M	88	3.3	4	CA1
44	YES	H	M	88	3.3	2.5	CA2
44	YES	H	M	88	3.3	2.4	CA3
44	YES	H	M	88	3.3	2	CA4
44	YES	H	M	88	3.3	6.3	EC

45	YES	I	F	86	4	7.3	CA1
45	YES	I	F	86	4	7.9	CA2
45	YES	I	F	86	4	6.2	CA3
45	YES	I	F	86	4	7.1	CA4
45	YES	I	F	86	4	6.7	EC
46	YES	I	F	88	4.5	6.6	CA1
46	YES	I	F	88	4.5	6.3	CA2
46	YES	I	F	88	4.5	6.6	CA3
46	YES	I	F	88	4.5	6.2	CA4
46	YES	I	F	88	4.5	7.5	EC
47	YES	H	F	85	4.5	4.8	CA1
47	YES	H	F	85	4.5	5.7	CA2
47	YES	H	F	85	4.5	4.8	CA3
47	YES	H	F	85	4.5	4.5	CA4
47	YES	H	F	85	4.5	4.3	EC
48	YES	H	M	85	23	5.1	CA1
48	YES	H	M	85	23	4.3	CA2
48	YES	H	M	85	23	3.9	CA3
48	YES	H	M	85	23	3.5	CA4
48	YES	H	M	85	23	4.7	EC
49	YES	H	F	90	3.5	5.4	CA1
49	YES	H	F	90	3.5	5.4	CA2
49	YES	H	F	90	3.5	4.4	CA3
49	YES	H	F	90	3.5	3.2	CA4
49	YES	H	F	90	3.5	4.2	EC
50	YES	H	M	89	4.5	4.8	CA1
50	YES	H	M	89	4.5	4.9	CA2
50	YES	H	M	89	4.5	4.7	CA3
50	YES	H	M	89	4.5	4.8	CA4
50	YES	H	M	89	4.5	5	EC
51	YES	H	M	89	3	7.3	CA1
51	YES	H	M	89	3	7.5	CA2
51	YES	H	M	89	3	7.5	CA3
51	YES	H	M	89	3	6.8	CA4
51	YES	H	M	89	3	6.7	EC
52	YES	H	M	85	26	3.9	CA1
52	YES	H	M	85	26	5.4	CA2
52	YES	H	M	85	26	4.5	CA3
52	YES	H	M	85	26	4.7	CA4
52	YES	H	M	85	26	5.2	EC
53	YES	H	M	89	4.5	4.3	CA1
53	YES	H	M	89	4.5	5.7	CA2
53	YES	H	M	89	4.5	5.7	CA3
53	YES	H	M	89	4.5	5.6	CA4

53	YES	H	M	89	4.5	4.1	EC
