# Supplementary material for: Integrative Transcriptomic Analyses of Hippocampal–Entorhinal System Subfields Identify Key Regulators in Alzheimer's Disease
Source: Adv Sci (Weinh). 2023 May 26;10(22):2300876. doi: 10.1002/advs.202300876 (PMC10401097; doi:10.1002/advs.202300876)
Supplement: Supplementary file 3 — Supplemental Table 2 [file ADVS-10-2300876-s009.pdf]

ADVANCED SCIENCE
Open Access

# Supporting Information

for *Adv. Sci.*, DOI 10.1002/advs.202300876

Integrative Transcriptomic Analyses of Hippocampal–Entorhinal System Subfields Identify Key Regulators in Alzheimer's Disease

*Dan Luo, Jingying Li, Hanyou Liu, Jiayu Wang, Yu Xia, Wenying Qiu, Naili Wang, Xue Wang, Xia Wang*, Chao Ma* and Wei Ge**

**Table S2. List of differentially expressed genes (DEGs) in each subfield in AD-pathology vs. control.**

| Gene | $log_2$FoldChange | pvalue | padj | Area |
|---|---|---|---|---|
| AC024592.3 | 1.523199 | 1.02E-08 | 0.000165 | CA1 |
| NDUFC1 | 0.673119 | 6.59E-08 | 0.000268 | CA1 |
| THRA | 0.865303 | 6.29E-08 | 0.000268 | CA1 |
| UBA52 | 0.94629 | 6.15E-08 | 0.000268 | CA1 |
| CHPF | 1.116392 | 9.74E-08 | 0.000317 | CA1 |
| RPL27 | 1.16335 | 1.66E-07 | 0.00045 | CA1 |
| ALKBH7 | 0.834207 | 2.28E-07 | 0.000512 | CA1 |
| COX8A | 1.407911 | 2.52E-07 | 0.000512 | CA1 |
| RTN4R | 1.507581 | 3.51E-07 | 0.000605 | CA1 |
| ZNHIT2 | 1.328701 | 3.72E-07 | 0.000605 | CA1 |
| CSRP1 | 1.145961 | 5.38E-07 | 0.000787 | CA1 |
| SHARPIN | 0.713218 | 5.81E-07 | 0.000787 | CA1 |
| C12orf57 | 0.952421 | 8.47E-07 | 0.00106 | CA1 |
| NDUFA11 | 0.839248 | 1.10E-06 | 0.001195 | CA1 |
| ARL2 | 0.732459 | 1.08E-06 | 0.001195 | CA1 |
| SP2 | 0.75342 | 1.31E-06 | 0.001334 | CA1 |
| NOSIP | 0.7365 | 1.88E-06 | 0.001358 | CA1 |
| MZT2B | 0.917686 | 1.85E-06 | 0.001358 | CA1 |
| RRAD | 2.143557 | 2.07E-06 | 0.001358 | CA1 |
| VKORC1 | 0.886369 | 2.02E-06 | 0.001358 | CA1 |
| VAMP5 | 1.221899 | 1.86E-06 | 0.001358 | CA1 |
| MED16 | 0.733717 | 1.83E-06 | 0.001358 | CA1 |
| POLR2L | 1.161886 | 1.59E-06 | 0.001358 | CA1 |
| MAF1 | 0.723002 | 2.09E-06 | 0.001358 | CA1 |
| POM121 | 0.714126 | 1.42E-06 | 0.001358 | CA1 |
| MATR3 | -0.85707 | 2.34E-06 | 0.001363 | CA1 |
| GTPBP1 | 0.582077 | 2.35E-06 | 0.001363 | CA1 |
| DTX1 | 0.71272 | 2.23E-06 | 0.001363 | CA1 |
| PNPLA2 | 0.93389 | 2.43E-06 | 0.001365 | CA1 |
| THAP4 | 0.676796 | 3.34E-06 | 0.001814 | CA1 |
| PLTP | 1.08522 | 4.08E-06 | 0.001899 | CA1 |
| POLR2I | 0.816377 | 3.80E-06 | 0.001899 | CA1 |
| NAA38 | 1.016098 | 3.85E-06 | 0.001899 | CA1 |
| RTN4RL2 | 1.35401 | 3.79E-06 | 0.001899 | CA1 |
| PSMB10 | 0.777101 | 4.00E-06 | 0.001899 | CA1 |
| UBB | 0.814218 | 4.40E-06 | 0.001936 | CA1 |
| GRIK1 | -1.30516 | 4.32E-06 | 0.001936 | CA1 |
| PRKG2 | -0.77609 | 4.64E-06 | 0.001986 | CA1 |
| RBM42 | 0.76135 | 4.77E-06 | 0.001989 | CA1 |
| PIGT | 0.780737 | 4.92E-06 | 0.002 | CA1 |
| CLPP | 0.90697 | 5.05E-06 | 0.002005 | CA1 |

| | | | | |
|---|---|---|---|---|
| C19orf53 | 1.003771 | 5.47E-06 | 0.002031 | CA1 |
| KIAA2013 | 0.767122 | 5.54E-06 | 0.002031 | CA1 |
| RAB11B | 0.610162 | 5.37E-06 | 0.002031 | CA1 |
| RAMP1 | 1.129735 | 5.92E-06 | 0.002096 | CA1 |
| DISP3 | 1.337449 | 6.27E-06 | 0.002171 | CA1 |
| RHBDD2 | 1.063378 | 7.21E-06 | 0.002348 | CA1 |
| PCSK1N | 1.389473 | 7.18E-06 | 0.002348 | CA1 |
| CDH4 | 0.772393 | 7.71E-06 | 0.002461 | CA1 |
| MOSPD3 | 0.707982 | 8.30E-06 | 0.002531 | CA1 |
| SCN9A | -1.00289 | 8.30E-06 | 0.002531 | CA1 |
| H4C12 | 1.508005 | 9.19E-06 | 0.002631 | CA1 |
| CCDC102B | -0.79132 | 9.40E-06 | 0.002636 | CA1 |
| COX6B1 | 1.033678 | 9.74E-06 | 0.002685 | CA1 |
| COMT | 0.794361 | 1.03E-05 | 0.002795 | CA1 |
| VPS51 | 0.734009 | 1.05E-05 | 0.002795 | CA1 |
| EDF1 | 0.877974 | 1.07E-05 | 0.002817 | CA1 |
| RABAC1 | 1.018719 | 1.20E-05 | 0.00305 | CA1 |
| DUSP23 | 1.197216 | 1.23E-05 | 0.003067 | CA1 |
| TECR | 0.735793 | 1.30E-05 | 0.003154 | CA1 |
| DNAL4 | 0.954918 | 1.36E-05 | 0.003154 | CA1 |
| TBC1D8B | -0.69623 | 1.34E-05 | 0.003154 | CA1 |
| DAGLA | 0.883878 | 1.33E-05 | 0.003154 | CA1 |
| DPM2 | 0.679288 | 1.36E-05 | 0.003154 | CA1 |
| ROMO1 | 1.181886 | 1.41E-05 | 0.003168 | CA1 |
| RAD18 | -0.61749 | 1.47E-05 | 0.003191 | CA1 |
| OGDH | 0.61548 | 1.47E-05 | 0.003191 | CA1 |
| ARAF | 0.590611 | 1.61E-05 | 0.003359 | CA1 |
| VANGL2 | 0.801036 | 1.60E-05 | 0.003359 | CA1 |
| AKAP7 | -0.65333 | 1.68E-05 | 0.003371 | CA1 |
| SPNS1 | 0.630338 | 1.67E-05 | 0.003371 | CA1 |
| LSM10 | 0.769153 | 1.72E-05 | 0.003371 | CA1 |
| ESRRG | -0.81743 | 1.69E-05 | 0.003371 | CA1 |
| TIMM13 | 0.774426 | 1.80E-05 | 0.003411 | CA1 |
| NDUFAF3 | 0.709853 | 1.80E-05 | 0.003411 | CA1 |
| NCKIPSD | 0.590486 | 1.79E-05 | 0.003411 | CA1 |
| LTBP4 | 0.774878 | 1.84E-05 | 0.003417 | CA1 |
| ELOF1 | 0.89269 | 1.85E-05 | 0.003417 | CA1 |
| MGAT5B | 0.857811 | 1.91E-05 | 0.00345 | CA1 |
| H4C2 | 1.531882 | 1.90E-05 | 0.00345 | CA1 |
| ZNF408 | 0.634937 | 2.08E-05 | 0.003726 | CA1 |
| GABRE | -1.37245 | 2.17E-05 | 0.003751 | CA1 |
| CNTN6 | -1.21726 | 2.16E-05 | 0.003751 | CA1 |
| CIAO2B | 1.001778 | 2.22E-05 | 0.003804 | CA1 |
| EEFSEC | 0.656615 | 2.26E-05 | 0.003837 | CA1 |

| | | | | |
|---|---|---|---|---|
| CCDC186 | -0.74316 | 2.34E-05 | 0.003934 | CA1 |
| BLOC1S1 | 0.857532 | 2.53E-05 | 0.004154 | CA1 |
| ZNF835 | 0.806925 | 2.69E-05 | 0.004328 | CA1 |
| TMSB10 | 1.017788 | 2.90E-05 | 0.004355 | CA1 |
| MRPS24 | 0.833233 | 2.85E-05 | 0.004355 | CA1 |
| SMOX | 0.775585 | 2.74E-05 | 0.004355 | CA1 |
| GNPTG | 0.603333 | 2.78E-05 | 0.004355 | CA1 |
| HSPB2 | 0.944439 | 2.92E-05 | 0.004355 | CA1 |
| GPAA1 | 0.678351 | 2.85E-05 | 0.004355 | CA1 |
| RBM41 | -0.77218 | 3.06E-05 | 0.004458 | CA1 |
| SCAMP4 | 0.64272 | 3.07E-05 | 0.004458 | CA1 |
| NRXN2 | 0.633758 | 3.15E-05 | 0.004459 | CA1 |
| PRDX5 | 0.848786 | 3.12E-05 | 0.004459 | CA1 |
| EPN1 | 0.620054 | 3.25E-05 | 0.004465 | CA1 |
| GMPR | 1.31783 | 3.23E-05 | 0.004465 | CA1 |
| POMGNT2 | 0.78045 | 3.25E-05 | 0.004465 | CA1 |
| SCAND1 | 1.015458 | 3.27E-05 | 0.004465 | CA1 |
| NDUFB7 | 0.940542 | 3.36E-05 | 0.004479 | CA1 |
| SEPTIN1 | 1.184483 | 3.34E-05 | 0.004479 | CA1 |
| LDB1 | 0.664258 | 3.33E-05 | 0.004479 | CA1 |
| FLYWCH1 | 0.655681 | 3.45E-05 | 0.004526 | CA1 |
| NTNG1 | -0.88583 | 3.51E-05 | 0.004531 | CA1 |
| WDR83OS | 0.792066 | 3.57E-05 | 0.004571 | CA1 |
| SH3GL1 | 0.615359 | 3.60E-05 | 0.004571 | CA1 |
| STIM1 | 0.583154 | 3.63E-05 | 0.004584 | CA1 |
| ZNF575 | 0.904814 | 3.70E-05 | 0.004631 | CA1 |
| ATP6V1F | 0.784339 | 3.75E-05 | 0.004663 | CA1 |
| GSTP1 | 0.771438 | 3.85E-05 | 0.004714 | CA1 |
| CRLF1 | 1.363671 | 3.99E-05 | 0.004715 | CA1 |
| POLD2 | 0.634766 | 3.96E-05 | 0.004715 | CA1 |
| RFX1 | 0.68832 | 4.05E-05 | 0.004715 | CA1 |
| NDST4 | -2.34707 | 4.02E-05 | 0.004715 | CA1 |
| BCL9L | 0.678161 | 3.98E-05 | 0.004715 | CA1 |
| POU3F4 | 0.981012 | 4.06E-05 | 0.004715 | CA1 |
| FAU | 0.716973 | 4.39E-05 | 0.004989 | CA1 |
| TMEM205 | 0.752879 | 4.54E-05 | 0.005065 | CA1 |
| OTX1 | 1.028689 | 4.74E-05 | 0.005112 | CA1 |
| ADRA2A | 1.456883 | 4.73E-05 | 0.005112 | CA1 |
| LONP1 | 0.584692 | 4.69E-05 | 0.005112 | CA1 |
| OST4 | 0.599745 | 4.67E-05 | 0.005112 | CA1 |
| MT2A | 1.201649 | 4.96E-05 | 0.005208 | CA1 |
| MZT2A | 0.800706 | 4.89E-05 | 0.005208 | CA1 |
| MFSD13A | 0.621259 | 5.00E-05 | 0.005211 | CA1 |
| C4orf48 | 1.354126 | 5.06E-05 | 0.005235 | CA1 |

| | | | | |
|---|---|---|---|---|
| CD81 | 0.870923 | 5.22E-05 | 0.005313 | CA1 |
| C5orf49 | 1.003212 | 5.42E-05 | 0.005441 | CA1 |
| SP100 | -0.73533 | 5.60E-05 | 0.005499 | CA1 |
| LGALS1 | 0.89351 | 5.53E-05 | 0.005499 | CA1 |
| PEPD | 0.759925 | 5.58E-05 | 0.005499 | CA1 |
| NTN1 | 0.792979 | 6.02E-05 | 0.005529 | CA1 |
| NAT14 | 0.921132 | 5.90E-05 | 0.005529 | CA1 |
| PLPPR2 | 0.960909 | 6.05E-05 | 0.005529 | CA1 |
| GPR63 | -0.92941 | 5.89E-05 | 0.005529 | CA1 |
| LLGL1 | 0.732431 | 5.82E-05 | 0.005529 | CA1 |
| CELSR2 | 0.73068 | 6.08E-05 | 0.005529 | CA1 |
| RBMS1 | -0.77096 | 6.11E-05 | 0.005529 | CA1 |
| ZDHHC1 | 0.736338 | 6.12E-05 | 0.005529 | CA1 |
| OXER1 | 1.479909 | 5.74E-05 | 0.005529 | CA1 |
| PSMA7 | 0.68979 | 6.20E-05 | 0.005544 | CA1 |
| ATP6V0E1 | 0.783923 | 6.22E-05 | 0.005544 | CA1 |
| ROGDI | 0.674417 | 6.70E-05 | 0.005566 | CA1 |
| METRN | 1.008354 | 6.63E-05 | 0.005566 | CA1 |
| ZNF574 | 0.657225 | 6.63E-05 | 0.005566 | CA1 |
| LRP1 | 0.712916 | 6.66E-05 | 0.005566 | CA1 |
| PPP1R1B | 0.812667 | 6.66E-05 | 0.005566 | CA1 |
| PAMR1 | 0.854725 | 6.48E-05 | 0.005566 | CA1 |
| CRYL1 | 0.587098 | 6.70E-05 | 0.005566 | CA1 |
| DAPK3 | 0.668196 | 6.66E-05 | 0.005566 | CA1 |
| DYNLRB1 | 0.880761 | 6.89E-05 | 0.005663 | CA1 |
| ANKRD49 | -0.63713 | 6.92E-05 | 0.005663 | CA1 |
| GLIS1 | 0.902836 | 6.93E-05 | 0.005663 | CA1 |
| RFX2 | 0.79924 | 7.17E-05 | 0.005747 | CA1 |
| CYSTM1 | 0.666469 | 7.16E-05 | 0.005747 | CA1 |
| MFAP4 | 1.637954 | 7.14E-05 | 0.005747 | CA1 |
| TMEM151A | 0.862228 | 7.08E-05 | 0.005747 | CA1 |
| H4C11 | 1.438203 | 7.25E-05 | 0.005784 | CA1 |
| MAPK3 | 0.610811 | 7.39E-05 | 0.005838 | CA1 |
| NOP10 | 1.090931 | 7.39E-05 | 0.005838 | CA1 |
| FBXL14 | 0.747724 | 7.49E-05 | 0.005884 | CA1 |
| DAD1 | 0.646486 | 7.59E-05 | 0.005939 | CA1 |
| GRINA | 0.726235 | 7.94E-05 | 0.006178 | CA1 |
| IRF2BPL | 0.687961 | 8.16E-05 | 0.006249 | CA1 |
| PEF1 | 0.637213 | 8.18E-05 | 0.006249 | CA1 |
| OXR1 | -0.6262 | 8.16E-05 | 0.006249 | CA1 |
| ARHGAP21 | -0.75987 | 8.28E-05 | 0.006257 | CA1 |
| INTS5 | 0.845937 | 8.47E-05 | 0.006324 | CA1 |
| RAVER1 | 0.676217 | 8.61E-05 | 0.00636 | CA1 |
| RAB4B-EGLN2 | 0.969016 | 8.68E-05 | 0.00636 | CA1 |

| | | | | |
|---|---|---|---|---|
| SUMO3 | 0.624566 | 8.68E-05 | 0.00636 | CA1 |
| GRIK5 | 0.759369 | 8.91E-05 | 0.006476 | CA1 |
| SIGMAR1 | 0.637781 | 9.03E-05 | 0.006481 | CA1 |
| EPHB6 | 0.737189 | 9.22E-05 | 0.006524 | CA1 |
| MTCH1 | 0.647437 | 9.18E-05 | 0.006524 | CA1 |
| RSPH14 | 0.919376 | 9.48E-05 | 0.006679 | CA1 |
| PPDPF | 0.991665 | 9.58E-05 | 0.006689 | CA1 |
| AGT | 1.020776 | 9.54E-05 | 0.006689 | CA1 |
| IFI27L2 | 0.795308 | 9.76E-05 | 0.006756 | CA1 |
| PMS1 | -0.70241 | 0.0001 | 0.006781 | CA1 |
| LSM7 | 0.98709 | 9.84E-05 | 0.006781 | CA1 |
| COL4A3 | -0.92141 | 9.97E-05 | 0.006781 | CA1 |
| TMEM94 | 0.583488 | 9.96E-05 | 0.006781 | CA1 |
| FBXO2 | 0.767803 | 0.000102 | 0.006847 | CA1 |
| AC007325.2 | 1.175522 | 0.000102 | 0.006847 | CA1 |
| U2AF2 | 0.580802 | 0.000108 | 0.006864 | CA1 |
| TRIP11 | -0.7117 | 0.000107 | 0.006864 | CA1 |
| ZNF423 | 0.774598 | 0.000107 | 0.006864 | CA1 |
| CLPTM1 | 0.582184 | 0.000104 | 0.006864 | CA1 |
| LUC7L3 | -0.77226 | 0.000106 | 0.006864 | CA1 |
| MRPS2 | 0.843067 | 0.000105 | 0.006864 | CA1 |
| TMBIM6 | 1.025081 | 0.000105 | 0.006864 | CA1 |
| CHST7 | 1.042827 | 0.000106 | 0.006864 | CA1 |
| NDUFA3 | 0.765937 | 0.000106 | 0.006864 | CA1 |
| DPM3 | 0.879149 | 0.000108 | 0.006864 | CA1 |
| C11orf68 | 0.616486 | 0.000109 | 0.006884 | CA1 |
| RPS18 | 0.806663 | 0.000109 | 0.006884 | CA1 |
| PGLS | 0.681131 | 0.00011 | 0.006902 | CA1 |
| FBLN1 | 0.979364 | 0.000112 | 0.006942 | CA1 |
| RSPH9 | 1.459252 | 0.000112 | 0.006942 | CA1 |
| PLEC | 0.797282 | 0.000112 | 0.006942 | CA1 |
| INAFM1 | 0.716879 | 0.000113 | 0.006987 | CA1 |
| PMM1 | 0.630078 | 0.000115 | 0.006998 | CA1 |
| MTLN | 1.054086 | 0.000115 | 0.006998 | CA1 |
| NOTCH1 | 0.715429 | 0.000117 | 0.007077 | CA1 |
| CPNE6 | 0.849764 | 0.000121 | 0.007257 | CA1 |
| PHPT1 | 0.672773 | 0.000124 | 0.007371 | CA1 |
| PHGDH | 0.802954 | 0.000125 | 0.007407 | CA1 |
| FBXL15 | 0.742235 | 0.000127 | 0.007488 | CA1 |
| SOX15 | 1.372277 | 0.000128 | 0.007488 | CA1 |
| WDR34 | 0.799294 | 0.00013 | 0.007578 | CA1 |
| SLC39A4 | 0.949757 | 0.00013 | 0.007578 | CA1 |
| GET3 | 0.682456 | 0.000132 | 0.007656 | CA1 |
| TNC | 1.253663 | 0.000133 | 0.007671 | CA1 |

| | | | | |
|---|---|---|---|---|
| MT3 | 1.028007 | 0.000135 | 0.007723 | CA1 |
| TMEM115 | 0.627743 | 0.000136 | 0.007723 | CA1 |
| PC | 0.587711 | 0.000136 | 0.007723 | CA1 |
| ZNF480 | -0.75969 | 0.000135 | 0.007723 | CA1 |
| PNPT1 | -0.65848 | 0.000137 | 0.007788 | CA1 |
| XAB2 | 0.604653 | 0.000142 | 0.007942 | CA1 |
| JUP | 0.732982 | 0.000142 | 0.007942 | CA1 |
| UBL5 | 1.041282 | 0.000144 | 0.007995 | CA1 |
| LRRC37A | -1.10466 | 0.000147 | 0.00811 | CA1 |
| KCNJ4 | 1.039678 | 0.000148 | 0.00815 | CA1 |
| ADIRF | 1.017137 | 0.000149 | 0.008183 | CA1 |
| THAP11 | 0.793123 | 0.000154 | 0.008355 | CA1 |
| TMEM256 | 0.927066 | 0.000158 | 0.008449 | CA1 |
| DDTL | 0.729062 | 0.000162 | 0.008601 | CA1 |
| APLP1 | 0.669225 | 0.000164 | 0.008649 | CA1 |
| BEST3 | -0.88793 | 0.000166 | 0.008704 | CA1 |
| FOXP4 | 0.815609 | 0.00017 | 0.008881 | CA1 |
| NMB | 1.160736 | 0.000171 | 0.008884 | CA1 |
| EIF6 | 0.620989 | 0.000171 | 0.008884 | CA1 |
| CST3 | 0.804318 | 0.000174 | 0.008989 | CA1 |
| LRFN3 | 0.667055 | 0.000176 | 0.008989 | CA1 |
| ZNF414 | 0.600037 | 0.000175 | 0.008989 | CA1 |
| WDR38 | 2.217966 | 0.000176 | 0.008989 | CA1 |
| ZBTB8B | -0.64601 | 0.000175 | 0.008989 | CA1 |
| H4C3 | 1.00682 | 0.000181 | 0.009163 | CA1 |
| CHST1 | 0.7464 | 0.000182 | 0.009203 | CA1 |
| H2AX | 0.726762 | 0.000185 | 0.009329 | CA1 |
| SLC6A15 | -0.63045 | 0.000186 | 0.009329 | CA1 |
| ARPC4 | 0.678633 | 0.000186 | 0.009337 | CA1 |
| HEPACAM | 0.709311 | 0.000188 | 0.009374 | CA1 |
| RPL13A | 0.712047 | 0.000191 | 0.00945 | CA1 |
| MEGF8 | 0.659818 | 0.000195 | 0.009603 | CA1 |
| SLC8A2 | 1.02227 | 0.000196 | 0.009603 | CA1 |
| BCLAF1 | -0.66509 | 0.0002 | 0.00968 | CA1 |
| GAMT | 0.723885 | 0.0002 | 0.00968 | CA1 |
| GOLGA4 | -0.58671 | 0.0002 | 0.00968 | CA1 |
| ACKR3 | 0.806882 | 0.000205 | 0.009861 | CA1 |
| JMJD4 | 0.695072 | 0.000206 | 0.009877 | CA1 |
| RPL8 | 0.587061 | 0.000208 | 0.009921 | CA1 |
| AC011005.1 | 1.174201 | 0.000208 | 0.009921 | CA1 |
| FAM155B | 0.802249 | 0.00021 | 0.009968 | CA1 |
| LAMB3 | 1.19978 | 0.000219 | 0.010263 | CA1 |
| NDUFB11 | 0.684912 | 0.00022 | 0.01027 | CA1 |
| SCNM1 | 0.605187 | 0.000224 | 0.010393 | CA1 |

| | | | | |
|---|---|---|---|---|
| BAHD1 | 0.591977 | 0.000225 | 0.010428 | CA1 |
| FBXW5 | 0.634893 | 0.000229 | 0.010495 | CA1 |
| NHP2 | 0.593021 | 0.00023 | 0.010505 | CA1 |
| HLA-DRB1 | 1.788787 | 0.000231 | 0.010511 | CA1 |
| DGCR2 | 0.600024 | 0.00024 | 0.010532 | CA1 |
| ICAM5 | 1.106378 | 0.000239 | 0.010532 | CA1 |
| EHD1 | 0.669322 | 0.000237 | 0.010532 | CA1 |
| ECT2 | -0.6742 | 0.000236 | 0.010532 | CA1 |
| SLC44A2 | 0.662754 | 0.00024 | 0.010532 | CA1 |
| MSI1 | 0.602619 | 0.00024 | 0.010532 | CA1 |
| CRB2 | 1.020547 | 0.000239 | 0.010532 | CA1 |
| ADAMTS9 | -1.33327 | 0.000237 | 0.010532 | CA1 |
| RAC3 | 0.917697 | 0.000236 | 0.010532 | CA1 |
| NPTX1 | 1.225852 | 0.000235 | 0.010532 | CA1 |
| RNF26 | 0.630441 | 0.000234 | 0.010532 | CA1 |
| GTPBP6 | 0.603947 | 0.000234 | 0.010532 | CA1 |
| POMZP3 | 0.739772 | 0.000243 | 0.010602 | CA1 |
| CRIP1 | 1.546647 | 0.000248 | 0.010732 | CA1 |
| PRDX6 | 0.586375 | 0.000249 | 0.010735 | CA1 |
| PLA2R1 | -0.83001 | 0.000255 | 0.010899 | CA1 |
| NOC4L | 0.711317 | 0.000257 | 0.010983 | CA1 |
| AC134669.1 | -1.92436 | 0.000266 | 0.011256 | CA1 |
| RPS11 | 0.721464 | 0.000272 | 0.011465 | CA1 |
| MTRES1 | -0.62174 | 0.000277 | 0.01166 | CA1 |
| PLGLB1 | -0.83281 | 0.000278 | 0.01166 | CA1 |
| ARMC6 | 0.648958 | 0.000284 | 0.011808 | CA1 |
| C9orf24 | 0.985723 | 0.000284 | 0.011808 | CA1 |
| GDPD2 | 1.001332 | 0.00029 | 0.011928 | CA1 |
| TSHZ1 | 0.581077 | 0.00029 | 0.011928 | CA1 |
| ATP5ME | 0.6918 | 0.000292 | 0.011986 | CA1 |
| MDK | 1.220261 | 0.000301 | 0.012194 | CA1 |
| APEX2 | 0.697791 | 0.000303 | 0.012217 | CA1 |
| MT1E | 0.903165 | 0.000302 | 0.012217 | CA1 |
| TTC14 | -0.62862 | 0.000304 | 0.012264 | CA1 |
| MRPS18A | 0.639227 | 0.000309 | 0.012315 | CA1 |
| TCIM | -1.16352 | 0.000308 | 0.012315 | CA1 |
| GNAI2 | 0.618996 | 0.00031 | 0.012324 | CA1 |
| OFD1 | -0.59349 | 0.000312 | 0.012341 | CA1 |
| MFSD10 | 0.590861 | 0.000313 | 0.012341 | CA1 |
| GAS2L1 | 0.746103 | 0.000322 | 0.012547 | CA1 |
| ETHE1 | 0.901978 | 0.000325 | 0.012606 | CA1 |
| JOSD2 | 0.631458 | 0.000327 | 0.012606 | CA1 |
| TMEM11 | 0.624225 | 0.000326 | 0.012606 | CA1 |
| C4B | 1.513446 | 0.000326 | 0.012606 | CA1 |

| | | | | |
|---|---|---|---|---|
| CAMKK1 | 0.844877 | 0.000337 | 0.012804 | CA1 |
| QPRT | 0.81801 | 0.000335 | 0.012804 | CA1 |
| LGI4 | 0.646649 | 0.000336 | 0.012804 | CA1 |
| IRX1 | 1.18028 | 0.000336 | 0.012804 | CA1 |
| HLA-DQA1 | 2.344972 | 0.000336 | 0.012804 | CA1 |
| ABHD14A | 0.792291 | 0.000338 | 0.012804 | CA1 |
| INMT-MINDY4 | -2.21677 | 0.000335 | 0.012804 | CA1 |
| RPLP2 | 0.640699 | 0.00034 | 0.01283 | CA1 |
| DDAH2 | 0.592215 | 0.000341 | 0.01283 | CA1 |
| ZNF282 | 0.636033 | 0.000347 | 0.012947 | CA1 |
| CAPS | 1.169459 | 0.000351 | 0.012956 | CA1 |
| SENP6 | -0.62119 | 0.000355 | 0.012956 | CA1 |
| DCAKD | 0.60034 | 0.000355 | 0.012956 | CA1 |
| LAMTOR4 | 0.608287 | 0.000354 | 0.012956 | CA1 |
| S100A13 | 0.617295 | 0.00035 | 0.012956 | CA1 |
| PLD3 | 0.724774 | 0.000357 | 0.012984 | CA1 |
| SNRPN | 0.615954 | 0.00036 | 0.013033 | CA1 |
| FSTL3 | 1.005994 | 0.000365 | 0.013182 | CA1 |
| NR2F1 | 0.714106 | 0.000369 | 0.013229 | CA1 |
| HSD17B10 | 0.607576 | 0.000375 | 0.013284 | CA1 |
| FXYD1 | 0.819364 | 0.000373 | 0.013284 | CA1 |
| H2AC19 | 1.012142 | 0.000372 | 0.013284 | CA1 |
| COL4A4 | -0.95581 | 0.000379 | 0.013393 | CA1 |
| TMEM54 | 0.922523 | 0.00038 | 0.013404 | CA1 |
| POLR3G | -0.8633 | 0.000387 | 0.013533 | CA1 |
| RHPN1 | 0.74317 | 0.000387 | 0.013533 | CA1 |
| NEURL2 | 0.827189 | 0.000393 | 0.013725 | CA1 |
| ANTKMT | 0.938972 | 0.000395 | 0.013748 | CA1 |
| FURIN | 0.580985 | 0.000398 | 0.013748 | CA1 |
| TFPI | -0.98993 | 0.000402 | 0.013849 | CA1 |
| SF3B5 | 0.727657 | 0.000405 | 0.013918 | CA1 |
| DNAJC2 | -0.68351 | 0.000407 | 0.013947 | CA1 |
| MESP1 | 0.881352 | 0.000409 | 0.013991 | CA1 |
| VASN | 1.12643 | 0.000413 | 0.014083 | CA1 |
| RNPEPL1 | 0.697778 | 0.000415 | 0.014092 | CA1 |
| ZNF300 | -0.88778 | 0.000415 | 0.014092 | CA1 |
| COL12A1 | -1.27526 | 0.000423 | 0.014183 | CA1 |
| KCNA5 | 0.820632 | 0.000422 | 0.014183 | CA1 |
| PEX14 | 0.602553 | 0.000424 | 0.014183 | CA1 |
| EMX2 | 0.707409 | 0.000421 | 0.014183 | CA1 |
| NELFB | 0.584417 | 0.00042 | 0.014183 | CA1 |
| RPL37A | 0.677959 | 0.000424 | 0.014183 | CA1 |
| ZBED6 | -0.59646 | 0.000424 | 0.014183 | CA1 |
| PDE1A | -0.75141 | 0.000429 | 0.014262 | CA1 |

| | | | | |
|---|---|---|---|---|
| MEX3D | 0.71531 | 0.000434 | 0.014355 | CA1 |
| TCF7L1 | 0.722624 | 0.000437 | 0.014422 | CA1 |
| SNED1 | 0.730964 | 0.00044 | 0.014458 | CA1 |
| CAMK2N2 | 1.007236 | 0.000443 | 0.014466 | CA1 |
| CTSD | 0.865793 | 0.000447 | 0.014535 | CA1 |
| GFAP | 0.923666 | 0.000446 | 0.014535 | CA1 |
| HNRNPDL | -0.58935 | 0.000449 | 0.014544 | CA1 |
| TMEM147 | 0.794447 | 0.000451 | 0.014562 | CA1 |
| PSAP | 0.743434 | 0.000452 | 0.014562 | CA1 |
| RBM48 | -0.7166 | 0.000457 | 0.014638 | CA1 |
| PFDN2 | 0.677911 | 0.000456 | 0.014638 | CA1 |
| METTL26 | 0.607355 | 0.000464 | 0.014728 | CA1 |
| MSC | 1.011312 | 0.000469 | 0.014746 | CA1 |
| PLIN1 | 1.028107 | 0.000471 | 0.014765 | CA1 |
| TAS2R13 | -0.86274 | 0.000475 | 0.014833 | CA1 |
| MMP9 | 2.75485 | 0.000476 | 0.014834 | CA1 |
| DCTPP1 | 0.709891 | 0.00048 | 0.014863 | CA1 |
| DUSP15 | 0.753361 | 0.000481 | 0.014871 | CA1 |
| ETAA1 | -0.68545 | 0.000487 | 0.014953 | CA1 |
| ZNF296 | 0.977209 | 0.000497 | 0.015179 | CA1 |
| ZNF397 | -0.60836 | 0.000502 | 0.015254 | CA1 |
| H4C8 | 0.731776 | 0.000505 | 0.015288 | CA1 |
| SLC6A20 | 1.341526 | 0.000505 | 0.015288 | CA1 |
| MFSD3 | 0.815606 | 0.000507 | 0.015316 | CA1 |
| AGFG2 | 0.745587 | 0.00051 | 0.01532 | CA1 |
| CLSTN1 | 0.665702 | 0.00051 | 0.01532 | CA1 |
| ANKRD26 | -0.77325 | 0.000515 | 0.01538 | CA1 |
| UBXN10 | 0.912602 | 0.000513 | 0.01538 | CA1 |
| TLR10 | -1.21245 | 0.000519 | 0.015441 | CA1 |
| C4orf33 | -0.66361 | 0.000522 | 0.015508 | CA1 |
| GPC4 | 0.776627 | 0.000528 | 0.015527 | CA1 |
| NAALAD2 | -0.84129 | 0.000527 | 0.015527 | CA1 |
| TICAM1 | 0.628381 | 0.000543 | 0.015859 | CA1 |
| CNTFR | 0.742357 | 0.000544 | 0.01587 | CA1 |
| GCK | 0.986473 | 0.000555 | 0.016142 | CA1 |
| ARID4A | -0.58331 | 0.000563 | 0.016326 | CA1 |
| ARVCF | 0.657505 | 0.000572 | 0.016506 | CA1 |
| GDAP1L1 | 0.741083 | 0.000575 | 0.016547 | CA1 |
| LRP5 | 0.72702 | 0.000586 | 0.016819 | CA1 |
| SLC30A3 | 1.261545 | 0.000589 | 0.016867 | CA1 |
| ZNF600 | -0.66616 | 0.000594 | 0.016968 | CA1 |
| FSD1L | -0.76024 | 0.000612 | 0.01735 | CA1 |
| RBP1 | 0.795754 | 0.000625 | 0.017646 | CA1 |
| TMEM198 | 0.910999 | 0.000632 | 0.017756 | CA1 |

| | | | | |
|---|---|---|---|---|
| SLC22A17 | 0.79961 | 0.000644 | 0.017955 | CA1 |
| RPS15 | 0.910691 | 0.000644 | 0.017955 | CA1 |
| ZFP69 | -0.63121 | 0.000644 | 0.017955 | CA1 |
| ENTPD2 | 1.147115 | 0.000658 | 0.018077 | CA1 |
| CCND3 | 0.681392 | 0.000651 | 0.018077 | CA1 |
| TCEAL4 | -0.94929 | 0.000655 | 0.018077 | CA1 |
| CAMK1 | 0.714117 | 0.000653 | 0.018077 | CA1 |
| CD163L1 | -0.81471 | 0.000654 | 0.018077 | CA1 |
| KLHL25 | 0.661536 | 0.000658 | 0.018077 | CA1 |
| FANCM | -0.74801 | 0.000668 | 0.018253 | CA1 |
| CXXC5 | 0.757286 | 0.000671 | 0.018281 | CA1 |
| TMEM59L | 0.823622 | 0.000672 | 0.018291 | CA1 |
| KIF15 | -0.74218 | 0.000673 | 0.018291 | CA1 |
| ESF1 | -0.72418 | 0.000678 | 0.018356 | CA1 |
| CENPJ | -0.64767 | 0.0007 | 0.01871 | CA1 |
| TCF7 | 0.983111 | 0.000704 | 0.018768 | CA1 |
| MELTF | 0.747259 | 0.000709 | 0.018816 | CA1 |
| RPS2 | 0.686946 | 0.000711 | 0.018855 | CA1 |
| ST3GAL6 | -0.67545 | 0.000717 | 0.018944 | CA1 |
| RPS5 | 0.647861 | 0.000725 | 0.019055 | CA1 |
| HSPB8 | 0.778145 | 0.000732 | 0.019177 | CA1 |
| REX1BD | 0.656695 | 0.000739 | 0.01927 | CA1 |
| NCDN | 0.859582 | 0.000745 | 0.019343 | CA1 |
| GPR68 | 1.595184 | 0.000744 | 0.019343 | CA1 |
| DENND3 | -0.67407 | 0.000752 | 0.019454 | CA1 |
| PPEF1 | -0.84858 | 0.000757 | 0.019478 | CA1 |
| SCN7A | -0.7851 | 0.000755 | 0.019478 | CA1 |
| EEF1G | 0.772929 | 0.000756 | 0.019478 | CA1 |
| AKAP9 | -0.73717 | 0.000764 | 0.019599 | CA1 |
| THEM6 | 0.593788 | 0.000778 | 0.019786 | CA1 |
| ZMYM5 | -0.61145 | 0.000787 | 0.019949 | CA1 |
| NBEAL1 | -0.58901 | 0.000799 | 0.020123 | CA1 |
| FBXL7 | 0.580962 | 0.000799 | 0.020123 | CA1 |
| COPZ2 | 0.730718 | 0.000803 | 0.020145 | CA1 |
| MRPL24 | 0.63697 | 0.000803 | 0.020145 | CA1 |
| PLPP3 | 0.734142 | 0.000803 | 0.020145 | CA1 |
| MCOLN1 | 0.582509 | 0.000813 | 0.020276 | CA1 |
| UGGT2 | -0.64275 | 0.000812 | 0.020276 | CA1 |
| CITED4 | 0.92542 | 0.000818 | 0.020357 | CA1 |
| FBXW9 | 0.760194 | 0.000825 | 0.020404 | CA1 |
| ZNF382 | -0.94268 | 0.000828 | 0.020404 | CA1 |
| LRRTM4 | -0.69439 | 0.000828 | 0.020404 | CA1 |
| GLIS2 | 0.760707 | 0.000831 | 0.020415 | CA1 |
| NTN3 | 0.99122 | 0.000832 | 0.020415 | CA1 |

| | | | | |
|---|---|---|---|---|
| SLC39A1 | 0.685861 | 0.000834 | 0.020428 | CA1 |
| PDYN | 1.945414 | 0.000842 | 0.020597 | CA1 |
| MT-ND6 | -1.18391 | 0.000845 | 0.020638 | CA1 |
| NPTX2 | 1.451281 | 0.000848 | 0.020699 | CA1 |
| LRRC43 | 0.58978 | 0.000855 | 0.020759 | CA1 |
| FZD7 | 0.741099 | 0.000868 | 0.020784 | CA1 |
| AURKAIP1 | 0.598352 | 0.00086 | 0.020784 | CA1 |
| PSMB8 | 0.714336 | 0.000898 | 0.021339 | CA1 |
| MIF | 0.782542 | 0.000905 | 0.021378 | CA1 |
| SCARF2 | 0.644142 | 0.000904 | 0.021378 | CA1 |
| NPTXR | 0.981782 | 0.000916 | 0.021572 | CA1 |
| NINJ1 | 0.709419 | 0.000923 | 0.021693 | CA1 |
| ZNF891 | -0.58965 | 0.00093 | 0.02177 | CA1 |
| MFAP3 | -0.66878 | 0.000939 | 0.021783 | CA1 |
| OSR1 | 1.921102 | 0.000939 | 0.021783 | CA1 |
| KCNH2 | 0.680979 | 0.000949 | 0.021906 | CA1 |
| NKAIN1 | 0.94912 | 0.000976 | 0.022069 | CA1 |
| MADCAM1 | 0.862111 | 0.000964 | 0.022069 | CA1 |
| HAGHL | 0.685813 | 0.000977 | 0.022069 | CA1 |
| C3orf52 | -1.3344 | 0.000976 | 0.022069 | CA1 |
| ABHD8 | 0.719206 | 0.000962 | 0.022069 | CA1 |
| ZNF385D | -1.01287 | 0.000958 | 0.022069 | CA1 |
| FAM171A2 | 0.750562 | 0.000965 | 0.022069 | CA1 |
| HLA-DQB1 | 2.196445 | 0.000978 | 0.022069 | CA1 |
| RPL41 | 1.168087 | 0.00096 | 0.022069 | CA1 |
| ANKRD36 | -0.69828 | 0.000984 | 0.022159 | CA1 |
| TATDN1 | -0.6346 | 0.000985 | 0.022159 | CA1 |
| CCDC167 | 0.803691 | 0.000989 | 0.022196 | CA1 |
| ABHD12 | 0.606808 | 0.001005 | 0.02241 | CA1 |
| H2AC21 | 0.800108 | 0.001005 | 0.02241 | CA1 |
| LINGO1 | 0.861858 | 0.00101 | 0.022456 | CA1 |
| ZFPM1 | 0.787471 | 0.001013 | 0.022484 | CA1 |
| RBM44 | -0.89326 | 0.00102 | 0.022525 | CA1 |
| IPO4 | 0.886733 | 0.00104 | 0.02274 | CA1 |
| ADPRS | 0.621013 | 0.001041 | 0.02274 | CA1 |
| UBE2L6 | 0.665185 | 0.001044 | 0.02277 | CA1 |
| SRM | 0.606013 | 0.001047 | 0.02281 | CA1 |
| CHCHD2 | 0.604989 | 0.00105 | 0.022821 | CA1 |
| ZSCAN23 | -0.6609 | 0.001052 | 0.022821 | CA1 |
| DNALI1 | 0.744971 | 0.001063 | 0.023006 | CA1 |
| PBXIP1 | 0.65692 | 0.001074 | 0.023106 | CA1 |
| ZNF213 | 0.61311 | 0.001084 | 0.023151 | CA1 |
| PRAG1 | 0.624861 | 0.001086 | 0.023169 | CA1 |
| CDH6 | -0.77119 | 0.001093 | 0.02324 | CA1 |

| | | | | |
|---|---|---|---|---|
| BAIAP2 | 0.621805 | 0.001095 | 0.02324 | CA1 |
| H4C5 | 0.932185 | 0.001095 | 0.02324 | CA1 |
| TMEM184B | 0.620783 | 0.001102 | 0.023261 | CA1 |
| DRD4 | 1.021489 | 0.001109 | 0.023333 | CA1 |
| PIBF1 | -0.65888 | 0.001111 | 0.023333 | CA1 |
| NLRX1 | 0.613619 | 0.001117 | 0.023402 | CA1 |
| GCFC2 | -0.59765 | 0.001124 | 0.023469 | CA1 |
| CADM3 | 0.646954 | 0.001125 | 0.023469 | CA1 |
| RPUSD1 | 0.672312 | 0.001131 | 0.023538 | CA1 |
| OSBPL10 | -0.60515 | 0.001133 | 0.023538 | CA1 |
| HLA-DRB5 | 2.266966 | 0.00114 | 0.023597 | CA1 |
| LSM2 | 0.650891 | 0.001145 | 0.02365 | CA1 |
| MPHOSPH10 | -0.61181 | 0.001153 | 0.023768 | CA1 |
| LRFN4 | 0.62376 | 0.001154 | 0.023768 | CA1 |
| SNTA1 | 0.643882 | 0.001177 | 0.024085 | CA1 |
| CCDC82 | -0.73626 | 0.001208 | 0.024562 | CA1 |
| RAB33A | 0.803938 | 0.00121 | 0.024571 | CA1 |
| CRACR2A | -0.6442 | 0.001214 | 0.024573 | CA1 |
| ZNF503 | 0.681304 | 0.001229 | 0.024703 | CA1 |
| DNPH1 | 0.617477 | 0.001232 | 0.024712 | CA1 |
| ADAMTS8 | 0.864712 | 0.001233 | 0.024712 | CA1 |
| ZDHHC12 | 0.758457 | 0.001237 | 0.024746 | CA1 |
| ZNF717 | -0.61286 | 0.001238 | 0.024746 | CA1 |
| DDR1 | 0.596194 | 0.001252 | 0.02491 | CA1 |
| TTC9B | 0.768576 | 0.00128 | 0.025284 | CA1 |
| ZNF160 | -0.58864 | 0.001293 | 0.025469 | CA1 |
| FHL2 | 0.814511 | 0.001319 | 0.025798 | CA1 |
| KIRREL3 | 0.84119 | 0.001328 | 0.02587 | CA1 |
| TOMM7 | 0.659873 | 0.00134 | 0.025977 | CA1 |
| SHISA4 | 0.621101 | 0.001341 | 0.025977 | CA1 |
| ARHGAP39 | 0.639261 | 0.001371 | 0.0264 | CA1 |
| PRR7 | 0.784367 | 0.001385 | 0.026549 | CA1 |
| RB1CC1 | -0.58496 | 0.001389 | 0.026596 | CA1 |
| EPHB2 | 0.608342 | 0.001392 | 0.026617 | CA1 |
| CACNA1I | 0.719004 | 0.001396 | 0.026633 | CA1 |
| ARL6IP6 | 0.592551 | 0.0014 | 0.026679 | CA1 |
| RGMA | 0.659842 | 0.001409 | 0.026738 | CA1 |
| LAGE3 | 0.798692 | 0.001407 | 0.026738 | CA1 |
| ATP2A3 | 0.822659 | 0.001414 | 0.026779 | CA1 |
| ZZZ3 | -0.64809 | 0.001417 | 0.026802 | CA1 |
| SSC5D | 0.907577 | 0.00143 | 0.026849 | CA1 |
| GABRD | 1.170196 | 0.001429 | 0.026849 | CA1 |
| BRCA1 | -0.6501 | 0.001435 | 0.026897 | CA1 |
| ADGRB2 | 0.643665 | 0.001441 | 0.026983 | CA1 |

| | | | | |
|---|---|---|---|---|
| LVRN | -0.97829 | 0.001449 | 0.027071 | CA1 |
| KLF8 | -0.69316 | 0.001456 | 0.027142 | CA1 |
| TP53I11 | 0.922594 | 0.001462 | 0.027186 | CA1 |
| EFNB1 | 0.69612 | 0.001465 | 0.027216 | CA1 |
| ZGRF1 | -0.84705 | 0.001472 | 0.027257 | CA1 |
| CEP135 | -0.71341 | 0.001482 | 0.02741 | CA1 |
| CD177 | 1.763383 | 0.001495 | 0.027612 | CA1 |
| MXRA8 | 0.8042 | 0.001509 | 0.027815 | CA1 |
| MAPKAPK3 | 0.582962 | 0.001512 | 0.027826 | CA1 |
| SLC44A5 | -0.77811 | 0.001518 | 0.027872 | CA1 |
| ULK1 | 0.625185 | 0.001528 | 0.028026 | CA1 |
| TBC1D25 | 0.639652 | 0.001535 | 0.028108 | CA1 |
| C1QTNF5 | 1.116685 | 0.001547 | 0.028255 | CA1 |
| FAAH2 | -0.8253 | 0.001559 | 0.028415 | CA1 |
| GPT | 0.832443 | 0.001581 | 0.028675 | CA1 |
| KATNBL1 | -0.58653 | 0.001591 | 0.028741 | CA1 |
| ERBB2 | 0.667564 | 0.001593 | 0.028741 | CA1 |
| BMP8A | 0.651061 | 0.001598 | 0.028803 | CA1 |
| MTERF1 | -0.6983 | 0.001607 | 0.028857 | CA1 |
| TST | 0.660447 | 0.001621 | 0.029008 | CA1 |
| SFRP2 | 1.137598 | 0.001635 | 0.029142 | CA1 |
| NTSR2 | 0.993848 | 0.001635 | 0.029142 | CA1 |
| PRAF2 | 0.614075 | 0.001638 | 0.029153 | CA1 |
| PLP2 | 0.91346 | 0.001651 | 0.029365 | CA1 |
| SCN1A | -0.59574 | 0.001673 | 0.029626 | CA1 |
| SACS | -1.0807 | 0.0017 | 0.029902 | CA1 |
| S100A1 | 0.675115 | 0.0017 | 0.029902 | CA1 |
| PRRT3 | 0.607391 | 0.001712 | 0.029993 | CA1 |
| TMEM156 | -1.06177 | 0.001743 | 0.030211 | CA1 |
| PLCD3 | 0.749484 | 0.001741 | 0.030211 | CA1 |
| CLEC11A | 1.01212 | 0.001757 | 0.030287 | CA1 |
| ACY3 | 1.489494 | 0.00177 | 0.030359 | CA1 |
| CDK2AP2 | 0.738039 | 0.001771 | 0.030359 | CA1 |
| C5orf34 | -1.04733 | 0.001788 | 0.030533 | CA1 |
| SYBU | 0.647187 | 0.001817 | 0.030814 | CA1 |
| ATP6V0C | 0.589098 | 0.001828 | 0.030951 | CA1 |
| CTXN1 | 1.001683 | 0.001837 | 0.031019 | CA1 |
| TOLLIP | 0.711683 | 0.001846 | 0.031062 | CA1 |
| F8A3 | 0.584679 | 0.001857 | 0.031086 | CA1 |
| VAV3 | -0.74622 | 0.001899 | 0.031539 | CA1 |
| ZNF703 | 0.587557 | 0.001909 | 0.031661 | CA1 |
| SLC52A2 | 0.61555 | 0.001934 | 0.03195 | CA1 |
| SPATA2L | 0.603507 | 0.001937 | 0.031959 | CA1 |
| CERS1 | 0.704346 | 0.001939 | 0.031969 | CA1 |

| | | | | |
|---|---|---|---|---|
| SSC4D | 0.890165 | 0.001952 | 0.03211 | CA1 |
| NR2C1 | -0.60372 | 0.001972 | 0.032282 | CA1 |
| FBLN5 | 0.762842 | 0.00197 | 0.032282 | CA1 |
| SAC3D1 | 0.796848 | 0.002015 | 0.032729 | CA1 |
| CNTN4 | -0.92602 | 0.002029 | 0.032782 | CA1 |
| ZNF92 | -0.60412 | 0.002031 | 0.032782 | CA1 |
| UQCC3 | 0.730136 | 0.002025 | 0.032781 | CA1 |
| ZCCHC9 | -0.6389 | 0.002036 | 0.032796 | CA1 |
| XRCC2 | -0.832 | 0.002097 | 0.033438 | CA1 |
| DHRS2 | 1.780723 | 0.00211 | 0.033499 | CA1 |
| LTBP2 | 0.864844 | 0.002112 | 0.033499 | CA1 |
| PRH1 | -0.61207 | 0.00211 | 0.033499 | CA1 |
| C4A | 1.146788 | 0.00211 | 0.033499 | CA1 |
| TOMM34 | 0.805489 | 0.002157 | 0.033624 | CA1 |
| UNC5A | 0.693714 | 0.002156 | 0.033624 | CA1 |
| ITGB4 | 0.915673 | 0.00214 | 0.033624 | CA1 |
| PHYHIP | 0.774326 | 0.002131 | 0.033624 | CA1 |
| C9orf16 | 0.765216 | 0.002146 | 0.033624 | CA1 |
| RNF152 | -0.76095 | 0.002158 | 0.033624 | CA1 |
| ZNF708 | -0.62966 | 0.002135 | 0.033624 | CA1 |
| UQCR10 | 0.598551 | 0.002151 | 0.033624 | CA1 |
| RNF208 | 0.768734 | 0.002152 | 0.033624 | CA1 |
| CCDC163 | 0.994293 | 0.00213 | 0.033624 | CA1 |
| ZUP1 | -0.67382 | 0.002167 | 0.033674 | CA1 |
| AHI1 | -0.64911 | 0.002174 | 0.033758 | CA1 |
| B3GALT2 | -0.75885 | 0.002196 | 0.033894 | CA1 |
| FRAT1 | 0.68799 | 0.002215 | 0.034138 | CA1 |
| IGFL4 | -0.72167 | 0.002253 | 0.034657 | CA1 |
| DMTN | 0.624603 | 0.002258 | 0.034688 | CA1 |
| B3GALT4 | 0.598804 | 0.002264 | 0.034698 | CA1 |
| HMGN5 | -0.67807 | 0.002275 | 0.03479 | CA1 |
| CDC7 | -0.61138 | 0.002288 | 0.034853 | CA1 |
| ATP5MC1 | 0.588274 | 0.002324 | 0.035305 | CA1 |
| ANO3 | -0.81052 | 0.002331 | 0.035315 | CA1 |
| CELF3 | 0.805531 | 0.002336 | 0.035364 | CA1 |
| TIMM23B-AGAP6 | -0.67046 | 0.002347 | 0.035487 | CA1 |
| PRR19 | 1.203766 | 0.002352 | 0.03551 | CA1 |
| RXRG | 0.73436 | 0.002388 | 0.035896 | CA1 |
| FZD2 | 1.032551 | 0.002389 | 0.035896 | CA1 |
| SLC25A22 | 0.762341 | 0.002406 | 0.036051 | CA1 |
| XPA | -0.62924 | 0.002416 | 0.036127 | CA1 |
| LINGO3 | 1.032543 | 0.002424 | 0.03615 | CA1 |
| MBOAT7 | 0.60483 | 0.002452 | 0.036188 | CA1 |
| CCDC175 | -1.11468 | 0.002451 | 0.036188 | CA1 |

| | | | | |
|---|---|---|---|---|
| CDC42EP4 | 0.615243 | 0.002454 | 0.036188 | CA1 |
| ZNF624 | -0.68106 | 0.002481 | 0.036376 | CA1 |
| SH2D5 | 0.743911 | 0.002496 | 0.036455 | CA1 |
| CCKBR | 0.853804 | 0.002507 | 0.036575 | CA1 |
| NPC2 | 0.638096 | 0.002513 | 0.036575 | CA1 |
| LGR6 | 1.497853 | 0.002511 | 0.036575 | CA1 |
| KCNK12 | 0.679113 | 0.002519 | 0.036575 | CA1 |
| SP140L | -0.66893 | 0.00252 | 0.036575 | CA1 |
| MRPL53 | 0.666175 | 0.00251 | 0.036575 | CA1 |
| PLEKHH2 | -0.7124 | 0.00253 | 0.036615 | CA1 |
| ZNF638 | -0.5811 | 0.002601 | 0.037292 | CA1 |
| LOXHD1 | 1.76647 | 0.002623 | 0.037454 | CA1 |
| ZNF224 | -0.60789 | 0.002623 | 0.037454 | CA1 |
| SLC43A2 | 0.693788 | 0.002632 | 0.03747 | CA1 |
| PPP1R13L | 0.762671 | 0.00265 | 0.037657 | CA1 |
| ELFN2 | 0.722236 | 0.002665 | 0.037745 | CA1 |
| BDP1 | -0.63978 | 0.002689 | 0.037887 | CA1 |
| CHD1 | -0.59571 | 0.002727 | 0.038156 | CA1 |
| PPL | 1.079714 | 0.002754 | 0.038391 | CA1 |
| CTF1 | 0.941489 | 0.002756 | 0.038391 | CA1 |
| CASP8AP2 | -0.65722 | 0.002765 | 0.038426 | CA1 |
| CCDC122 | -0.87573 | 0.002763 | 0.038426 | CA1 |
| FAM117A | 0.730375 | 0.002796 | 0.038629 | CA1 |
| RHOG | 0.641187 | 0.002799 | 0.038629 | CA1 |
| ASAH2B | -0.7279 | 0.002796 | 0.038629 | CA1 |
| FKRP | 1.001636 | 0.00281 | 0.03868 | CA1 |
| CAPNS1 | 0.5934 | 0.002844 | 0.039025 | CA1 |
| BLVRB | 0.616371 | 0.002863 | 0.039118 | CA1 |
| SELENBP1 | 0.619628 | 0.002898 | 0.039402 | CA1 |
| SLC35E4 | 0.605325 | 0.002914 | 0.03953 | CA1 |
| A1BG | 0.595803 | 0.00293 | 0.039665 | CA1 |
| IFITM3 | 0.761491 | 0.002934 | 0.039685 | CA1 |
| PIK3R2 | 0.770533 | 0.002964 | 0.039985 | CA1 |
| BCAT1 | -0.64918 | 0.002993 | 0.040151 | CA1 |
| CEP290 | -0.79606 | 0.003016 | 0.040393 | CA1 |
| RASL11A | 0.899215 | 0.00303 | 0.040551 | CA1 |
| RPE65 | 1.98803 | 0.003039 | 0.04063 | CA1 |
| FGF12 | -0.69336 | 0.003045 | 0.040652 | CA1 |
| EMP3 | 0.842239 | 0.003063 | 0.040652 | CA1 |
| H4C6 | 1.016238 | 0.003083 | 0.040652 | CA1 |
| SLC15A3 | 0.678316 | 0.003121 | 0.04105 | CA1 |
| NDUFA1 | 0.783891 | 0.003127 | 0.041104 | CA1 |
| RESP18 | 1.232367 | 0.003137 | 0.041167 | CA1 |
| H2BC12 | 0.840096 | 0.003151 | 0.041283 | CA1 |

| | | | | |
|---|---|---|---|---|
| ELAVL4 | -0.76461 | 0.003166 | 0.041374 | CA1 |
| EVA1B | 0.935566 | 0.003192 | 0.041505 | CA1 |
| CCND1 | -0.62711 | 0.003207 | 0.04152 | CA1 |
| KCNK1 | 0.758653 | 0.003207 | 0.04152 | CA1 |
| ZC3H10 | 1.406184 | 0.003241 | 0.041553 | CA1 |
| PHYHD1 | 0.649024 | 0.003246 | 0.041553 | CA1 |
| CEP57L1 | -0.60328 | 0.003247 | 0.041553 | CA1 |
| ZFP69B | -0.70444 | 0.003268 | 0.041616 | CA1 |
| KLRC3 | -0.93135 | 0.003264 | 0.041616 | CA1 |
| ZFP36L1 | 0.651064 | 0.003275 | 0.041664 | CA1 |
| COL24A1 | -1.36836 | 0.003301 | 0.041804 | CA1 |
| POP7 | 0.697699 | 0.0033 | 0.041804 | CA1 |
| CFAP126 | 1.719393 | 0.003314 | 0.04193 | CA1 |
| VEGFD | -0.78479 | 0.003332 | 0.042095 | CA1 |
| DEDD2 | 0.626934 | 0.003351 | 0.042135 | CA1 |
| TMEM109 | 0.669453 | 0.003387 | 0.042522 | CA1 |
| CRYAB | 0.726263 | 0.003432 | 0.042936 | CA1 |
| BBC3 | 0.725642 | 0.003464 | 0.043226 | CA1 |
| FAM83F | 0.895626 | 0.003465 | 0.043226 | CA1 |
| C10orf105 | 0.955953 | 0.003467 | 0.043226 | CA1 |
| SNAPC2 | 0.629208 | 0.003485 | 0.043327 | CA1 |
| ERBIN | -0.68042 | 0.003484 | 0.043327 | CA1 |
| C1QL2 | 1.04098 | 0.003499 | 0.043404 | CA1 |
| IFITM2 | 0.924755 | 0.003504 | 0.043424 | CA1 |
| UBE2S | 0.688255 | 0.003537 | 0.043597 | CA1 |
| ELFN1 | 0.660088 | 0.003575 | 0.043867 | CA1 |
| BLM | -0.60011 | 0.003615 | 0.044159 | CA1 |
| LDOC1 | 0.656256 | 0.003635 | 0.044243 | CA1 |
| TRIL | 0.796276 | 0.003625 | 0.044243 | CA1 |
| ZNF248 | -0.60557 | 0.003708 | 0.044789 | CA1 |
| PCDHGA7 | 1.029643 | 0.003707 | 0.044789 | CA1 |
| MT-ATP6 | 1.451433 | 0.003727 | 0.044952 | CA1 |
| SLC25A10 | 0.58446 | 0.003762 | 0.045245 | CA1 |
| SLC35B2 | 0.68315 | 0.003806 | 0.045463 | CA1 |
| GALNT17 | 0.741059 | 0.003823 | 0.045546 | CA1 |
| SLC26A4 | -0.83531 | 0.003844 | 0.045655 | CA1 |
| CCDC112 | -0.6157 | 0.003857 | 0.04566 | CA1 |
| TRMT9B | -0.70287 | 0.00386 | 0.04566 | CA1 |
| TAS2R30 | -0.78728 | 0.00385 | 0.04566 | CA1 |
| FXYD5 | 0.735565 | 0.003907 | 0.045897 | CA1 |
| ANKFN1 | 0.642089 | 0.003913 | 0.045939 | CA1 |
| CRTC1 | 0.62461 | 0.003927 | 0.045942 | CA1 |
| TRMT112 | 0.592168 | 0.003927 | 0.045942 | CA1 |
| TSPAN33 | 0.619565 | 0.003938 | 0.046037 | CA1 |

| | | | | |
|---|---|---|---|---|
| FIBIN | 0.757462 | 0.004017 | 0.046789 | CA1 |
| MT1F | 0.884664 | 0.004021 | 0.046803 | CA1 |
| COX7A1 | 1.009845 | 0.004029 | 0.046858 | CA1 |
| HSD17B8 | 0.815765 | 0.004058 | 0.047069 | CA1 |
| BAZ1A | -0.61311 | 0.004067 | 0.047134 | CA1 |
| NINJ2 | 0.743835 | 0.004098 | 0.047271 | CA1 |
| OTOF | 1.845522 | 0.004118 | 0.047303 | CA1 |
| CBWD5 | -0.62122 | 0.004124 | 0.047329 | CA1 |
| SEPTIN7 | -0.68656 | 0.004179 | 0.047515 | CA1 |
| CDC42EP1 | 0.597725 | 0.004174 | 0.047515 | CA1 |
| GAL3ST3 | 0.635131 | 0.004188 | 0.047551 | CA1 |
| ANKRD20A1 | -0.9314 | 0.004193 | 0.047581 | CA1 |
| FBLL1 | 0.788544 | 0.0042 | 0.04759 | CA1 |
| ZNF254 | -0.6842 | 0.004251 | 0.047966 | CA1 |
| SLC22A4 | 0.616178 | 0.004257 | 0.047971 | CA1 |
| WNK4 | 1.407825 | 0.004284 | 0.04817 | CA1 |
| CBWD6 | -0.64351 | 0.00429 | 0.048178 | CA1 |
| DLG1 | -0.58928 | 0.004363 | 0.048426 | CA1 |
| GYPC | 0.755482 | 0.004362 | 0.048426 | CA1 |
| ZNF781 | -0.60138 | 0.004353 | 0.048426 | CA1 |
| NGFR | 1.085908 | 0.004409 | 0.048792 | CA1 |
| SEMA3B | 0.678908 | 0.004451 | 0.049 | CA1 |
| C16orf91 | 0.58334 | 0.004442 | 0.049 | CA1 |
| NDUFA2 | 0.664012 | 0.004464 | 0.049 | CA1 |
| NSRP1 | -0.645 | 0.004497 | 0.049013 | CA1 |
| SLC4A11 | 0.808256 | 0.004529 | 0.04913 | CA1 |
| CBWD3 | -0.68318 | 0.004541 | 0.049199 | CA1 |
| PTCHD4 | -0.79344 | 0.004545 | 0.049209 | CA1 |
| ZNF621 | -0.58338 | 0.004563 | 0.049333 | CA1 |
| FLT3 | -1.46341 | 0.004581 | 0.049453 | CA1 |
| CNTRL | -0.59205 | 0.004607 | 0.049587 | CA1 |
| ZDHHC22 | 0.716887 | 0.004629 | 0.049622 | CA1 |
| CHEK2 | -0.67108 | 0.00468 | 0.049905 | CA1 |
| TUBA8 | 0.938754 | 0.0047 | 0.049956 | CA1 |
| INSRR | 2.642791 | 0.00117 | 0.023971 | CA1 |
| C6 | 2.104731 | 0.001546 | 0.028255 | CA1 |
| TBX21 | 2.515443 | 0.000283 | 0.011804 | CA1 |
| FAT2 | 2.865041 | 0.001362 | 0.026297 | CA1 |
| WNT2 | 2.225015 | 0.00244 | 0.036188 | CA1 |
| TLX2 | 1.984746 | 0.00075 | 0.019434 | CA1 |
| GRIN3B | 3.259816 | 0.002955 | 0.039907 | CA1 |
| CD2 | 2.777119 | 0.000491 | 0.015037 | CA1 |
| NEUROG3 | 2.047231 | 0.001705 | 0.029933 | CA1 |
| PIWIL1 | -2.22815 | 0.003299 | 0.041804 | CA1 |

| | | | | |
|---|---|---|---|---|
| GNRH2 | 1.396629 | 0.003089 | 0.0407 | CA1 |
| CLDN3 | 2.818555 | 0.001079 | 0.023108 | CA1 |
| LPO | -1.68925 | 0.003751 | 0.045157 | CA1 |
| KCNK7 | 0.964593 | 0.004526 | 0.04913 | CA1 |
| CD7 | 1.401861 | 0.002063 | 0.03311 | CA1 |
| GPR149 | -1.72768 | 0.004314 | 0.048252 | CA1 |
| RTP1 | 1.691219 | 0.001002 | 0.022395 | CA1 |
| ZAR1 | 1.677658 | 0.004473 | 0.049 | CA1 |
| GZMM | 1.375763 | 0.004624 | 0.049622 | CA1 |
| LTB | 2.38875 | 0.001745 | 0.030211 | CA1 |
| AC008878.2 | -2.16289 | 0.001242 | 0.024759 | CA1 |
| CTAGE6 | -1.63967 | 0.004128 | 0.047331 | CA1 |
| | | | | |
| RICTOR | -0.7197 | 2.63E-12 | 4.30E-08 | CA2 |
| C19orf53 | 1.363433 | 1.54E-09 | 7.66E-06 | CA2 |
| CHPF | 1.096617 | 2.78E-09 | 7.66E-06 | CA2 |
| TBC1D8B | -0.85696 | 1.89E-09 | 7.66E-06 | CA2 |
| CCDC138 | -0.84169 | 2.82E-09 | 7.66E-06 | CA2 |
| SMARCAD1 | -0.62392 | 2.22E-09 | 7.66E-06 | CA2 |
| PNPLA2 | 0.966807 | 3.41E-09 | 7.96E-06 | CA2 |
| C12orf57 | 1.035728 | 6.91E-09 | 1.25E-05 | CA2 |
| RPL27 | 1.216734 | 6.24E-09 | 1.25E-05 | CA2 |
| DUSP15 | 0.909303 | 1.03E-08 | 1.68E-05 | CA2 |
| COX8A | 1.399356 | 1.19E-08 | 1.77E-05 | CA2 |
| PUS7L | -0.81273 | 1.89E-08 | 2.47E-05 | CA2 |
| DTX1 | 0.800891 | 2.06E-08 | 2.47E-05 | CA2 |
| RPS11 | 0.938991 | 2.12E-08 | 2.47E-05 | CA2 |
| MRPL24 | 0.906002 | 2.46E-08 | 2.67E-05 | CA2 |
| MZT2B | 1.045902 | 3.05E-08 | 2.85E-05 | CA2 |
| MTLN | 1.402703 | 2.87E-08 | 2.85E-05 | CA2 |
| RPL41 | 1.685533 | 3.14E-08 | 2.85E-05 | CA2 |
| PLEC | 0.821605 | 4.40E-08 | 3.78E-05 | CA2 |
| TESK1 | 0.780084 | 5.94E-08 | 4.41E-05 | CA2 |
| C4orf48 | 1.730238 | 5.44E-08 | 4.41E-05 | CA2 |
| TECR | 0.804605 | 8.19E-08 | 4.95E-05 | CA2 |
| LRP1 | 0.789377 | 7.22E-08 | 4.95E-05 | CA2 |
| ROMO1 | 1.306188 | 7.81E-08 | 4.95E-05 | CA2 |
| RAB11B | 0.658984 | 8.01E-08 | 4.95E-05 | CA2 |
| AC007325.2 | 1.15838 | 7.42E-08 | 4.95E-05 | CA2 |
| ASTN2 | 0.64335 | 1.35E-07 | 7.88E-05 | CA2 |
| PRAF2 | 0.896299 | 1.40E-07 | 7.88E-05 | CA2 |
| XAB2 | 0.650903 | 1.83E-07 | 8.73E-05 | CA2 |
| PIAS2 | -0.63516 | 1.89E-07 | 8.73E-05 | CA2 |
| CLPP | 0.891186 | 1.66E-07 | 8.73E-05 | CA2 |

| | | | | |
|---|---|---|---|---|
| PRDX5 | 0.924285 | 1.90E-07 | 8.73E-05 | CA2 |
| BLOC1S1 | 0.900537 | 1.93E-07 | 8.73E-05 | CA2 |
| PTPRF | 0.616724 | 1.73E-07 | 8.73E-05 | CA2 |
| POLR2L | 1.170509 | 1.71E-07 | 8.73E-05 | CA2 |
| BAX | 0.736432 | 2.13E-07 | 8.93E-05 | CA2 |
| MGAT1 | 0.813298 | 2.03E-07 | 8.93E-05 | CA2 |
| SP2 | 0.660398 | 2.12E-07 | 8.93E-05 | CA2 |
| MEA1 | 0.748877 | 2.25E-07 | 9.16E-05 | CA2 |
| RAMP1 | 1.099666 | 2.35E-07 | 9.35E-05 | CA2 |
| NDUFA3 | 0.90044 | 2.44E-07 | 9.47E-05 | CA2 |
| SLC16A7 | -1.43316 | 2.74E-07 | 0.000104 | CA2 |
| PLEKHH3 | 0.723473 | 2.85E-07 | 0.000106 | CA2 |
| PCSK1N | 1.511013 | 3.13E-07 | 0.000106 | CA2 |
| ATN1 | 0.64873 | 3.01E-07 | 0.000106 | CA2 |
| ZNF540 | -0.98338 | 3.05E-07 | 0.000106 | CA2 |
| MEX3D | 0.929336 | 3.13E-07 | 0.000106 | CA2 |
| CRAT | 0.676846 | 3.38E-07 | 0.00011 | CA2 |
| CLIP2 | 0.591305 | 3.65E-07 | 0.000111 | CA2 |
| EDF1 | 0.977182 | 3.88E-07 | 0.000111 | CA2 |
| COX6B1 | 1.076108 | 3.65E-07 | 0.000111 | CA2 |
| G6PD | 0.728325 | 3.61E-07 | 0.000111 | CA2 |
| ZFPL1 | 0.589678 | 3.76E-07 | 0.000111 | CA2 |
| TAF1D | -0.75194 | 3.77E-07 | 0.000111 | CA2 |
| NFKBIL1 | 0.681668 | 4.24E-07 | 0.000119 | CA2 |
| KIAA2013 | 0.742725 | 4.54E-07 | 0.000124 | CA2 |
| PSMB10 | 0.988617 | 4.56E-07 | 0.000124 | CA2 |
| MED16 | 0.693476 | 4.68E-07 | 0.000125 | CA2 |
| PLGLB1 | -1.0299 | 4.76E-07 | 0.000125 | CA2 |
| THAP4 | 0.691196 | 4.84E-07 | 0.000125 | CA2 |
| CRTC2 | 0.657026 | 5.07E-07 | 0.000129 | CA2 |
| LTBP3 | 0.614934 | 5.19E-07 | 0.00013 | CA2 |
| RABAC1 | 1.005988 | 5.58E-07 | 0.000138 | CA2 |
| WDR18 | 0.82576 | 5.87E-07 | 0.000143 | CA2 |
| H2AX | 0.747317 | 6.36E-07 | 0.000153 | CA2 |
| NAA38 | 1.178028 | 6.77E-07 | 0.000158 | CA2 |
| DYNLRB1 | 0.994052 | 7.14E-07 | 0.000164 | CA2 |
| ANTKMT | 1.058544 | 7.27E-07 | 0.000165 | CA2 |
| TIMM13 | 0.842588 | 7.63E-07 | 0.000166 | CA2 |
| NUDCD1 | -0.73569 | 7.57E-07 | 0.000166 | CA2 |
| LGI4 | 0.776523 | 7.50E-07 | 0.000166 | CA2 |
| CRB2 | 0.95972 | 8.71E-07 | 0.000185 | CA2 |
| RAD18 | -0.5994 | 9.70E-07 | 0.000203 | CA2 |
| VPS50 | -0.5964 | 1.07E-06 | 0.000218 | CA2 |
| ERO1B | -0.71345 | 1.25E-06 | 0.000228 | CA2 |

| | | | | |
|---|---|---|---|---|
| METRN | 1.182774 | 1.22E-06 | 0.000228 | CA2 |
| ETFB | 0.690294 | 1.25E-06 | 0.000228 | CA2 |
| UBE3A | -0.66609 | 1.18E-06 | 0.000228 | CA2 |
| BLZF1 | -0.68815 | 1.20E-06 | 0.000228 | CA2 |
| ZBTB45 | 0.806498 | 1.17E-06 | 0.000228 | CA2 |
| RPS18 | 0.91867 | 1.19E-06 | 0.000228 | CA2 |
| CHCHD10 | 1.045693 | 1.24E-06 | 0.000228 | CA2 |
| SEPTIN1 | 1.30822 | 1.34E-06 | 0.000241 | CA2 |
| WDR24 | 0.644139 | 1.37E-06 | 0.000241 | CA2 |
| ZNF37A | -0.72344 | 1.46E-06 | 0.000247 | CA2 |
| TBCB | 0.729418 | 1.47E-06 | 0.000247 | CA2 |
| DNPH1 | 0.863348 | 1.48E-06 | 0.000247 | CA2 |
| TATDN1 | -0.72368 | 1.45E-06 | 0.000247 | CA2 |
| DNAAF5 | 0.585685 | 1.55E-06 | 0.000255 | CA2 |
| KCNH2 | 0.941161 | 1.59E-06 | 0.000257 | CA2 |
| NDUFB7 | 1.042419 | 1.60E-06 | 0.000257 | CA2 |
| PSMA7 | 0.697138 | 1.70E-06 | 0.000257 | CA2 |
| CLPTM1 | 0.71153 | 1.64E-06 | 0.000257 | CA2 |
| TMEM147 | 0.856091 | 1.71E-06 | 0.000257 | CA2 |
| OTX1 | 1.136467 | 1.72E-06 | 0.000257 | CA2 |
| ADRM1 | 0.659033 | 1.62E-06 | 0.000257 | CA2 |
| DUSP23 | 1.310156 | 1.67E-06 | 0.000257 | CA2 |
| SAC3D1 | 1.043395 | 1.68E-06 | 0.000257 | CA2 |
| ZNF703 | 0.908207 | 1.68E-06 | 0.000257 | CA2 |
| ABHD13 | -0.8105 | 1.76E-06 | 0.000261 | CA2 |
| LSM8 | -0.64176 | 1.78E-06 | 0.000261 | CA2 |
| CHMP6 | 0.66862 | 1.86E-06 | 0.000271 | CA2 |
| RNASEK-C17orf49 | 1.081849 | 1.89E-06 | 0.000273 | CA2 |
| GTPBP6 | 0.753201 | 1.95E-06 | 0.000277 | CA2 |
| VASN | 1.13655 | 2.04E-06 | 0.000286 | CA2 |
| NDUFA11 | 0.742584 | 2.05E-06 | 0.000286 | CA2 |
| LONP1 | 0.666217 | 2.07E-06 | 0.000286 | CA2 |
| GNPTG | 0.586215 | 2.10E-06 | 0.000289 | CA2 |
| ATR | -0.58203 | 2.15E-06 | 0.000292 | CA2 |
| FAU | 0.799243 | 2.19E-06 | 0.000293 | CA2 |
| ZNF286A | -0.68924 | 2.25E-06 | 0.000299 | CA2 |
| HAUS3 | -0.7353 | 2.29E-06 | 0.000302 | CA2 |
| SOD3 | 1.472522 | 2.41E-06 | 0.000309 | CA2 |
| FASN | 0.716135 | 2.42E-06 | 0.000309 | CA2 |
| RPL37A | 0.81769 | 2.42E-06 | 0.000309 | CA2 |
| HDX | -0.62651 | 2.49E-06 | 0.000316 | CA2 |
| CPZ | 3.485657 | 2.54E-06 | 0.000319 | CA2 |
| RNPEPL1 | 0.780582 | 2.58E-06 | 0.000319 | CA2 |
| ZYX | 0.672334 | 2.57E-06 | 0.000319 | CA2 |

| | | | | |
|---|---|---|---|---|
| MICU3 | -0.80876 | 2.70E-06 | 0.000329 | CA2 |
| PTBP3 | -0.69169 | 2.79E-06 | 0.000335 | CA2 |
| CIAO2B | 1.018468 | 2.83E-06 | 0.000335 | CA2 |
| SF3B5 | 0.983903 | 2.82E-06 | 0.000335 | CA2 |
| ZNF408 | 0.686108 | 3.02E-06 | 0.000354 | CA2 |
| AIP | 0.640124 | 3.06E-06 | 0.000357 | CA2 |
| PUS10 | -0.87201 | 3.20E-06 | 0.000365 | CA2 |
| ADORA1 | 0.893401 | 3.18E-06 | 0.000365 | CA2 |
| SCAND1 | 1.100835 | 3.22E-06 | 0.000365 | CA2 |
| TMEM94 | 0.647493 | 3.35E-06 | 0.000374 | CA2 |
| CASD1 | -0.62903 | 3.47E-06 | 0.000377 | CA2 |
| BCL9L | 0.660241 | 3.45E-06 | 0.000377 | CA2 |
| SKIL | -0.76039 | 3.59E-06 | 0.000383 | CA2 |
| ABCA5 | -0.7846 | 3.85E-06 | 0.000408 | CA2 |
| MADCAM1 | 1.224435 | 4.02E-06 | 0.000418 | CA2 |
| ZNF423 | 0.879484 | 4.02E-06 | 0.000418 | CA2 |
| PM20D2 | -0.90644 | 3.97E-06 | 0.000418 | CA2 |
| NDUFS7 | 0.657964 | 4.06E-06 | 0.00042 | CA2 |
| ILVBL | 0.614258 | 4.35E-06 | 0.000433 | CA2 |
| KRR1 | -0.68903 | 4.29E-06 | 0.000433 | CA2 |
| ZNF431 | -0.68213 | 4.26E-06 | 0.000433 | CA2 |
| SOX15 | 1.525158 | 4.41E-06 | 0.000433 | CA2 |
| ZNF829 | -0.66949 | 4.49E-06 | 0.000439 | CA2 |
| MRPS18A | 0.625523 | 4.56E-06 | 0.000441 | CA2 |
| NDUFS6 | 0.898228 | 4.57E-06 | 0.000441 | CA2 |
| WDR75 | -0.5921 | 4.61E-06 | 0.000442 | CA2 |
| MICU2 | -0.67338 | 4.73E-06 | 0.000448 | CA2 |
| GLDC | 0.628031 | 4.85E-06 | 0.000455 | CA2 |
| RB1CC1 | -0.63745 | 5.03E-06 | 0.000464 | CA2 |
| SAP130 | 0.586032 | 5.18E-06 | 0.000475 | CA2 |
| ARVCF | 0.651907 | 5.41E-06 | 0.000487 | CA2 |
| MEGF8 | 0.706648 | 5.38E-06 | 0.000487 | CA2 |
| RAC3 | 0.968949 | 5.50E-06 | 0.000494 | CA2 |
| VPS51 | 0.679157 | 5.80E-06 | 0.000507 | CA2 |
| AP2A1 | 0.58418 | 5.80E-06 | 0.000507 | CA2 |
| INAFM1 | 0.696182 | 5.94E-06 | 0.000513 | CA2 |
| TRMT10C | -0.67509 | 6.03E-06 | 0.000518 | CA2 |
| PPP4C | 0.596507 | 6.35E-06 | 0.00054 | CA2 |
| AP1B1 | 0.592648 | 6.83E-06 | 0.000572 | CA2 |
| ARHGAP5 | -1.25747 | 6.80E-06 | 0.000572 | CA2 |
| ZNF781 | -0.88149 | 6.77E-06 | 0.000572 | CA2 |
| DTWD1 | -0.88937 | 6.92E-06 | 0.000576 | CA2 |
| CTDSPL2 | -0.66833 | 7.03E-06 | 0.000582 | CA2 |
| POLR2I | 0.804202 | 7.23E-06 | 0.000591 | CA2 |

| | | | | |
|---|---|---|---|---|
| ZNF865 | 0.773853 | 7.24E-06 | 0.000591 | CA2 |
| ODF2L | -0.79663 | 7.42E-06 | 0.000597 | CA2 |
| LCORL | -0.68112 | 7.39E-06 | 0.000597 | CA2 |
| IFI27L2 | 0.712828 | 7.98E-06 | 0.000623 | CA2 |
| PEX14 | 0.635278 | 7.85E-06 | 0.000623 | CA2 |
| CTF1 | 1.053789 | 7.89E-06 | 0.000623 | CA2 |
| TMX3 | -0.65894 | 7.95E-06 | 0.000623 | CA2 |
| CERS1 | 0.8594 | 7.91E-06 | 0.000623 | CA2 |
| GCFC2 | -0.71762 | 8.15E-06 | 0.000634 | CA2 |
| MFSD3 | 0.958921 | 8.35E-06 | 0.00064 | CA2 |
| UGGT2 | -0.69751 | 8.59E-06 | 0.000651 | CA2 |
| HECTD2 | -0.7114 | 8.62E-06 | 0.000651 | CA2 |
| NAT8L | 0.729965 | 8.70E-06 | 0.000652 | CA2 |
| ZNF235 | -0.59176 | 8.93E-06 | 0.000663 | CA2 |
| DDX54 | 0.59503 | 9.10E-06 | 0.000665 | CA2 |
| NR2F1 | 0.731595 | 9.16E-06 | 0.000665 | CA2 |
| RPF2 | -0.72664 | 9.21E-06 | 0.000665 | CA2 |
| TOMM7 | 0.742993 | 9.32E-06 | 0.000667 | CA2 |
| CEP44 | -0.62798 | 9.49E-06 | 0.00067 | CA2 |
| INTS5 | 0.777091 | 9.48E-06 | 0.00067 | CA2 |
| EEFSEC | 0.606199 | 9.86E-06 | 0.000693 | CA2 |
| ZSCAN30 | -0.68891 | 1.01E-05 | 0.000699 | CA2 |
| RPP21 | 0.82341 | 1.00E-05 | 0.000699 | CA2 |
| JAK2 | -0.63046 | 1.09E-05 | 0.000744 | CA2 |
| PC | 0.624917 | 1.10E-05 | 0.000746 | CA2 |
| NDUFB2 | 0.695107 | 1.11E-05 | 0.000749 | CA2 |
| DIAPH2 | -0.85336 | 1.12E-05 | 0.000755 | CA2 |
| LRRIQ3 | -1.81275 | 1.14E-05 | 0.00076 | CA2 |
| CLEC11A | 1.241595 | 1.15E-05 | 0.000761 | CA2 |
| NDUFA1 | 1.128189 | 1.16E-05 | 0.000768 | CA2 |
| PAMR1 | 0.870535 | 1.19E-05 | 0.000784 | CA2 |
| CTSD | 0.935303 | 1.23E-05 | 0.000801 | CA2 |
| IFT80 | -0.7866 | 1.23E-05 | 0.000802 | CA2 |
| ZNF141 | -0.80679 | 1.24E-05 | 0.000804 | CA2 |
| MSI1 | 0.611179 | 1.26E-05 | 0.000812 | CA2 |
| NDUFAF3 | 0.653174 | 1.27E-05 | 0.000812 | CA2 |
| SHARPIN | 0.609506 | 1.28E-05 | 0.000815 | CA2 |
| ARMC5 | 0.638349 | 1.30E-05 | 0.000823 | CA2 |
| ARL15 | -0.82523 | 1.33E-05 | 0.000837 | CA2 |
| MAP3K2 | -0.61963 | 1.34E-05 | 0.000841 | CA2 |
| POLR3G | -0.96209 | 1.35E-05 | 0.000842 | CA2 |
| FOXP4 | 0.62445 | 1.35E-05 | 0.000844 | CA2 |
| CRYBG3 | -0.64355 | 1.37E-05 | 0.000844 | CA2 |
| SENP6 | -0.58995 | 1.39E-05 | 0.000844 | CA2 |

| | | | | |
|---|---|---|---|---|
| THAP5 | -0.86353 | 1.40E-05 | 0.000844 | CA2 |
| MIS18BP1 | -0.87187 | 1.42E-05 | 0.000851 | CA2 |
| PAX6 | 0.640837 | 1.44E-05 | 0.000856 | CA2 |
| SART1 | 0.601368 | 1.43E-05 | 0.000856 | CA2 |
| ANGEL2 | -0.68903 | 1.48E-05 | 0.000871 | CA2 |
| RPL36AL | 0.851595 | 1.53E-05 | 0.000886 | CA2 |
| FBLN1 | 0.945563 | 1.55E-05 | 0.000887 | CA2 |
| MDK | 1.162021 | 1.54E-05 | 0.000887 | CA2 |
| FLYWCH1 | 0.610447 | 1.57E-05 | 0.000891 | CA2 |
| ABCE1 | -0.68163 | 1.59E-05 | 0.000897 | CA2 |
| SMC6 | -0.61907 | 1.60E-05 | 0.0009 | CA2 |
| UFM1 | -0.69415 | 1.64E-05 | 0.000911 | CA2 |
| MAP9 | -0.84471 | 1.64E-05 | 0.000911 | CA2 |
| U2AF2 | 0.584563 | 1.66E-05 | 0.000919 | CA2 |
| LHPP | 0.838818 | 1.68E-05 | 0.00092 | CA2 |
| F8A3 | 0.737733 | 1.68E-05 | 0.00092 | CA2 |
| MT3 | 1.067109 | 1.69E-05 | 0.000921 | CA2 |
| PTRH1 | 0.794686 | 1.72E-05 | 0.000936 | CA2 |
| GSTP1 | 0.767662 | 1.75E-05 | 0.000944 | CA2 |
| ABCD1 | 0.708341 | 1.75E-05 | 0.000944 | CA2 |
| USP45 | -0.60747 | 1.77E-05 | 0.000949 | CA2 |
| TIMM29 | 0.771913 | 1.80E-05 | 0.000964 | CA2 |
| SLC22A17 | 0.833461 | 1.82E-05 | 0.000967 | CA2 |
| SHISA4 | 0.727486 | 1.82E-05 | 0.000967 | CA2 |
| CCDC186 | -0.71873 | 1.83E-05 | 0.000971 | CA2 |
| CCDC82 | -0.79606 | 1.84E-05 | 0.000974 | CA2 |
| FANCM | -0.80565 | 1.85E-05 | 0.000976 | CA2 |
| CCDC91 | -0.58146 | 1.87E-05 | 0.000981 | CA2 |
| KATNBL1 | -0.70109 | 1.90E-05 | 0.000987 | CA2 |
| MZT2A | 0.859641 | 1.89E-05 | 0.000987 | CA2 |
| ZNF891 | -0.67828 | 1.90E-05 | 0.000987 | CA2 |
| UQCRQ | 0.750294 | 1.93E-05 | 0.000996 | CA2 |
| ECSIT | 0.641493 | 1.96E-05 | 0.001002 | CA2 |
| SUOX | 0.787992 | 1.97E-05 | 0.001002 | CA2 |
| SNAPC2 | 0.696829 | 1.98E-05 | 0.001003 | CA2 |
| PEPD | 0.711018 | 1.99E-05 | 0.001003 | CA2 |
| H4C3 | 1.04297 | 2.06E-05 | 0.001035 | CA2 |
| ATG12 | -0.66879 | 2.09E-05 | 0.001048 | CA2 |
| MIGA1 | -0.65147 | 2.16E-05 | 0.001078 | CA2 |
| ZNF574 | 0.710632 | 2.18E-05 | 0.001087 | CA2 |
| JUP | 0.704346 | 2.21E-05 | 0.001096 | CA2 |
| GRINA | 0.77108 | 2.22E-05 | 0.001096 | CA2 |
| AKT1S1 | 0.611204 | 2.25E-05 | 0.001102 | CA2 |
| LYRM7 | -0.82561 | 2.29E-05 | 0.001111 | CA2 |

| | | | | |
|---|---|---|---|---|
| NTN1 | 0.658409 | 2.32E-05 | 0.00112 | CA2 |
| G2E3 | -0.67961 | 2.31E-05 | 0.00112 | CA2 |
| FBXL7 | 0.703841 | 2.39E-05 | 0.001128 | CA2 |
| GPAA1 | 0.603385 | 2.38E-05 | 0.001128 | CA2 |
| TMEM256 | 0.847412 | 2.42E-05 | 0.001131 | CA2 |
| CLUH | 0.639072 | 2.45E-05 | 0.001136 | CA2 |
| H4C2 | 1.362729 | 2.45E-05 | 0.001136 | CA2 |
| EVC | 0.690709 | 2.47E-05 | 0.001137 | CA2 |
| RALGPS2 | -0.75974 | 2.47E-05 | 0.001137 | CA2 |
| TMEM260 | -0.65377 | 2.54E-05 | 0.001157 | CA2 |
| NDUFB9 | 0.823429 | 2.55E-05 | 0.001157 | CA2 |
| NOTCH1 | 0.76744 | 2.55E-05 | 0.001157 | CA2 |
| CASTOR2 | 0.620434 | 2.59E-05 | 0.001169 | CA2 |
| ATP1A2 | 0.808769 | 2.64E-05 | 0.001185 | CA2 |
| POMGNT2 | 0.772123 | 2.66E-05 | 0.001185 | CA2 |
| AURKAIP1 | 0.685121 | 2.66E-05 | 0.001185 | CA2 |
| FAR1 | -0.64556 | 2.66E-05 | 0.001185 | CA2 |
| NDUFB11 | 0.774589 | 2.69E-05 | 0.001193 | CA2 |
| SCAMP3 | 0.595757 | 2.71E-05 | 0.001194 | CA2 |
| TUBG1 | 0.659446 | 2.71E-05 | 0.001194 | CA2 |
| RASA2 | -0.69076 | 2.73E-05 | 0.001199 | CA2 |
| METTL26 | 0.736651 | 2.82E-05 | 0.001224 | CA2 |
| UBE2L6 | 0.722088 | 2.82E-05 | 0.001224 | CA2 |
| SNRPC | 0.624845 | 2.86E-05 | 0.001233 | CA2 |
| WDR36 | -0.61275 | 2.91E-05 | 0.001239 | CA2 |
| TUBE1 | -0.66251 | 2.95E-05 | 0.001242 | CA2 |
| SCAMP4 | 0.580985 | 2.94E-05 | 0.001242 | CA2 |
| MRPS24 | 0.845596 | 3.02E-05 | 0.001251 | CA2 |
| CARNMT1 | -0.6966 | 3.02E-05 | 0.001251 | CA2 |
| DNAJB14 | -0.67679 | 3.03E-05 | 0.001251 | CA2 |
| ESCO1 | -0.69808 | 3.04E-05 | 0.001254 | CA2 |
| TSR3 | 0.598263 | 3.06E-05 | 0.001256 | CA2 |
| RPLP2 | 0.693297 | 3.07E-05 | 0.001258 | CA2 |
| ZNF506 | -0.66285 | 3.13E-05 | 0.001268 | CA2 |
| FBXW5 | 0.728102 | 3.13E-05 | 0.001268 | CA2 |
| ZNF708 | -0.78706 | 3.13E-05 | 0.001268 | CA2 |
| ZNF326 | -0.77268 | 3.21E-05 | 0.001289 | CA2 |
| VCPKMT | -0.75297 | 3.25E-05 | 0.001296 | CA2 |
| FBXO2 | 0.767414 | 3.24E-05 | 0.001296 | CA2 |
| AZI2 | -0.59882 | 3.24E-05 | 0.001296 | CA2 |
| LGALSL | -0.62865 | 3.29E-05 | 0.001302 | CA2 |
| ARID4B | -0.61519 | 3.30E-05 | 0.001302 | CA2 |
| EIF6 | 0.644328 | 3.30E-05 | 0.001302 | CA2 |
| NUDT1 | 0.924489 | 3.35E-05 | 0.001308 | CA2 |

| | | | | |
|---|---|---|---|---|
| PPDPF | 1.032816 | 3.35E-05 | 0.001308 | CA2 |
| COLCA2 | 0.853596 | 3.35E-05 | 0.001308 | CA2 |
| AL928654.3 | 1.870971 | 3.33E-05 | 0.001308 | CA2 |
| IFI27L1 | 0.630014 | 3.43E-05 | 0.001323 | CA2 |
| MPHOSPH9 | -0.62571 | 3.45E-05 | 0.00133 | CA2 |
| ARMC6 | 0.62115 | 3.48E-05 | 0.001331 | CA2 |
| ATP6V1F | 0.759672 | 3.53E-05 | 0.001336 | CA2 |
| IRF2BP1 | 0.581878 | 3.54E-05 | 0.001336 | CA2 |
| RBM42 | 0.631664 | 3.57E-05 | 0.001336 | CA2 |
| HERC4 | -0.64242 | 3.59E-05 | 0.001336 | CA2 |
| GLMN | -0.7484 | 3.60E-05 | 0.001338 | CA2 |
| TRAF7 | 0.604101 | 3.66E-05 | 0.001353 | CA2 |
| NOP10 | 1.065665 | 3.66E-05 | 0.001353 | CA2 |
| TMSB10 | 0.947848 | 3.72E-05 | 0.001367 | CA2 |
| LLGL1 | 0.59153 | 3.75E-05 | 0.001369 | CA2 |
| UQCC3 | 0.865566 | 3.76E-05 | 0.001369 | CA2 |
| TRDMT1 | -0.71105 | 3.78E-05 | 0.001369 | CA2 |
| GAMT | 0.686697 | 3.83E-05 | 0.00138 | CA2 |
| TIA1 | -0.62186 | 3.86E-05 | 0.001388 | CA2 |
| PHGDH | 0.675535 | 3.89E-05 | 0.001391 | CA2 |
| POLRMT | 0.582644 | 3.90E-05 | 0.001393 | CA2 |
| GPR22 | -1.19227 | 3.96E-05 | 0.001409 | CA2 |
| N4BP2L2 | -0.60828 | 4.14E-05 | 0.001459 | CA2 |
| CYB5R4 | -0.5976 | 4.18E-05 | 0.001465 | CA2 |
| RPLP1 | 0.647026 | 4.20E-05 | 0.001467 | CA2 |
| MRPS2 | 0.932013 | 4.22E-05 | 0.001471 | CA2 |
| EMX2 | 0.709071 | 4.24E-05 | 0.001474 | CA2 |
| ZNF654 | -0.65073 | 4.35E-05 | 0.001507 | CA2 |
| PFDN2 | 0.801085 | 4.40E-05 | 0.00152 | CA2 |
| IPMK | -0.65881 | 4.46E-05 | 0.001541 | CA2 |
| LGALS1 | 0.821504 | 4.58E-05 | 0.001569 | CA2 |
| RPS21 | 0.755763 | 4.62E-05 | 0.00158 | CA2 |
| NELFB | 0.60401 | 4.63E-05 | 0.00158 | CA2 |
| RPL38 | 0.619464 | 4.75E-05 | 0.00162 | CA2 |
| UBE2W | -0.907 | 4.77E-05 | 0.001622 | CA2 |
| ERGIC2 | -0.66027 | 4.81E-05 | 0.001634 | CA2 |
| PMS1 | -0.61897 | 4.85E-05 | 0.001639 | CA2 |
| SIGMAR1 | 0.633686 | 4.88E-05 | 0.001646 | CA2 |
| SSC5D | 0.949691 | 4.90E-05 | 0.00165 | CA2 |
| FGFR3 | 0.777606 | 4.94E-05 | 0.001652 | CA2 |
| RTL8C | 0.703257 | 4.93E-05 | 0.001652 | CA2 |
| PNPT1 | -0.62464 | 5.00E-05 | 0.001657 | CA2 |
| CSTB | 0.68613 | 5.00E-05 | 0.001657 | CA2 |
| SNRNP25 | 0.591054 | 4.98E-05 | 0.001657 | CA2 |

| | | | | |
|---|---|---|---|---|
| NECAB1 | -0.8596 | 5.05E-05 | 0.001663 | CA2 |
| ATP5ME | 0.792988 | 5.03E-05 | 0.001663 | CA2 |
| BICRA | 0.641043 | 5.11E-05 | 0.001673 | CA2 |
| ALYREF | 0.719101 | 5.22E-05 | 0.001701 | CA2 |
| TAS2R13 | -0.93034 | 5.32E-05 | 0.001721 | CA2 |
| PRIM2 | -0.64689 | 5.34E-05 | 0.001722 | CA2 |
| C12orf29 | -0.67206 | 5.37E-05 | 0.001727 | CA2 |
| YIPF4 | -0.59386 | 5.50E-05 | 0.001759 | CA2 |
| NLRX1 | 0.661421 | 5.62E-05 | 0.001793 | CA2 |
| PGAP6 | 0.635758 | 5.64E-05 | 0.001795 | CA2 |
| CST3 | 0.851726 | 5.73E-05 | 0.001808 | CA2 |
| ADAMTS9 | -1.15525 | 5.70E-05 | 0.001808 | CA2 |
| VAMP5 | 0.919981 | 5.71E-05 | 0.001808 | CA2 |
| ARL2 | 0.633816 | 5.72E-05 | 0.001808 | CA2 |
| CRLF1 | 1.247961 | 5.75E-05 | 0.00181 | CA2 |
| UBA6 | -0.5851 | 5.83E-05 | 0.001819 | CA2 |
| ZNF317 | 0.602575 | 5.82E-05 | 0.001819 | CA2 |
| ADRA2C | 0.886441 | 5.82E-05 | 0.001819 | CA2 |
| GYPC | 0.810746 | 5.85E-05 | 0.001821 | CA2 |
| WDR83OS | 0.730193 | 5.86E-05 | 0.001821 | CA2 |
| VPS13A | -0.65246 | 5.94E-05 | 0.001839 | CA2 |
| TFPI | -0.91605 | 5.99E-05 | 0.00185 | CA2 |
| EYA2 | 0.735875 | 6.03E-05 | 0.001856 | CA2 |
| HSD17B10 | 0.674131 | 6.04E-05 | 0.001857 | CA2 |
| PRKAA1 | -0.58626 | 6.15E-05 | 0.001881 | CA2 |
| DPY19L2 | -0.78933 | 6.15E-05 | 0.001881 | CA2 |
| TMEM160 | 1.061698 | 6.27E-05 | 0.001913 | CA2 |
| CYP11A1 | 0.906497 | 6.31E-05 | 0.001921 | CA2 |
| ZDBF2 | -0.71247 | 6.42E-05 | 0.001946 | CA2 |
| CBR4 | -0.66104 | 6.60E-05 | 0.001977 | CA2 |
| YIPF6 | -1.36981 | 6.60E-05 | 0.001977 | CA2 |
| ZWILCH | -0.89835 | 6.68E-05 | 0.001995 | CA2 |
| ICE2 | -0.66297 | 6.77E-05 | 0.002016 | CA2 |
| ZNF483 | -0.60793 | 6.84E-05 | 0.002024 | CA2 |
| MAGOHB | -0.5952 | 6.95E-05 | 0.002052 | CA2 |
| LRP5 | 0.790585 | 6.99E-05 | 0.002058 | CA2 |
| TRNT1 | -0.62846 | 7.20E-05 | 0.002101 | CA2 |
| EPHB3 | 0.708474 | 7.21E-05 | 0.002101 | CA2 |
| ISG15 | 1.037108 | 7.21E-05 | 0.002101 | CA2 |
| FBXL14 | 0.707968 | 7.31E-05 | 0.002122 | CA2 |
| WBP1 | 0.682805 | 7.41E-05 | 0.002144 | CA2 |
| JOSD2 | 0.672476 | 7.54E-05 | 0.002167 | CA2 |
| ZUP1 | -0.58954 | 7.60E-05 | 0.002173 | CA2 |
| ERI2 | -0.72007 | 7.59E-05 | 0.002173 | CA2 |

| | | | | |
|---|---|---|---|---|
| RTN4R | 1.237623 | 7.67E-05 | 0.002188 | CA2 |
| PPP1R1B | 0.759386 | 8.13E-05 | 0.002292 | CA2 |
| RTL8A | 0.718077 | 8.15E-05 | 0.002295 | CA2 |
| COX7A1 | 1.214026 | 8.23E-05 | 0.002304 | CA2 |
| ANAPC11 | 0.591559 | 8.31E-05 | 0.002318 | CA2 |
| RNF138 | -0.70343 | 8.35E-05 | 0.002325 | CA2 |
| RBM48 | -0.79457 | 8.56E-05 | 0.002367 | CA2 |
| TSPAN4 | 0.720851 | 8.63E-05 | 0.002376 | CA2 |
| LRRC24 | 0.702802 | 8.66E-05 | 0.002376 | CA2 |
| H4C11 | 1.312161 | 8.72E-05 | 0.00238 | CA2 |
| FXYD1 | 0.873766 | 8.73E-05 | 0.00238 | CA2 |
| ZRANB2 | -0.61418 | 9.01E-05 | 0.002415 | CA2 |
| ZGRF1 | -0.81894 | 9.03E-05 | 0.002415 | CA2 |
| LPAR6 | -1.08287 | 8.92E-05 | 0.002415 | CA2 |
| PRXL2C | -0.72988 | 8.89E-05 | 0.002415 | CA2 |
| SLC16A11 | 1.063388 | 8.99E-05 | 0.002415 | CA2 |
| RPL12 | 0.637944 | 8.96E-05 | 0.002415 | CA2 |
| ENPP4 | -0.7386 | 9.43E-05 | 0.002486 | CA2 |
| CHST7 | 0.816695 | 9.42E-05 | 0.002486 | CA2 |
| FAM161A | -0.61301 | 9.40E-05 | 0.002486 | CA2 |
| MIF | 0.871651 | 9.38E-05 | 0.002486 | CA2 |
| AASDHPPT | -0.59677 | 9.54E-05 | 0.002496 | CA2 |
| ATF7IP2 | -0.65188 | 9.50E-05 | 0.002496 | CA2 |
| PRDM16 | 0.759917 | 9.57E-05 | 0.0025 | CA2 |
| AIFM3 | 0.67463 | 9.59E-05 | 0.002502 | CA2 |
| LACC1 | -0.91527 | 9.69E-05 | 0.002511 | CA2 |
| TCF7 | 0.965172 | 9.78E-05 | 0.002521 | CA2 |
| THRA | 0.58435 | 9.85E-05 | 0.002531 | CA2 |
| PLEKHA1 | -0.62202 | 9.98E-05 | 0.002549 | CA2 |
| RMDN2 | -0.65543 | 0.0001 | 0.002554 | CA2 |
| THOC1 | -0.64409 | 0.000102 | 0.002572 | CA2 |
| SLC25A1 | 0.629079 | 0.000102 | 0.002572 | CA2 |
| RFX1 | 0.587522 | 0.000101 | 0.002572 | CA2 |
| ZNF138 | -0.79047 | 0.000102 | 0.002572 | CA2 |
| C5orf49 | 0.803402 | 0.000102 | 0.002572 | CA2 |
| SCARF2 | 0.760687 | 0.000102 | 0.002572 | CA2 |
| MFSD10 | 0.660316 | 0.000105 | 0.002612 | CA2 |
| GPT | 0.802998 | 0.000105 | 0.002612 | CA2 |
| ZNF519 | -0.8177 | 0.000106 | 0.00262 | CA2 |
| LSM7 | 0.8557 | 0.000107 | 0.002633 | CA2 |
| RAD54B | -0.751 | 0.000107 | 0.002647 | CA2 |
| TBC1D25 | 0.64705 | 0.000108 | 0.002657 | CA2 |
| MTCH1 | 0.582134 | 0.000108 | 0.002659 | CA2 |
| PCDH15 | -0.81762 | 0.000109 | 0.002668 | CA2 |

| | | | | |
|---|---|---|---|---|
| MLST8 | 0.615012 | 0.00011 | 0.002699 | CA2 |
| RNF187 | 0.690665 | 0.000111 | 0.002712 | CA2 |
| ZNF503 | 0.780812 | 0.000112 | 0.002728 | CA2 |
| ZNF575 | 0.866691 | 0.000112 | 0.002728 | CA2 |
| PPP1R13L | 0.750751 | 0.000113 | 0.002737 | CA2 |
| LGALS8 | -0.64605 | 0.000115 | 0.002759 | CA2 |
| AVPI1 | 0.66253 | 0.000115 | 0.002759 | CA2 |
| EMP3 | 0.879701 | 0.000115 | 0.002759 | CA2 |
| CCDC102B | -0.658 | 0.000114 | 0.002759 | CA2 |
| TUBA1C | 0.735109 | 0.000116 | 0.002759 | CA2 |
| BCLAF3 | -0.5814 | 0.000115 | 0.002759 | CA2 |
| GDAP1 | -0.60407 | 0.000117 | 0.002782 | CA2 |
| UBE3D | -0.60398 | 0.000117 | 0.002795 | CA2 |
| SLC26A2 | -0.87274 | 0.000118 | 0.002796 | CA2 |
| LDOC1 | 0.830656 | 0.000119 | 0.002818 | CA2 |
| CHRNA4 | 0.839063 | 0.00012 | 0.00283 | CA2 |
| MZT1 | -0.91469 | 0.00012 | 0.002833 | CA2 |
| SRM | 0.682308 | 0.000121 | 0.002836 | CA2 |
| IRF2BPL | 0.58777 | 0.000121 | 0.002836 | CA2 |
| KCNT2 | -0.73994 | 0.000121 | 0.002836 | CA2 |
| PIGT | 0.671583 | 0.000121 | 0.002838 | CA2 |
| VAMP4 | -0.70007 | 0.000122 | 0.002854 | CA2 |
| EFCAB7 | -0.63976 | 0.000123 | 0.002854 | CA2 |
| EPHB6 | 0.71714 | 0.000123 | 0.002857 | CA2 |
| TUBB | 0.645311 | 0.000124 | 0.002878 | CA2 |
| MFRP | 3.266599 | 0.000124 | 0.002878 | CA2 |
| REX1BD | 0.592385 | 0.000126 | 0.002915 | CA2 |
| CENPC | -0.61202 | 0.000129 | 0.002942 | CA2 |
| ZNF23 | -0.74981 | 0.000129 | 0.002942 | CA2 |
| RRAS | 0.783138 | 0.00013 | 0.002967 | CA2 |
| ZSWIM4 | 0.583468 | 0.00013 | 0.002967 | CA2 |
| FXYD5 | 0.854251 | 0.000135 | 0.003041 | CA2 |
| GTPBP10 | -0.72251 | 0.000135 | 0.003041 | CA2 |
| DOHH | 0.663952 | 0.000135 | 0.003041 | CA2 |
| PDLIM4 | 0.825548 | 0.000135 | 0.003041 | CA2 |
| NAPG | -0.6183 | 0.000136 | 0.003048 | CA2 |
| SLC39A13 | 0.613987 | 0.000137 | 0.003055 | CA2 |
| ZNF638 | -0.60315 | 0.000139 | 0.003088 | CA2 |
| ATP13A2 | 0.675862 | 0.000139 | 0.003093 | CA2 |
| CASP3 | -0.59648 | 0.000141 | 0.003113 | CA2 |
| PLD3 | 0.804494 | 0.000143 | 0.003158 | CA2 |
| SLC12A4 | 0.584214 | 0.000144 | 0.003161 | CA2 |
| AP5M1 | -0.60223 | 0.000145 | 0.003167 | CA2 |
| NAGLU | 0.618315 | 0.000145 | 0.003167 | CA2 |

| | | | | |
|---|---|---|---|---|
| CYB5R3 | 0.657467 | 0.000146 | 0.003181 | CA2 |
| DOCK11 | -0.59027 | 0.000148 | 0.003202 | CA2 |
| MOB1B | -0.65769 | 0.000148 | 0.003202 | CA2 |
| ZNF184 | -1.01472 | 0.000152 | 0.003265 | CA2 |
| SORBS3 | 0.596497 | 0.000152 | 0.003268 | CA2 |
| RENBP | 0.580279 | 0.000153 | 0.003276 | CA2 |
| BLOC1S3 | 0.934611 | 0.000154 | 0.003276 | CA2 |
| NIPA1 | -1.44706 | 0.000154 | 0.003279 | CA2 |
| FOXN2 | -0.72401 | 0.000155 | 0.003282 | CA2 |
| ATG4C | -0.79309 | 0.000156 | 0.003298 | CA2 |
| UBA52 | 0.700469 | 0.000159 | 0.003343 | CA2 |
| BORA | -0.89083 | 0.00016 | 0.003347 | CA2 |
| ITGB4 | 0.848933 | 0.000161 | 0.003361 | CA2 |
| RRAD | 1.353128 | 0.000162 | 0.003371 | CA2 |
| UBB | 0.687329 | 0.000162 | 0.003379 | CA2 |
| LDB1 | 0.612253 | 0.000163 | 0.003391 | CA2 |
| SENP7 | -0.59148 | 0.000171 | 0.003488 | CA2 |
| WFIKKN2 | 2.038559 | 0.00017 | 0.003488 | CA2 |
| PPP4R2 | -0.69021 | 0.000172 | 0.0035 | CA2 |
| TLE5 | 0.665176 | 0.000176 | 0.003554 | CA2 |
| GCHFR | 1.136064 | 0.000175 | 0.003554 | CA2 |
| C1orf35 | 0.718208 | 0.000177 | 0.003575 | CA2 |
| TUBB4B | 0.673034 | 0.000177 | 0.003575 | CA2 |
| CYSTM1 | 0.588714 | 0.000178 | 0.003581 | CA2 |
| VPS26A | -0.66805 | 0.000178 | 0.003581 | CA2 |
| IRAK1BP1 | -0.71894 | 0.000178 | 0.003581 | CA2 |
| POLR2J | 0.618171 | 0.000179 | 0.003586 | CA2 |
| NR1D1 | 0.820054 | 0.00018 | 0.003608 | CA2 |
| GK5 | -0.59541 | 0.00018 | 0.003608 | CA2 |
| EPN1 | 0.597209 | 0.000181 | 0.003608 | CA2 |
| S100A1 | 0.737883 | 0.000181 | 0.003608 | CA2 |
| TRPV4 | 1.709365 | 0.000185 | 0.003674 | CA2 |
| FAM181B | 0.648487 | 0.000185 | 0.003676 | CA2 |
| H4C5 | 0.981904 | 0.000187 | 0.003685 | CA2 |
| TMEM132A | 0.73512 | 0.000188 | 0.003692 | CA2 |
| PRPF39 | -0.61028 | 0.000188 | 0.003692 | CA2 |
| NDUFA6 | 0.602582 | 0.000191 | 0.003721 | CA2 |
| RBP1 | 0.719579 | 0.000192 | 0.003729 | CA2 |
| UBL5 | 0.889812 | 0.000193 | 0.003735 | CA2 |
| ACTR3C | -0.61751 | 0.000195 | 0.003756 | CA2 |
| MUSTN1 | 1.859776 | 0.000195 | 0.003768 | CA2 |
| ADIRF | 0.870062 | 0.000196 | 0.003777 | CA2 |
| CPTP | 0.629516 | 0.000197 | 0.003792 | CA2 |
| NDUFS5 | 0.843019 | 0.000198 | 0.003797 | CA2 |

| | | | | |
|---|---|---|---|---|
| TVP23B | -0.75582 | 0.000198 | 0.003797 | CA2 |
| GPT2 | 0.639078 | 0.000199 | 0.003813 | CA2 |
| RBM41 | -0.62108 | 0.000203 | 0.00386 | CA2 |
| RPL8 | 0.603203 | 0.000203 | 0.00386 | CA2 |
| CHST9 | -0.90105 | 0.000205 | 0.003888 | CA2 |
| AP3S1 | -0.6192 | 0.000205 | 0.003888 | CA2 |
| CASP8AP2 | -0.66441 | 0.000206 | 0.003891 | CA2 |
| RNF26 | 0.582587 | 0.00021 | 0.003963 | CA2 |
| ANKRD49 | -0.64859 | 0.000212 | 0.003976 | CA2 |
| SECISBP2L | -0.63349 | 0.000214 | 0.004008 | CA2 |
| RPL29 | 0.787572 | 0.000218 | 0.004056 | CA2 |
| HLA-DRB1 | 1.691531 | 0.000223 | 0.004116 | CA2 |
| PLTP | 0.76113 | 0.000225 | 0.004132 | CA2 |
| ANKRD36 | -0.67396 | 0.000228 | 0.004172 | CA2 |
| ZNF181 | -0.59024 | 0.000229 | 0.004172 | CA2 |
| PTCH2 | 0.707165 | 0.000231 | 0.00419 | CA2 |
| C7orf25 | -0.75864 | 0.000231 | 0.00419 | CA2 |
| NTN3 | 1.037902 | 0.000231 | 0.00419 | CA2 |
| AC024592.3 | 1.11429 | 0.000232 | 0.004205 | CA2 |
| DNAH8 | -1.19884 | 0.000233 | 0.004208 | CA2 |
| ZDHHC20 | -0.72713 | 0.000233 | 0.004212 | CA2 |
| ZFP2 | -0.95152 | 0.000234 | 0.004212 | CA2 |
| LSM2 | 0.603036 | 0.000234 | 0.00422 | CA2 |
| ERO1A | -0.63382 | 0.000235 | 0.004225 | CA2 |
| AC098850.3 | -2.07141 | 0.000236 | 0.004236 | CA2 |
| RTN4RL2 | 1.146128 | 0.000238 | 0.004271 | CA2 |
| ARHGAP18 | -0.65991 | 0.000245 | 0.004348 | CA2 |
| ABHD14A | 0.776158 | 0.000247 | 0.004377 | CA2 |
| RGS17 | -0.89755 | 0.000248 | 0.004383 | CA2 |
| HELLS | -0.63544 | 0.00025 | 0.004408 | CA2 |
| TMEM109 | 0.63681 | 0.000252 | 0.004418 | CA2 |
| COMT | 0.665703 | 0.000254 | 0.004445 | CA2 |
| AC073896.1 | 0.863104 | 0.000259 | 0.004518 | CA2 |
| ZNF761 | -0.6903 | 0.000268 | 0.004659 | CA2 |
| CACNG7 | 0.64879 | 0.000269 | 0.004661 | CA2 |
| SLC13A4 | 2.711421 | 0.000276 | 0.004766 | CA2 |
| MPG | 0.600509 | 0.000279 | 0.00479 | CA2 |
| ATAD5 | -0.7805 | 0.000279 | 0.00479 | CA2 |
| HCN2 | 0.846601 | 0.000279 | 0.004792 | CA2 |
| OPRL1 | 0.693832 | 0.000282 | 0.004812 | CA2 |
| RNF208 | 0.895623 | 0.000281 | 0.004812 | CA2 |
| SNX16 | -0.62891 | 0.000293 | 0.00492 | CA2 |
| TCF7L1 | 0.641364 | 0.000294 | 0.004923 | CA2 |
| THAP8 | 0.629411 | 0.000294 | 0.004923 | CA2 |

| | | | | |
|---|---|---|---|---|
| NDUFA7 | 0.87052 | 0.000294 | 0.004923 | CA2 |
| RPL11 | 0.615307 | 0.000297 | 0.004955 | CA2 |
| SERTAD1 | 0.869925 | 0.000299 | 0.00498 | CA2 |
| LRRC8B | -0.67282 | 0.000305 | 0.005073 | CA2 |
| ADAMTS2 | 0.987817 | 0.000307 | 0.00509 | CA2 |
| SAMD3 | -1.3276 | 0.000308 | 0.00509 | CA2 |
| GNG5 | 0.733691 | 0.000307 | 0.00509 | CA2 |
| N4BP2 | -0.5973 | 0.000308 | 0.005093 | CA2 |
| HSPB6 | 0.991689 | 0.000309 | 0.005108 | CA2 |
| FGFRL1 | 0.824335 | 0.000311 | 0.00512 | CA2 |
| POP7 | 0.753216 | 0.000313 | 0.00514 | CA2 |
| TFPT | 0.606581 | 0.000313 | 0.005141 | CA2 |
| PCDHGB2 | 0.774743 | 0.000314 | 0.005149 | CA2 |
| COL1A1 | 1.256449 | 0.000316 | 0.005172 | CA2 |
| IRX1 | 1.223893 | 0.000317 | 0.005174 | CA2 |
| DNLZ | 0.860141 | 0.000317 | 0.005178 | CA2 |
| BPNT2 | -0.58317 | 0.000321 | 0.005214 | CA2 |
| MTERF1 | -0.75687 | 0.00032 | 0.005214 | CA2 |
| PLAG1 | -0.68845 | 0.000324 | 0.005255 | CA2 |
| PNMT | 0.94385 | 0.000326 | 0.005274 | CA2 |
| CDC42SE2 | -0.81323 | 0.00033 | 0.005321 | CA2 |
| YIF1A | 0.642413 | 0.000332 | 0.005342 | CA2 |
| HCST | 1.587212 | 0.000337 | 0.005407 | CA2 |
| LAMB2 | 0.669881 | 0.000338 | 0.005419 | CA2 |
| CFL1 | 0.735708 | 0.000339 | 0.005431 | CA2 |
| TOMM6 | -1.87031 | 0.000339 | 0.005431 | CA2 |
| TMEM54 | 0.924663 | 0.000343 | 0.005469 | CA2 |
| C6orf226 | 0.980181 | 0.000344 | 0.005469 | CA2 |
| COPS9 | 0.679341 | 0.000347 | 0.005503 | CA2 |
| PCDHGC4 | 1.196898 | 0.000349 | 0.005515 | CA2 |
| SLC6A15 | -0.61174 | 0.000353 | 0.005558 | CA2 |
| CEND1 | 0.835955 | 0.000353 | 0.005558 | CA2 |
| CUZD1 | -0.78637 | 0.000362 | 0.005646 | CA2 |
| EFCAB10 | -1.21196 | 0.000362 | 0.005648 | CA2 |
| ZNF579 | 0.641112 | 0.000365 | 0.005662 | CA2 |
| PAX2 | 1.805117 | 0.000367 | 0.005687 | CA2 |
| GAS2L1 | 0.744373 | 0.000367 | 0.005689 | CA2 |
| ATP5MF-PTCD1 | 1.300603 | 0.000368 | 0.005689 | CA2 |
| CLEC2B | -1.05077 | 0.00037 | 0.005712 | CA2 |
| TICAM1 | 0.596543 | 0.000372 | 0.005715 | CA2 |
| CBS | 0.725697 | 0.000371 | 0.005715 | CA2 |
| TAS2R14 | -0.88355 | 0.000372 | 0.005715 | CA2 |
| TMEM151A | 0.743156 | 0.000375 | 0.005755 | CA2 |
| RSC1A1 | -2.73072 | 0.000377 | 0.005763 | CA2 |

| | | | | |
|---|---|---|---|---|
| TMEM161B | -0.60568 | 0.00038 | 0.005792 | CA2 |
| PTGS2 | -1.26469 | 0.000385 | 0.005832 | CA2 |
| ZNF189 | -0.58321 | 0.000386 | 0.005841 | CA2 |
| COPZ2 | 0.651223 | 0.000395 | 0.005936 | CA2 |
| WFS1 | 0.74952 | 0.000395 | 0.005936 | CA2 |
| BORCS8-MEF2B | 0.895969 | 0.000398 | 0.005952 | CA2 |
| EGR4 | 1.511026 | 0.000398 | 0.005952 | CA2 |
| BANF1 | 0.73766 | 0.000399 | 0.005962 | CA2 |
| TEX15 | -0.74181 | 0.000404 | 0.006002 | CA2 |
| SLC43A2 | 0.66832 | 0.000406 | 0.006012 | CA2 |
| NOC4L | 0.655668 | 0.000406 | 0.006012 | CA2 |
| EEF1G | 0.842964 | 0.000412 | 0.006058 | CA2 |
| DDTL | 0.704646 | 0.000419 | 0.006124 | CA2 |
| FAM171A2 | 0.869011 | 0.000418 | 0.006124 | CA2 |
| SPCS3 | -0.67714 | 0.000421 | 0.006126 | CA2 |
| DGKI | -0.70553 | 0.00042 | 0.006126 | CA2 |
| SNCG | 0.866548 | 0.000419 | 0.006126 | CA2 |
| CEP83 | -0.69171 | 0.000421 | 0.006126 | CA2 |
| FAM241A | -0.85761 | 0.00042 | 0.006126 | CA2 |
| ERBB2 | 0.646802 | 0.00043 | 0.006226 | CA2 |
| CSRNP3 | -0.83346 | 0.000431 | 0.006226 | CA2 |
| FAM174C | 0.716802 | 0.000442 | 0.006347 | CA2 |
| AGT | 0.795332 | 0.000451 | 0.006451 | CA2 |
| HINT3 | -0.63476 | 0.000452 | 0.006461 | CA2 |
| KIF20B | -0.67135 | 0.000454 | 0.006461 | CA2 |
| VKORC1 | 0.650277 | 0.000464 | 0.006558 | CA2 |
| GAL3ST3 | 0.86173 | 0.000464 | 0.006558 | CA2 |
| PBXIP1 | 0.591478 | 0.000468 | 0.00659 | CA2 |
| FSD1L | -0.60965 | 0.00047 | 0.006611 | CA2 |
| HELB | -0.6208 | 0.000474 | 0.006618 | CA2 |
| KCNMB2 | -0.72805 | 0.000474 | 0.006618 | CA2 |
| TAS2R19 | -0.79437 | 0.000474 | 0.006618 | CA2 |
| CORO7 | 0.662369 | 0.000474 | 0.006618 | CA2 |
| CHCHD2 | 0.593534 | 0.000475 | 0.006625 | CA2 |
| MAPK11 | 0.607621 | 0.000476 | 0.006626 | CA2 |
| ABCA4 | 1.826524 | 0.000491 | 0.006768 | CA2 |
| CHD1 | -0.61597 | 0.000492 | 0.006776 | CA2 |
| FKBP2 | 0.771626 | 0.000502 | 0.006843 | CA2 |
| FAM107B | -0.76559 | 0.000504 | 0.006864 | CA2 |
| CHRNA2 | 2.504532 | 0.000511 | 0.006937 | CA2 |
| TCF20 | -1.10703 | 0.000513 | 0.006957 | CA2 |
| CA4 | 0.988526 | 0.00052 | 0.007015 | CA2 |
| ST8SIA4 | -0.79566 | 0.000525 | 0.007059 | CA2 |
| PHPT1 | 0.581131 | 0.000531 | 0.00714 | CA2 |

| | | | | |
|---|---|---|---|---|
| HMGN1 | -0.59502 | 0.000538 | 0.007211 | CA2 |
| CDIPT | 0.631911 | 0.000543 | 0.007266 | CA2 |
| ABHD17A | 0.611597 | 0.000545 | 0.007286 | CA2 |
| TMEM205 | 0.597869 | 0.000546 | 0.007293 | CA2 |
| FBXW9 | 0.688007 | 0.00055 | 0.007347 | CA2 |
| SCRT2 | 2.496394 | 0.000558 | 0.007418 | CA2 |
| LRFN3 | 0.699396 | 0.000559 | 0.007421 | CA2 |
| IKZF2 | -0.60988 | 0.000563 | 0.007463 | CA2 |
| PPFIA2 | -0.86568 | 0.000564 | 0.007466 | CA2 |
| FKRP | 1.151936 | 0.000569 | 0.007519 | CA2 |
| SYDE2 | -0.65419 | 0.000572 | 0.00753 | CA2 |
| TNFSF13B | -0.8021 | 0.000575 | 0.007562 | CA2 |
| MCTS2P | -1.73941 | 0.000578 | 0.007595 | CA2 |
| LRRC75A | 0.638997 | 0.000581 | 0.007621 | CA2 |
| ELL2 | -0.63673 | 0.000585 | 0.007662 | CA2 |
| SLC15A3 | 0.583962 | 0.000593 | 0.007707 | CA2 |
| IFITM2 | 0.946508 | 0.000591 | 0.007707 | CA2 |
| AL662899.2 | 2.69983 | 0.000597 | 0.007755 | CA2 |
| FSCN1 | 0.598467 | 0.000599 | 0.00778 | CA2 |
| GZMM | 1.415664 | 0.000606 | 0.007843 | CA2 |
| KLHL23 | -0.76298 | 0.000607 | 0.007855 | CA2 |
| MLKL | -0.73589 | 0.000609 | 0.007868 | CA2 |
| NUDT8 | 0.933651 | 0.000615 | 0.007918 | CA2 |
| ZNF213 | 0.61078 | 0.000616 | 0.007924 | CA2 |
| NFYB | -0.66344 | 0.000622 | 0.00795 | CA2 |
| GDPD2 | 0.826789 | 0.000623 | 0.00795 | CA2 |
| TRPM4 | 0.649685 | 0.000622 | 0.00795 | CA2 |
| PRDX2 | 0.630228 | 0.000623 | 0.00795 | CA2 |
| THAP11 | 0.608741 | 0.000622 | 0.00795 | CA2 |
| BGLAP | 1.08168 | 0.00062 | 0.00795 | CA2 |
| RMI1 | -0.60746 | 0.000634 | 0.008059 | CA2 |
| SLC36A4 | -0.59264 | 0.000636 | 0.008067 | CA2 |
| SAXO2 | -0.60727 | 0.000644 | 0.008144 | CA2 |
| ZNF624 | -0.58893 | 0.000653 | 0.00825 | CA2 |
| PAPLN | 0.800773 | 0.000656 | 0.008255 | CA2 |
| RAB33B | -0.58473 | 0.000655 | 0.008255 | CA2 |
| CCDC167 | 0.819476 | 0.000655 | 0.008255 | CA2 |
| TRIL | 0.742075 | 0.000655 | 0.008255 | CA2 |
| LGI3 | 0.703905 | 0.000663 | 0.008305 | CA2 |
| ZFP37 | -0.66601 | 0.000672 | 0.008381 | CA2 |
| FSIP2 | -1.15694 | 0.000672 | 0.008381 | CA2 |
| ASGR1 | 0.737857 | 0.00068 | 0.008433 | CA2 |
| PGAP1 | -0.61956 | 0.000678 | 0.008433 | CA2 |
| AC069368.1 | 1.398313 | 0.000687 | 0.00849 | CA2 |

| | | | | |
|---|---|---|---|---|
| SMIM10L1 | -0.60527 | 0.000689 | 0.008505 | CA2 |
| ETHE1 | 0.647806 | 0.0007 | 0.008551 | CA2 |
| CCNC | -0.61722 | 0.000698 | 0.008551 | CA2 |
| TTLL7 | -0.62755 | 0.0007 | 0.008551 | CA2 |
| NME5 | -0.75626 | 0.000702 | 0.008562 | CA2 |
| LUZP2 | -0.7891 | 0.000715 | 0.00866 | CA2 |
| RFK | -0.89198 | 0.000723 | 0.008747 | CA2 |
| MAP2K3 | 0.586604 | 0.000724 | 0.008752 | CA2 |
| APLP1 | 0.587633 | 0.000735 | 0.008809 | CA2 |
| GIN1 | -0.65414 | 0.000735 | 0.008809 | CA2 |
| MELTF | 0.719076 | 0.000735 | 0.008809 | CA2 |
| DPM3 | 0.644282 | 0.000733 | 0.008809 | CA2 |
| CTAGE6 | -1.43239 | 0.000736 | 0.008809 | CA2 |
| TDO2 | -1.52962 | 0.000737 | 0.008811 | CA2 |
| AC093155.3 | -1.47052 | 0.000738 | 0.008811 | CA2 |
| POLR3K | 0.590858 | 0.000747 | 0.008876 | CA2 |
| LUC7L3 | -0.60786 | 0.000765 | 0.009055 | CA2 |
| ENDOG | 0.658785 | 0.000765 | 0.009055 | CA2 |
| H2BC21 | 0.598179 | 0.000766 | 0.009055 | CA2 |
| ATP6V0C | 0.66849 | 0.000775 | 0.009122 | CA2 |
| IRX3 | 1.255766 | 0.000782 | 0.00918 | CA2 |
| KIAA0408 | -0.63931 | 0.000787 | 0.009227 | CA2 |
| WTIP | 0.774297 | 0.00079 | 0.009245 | CA2 |
| ARL5B | -0.66805 | 0.000796 | 0.009266 | CA2 |
| MRPS12 | 0.688389 | 0.000805 | 0.009329 | CA2 |
| SLC5A5 | 2.849669 | 0.000815 | 0.009424 | CA2 |
| ANKRD26 | -0.71681 | 0.000837 | 0.009583 | CA2 |
| RPL13A | 0.643663 | 0.000842 | 0.009628 | CA2 |
| SEPTIN9 | 0.677642 | 0.000842 | 0.009628 | CA2 |
| SS18L2 | -0.59287 | 0.000845 | 0.009637 | CA2 |
| PHOSPHO2 | -0.67797 | 0.000847 | 0.009651 | CA2 |
| USP12 | -0.62643 | 0.00085 | 0.009667 | CA2 |
| ZDHHC21 | -0.59008 | 0.000849 | 0.009667 | CA2 |
| METRNL | 0.666917 | 0.00086 | 0.009757 | CA2 |
| ACTG1 | 0.59317 | 0.000862 | 0.00976 | CA2 |
| SMIM27 | 0.96326 | 0.000861 | 0.00976 | CA2 |
| ATP6V0E1 | 0.626848 | 0.00087 | 0.009812 | CA2 |
| TPRKB | -0.63351 | 0.000879 | 0.009861 | CA2 |
| SLC38A3 | 0.725871 | 0.000878 | 0.009861 | CA2 |
| COMMD8 | -0.72486 | 0.000887 | 0.009909 | CA2 |
| CEP162 | -0.64172 | 0.0009 | 0.009998 | CA2 |
| SLC35A3 | -0.78489 | 0.000902 | 0.010016 | CA2 |
| NECTIN2 | 0.596986 | 0.000904 | 0.010026 | CA2 |
| FBLL1 | 1.008016 | 0.000909 | 0.010058 | CA2 |

| | | | | |
|---|---|---|---|---|
| MRPL12 | 0.605671 | 0.000909 | 0.010058 | CA2 |
| EPOR | 0.653694 | 0.000913 | 0.010066 | CA2 |
| TRIQK | -0.58314 | 0.000912 | 0.010066 | CA2 |
| CCDC7 | -0.70488 | 0.000913 | 0.010066 | CA2 |
| SGO2 | -0.68208 | 0.000915 | 0.010073 | CA2 |
| DEDD2 | 0.605583 | 0.000917 | 0.010094 | CA2 |
| C4B | 1.137962 | 0.000925 | 0.010137 | CA2 |
| MRPL53 | 0.651083 | 0.000931 | 0.010195 | CA2 |
| PLGLB2 | -0.76877 | 0.000942 | 0.010269 | CA2 |
| LPIN3 | 0.746657 | 0.000942 | 0.010269 | CA2 |
| AP2S1 | 0.75315 | 0.000946 | 0.010294 | CA2 |
| FAM110A | 0.795754 | 0.000948 | 0.010301 | CA2 |
| TMEM121 | 1.120181 | 0.000948 | 0.010301 | CA2 |
| PLP2 | 0.803056 | 0.000958 | 0.010341 | CA2 |
| E2F3 | -0.64629 | 0.000955 | 0.010341 | CA2 |
| SREK1IP1 | -0.58605 | 0.000957 | 0.010341 | CA2 |
| MAP3K15 | 2.639162 | 0.000958 | 0.010341 | CA2 |
| NPB | 0.978342 | 0.000954 | 0.010341 | CA2 |
| CITED4 | 0.798875 | 0.000964 | 0.010391 | CA2 |
| IFI6 | 0.86436 | 0.00097 | 0.010443 | CA2 |
| LDHD | 0.592977 | 0.000971 | 0.010449 | CA2 |
| ANP32E | -0.59064 | 0.000984 | 0.010532 | CA2 |
| SLC35B3 | -0.59905 | 0.001006 | 0.010719 | CA2 |
| TGFB1 | 0.635359 | 0.00101 | 0.010757 | CA2 |
| CDC42EP1 | 0.650421 | 0.001018 | 0.010814 | CA2 |
| MFAP4 | 1.150088 | 0.001024 | 0.010816 | CA2 |
| RAB9B | -0.5844 | 0.00103 | 0.01087 | CA2 |
| SAGE1 | 2.507336 | 0.001033 | 0.01087 | CA2 |
| CCR10 | 0.864934 | 0.001041 | 0.010946 | CA2 |
| FLNC | 0.85047 | 0.001045 | 0.010966 | CA2 |
| BDP1 | -0.59278 | 0.001055 | 0.011034 | CA2 |
| CXADR | -0.6334 | 0.001058 | 0.01106 | CA2 |
| SNRNP48 | -0.62259 | 0.001066 | 0.011125 | CA2 |
| MKI67 | -2.03965 | 0.001069 | 0.011126 | CA2 |
| NAA80 | 0.601336 | 0.001069 | 0.011126 | CA2 |
| SYS1-DBNDD2 | 1.552055 | 0.001071 | 0.011134 | CA2 |
| BOP1 | 0.581837 | 0.001072 | 0.011134 | CA2 |
| IL1RAP | -0.61214 | 0.001078 | 0.011172 | CA2 |
| SASS6 | -0.58683 | 0.001081 | 0.011194 | CA2 |
| HLA-DQB1 | 2.090452 | 0.001082 | 0.011197 | CA2 |
| MGAM | 0.737731 | 0.001096 | 0.011331 | CA2 |
| ZNF300 | -0.65096 | 0.001112 | 0.011447 | CA2 |
| PYGM | 0.715822 | 0.001121 | 0.011509 | CA2 |
| SCAI | -0.59052 | 0.001124 | 0.011526 | CA2 |

| | | | | |
|---|---|---|---|---|
| AJM1 | 0.776206 | 0.001124 | 0.011526 | CA2 |
| D2HGDH | 0.582993 | 0.001127 | 0.011543 | CA2 |
| CBWD3 | -0.70554 | 0.001136 | 0.011578 | CA2 |
| AC002996.1 | 0.705486 | 0.001142 | 0.011624 | CA2 |
| RGPD6 | -0.63343 | 0.001161 | 0.011726 | CA2 |
| ZNF566 | -0.66345 | 0.00116 | 0.011726 | CA2 |
| AC011005.1 | 0.898304 | 0.001161 | 0.011726 | CA2 |
| TMEM168 | -0.66408 | 0.001164 | 0.011747 | CA2 |
| PLCD3 | 0.701079 | 0.001177 | 0.011834 | CA2 |
| LAGE3 | 0.725822 | 0.001191 | 0.011934 | CA2 |
| SNTA1 | 0.598657 | 0.001201 | 0.011997 | CA2 |
| FNDC10 | 0.889724 | 0.001208 | 0.012053 | CA2 |
| EIF1AX | -0.63271 | 0.001217 | 0.012103 | CA2 |
| ZNF709 | -0.88718 | 0.001225 | 0.012147 | CA2 |
| LRRC69 | -0.80278 | 0.001227 | 0.012162 | CA2 |
| MT2A | 0.959242 | 0.001234 | 0.012196 | CA2 |
| CD151 | 0.584445 | 0.001246 | 0.012289 | CA2 |
| TMEM221 | 0.847862 | 0.001253 | 0.012344 | CA2 |
| ATP6V0B | 0.588557 | 0.001256 | 0.01236 | CA2 |
| SCAMP1 | -0.60371 | 0.001279 | 0.012485 | CA2 |
| H4C12 | 0.989103 | 0.001277 | 0.012485 | CA2 |
| SLC27A5 | 0.589623 | 0.001295 | 0.012571 | CA2 |
| ARMC7 | 0.603573 | 0.001301 | 0.012617 | CA2 |
| CSMD3 | -0.65962 | 0.001303 | 0.012627 | CA2 |
| ZSCAN23 | -0.59771 | 0.001318 | 0.012722 | CA2 |
| CCDC85B | 0.815612 | 0.001343 | 0.012937 | CA2 |
| TRIM47 | 0.653014 | 0.001347 | 0.012952 | CA2 |
| GPR37L1 | 0.657721 | 0.001354 | 0.012991 | CA2 |
| ZNF793 | -0.62094 | 0.001363 | 0.013046 | CA2 |
| ZNF296 | 0.796581 | 0.001365 | 0.01306 | CA2 |
| TRMT112 | 0.602806 | 0.001369 | 0.013082 | CA2 |
| ASAH2B | -0.76804 | 0.00137 | 0.013083 | CA2 |
| TLR9 | 1.090289 | 0.001371 | 0.013083 | CA2 |
| LENG9 | 0.64399 | 0.001389 | 0.013213 | CA2 |
| RAB4B-EGLN2 | 0.746244 | 0.001406 | 0.013338 | CA2 |
| ZFPM1 | 0.756547 | 0.001426 | 0.013482 | CA2 |
| GLIS2 | 0.676314 | 0.001432 | 0.013508 | CA2 |
| PLPP3 | 0.59856 | 0.001431 | 0.013508 | CA2 |
| TRMT13 | -0.6608 | 0.001434 | 0.013522 | CA2 |
| SH3PXD2B | 0.767362 | 0.001463 | 0.013677 | CA2 |
| C19orf81 | 1.063436 | 0.001483 | 0.013807 | CA2 |
| HGFAC | 1.045099 | 0.001489 | 0.013845 | CA2 |
| CBR3 | 0.782451 | 0.001518 | 0.014039 | CA2 |
| XIAP | -0.91563 | 0.001521 | 0.01406 | CA2 |

| | | | | |
|---|---|---|---|---|
| RPS5 | 0.58078 | 0.00154 | 0.014187 | CA2 |
| C1orf194 | 1.122721 | 0.00155 | 0.014232 | CA2 |
| PIBF1 | -0.60307 | 0.001551 | 0.014233 | CA2 |
| SLC6A13 | 0.92182 | 0.001561 | 0.014294 | CA2 |
| GAREM2 | 0.687145 | 0.001563 | 0.014294 | CA2 |
| DMWD | 0.591614 | 0.001563 | 0.014294 | CA2 |
| BEST3 | -0.67633 | 0.001572 | 0.014328 | CA2 |
| LCN12 | 0.965306 | 0.001595 | 0.01447 | CA2 |
| LIMK1 | 0.623757 | 0.001603 | 0.014509 | CA2 |
| NDUFA2 | 0.597276 | 0.001604 | 0.014509 | CA2 |
| CNTFR | 0.726157 | 0.001648 | 0.014765 | CA2 |
| SH2B2 | 0.690073 | 0.001649 | 0.014765 | CA2 |
| ACOX2 | 0.603159 | 0.001683 | 0.014991 | CA2 |
| H2AC4 | 1.139945 | 0.001699 | 0.015097 | CA2 |
| B4GAT1 | 0.678086 | 0.001704 | 0.015135 | CA2 |
| IDH3G | 0.638119 | 0.001719 | 0.015235 | CA2 |
| TTYH3 | 0.597914 | 0.001727 | 0.015288 | CA2 |
| IFITM3 | 0.709835 | 0.001742 | 0.015374 | CA2 |
| NTSR2 | 0.904564 | 0.001751 | 0.01542 | CA2 |
| LINGO3 | 1.007275 | 0.001773 | 0.015566 | CA2 |
| SLC6A20 | 1.654685 | 0.001788 | 0.015634 | CA2 |
| SIX5 | 0.609131 | 0.001784 | 0.015634 | CA2 |
| MBLAC1 | 0.840932 | 0.001799 | 0.015701 | CA2 |
| CASP4 | -0.70102 | 0.001815 | 0.01582 | CA2 |
| FBXL15 | 0.590645 | 0.001817 | 0.015823 | CA2 |
| LRRTM4 | -0.7383 | 0.001831 | 0.015894 | CA2 |
| EVA1B | 0.879225 | 0.001837 | 0.015938 | CA2 |
| TCAP | 0.742723 | 0.001849 | 0.016002 | CA2 |
| C1GALT1 | -0.74373 | 0.001853 | 0.016018 | CA2 |
| VWA1 | 0.624713 | 0.001869 | 0.016096 | CA2 |
| CARD14 | 0.870419 | 0.001887 | 0.016191 | CA2 |
| IKBIP | -0.60266 | 0.001891 | 0.01621 | CA2 |
| TOPAZ1 | -1.54302 | 0.001904 | 0.016252 | CA2 |
| WLS | 0.707058 | 0.001906 | 0.016259 | CA2 |
| GNG10 | -0.7951 | 0.001907 | 0.016259 | CA2 |
| CPLANE2 | 0.583812 | 0.001916 | 0.016301 | CA2 |
| TUBB2A | 0.737633 | 0.001916 | 0.016301 | CA2 |
| RPRM | 1.361159 | 0.001933 | 0.016409 | CA2 |
| RHBDL1 | 0.689867 | 0.001954 | 0.016539 | CA2 |
| CLDN2 | 2.902365 | 0.001965 | 0.016614 | CA2 |
| FAM177A1 | -0.6316 | 0.001985 | 0.016719 | CA2 |
| PWWP2B | 0.581884 | 0.001987 | 0.016721 | CA2 |
| CCDC89 | 0.989178 | 0.001987 | 0.016721 | CA2 |
| SURF2 | 0.634527 | 0.001996 | 0.016766 | CA2 |

| | | | | |
|---|---|---|---|---|
| CACNA2D1 | -0.60149 | 0.002001 | 0.016785 | CA2 |
| PLEKHF1 | 0.644941 | 0.002027 | 0.016947 | CA2 |
| CD36 | -1.227 | 0.002031 | 0.016948 | CA2 |
| SPATA2L | 0.626172 | 0.002039 | 0.016983 | CA2 |
| SNX6 | -0.59003 | 0.002046 | 0.017009 | CA2 |
| FAT4 | -0.61411 | 0.002048 | 0.017009 | CA2 |
| ZNF24 | -0.81636 | 0.002052 | 0.017037 | CA2 |
| CCDC110 | -0.89834 | 0.002057 | 0.017073 | CA2 |
| APOE | 0.705342 | 0.002062 | 0.017089 | CA2 |
| SP100 | -0.60354 | 0.00208 | 0.017217 | CA2 |
| GRID2IP | 0.953165 | 0.002095 | 0.017234 | CA2 |
| AKAP9 | -0.60926 | 0.0021 | 0.017249 | CA2 |
| RLBP1 | 0.62291 | 0.002132 | 0.01743 | CA2 |
| GAL3ST1 | 0.63542 | 0.002139 | 0.017461 | CA2 |
| RBMS1 | -0.59696 | 0.002138 | 0.017461 | CA2 |
| RNF144B | -0.71899 | 0.002142 | 0.017475 | CA2 |
| MESP1 | 0.728797 | 0.002151 | 0.017521 | CA2 |
| NEK3 | -0.61646 | 0.002161 | 0.017542 | CA2 |
| SLC25A28 | 0.591816 | 0.002159 | 0.017542 | CA2 |
| NDUFA13 | 0.58205 | 0.00216 | 0.017542 | CA2 |
| CDKN2D | 0.664501 | 0.002189 | 0.017751 | CA2 |
| CCDC112 | -0.63617 | 0.002216 | 0.01791 | CA2 |
| AC244197.3 | -0.72724 | 0.002267 | 0.018181 | CA2 |
| MITD1 | -0.67848 | 0.002269 | 0.018187 | CA2 |
| KCTD16 | -0.87736 | 0.002281 | 0.01827 | CA2 |
| CCP110 | -0.73621 | 0.002283 | 0.018278 | CA2 |
| FRAT1 | 0.706725 | 0.002294 | 0.018352 | CA2 |
| LFNG | 0.59332 | 0.002298 | 0.018367 | CA2 |
| SMIM1 | 1.237488 | 0.002303 | 0.018392 | CA2 |
| TMEM59L | 0.77748 | 0.002317 | 0.018453 | CA2 |
| TM2D1 | -0.58482 | 0.002326 | 0.018471 | CA2 |
| GCA | -0.65525 | 0.00233 | 0.018475 | CA2 |
| CHCHD5 | 0.580309 | 0.002333 | 0.018475 | CA2 |
| CBWD5 | -0.65017 | 0.002345 | 0.01854 | CA2 |
| PALM3 | 0.927146 | 0.002358 | 0.018613 | CA2 |
| FBLN2 | 0.869599 | 0.002363 | 0.018632 | CA2 |
| HSPB2 | 0.62359 | 0.002362 | 0.018632 | CA2 |
| SLC26A7 | -1.26569 | 0.002383 | 0.018725 | CA2 |
| EXOC3L1 | 1.142574 | 0.002398 | 0.018809 | CA2 |
| GCK | 0.935698 | 0.002404 | 0.018844 | CA2 |
| C18orf54 | -0.77219 | 0.002406 | 0.018848 | CA2 |
| MMP16 | -0.67694 | 0.002434 | 0.019016 | CA2 |
| GPR85 | -0.83242 | 0.002436 | 0.019016 | CA2 |
| SLC10A3 | 0.638078 | 0.002443 | 0.019026 | CA2 |

| | | | | |
|---|---|---|---|---|
| NPM3 | 0.621156 | 0.002447 | 0.019041 | CA2 |
| NAALAD2 | -0.61623 | 0.002468 | 0.019159 | CA2 |
| GRN | 0.614545 | 0.002475 | 0.019193 | CA2 |
| SLC25A22 | 0.846505 | 0.002493 | 0.019297 | CA2 |
| ZNF841 | -0.83635 | 0.002506 | 0.019353 | CA2 |
| CYP26B1 | 0.716945 | 0.002508 | 0.019357 | CA2 |
| TPP1 | 0.580554 | 0.002514 | 0.019394 | CA2 |
| AC004832.3 | -1.79454 | 0.002539 | 0.019518 | CA2 |
| ULK1 | 0.602815 | 0.002561 | 0.019601 | CA2 |
| FZD8 | 0.738311 | 0.002559 | 0.019601 | CA2 |
| ZSWIM9 | 0.602336 | 0.002579 | 0.019677 | CA2 |
| SARDH | 0.662713 | 0.002597 | 0.019764 | CA2 |
| C5orf34 | -0.92924 | 0.002609 | 0.019837 | CA2 |
| NEURL2 | 0.67756 | 0.002638 | 0.019984 | CA2 |
| CPLX1 | 0.803788 | 0.002648 | 0.020032 | CA2 |
| NMB | 0.812221 | 0.00267 | 0.020133 | CA2 |
| PIK3R2 | 0.654224 | 0.002691 | 0.020231 | CA2 |
| ASPM | -1.46829 | 0.00274 | 0.020479 | CA2 |
| ZNF738 | -0.70483 | 0.002745 | 0.020479 | CA2 |
| QSER1 | -0.5982 | 0.002757 | 0.020549 | CA2 |
| CTBS | -0.71491 | 0.002788 | 0.020716 | CA2 |
| ENPEP | -0.95968 | 0.002798 | 0.020742 | CA2 |
| CDC7 | -0.58354 | 0.002817 | 0.020826 | CA2 |
| NHLRC3 | -0.59821 | 0.002832 | 0.020849 | CA2 |
| MMP28 | 0.733217 | 0.00283 | 0.020849 | CA2 |
| SUMO4 | -0.581 | 0.002849 | 0.020951 | CA2 |
| TCEAL6 | 0.761184 | 0.002849 | 0.020951 | CA2 |
| GMPR | 0.737665 | 0.002852 | 0.020952 | CA2 |
| FAM89B | 0.638284 | 0.002851 | 0.020952 | CA2 |
| PLPPR2 | 0.674676 | 0.002888 | 0.021144 | CA2 |
| CYS1 | 0.595473 | 0.002889 | 0.021144 | CA2 |
| ZNF678 | -0.95649 | 0.002896 | 0.021182 | CA2 |
| SLC7A10 | 0.803447 | 0.002915 | 0.021261 | CA2 |
| RHOV | 1.392331 | 0.002938 | 0.021363 | CA2 |
| CEMIP | 1.059564 | 0.002947 | 0.02137 | CA2 |
| MSANTD3-TMEFF1 | -0.87973 | 0.002943 | 0.02137 | CA2 |
| CD320 | 0.592388 | 0.00295 | 0.021384 | CA2 |
| H3C1 | 0.979139 | 0.002959 | 0.021418 | CA2 |
| CD81 | 0.631731 | 0.002967 | 0.021445 | CA2 |
| DUSP19 | -0.83934 | 0.002968 | 0.021445 | CA2 |
| TGFBR3L | 1.156194 | 0.002967 | 0.021445 | CA2 |
| FBXL19 | 0.647537 | 0.003002 | 0.021631 | CA2 |
| DIRAS1 | 0.668385 | 0.003003 | 0.021631 | CA2 |
| FA2H | 0.744298 | 0.003012 | 0.02166 | CA2 |

| | | | | |
|---|---|---|---|---|
| CXCL8 | -1.55108 | 0.003021 | 0.021692 | CA2 |
| SCRT1 | 0.879409 | 0.00304 | 0.021757 | CA2 |
| CEP290 | -0.68629 | 0.003045 | 0.02178 | CA2 |
| ARF1 | 0.833386 | 0.003046 | 0.021781 | CA2 |
| TUBA1B | 0.590822 | 0.003072 | 0.0219 | CA2 |
| DAGLA | 0.655697 | 0.003069 | 0.0219 | CA2 |
| DMD | -0.59629 | 0.003093 | 0.02203 | CA2 |
| TOMM34 | 0.774115 | 0.003126 | 0.022123 | CA2 |
| FILIP1 | -0.63807 | 0.00312 | 0.022123 | CA2 |
| SLC22A8 | 3.980094 | 0.003127 | 0.022123 | CA2 |
| INKA1 | 0.845046 | 0.003128 | 0.022123 | CA2 |
| RNF152 | -0.71966 | 0.003155 | 0.02221 | CA2 |
| RNF43 | 0.680995 | 0.003169 | 0.022264 | CA2 |
| NUDT16L1 | 0.659564 | 0.003193 | 0.02237 | CA2 |
| CTXN1 | 0.848643 | 0.003201 | 0.022404 | CA2 |
| GLI1 | 1.730976 | 0.003211 | 0.022445 | CA2 |
| C17orf100 | 0.581103 | 0.003229 | 0.022521 | CA2 |
| DENND3 | -0.59033 | 0.003243 | 0.022582 | CA2 |
| B3GALT2 | -0.68526 | 0.003242 | 0.022582 | CA2 |
| ZNF714 | -0.75978 | 0.003246 | 0.022593 | CA2 |
| TLR2 | -0.98544 | 0.003284 | 0.022754 | CA2 |
| UFSP1 | 0.845433 | 0.003284 | 0.022754 | CA2 |
| SSX2IP | -0.5877 | 0.003298 | 0.022822 | CA2 |
| UGCG | -0.66123 | 0.003297 | 0.022822 | CA2 |
| CCDC122 | -0.87015 | 0.00331 | 0.022862 | CA2 |
| GMFB | -0.72239 | 0.003307 | 0.022862 | CA2 |
| AMACR | -0.58889 | 0.003316 | 0.022899 | CA2 |
| SEPTIN7 | -0.5895 | 0.003319 | 0.022901 | CA2 |
| NDUFA5 | -0.62032 | 0.003338 | 0.022981 | CA2 |
| PCDHGB4 | 1.051575 | 0.003341 | 0.022981 | CA2 |
| CAPS | 0.739811 | 0.003345 | 0.022982 | CA2 |
| BAG3 | 0.712044 | 0.003348 | 0.022985 | CA2 |
| OAF | 0.651604 | 0.003351 | 0.022985 | CA2 |
| PSTPIP2 | -0.7816 | 0.003361 | 0.023035 | CA2 |
| PNMA8B | 0.777463 | 0.003366 | 0.023058 | CA2 |
| BCL2L12 | 0.611963 | 0.003379 | 0.023106 | CA2 |
| ENTPD2 | 0.714948 | 0.00339 | 0.023165 | CA2 |
| CENPU | -1.0521 | 0.003406 | 0.023255 | CA2 |
| PRELP | 0.744364 | 0.003418 | 0.023317 | CA2 |
| NEK10 | -0.65554 | 0.003487 | 0.023645 | CA2 |
| SHTN1 | -0.71301 | 0.003488 | 0.023645 | CA2 |
| NPY1R | -1.13213 | 0.003498 | 0.023681 | CA2 |
| ZNF583 | -0.74807 | 0.003499 | 0.023681 | CA2 |
| PDE11A | -0.76408 | 0.003504 | 0.023695 | CA2 |

| | | | | |
|---|---|---|---|---|
| ADGRA1 | 0.69258 | 0.003503 | 0.023695 | CA2 |
| REEP6 | 0.603605 | 0.003589 | 0.024139 | CA2 |
| CLU | 0.718945 | 0.003595 | 0.024167 | CA2 |
| PYROXD1 | -0.58852 | 0.0036 | 0.024174 | CA2 |
| SYT12 | 0.67481 | 0.0036 | 0.024174 | CA2 |
| FAM133B | -0.60387 | 0.0036 | 0.024174 | CA2 |
| H2AC19 | 0.795809 | 0.003614 | 0.02425 | CA2 |
| STXBP5L | -0.69827 | 0.003618 | 0.024262 | CA2 |
| NRROS | 0.601855 | 0.003626 | 0.024294 | CA2 |
| CFAP69 | -0.72485 | 0.003669 | 0.024508 | CA2 |
| AMIGO3 | 1.591031 | 0.003669 | 0.024508 | CA2 |
| ZFP30 | -0.68205 | 0.003692 | 0.024624 | CA2 |
| CAMK2N2 | 0.909639 | 0.003694 | 0.024624 | CA2 |
| ZNF766 | -0.68069 | 0.003694 | 0.024624 | CA2 |
| EFCAB5 | -0.68352 | 0.003708 | 0.024685 | CA2 |
| SAMD9 | -0.58824 | 0.003721 | 0.02471 | CA2 |
| H3-3A | -0.65182 | 0.003749 | 0.024778 | CA2 |
| CENPE | -0.73485 | 0.003765 | 0.024854 | CA2 |
| ATP1A4 | 0.906797 | 0.00385 | 0.02527 | CA2 |
| ZNF117 | -0.74336 | 0.00387 | 0.025373 | CA2 |
| NEK8 | 0.70797 | 0.003881 | 0.025414 | CA2 |
| C2CD6 | -0.773 | 0.003896 | 0.025477 | CA2 |
| AC008764.1 | 1.770581 | 0.003975 | 0.025853 | CA2 |
| S100A2 | 1.38382 | 0.003993 | 0.025905 | CA2 |
| SNX10 | -0.75517 | 0.004011 | 0.025989 | CA2 |
| KCNQ4 | 0.691303 | 0.004014 | 0.026003 | CA2 |
| FGF8 | 1.465662 | 0.004044 | 0.026121 | CA2 |
| EPHA7 | -0.82884 | 0.004065 | 0.026217 | CA2 |
| SERPINH1 | 0.939374 | 0.004121 | 0.02646 | CA2 |
| ABHD8 | 0.604348 | 0.004136 | 0.026526 | CA2 |
| TRAK2 | -0.61209 | 0.004149 | 0.026597 | CA2 |
| TLR10 | -0.96532 | 0.004163 | 0.02663 | CA2 |
| KCTD4 | -0.7263 | 0.004198 | 0.026742 | CA2 |
| PLIN4 | 0.596622 | 0.004205 | 0.026769 | CA2 |
| GJC2 | 0.709355 | 0.004236 | 0.026931 | CA2 |
| HLA-DRB5 | 2.01992 | 0.004294 | 0.027123 | CA2 |
| RARRES2 | 0.602091 | 0.004301 | 0.027135 | CA2 |
| H3C13 | 0.938633 | 0.004304 | 0.027135 | CA2 |
| SINHCAF | -0.73329 | 0.004348 | 0.027297 | CA2 |
| FTL | 0.59538 | 0.004354 | 0.027305 | CA2 |
| IGSF21 | 0.610711 | 0.004395 | 0.027462 | CA2 |
| CHRM4 | 0.997659 | 0.004398 | 0.027464 | CA2 |
| CSF3 | -2.56145 | 0.004435 | 0.027619 | CA2 |
| CBR1 | 0.608692 | 0.004534 | 0.028065 | CA2 |

| | | | | |
|---|---|---|---|---|
| RWDD1 | -0.59829 | 0.004581 | 0.028255 | CA2 |
| HLA-DQA1 | 1.853716 | 0.004593 | 0.028281 | CA2 |
| CCN5 | 1.212438 | 0.004609 | 0.028358 | CA2 |
| SCNN1A | 2.091473 | 0.00463 | 0.028462 | CA2 |
| SYNGR3 | 0.811507 | 0.004647 | 0.028528 | CA2 |
| CHMP2B | -0.58194 | 0.004668 | 0.028611 | CA2 |
| MPV17L2 | 0.622757 | 0.004706 | 0.028803 | CA2 |
| CDA | 1.13903 | 0.004744 | 0.028993 | CA2 |
| PRR36 | 0.796739 | 0.004744 | 0.028993 | CA2 |
| ANO3 | -0.84439 | 0.004767 | 0.029113 | CA2 |
| SYCP2L | -0.60585 | 0.00484 | 0.029392 | CA2 |
| TMEM106B | -0.58948 | 0.004858 | 0.029445 | CA2 |
| RGPD4 | -1.00799 | 0.004957 | 0.029825 | CA2 |
| ZNF33A | -0.60075 | 0.004974 | 0.029904 | CA2 |
| ACY3 | 1.397729 | 0.005033 | 0.03019 | CA2 |
| ETFRF1 | -0.60989 | 0.005037 | 0.030203 | CA2 |
| AC233723.1 | 1.100101 | 0.005046 | 0.030228 | CA2 |
| SERPING1 | 0.704765 | 0.005064 | 0.0303 | CA2 |
| ZBTB43 | -0.77336 | 0.005074 | 0.030336 | CA2 |
| MT1E | 0.759014 | 0.005072 | 0.030336 | CA2 |
| TMEM88B | 0.886656 | 0.005111 | 0.030434 | CA2 |
| STK17B | -0.64377 | 0.005136 | 0.03053 | CA2 |
| LEPR | -0.60493 | 0.005176 | 0.030708 | CA2 |
| C1QL1 | 0.665863 | 0.00519 | 0.030763 | CA2 |
| KIAA1586 | -0.74369 | 0.005223 | 0.030948 | CA2 |
| CDH23 | 0.671827 | 0.005242 | 0.031025 | CA2 |
| MAB21L1 | 1.216283 | 0.005246 | 0.031037 | CA2 |
| ARX | 0.582477 | 0.005298 | 0.031242 | CA2 |
| CHST1 | 0.628832 | 0.005309 | 0.031263 | CA2 |
| ZNF780B | -0.66518 | 0.005318 | 0.031293 | CA2 |
| FAM43B | 0.58959 | 0.005327 | 0.031336 | CA2 |
| FABP6 | 1.034397 | 0.005367 | 0.031496 | CA2 |
| DRD4 | 0.811863 | 0.005397 | 0.031555 | CA2 |
| MAP1LC3B2 | 0.594388 | 0.005412 | 0.031621 | CA2 |
| TLR6 | -0.65685 | 0.005424 | 0.031667 | CA2 |
| ZNF41 | -0.7632 | 0.005459 | 0.031802 | CA2 |
| BAIAP2L2 | 0.627943 | 0.005511 | 0.032004 | CA2 |
| MAP1S | 0.665624 | 0.00551 | 0.032004 | CA2 |
| IL1RAPL1 | -0.5867 | 0.005557 | 0.032168 | CA2 |
| ZNHIT2 | 0.61788 | 0.0056 | 0.032321 | CA2 |
| TSPO | 0.626799 | 0.005604 | 0.032336 | CA2 |
| APOM | 0.927311 | 0.005609 | 0.032336 | CA2 |
| CHRNA5 | 1.217282 | 0.005637 | 0.032435 | CA2 |
| TMPRSS3 | 1.210858 | 0.005649 | 0.032491 | CA2 |

| | | | | |
|---|---|---|---|---|
| AC093525.2 | 0.708263 | 0.005749 | 0.03296 | CA2 |
| FZD2 | 0.810507 | 0.005762 | 0.033011 | CA2 |
| TOX2 | 0.612428 | 0.005785 | 0.033084 | CA2 |
| CDK6 | -0.70955 | 0.005793 | 0.033122 | CA2 |
| ARSL | 0.662596 | 0.005833 | 0.03329 | CA2 |
| RHBDD2 | 0.709056 | 0.005836 | 0.033293 | CA2 |
| METTL25 | -0.59091 | 0.005877 | 0.033424 | CA2 |
| TMEM240 | 0.624636 | 0.005893 | 0.033493 | CA2 |
| PLPPR3 | 0.86752 | 0.005906 | 0.033557 | CA2 |
| SLC28A1 | 1.389207 | 0.005964 | 0.033794 | CA2 |
| SLC25A10 | 0.653757 | 0.005965 | 0.033794 | CA2 |
| ZNF518A | -0.73482 | 0.005978 | 0.033836 | CA2 |
| FGF9 | -0.94681 | 0.006004 | 0.033956 | CA2 |
| MAP3K10 | 0.634592 | 0.006024 | 0.034044 | CA2 |
| NPIPB12 | -0.78068 | 0.006083 | 0.034291 | CA2 |
| CSDC2 | 0.6591 | 0.006084 | 0.034291 | CA2 |
| MCTP1 | -0.5916 | 0.006115 | 0.034442 | CA2 |
| WASHC1 | 0.683679 | 0.006131 | 0.034497 | CA2 |
| MFSD5 | 0.59372 | 0.006165 | 0.034593 | CA2 |
| REEP3 | -0.69866 | 0.006242 | 0.034869 | CA2 |
| LRRC7 | -0.63908 | 0.00625 | 0.034894 | CA2 |
| GUCY1A1 | -0.58646 | 0.006328 | 0.035216 | CA2 |
| LRRC63 | -0.85061 | 0.006347 | 0.035271 | CA2 |
| CRYAB | 0.662641 | 0.006402 | 0.035482 | CA2 |
| GRIK5 | 0.616175 | 0.00645 | 0.035654 | CA2 |
| RAB3IP | -0.63533 | 0.006524 | 0.035926 | CA2 |
| ACKR3 | 0.717559 | 0.006541 | 0.035981 | CA2 |
| C9orf24 | 0.583074 | 0.006601 | 0.036205 | CA2 |
| KCNK12 | 0.639823 | 0.006608 | 0.036231 | CA2 |
| MMP11 | 0.838459 | 0.006634 | 0.03635 | CA2 |
| KANK2 | 0.595602 | 0.006651 | 0.036433 | CA2 |
| SYPL2 | 0.740946 | 0.006667 | 0.036484 | CA2 |
| CDH22 | 0.580824 | 0.006667 | 0.036484 | CA2 |
| CPNE6 | 0.615813 | 0.006698 | 0.036594 | CA2 |
| CALY | 0.807219 | 0.006736 | 0.036713 | CA2 |
| TMEM198 | 0.637026 | 0.006753 | 0.036792 | CA2 |
| C4A | 0.851687 | 0.006755 | 0.036792 | CA2 |
| LINGO1 | 0.787505 | 0.006787 | 0.036917 | CA2 |
| BCAP29 | -0.58475 | 0.006795 | 0.036948 | CA2 |
| CCNT2 | -0.5894 | 0.006952 | 0.037639 | CA2 |
| ANKRD22 | -1.61234 | 0.007089 | 0.038213 | CA2 |
| ARL8B | -0.62967 | 0.007119 | 0.038314 | CA2 |
| GPX3 | 0.837267 | 0.007138 | 0.038392 | CA2 |
| ARHGAP29 | -0.70148 | 0.007149 | 0.038426 | CA2 |

| | | | | |
|---|---|---|---|---|
| AEBP1 | 0.729327 | 0.007193 | 0.038545 | CA2 |
| TLCD5 | -0.74226 | 0.007208 | 0.038577 | CA2 |
| B9D2 | 0.67166 | 0.007217 | 0.038599 | CA2 |
| VN1R1 | -0.6483 | 0.007234 | 0.038634 | CA2 |
| GPR65 | -0.88348 | 0.00728 | 0.038836 | CA2 |
| RGS9BP | 0.734019 | 0.007339 | 0.039045 | CA2 |
| LY6H | 0.70168 | 0.007365 | 0.039149 | CA2 |
| H2AC21 | 0.649476 | 0.007387 | 0.039214 | CA2 |
| RAMP2 | 0.84478 | 0.007419 | 0.039295 | CA2 |
| CCKBR | 0.80456 | 0.007448 | 0.039395 | CA2 |
| STX1B | 0.676548 | 0.007482 | 0.039513 | CA2 |
| CD72 | 0.711166 | 0.007516 | 0.039615 | CA2 |
| DCP2 | -0.65527 | 0.007522 | 0.039615 | CA2 |
| ZP3 | 0.719846 | 0.007524 | 0.039615 | CA2 |
| ZNF534 | -0.74634 | 0.007539 | 0.039631 | CA2 |
| AURKA | -0.71563 | 0.007607 | 0.039911 | CA2 |
| GAS2L3 | -0.65154 | 0.007622 | 0.039966 | CA2 |
| GYG2 | 0.651978 | 0.007707 | 0.040347 | CA2 |
| ARHGAP11A | -0.64309 | 0.007719 | 0.040359 | CA2 |
| S100A10 | 0.72305 | 0.007754 | 0.040439 | CA2 |
| SAMD12 | -0.76225 | 0.007833 | 0.040749 | CA2 |
| RPL39 | 0.73625 | 0.007866 | 0.040853 | CA2 |
| ITGA1 | -0.64402 | 0.007879 | 0.040901 | CA2 |
| FGF12 | -0.61474 | 0.007918 | 0.04098 | CA2 |
| SEZ6L2 | 0.684 | 0.007924 | 0.040997 | CA2 |
| LINC00634 | 0.641706 | 0.00794 | 0.041051 | CA2 |
| SDSL | 0.760772 | 0.007948 | 0.041053 | CA2 |
| FEZF2 | 0.855594 | 0.00796 | 0.041084 | CA2 |
| IL18 | -0.7604 | 0.007974 | 0.041114 | CA2 |
| TNC | 0.768506 | 0.007978 | 0.041118 | CA2 |
| ITGB3BP | -0.60739 | 0.008039 | 0.041251 | CA2 |
| DUSP2 | 0.862345 | 0.008077 | 0.041408 | CA2 |
| GALR1 | -0.77006 | 0.008136 | 0.041638 | CA2 |
| C9orf50 | 0.981303 | 0.008184 | 0.041771 | CA2 |
| TAS2R50 | -0.74825 | 0.008186 | 0.041771 | CA2 |
| ANKRD20A3P | -0.61666 | 0.008186 | 0.041771 | CA2 |
| SLITRK4 | -0.73246 | 0.008203 | 0.041831 | CA2 |
| GRIA2 | -0.61469 | 0.008447 | 0.042687 | CA2 |
| CLIC2 | -0.58967 | 0.008485 | 0.042827 | CA2 |
| KIF5B | -0.59812 | 0.008618 | 0.043319 | CA2 |
| ACAP1 | 0.638111 | 0.008629 | 0.04334 | CA2 |
| HSPB1 | 0.898868 | 0.008692 | 0.043546 | CA2 |
| ZNF816 | -0.58794 | 0.008691 | 0.043546 | CA2 |
| APOD | 0.621282 | 0.008932 | 0.044411 | CA2 |

| | | | | |
|---|---|---|---|---|
| C9orf16 | 0.656429 | 0.008957 | 0.044508 | CA2 |
| MOSPD1 | -0.63448 | 0.00897 | 0.044525 | CA2 |
| TCTE3 | -0.59638 | 0.008986 | 0.044555 | CA2 |
| ZFP69B | -0.68158 | 0.00903 | 0.044734 | CA2 |
| ACTA1 | 1.297868 | 0.009054 | 0.044817 | CA2 |
| BTBD19 | 0.671778 | 0.009089 | 0.044969 | CA2 |
| STX1A | 0.694217 | 0.009119 | 0.04508 | CA2 |
| TRAF3 | 0.637331 | 0.009178 | 0.045261 | CA2 |
| ADPRHL1 | 0.744287 | 0.00922 | 0.045384 | CA2 |
| GJA9 | -1.35466 | 0.009271 | 0.045526 | CA2 |
| BRCA2 | -0.82839 | 0.009411 | 0.045953 | CA2 |
| NINJ2 | 0.696031 | 0.009458 | 0.046109 | CA2 |
| F10 | 1.182662 | 0.009514 | 0.046271 | CA2 |
| TEX9 | -0.65905 | 0.009579 | 0.046531 | CA2 |
| CD93 | -0.84509 | 0.009587 | 0.046533 | CA2 |
| LRRC66 | -0.61991 | 0.009585 | 0.046533 | CA2 |
| SLC4A5 | 0.653763 | 0.009595 | 0.046556 | CA2 |
| C10orf67 | -0.60782 | 0.009636 | 0.046688 | CA2 |
| PLAC8L1 | -0.8503 | 0.009658 | 0.046736 | CA2 |
| FBXL16 | 0.783603 | 0.009706 | 0.046916 | CA2 |
| SFRP5 | 1.507339 | 0.009793 | 0.047223 | CA2 |
| SAMSN1 | -0.90198 | 0.009852 | 0.047411 | CA2 |
| ANKRD20A1 | -0.84698 | 0.009879 | 0.04747 | CA2 |
| MCOLN3 | -1.14183 | 0.009901 | 0.047503 | CA2 |
| KNL1 | -1.06879 | 0.009903 | 0.047503 | CA2 |
| ZSWIM2 | -1.24916 | 0.009901 | 0.047503 | CA2 |
| KCNJ12 | 0.785894 | 0.009899 | 0.047503 | CA2 |
| KCNS1 | 0.967264 | 0.009944 | 0.047668 | CA2 |
| PIGR | 3.56704 | 0.009946 | 0.047668 | CA2 |
| IRAK3 | -0.59881 | 0.009962 | 0.047675 | CA2 |
| HEMK1 | 0.775244 | 0.009963 | 0.047675 | CA2 |
| OLFML1 | 0.612955 | 0.010031 | 0.047933 | CA2 |
| CLDND1 | -0.60094 | 0.010124 | 0.048265 | CA2 |
| ABRAXAS1 | -0.59134 | 0.010146 | 0.04832 | CA2 |
| OMP | 1.043074 | 0.010179 | 0.04844 | CA2 |
| ZNF721 | -0.59424 | 0.010199 | 0.04851 | CA2 |
| NEFH | 1.180052 | 0.010212 | 0.048557 | CA2 |
| DPEP2 | 0.848897 | 0.010233 | 0.048583 | CA2 |
| STK31 | -0.77059 | 0.010265 | 0.048693 | CA2 |
| AC124312.1 | -1.02362 | 0.010272 | 0.048712 | CA2 |
| ELFN1 | 0.605718 | 0.010284 | 0.048755 | CA2 |
| NUP37 | -0.58449 | 0.010301 | 0.048794 | CA2 |
| CAMK4 | -0.65546 | 0.010399 | 0.049174 | CA2 |
| LGR6 | 1.21529 | 0.010494 | 0.049453 | CA2 |

| | | | | |
|---|---|---|---|---|
| SOX18 | 0.92743 | 0.010486 | 0.049453 | CA2 |
| CENPVL1 | 0.99882 | 0.010573 | 0.049764 | CA2 |
| CST7 | 1.746092 | 0.009962 | 0.047675 | CA2 |
| SULT2B1 | 2.237692 | 0.001236 | 0.012217 | CA2 |
| S1PR4 | 2.401056 | 0.000189 | 0.003692 | CA2 |
| BICDL2 | 1.904008 | 0.005337 | 0.03137 | CA2 |
| GPR82 | -1.5344 | 0.007164 | 0.038442 | CA2 |
| PPIAL4C | -1.25145 | 0.001215 | 0.01209 | CA2 |
| URGCP-MRPS24 | 3.026125 | 1.36E-05 | 0.000844 | CA2 |
| | | | | |
| ZNF408 | 0.857996 | 1.30E-09 | 2.04E-05 | CA3 |
| ZNF575 | 1.290603 | 2.72E-09 | 2.04E-05 | CA3 |
| BCL9L | 0.868152 | 3.76E-09 | 2.04E-05 | CA3 |
| CHPF | 1.16351 | 7.51E-09 | 3.05E-05 | CA3 |
| MRPS2 | 1.127689 | 3.52E-08 | 0.000114 | CA3 |
| NDUFA11 | 0.839808 | 6.02E-08 | 0.000163 | CA3 |
| GLUD2 | 0.7568 | 7.94E-08 | 0.000184 | CA3 |
| MZT2B | 1.086446 | 1.11E-07 | 0.000225 | CA3 |
| ARMC5 | 0.836527 | 1.28E-07 | 0.000231 | CA3 |
| RFX1 | 0.717298 | 1.42E-07 | 0.000232 | CA3 |
| PCSK1N | 1.696501 | 2.39E-07 | 0.000332 | CA3 |
| CLPP | 0.959869 | 2.58E-07 | 0.000332 | CA3 |
| POLR2L | 1.200467 | 2.65E-07 | 0.000332 | CA3 |
| SHISA5 | 0.902323 | 2.97E-07 | 0.000345 | CA3 |
| TESK1 | 0.707696 | 4.00E-07 | 0.000398 | CA3 |
| RPL27 | 1.258292 | 3.78E-07 | 0.000398 | CA3 |
| POM121 | 0.690105 | 4.17E-07 | 0.000398 | CA3 |
| ACTA2 | 2.590801 | 4.71E-07 | 0.000401 | CA3 |
| NAA38 | 1.233124 | 4.63E-07 | 0.000401 | CA3 |
| ZNF703 | 0.931541 | 4.93E-07 | 0.000401 | CA3 |
| DTX1 | 0.821384 | 5.68E-07 | 0.00044 | CA3 |
| TMEM205 | 0.868898 | 6.57E-07 | 0.000486 | CA3 |
| SRF | 0.638638 | 6.99E-07 | 0.000494 | CA3 |
| SOD3 | 1.564611 | 7.97E-07 | 0.000499 | CA3 |
| ZYX | 0.748042 | 7.69E-07 | 0.000499 | CA3 |
| MZT2A | 0.988624 | 1.07E-06 | 0.000643 | CA3 |
| MYH11 | 3.001257 | 1.13E-06 | 0.000656 | CA3 |
| SCARF2 | 0.825287 | 1.20E-06 | 0.000673 | CA3 |
| OTUD5 | 0.646365 | 1.44E-06 | 0.000783 | CA3 |
| EEFSEC | 0.602788 | 1.58E-06 | 0.000803 | CA3 |
| RAB11B | 0.627704 | 1.57E-06 | 0.000803 | CA3 |
| EPN1 | 0.775303 | 1.69E-06 | 0.000834 | CA3 |
| FLYWCH1 | 0.681174 | 1.90E-06 | 0.000881 | CA3 |
| RABAC1 | 1.143906 | 2.04E-06 | 0.000881 | CA3 |

| | | | | |
|---|---|---|---|---|
| EDF1 | 0.984503 | 2.33E-06 | 0.000881 | CA3 |
| LRP1 | 0.786491 | 2.09E-06 | 0.000881 | CA3 |
| UBE2M | 0.628141 | 2.54E-06 | 0.000881 | CA3 |
| POMGNT2 | 0.811617 | 2.35E-06 | 0.000881 | CA3 |
| VPS51 | 0.738847 | 2.55E-06 | 0.000881 | CA3 |
| WNT4 | 1.156691 | 2.47E-06 | 0.000881 | CA3 |
| SMARCAD1 | -0.58632 | 2.17E-06 | 0.000881 | CA3 |
| VASN | 1.177019 | 2.03E-06 | 0.000881 | CA3 |
| MED16 | 0.720368 | 2.27E-06 | 0.000881 | CA3 |
| VPS37D | 0.899341 | 1.94E-06 | 0.000881 | CA3 |
| ZFPM1 | 1.14883 | 2.47E-06 | 0.000881 | CA3 |
| CRYBG3 | -0.80715 | 2.96E-06 | 0.000981 | CA3 |
| CST3 | 1.139344 | 2.93E-06 | 0.000981 | CA3 |
| PNPLA2 | 0.936104 | 3.02E-06 | 0.000981 | CA3 |
| SLC39A13 | 0.785323 | 3.11E-06 | 0.000992 | CA3 |
| RTN4R | 1.315706 | 3.30E-06 | 0.001031 | CA3 |
| CIAO2B | 1.023167 | 3.71E-06 | 0.001118 | CA3 |
| SAC3D1 | 1.025545 | 4.14E-06 | 0.001223 | CA3 |
| TLNRD1 | 0.703901 | 4.40E-06 | 0.001233 | CA3 |
| SCAND1 | 1.25502 | 4.35E-06 | 0.001233 | CA3 |
| LRFN4 | 0.784512 | 4.33E-06 | 0.001233 | CA3 |
| TIMM13 | 0.897052 | 4.71E-06 | 0.001257 | CA3 |
| CHMP6 | 0.608455 | 4.67E-06 | 0.001257 | CA3 |
| OAF | 1.018042 | 4.66E-06 | 0.001257 | CA3 |
| CTXN1 | 1.233645 | 4.99E-06 | 0.00131 | CA3 |
| TMSB10 | 1.284709 | 5.79E-06 | 0.001404 | CA3 |
| SAP130 | 0.604558 | 5.87E-06 | 0.001404 | CA3 |
| G6PD | 0.719849 | 5.65E-06 | 0.001404 | CA3 |
| DPM3 | 0.969245 | 5.75E-06 | 0.001404 | CA3 |
| F8A3 | 0.848631 | 5.73E-06 | 0.001404 | CA3 |
| CRAT | 0.657289 | 6.46E-06 | 0.001475 | CA3 |
| NDUFB7 | 1.08024 | 7.86E-06 | 0.001475 | CA3 |
| ATN1 | 0.625743 | 7.17E-06 | 0.001475 | CA3 |
| C12orf57 | 0.949297 | 7.71E-06 | 0.001475 | CA3 |
| ELOF1 | 0.857108 | 6.78E-06 | 0.001475 | CA3 |
| RAMP1 | 1.182545 | 7.83E-06 | 0.001475 | CA3 |
| BRCA2 | -1.39549 | 6.48E-06 | 0.001475 | CA3 |
| RRAD | 1.614244 | 7.21E-06 | 0.001475 | CA3 |
| NTSR2 | 1.257579 | 6.94E-06 | 0.001475 | CA3 |
| FBXL14 | 0.750511 | 7.36E-06 | 0.001475 | CA3 |
| PC | 0.636487 | 7.88E-06 | 0.001475 | CA3 |
| MAF1 | 0.677138 | 7.98E-06 | 0.001475 | CA3 |
| NELFB | 0.623269 | 7.83E-06 | 0.001475 | CA3 |
| TSPAN4 | 0.730929 | 7.16E-06 | 0.001475 | CA3 |

| | | | | |
|---|---|---|---|---|
| CPTP | 0.801399 | 7.04E-06 | 0.001475 | CA3 |
| RPL41 | 1.602227 | 6.98E-06 | 0.001475 | CA3 |
| ZNF865 | 0.738686 | 7.43E-06 | 0.001475 | CA3 |
| AC007325.2 | 1.298469 | 7.56E-06 | 0.001475 | CA3 |
| ST6GALNAC4 | 0.691603 | 8.12E-06 | 0.001483 | CA3 |
| AL359736.1 | 1.565142 | 8.67E-06 | 0.001567 | CA3 |
| VKORC1 | 0.802975 | 9.42E-06 | 0.001683 | CA3 |
| AJM1 | 0.987288 | 1.03E-05 | 0.001822 | CA3 |
| KIAA2013 | 0.631584 | 1.08E-05 | 0.001835 | CA3 |
| AHDC1 | 0.629285 | 1.07E-05 | 0.001835 | CA3 |
| BLOC1S3 | 1.171331 | 1.07E-05 | 0.001835 | CA3 |
| ZNF777 | 0.63191 | 1.08E-05 | 0.001835 | CA3 |
| IRS2 | 0.610959 | 1.16E-05 | 0.001942 | CA3 |
| ADRA2C | 0.864557 | 1.20E-05 | 0.001986 | CA3 |
| CASTOR2 | 0.615086 | 1.21E-05 | 0.001991 | CA3 |
| ANTKMT | 1.027528 | 1.31E-05 | 0.002 | CA3 |
| C19orf53 | 1.071226 | 1.26E-05 | 0.002 | CA3 |
| MEGF8 | 0.731509 | 1.33E-05 | 0.002 | CA3 |
| EHD1 | 0.700955 | 1.25E-05 | 0.002 | CA3 |
| CNN1 | 2.659856 | 1.32E-05 | 0.002 | CA3 |
| BAHD1 | 0.58688 | 1.28E-05 | 0.002 | CA3 |
| PRIM2 | -0.86252 | 1.29E-05 | 0.002 | CA3 |
| LDB1 | 0.757291 | 1.31E-05 | 0.002 | CA3 |
| UBA52 | 0.860076 | 1.34E-05 | 0.002 | CA3 |
| CRIP2 | 0.626181 | 1.38E-05 | 0.002041 | CA3 |
| METRN | 1.049194 | 1.41E-05 | 0.002068 | CA3 |
| CAMK2N2 | 1.258451 | 1.43E-05 | 0.002077 | CA3 |
| UBTD1 | 0.636934 | 1.46E-05 | 0.002104 | CA3 |
| SLC26A11 | 0.609935 | 1.48E-05 | 0.002116 | CA3 |
| TCEA2 | 0.6064 | 1.52E-05 | 0.002148 | CA3 |
| RNPEPL1 | 0.6618 | 1.54E-05 | 0.002152 | CA3 |
| PRAF2 | 0.903006 | 1.65E-05 | 0.002278 | CA3 |
| DDX54 | 0.630359 | 1.69E-05 | 0.002306 | CA3 |
| PLEC | 0.732563 | 1.83E-05 | 0.002463 | CA3 |
| NLRX1 | 0.727313 | 1.87E-05 | 0.002481 | CA3 |
| CHCHD10 | 1.004613 | 1.88E-05 | 0.002481 | CA3 |
| KCNH2 | 0.828368 | 1.92E-05 | 0.002515 | CA3 |
| NOL8 | -0.59764 | 1.94E-05 | 0.002518 | CA3 |
| ZNF142 | 0.584694 | 1.97E-05 | 0.002531 | CA3 |
| RPS11 | 0.909835 | 2.01E-05 | 0.002556 | CA3 |
| ROMO1 | 1.180565 | 2.04E-05 | 0.002556 | CA3 |
| LRFN3 | 0.782379 | 2.03E-05 | 0.002556 | CA3 |
| MRPS24 | 0.889926 | 2.09E-05 | 0.00256 | CA3 |
| NCK2 | 0.619888 | 2.11E-05 | 0.00256 | CA3 |

| | | | | |
|---|---|---|---|---|
| CTF1 | 1.097799 | 2.12E-05 | 0.00256 | CA3 |
| UBE2L6 | 0.735885 | 2.12E-05 | 0.00256 | CA3 |
| COX8A | 1.279073 | 2.18E-05 | 0.002583 | CA3 |
| ARL2 | 0.701831 | 2.25E-05 | 0.002646 | CA3 |
| IMP3 | 1.110318 | 2.28E-05 | 0.002647 | CA3 |
| TLE5 | 0.748424 | 2.38E-05 | 0.002687 | CA3 |
| PPDPF | 1.063597 | 2.40E-05 | 0.002687 | CA3 |
| WTIP | 1.037678 | 2.38E-05 | 0.002687 | CA3 |
| THAP11 | 0.833767 | 2.41E-05 | 0.002687 | CA3 |
| MIGA1 | -0.70524 | 2.46E-05 | 0.002724 | CA3 |
| NDUFA2 | 0.809961 | 2.49E-05 | 0.002736 | CA3 |
| CHST7 | 1.100103 | 2.55E-05 | 0.002783 | CA3 |
| SHLD2 | -0.58195 | 2.64E-05 | 0.002837 | CA3 |
| METTL26 | 0.731829 | 2.65E-05 | 0.002837 | CA3 |
| PTRH1 | 0.860215 | 2.63E-05 | 0.002837 | CA3 |
| LGI4 | 0.726112 | 2.71E-05 | 0.002857 | CA3 |
| THAP4 | 0.587529 | 2.75E-05 | 0.002879 | CA3 |
| PRDX5 | 0.897894 | 2.95E-05 | 0.002955 | CA3 |
| ADRM1 | 0.645201 | 2.88E-05 | 0.002955 | CA3 |
| ITGA7 | 0.668552 | 2.96E-05 | 0.002955 | CA3 |
| MKI67 | -2.96487 | 2.95E-05 | 0.002955 | CA3 |
| RAVER1 | 0.625938 | 2.96E-05 | 0.002955 | CA3 |
| RNF208 | 1.034022 | 2.92E-05 | 0.002955 | CA3 |
| ZSWIM4 | 0.686817 | 3.13E-05 | 0.003067 | CA3 |
| MRPL4 | 0.679137 | 3.22E-05 | 0.003132 | CA3 |
| ATP6V1F | 0.906758 | 3.37E-05 | 0.003258 | CA3 |
| DNAL4 | 0.92159 | 3.43E-05 | 0.003285 | CA3 |
| CPLANE2 | 0.747764 | 3.55E-05 | 0.003372 | CA3 |
| SRM | 0.756979 | 3.57E-05 | 0.003379 | CA3 |
| ANAPC4 | -0.58916 | 3.70E-05 | 0.003438 | CA3 |
| MLST8 | 0.681642 | 3.68E-05 | 0.003438 | CA3 |
| PAMR1 | 0.795359 | 3.76E-05 | 0.003443 | CA3 |
| HRAS | 0.600067 | 3.75E-05 | 0.003443 | CA3 |
| PRELP | 1.136678 | 3.99E-05 | 0.003601 | CA3 |
| CLEC11A | 1.120848 | 4.08E-05 | 0.003627 | CA3 |
| ITGA3 | 0.619708 | 4.22E-05 | 0.003631 | CA3 |
| MYL9 | 2.225113 | 4.31E-05 | 0.003631 | CA3 |
| LTBP2 | 1.388458 | 4.23E-05 | 0.003631 | CA3 |
| GLIS2 | 0.780867 | 4.32E-05 | 0.003631 | CA3 |
| PRR7 | 1.008872 | 4.17E-05 | 0.003631 | CA3 |
| SMC2 | -0.62128 | 4.13E-05 | 0.003631 | CA3 |
| ZNF300 | -1.00242 | 4.31E-05 | 0.003631 | CA3 |
| NDUFA3 | 0.756009 | 4.22E-05 | 0.003631 | CA3 |
| RTN4RL2 | 1.334214 | 4.18E-05 | 0.003631 | CA3 |

| | | | | |
|---|---|---|---|---|
| POLK | -0.63828 | 4.44E-05 | 0.003687 | CA3 |
| CERS1 | 0.890327 | 4.44E-05 | 0.003687 | CA3 |
| NACC1 | 0.637713 | 4.55E-05 | 0.003753 | CA3 |
| SLC22A17 | 0.820231 | 4.58E-05 | 0.003762 | CA3 |
| SSC5D | 0.930054 | 4.64E-05 | 0.003789 | CA3 |
| SEPTIN1 | 1.183492 | 4.71E-05 | 0.003826 | CA3 |
| LRRC24 | 0.795042 | 4.75E-05 | 0.003842 | CA3 |
| AOC3 | 1.496603 | 4.97E-05 | 0.00396 | CA3 |
| PGM1 | 0.655703 | 5.14E-05 | 0.004062 | CA3 |
| UHRF2 | -0.58535 | 5.15E-05 | 0.004062 | CA3 |
| AGFG2 | 0.628971 | 5.20E-05 | 0.004066 | CA3 |
| KNL1 | -1.69869 | 5.30E-05 | 0.004066 | CA3 |
| ADRA1B | 1.713279 | 5.32E-05 | 0.004066 | CA3 |
| MITD1 | -0.78235 | 5.50E-05 | 0.004104 | CA3 |
| ZNF771 | 0.775359 | 5.45E-05 | 0.004104 | CA3 |
| C4orf48 | 1.526447 | 5.54E-05 | 0.00411 | CA3 |
| SHARPIN | 0.587419 | 5.64E-05 | 0.004149 | CA3 |
| TAF6 | 0.581945 | 5.77E-05 | 0.004227 | CA3 |
| CEP63 | -0.58224 | 5.81E-05 | 0.004239 | CA3 |
| LINGO3 | 1.229796 | 5.84E-05 | 0.004241 | CA3 |
| UBE2S | 0.909816 | 6.04E-05 | 0.004348 | CA3 |
| RPF2 | -0.72253 | 6.10E-05 | 0.004369 | CA3 |
| CYSTM1 | 0.664238 | 6.16E-05 | 0.004392 | CA3 |
| DDA1 | 1.423305 | 6.26E-05 | 0.004423 | CA3 |
| KLHL34 | 1.985816 | 6.43E-05 | 0.004523 | CA3 |
| SF3B5 | 0.968599 | 6.71E-05 | 0.004702 | CA3 |
| PPP1R1A | 0.997852 | 6.88E-05 | 0.004759 | CA3 |
| PLPPR2 | 0.890611 | 6.94E-05 | 0.00476 | CA3 |
| MICOS13 | 0.681283 | 6.99E-05 | 0.004777 | CA3 |
| H2AX | 0.727352 | 7.03E-05 | 0.004782 | CA3 |
| PLEKHA1 | -0.74765 | 7.15E-05 | 0.004787 | CA3 |
| NAT8L | 0.709203 | 7.10E-05 | 0.004787 | CA3 |
| MDM4 | -0.67422 | 7.14E-05 | 0.004787 | CA3 |
| POLRMT | 0.580773 | 7.23E-05 | 0.0048 | CA3 |
| FBXW5 | 0.70797 | 7.21E-05 | 0.0048 | CA3 |
| OBSL1 | 0.599143 | 7.28E-05 | 0.004813 | CA3 |
| CCDC124 | 0.638685 | 7.40E-05 | 0.004854 | CA3 |
| NDUFA1 | 1.104309 | 7.37E-05 | 0.004854 | CA3 |
| ZNF419 | -0.67897 | 7.55E-05 | 0.004872 | CA3 |
| DES | 3.563794 | 7.54E-05 | 0.004872 | CA3 |
| TTLL7 | -0.91748 | 7.98E-05 | 0.0051 | CA3 |
| FBXL15 | 0.823318 | 8.04E-05 | 0.005109 | CA3 |
| ATM | -0.74781 | 8.42E-05 | 0.005267 | CA3 |
| HSPB2 | 1.036162 | 8.37E-05 | 0.005267 | CA3 |

| | | | | |
|---|---|---|---|---|
| ALYREF | 0.812021 | 8.41E-05 | 0.005267 | CA3 |
| INTS5 | 0.771665 | 8.61E-05 | 0.005363 | CA3 |
| MMP9 | 3.896285 | 8.82E-05 | 0.005455 | CA3 |
| CCDC71 | 0.61951 | 8.99E-05 | 0.005515 | CA3 |
| DUSP23 | 1.181902 | 9.28E-05 | 0.005567 | CA3 |
| TP53I11 | 1.129047 | 9.29E-05 | 0.005567 | CA3 |
| GTPBP6 | 0.61247 | 9.28E-05 | 0.005567 | CA3 |
| CDH4 | 0.648708 | 9.25E-05 | 0.005567 | CA3 |
| ARHGDIA | 0.580447 | 9.59E-05 | 0.005651 | CA3 |
| VPS13C | -0.60568 | 9.68E-05 | 0.005684 | CA3 |
| MIF | 1.034312 | 9.75E-05 | 0.005701 | CA3 |
| ABHD8 | 0.794413 | 9.97E-05 | 0.005789 | CA3 |
| PSMA7 | 0.671773 | 0.0001 | 0.005814 | CA3 |
| SGK3 | -0.90188 | 0.000101 | 0.005832 | CA3 |
| EMP3 | 0.922517 | 0.000104 | 0.005895 | CA3 |
| RPL37A | 0.777408 | 0.000103 | 0.005895 | CA3 |
| RPL28 | 0.63393 | 0.000106 | 0.005908 | CA3 |
| TMEM54 | 0.991574 | 0.000107 | 0.005916 | CA3 |
| HSPB6 | 1.210408 | 0.000111 | 0.005986 | CA3 |
| ZNF506 | -0.68748 | 0.000109 | 0.005986 | CA3 |
| MT2A | 1.151535 | 0.000111 | 0.005986 | CA3 |
| LIPT1 | -1.07536 | 0.000111 | 0.005986 | CA3 |
| FAM171A2 | 1.001342 | 0.00011 | 0.005986 | CA3 |
| MFAP4 | 1.37382 | 0.000111 | 0.005986 | CA3 |
| VAMP5 | 1.035553 | 0.000112 | 0.005986 | CA3 |
| TMEM160 | 0.963767 | 0.000114 | 0.006024 | CA3 |
| IRF2BP1 | 0.584425 | 0.000114 | 0.006024 | CA3 |
| AC024592.3 | 1.364496 | 0.000122 | 0.00635 | CA3 |
| TCF7L1 | 0.762755 | 0.000123 | 0.006381 | CA3 |
| IRS1 | 0.719345 | 0.000124 | 0.0064 | CA3 |
| FAM89B | 0.757807 | 0.000124 | 0.0064 | CA3 |
| ADAMTS9 | -1.18862 | 0.000126 | 0.006441 | CA3 |
| TMUB1 | 0.594492 | 0.000128 | 0.006509 | CA3 |
| TAF1D | -0.64868 | 0.000129 | 0.006509 | CA3 |
| ULK1 | 0.751135 | 0.000129 | 0.006509 | CA3 |
| PPP2R3C | -0.58351 | 0.00013 | 0.006535 | CA3 |
| PUS7L | -0.59083 | 0.000132 | 0.006545 | CA3 |
| AC011005.1 | 1.187952 | 0.000132 | 0.006554 | CA3 |
| FAM107B | -0.99771 | 0.000133 | 0.00656 | CA3 |
| INAFM1 | 0.686696 | 0.000134 | 0.006574 | CA3 |
| AL157935.2 | 1.916597 | 0.000136 | 0.006636 | CA3 |
| SHISA4 | 0.750539 | 0.000137 | 0.006666 | CA3 |
| TECR | 0.636171 | 0.00014 | 0.006735 | CA3 |
| RARA | 0.712603 | 0.000142 | 0.006852 | CA3 |

| | | | | |
|---|---|---|---|---|
| OFD1 | -0.70071 | 0.000145 | 0.006937 | CA3 |
| TNFRSF12A | 1.266037 | 0.000148 | 0.006983 | CA3 |
| NXPE3 | -0.6237 | 0.000148 | 0.006983 | CA3 |
| CCNL1 | -0.64789 | 0.000149 | 0.006983 | CA3 |
| LMOD1 | 1.292789 | 0.00015 | 0.007 | CA3 |
| RTL8A | 0.772912 | 0.00015 | 0.007 | CA3 |
| H4C3 | 0.971122 | 0.000152 | 0.007081 | CA3 |
| MPV17L2 | 0.84588 | 0.000154 | 0.007126 | CA3 |
| PWWP2A | -0.75182 | 0.000155 | 0.007138 | CA3 |
| NR2F1 | 0.730219 | 0.000156 | 0.00718 | CA3 |
| POLR2I | 0.793371 | 0.00016 | 0.007303 | CA3 |
| ATP5ME | 0.790824 | 0.00016 | 0.007303 | CA3 |
| RAB11FIP4 | 0.659773 | 0.000161 | 0.007338 | CA3 |
| PFDN2 | 0.879921 | 0.000162 | 0.00735 | CA3 |
| PRIM1 | -0.82169 | 0.000164 | 0.007424 | CA3 |
| COL3A1 | 1.903782 | 0.000167 | 0.007483 | CA3 |
| OTX1 | 0.996129 | 0.00017 | 0.007572 | CA3 |
| FAM155B | 0.632918 | 0.000171 | 0.007622 | CA3 |
| DPM2 | 0.627226 | 0.000172 | 0.007622 | CA3 |
| DYNLRB1 | 0.872809 | 0.000173 | 0.007684 | CA3 |
| CCDC186 | -0.65498 | 0.000177 | 0.007746 | CA3 |
| CNTFR | 0.863335 | 0.000179 | 0.007794 | CA3 |
| SMOX | 0.65989 | 0.000182 | 0.007887 | CA3 |
| FLNA | 0.867477 | 0.000183 | 0.007905 | CA3 |
| LTBP4 | 0.751617 | 0.000185 | 0.007994 | CA3 |
| CCDC120 | 0.597745 | 0.000188 | 0.008048 | CA3 |
| BAX | 0.603404 | 0.000191 | 0.008057 | CA3 |
| CCDC85B | 0.890228 | 0.000191 | 0.008057 | CA3 |
| UQCR10 | 0.832787 | 0.000191 | 0.008057 | CA3 |
| IFT80 | -0.83747 | 0.000194 | 0.00817 | CA3 |
| FXYD1 | 0.861335 | 0.000196 | 0.008174 | CA3 |
| TENT5B | 1.902576 | 0.000198 | 0.008254 | CA3 |
| SOX15 | 1.272772 | 0.000202 | 0.008388 | CA3 |
| ZNF213 | 0.592279 | 0.000212 | 0.008571 | CA3 |
| CLPTM1 | 0.615399 | 0.000214 | 0.008571 | CA3 |
| SMC4 | -0.75192 | 0.000213 | 0.008571 | CA3 |
| PLCL1 | -0.66871 | 0.000214 | 0.008571 | CA3 |
| CLUH | 0.60785 | 0.000208 | 0.008571 | CA3 |
| ADIRF | 1.030832 | 0.000215 | 0.008571 | CA3 |
| CARD19 | 0.668385 | 0.00021 | 0.008571 | CA3 |
| SP2 | 0.606288 | 0.000209 | 0.008571 | CA3 |
| CLSTN1 | 0.695561 | 0.000217 | 0.008571 | CA3 |
| TMEM94 | 0.582068 | 0.000215 | 0.008571 | CA3 |
| KLHL25 | 0.600518 | 0.000213 | 0.008571 | CA3 |

| | | | | |
|---|---|---|---|---|
| BMP8A | 0.817687 | 0.000214 | 0.008571 | CA3 |
| GAS2L1 | 0.744305 | 0.000217 | 0.008571 | CA3 |
| NKAIN2 | -0.71667 | 0.000215 | 0.008571 | CA3 |
| DACT3 | 0.619197 | 0.00021 | 0.008571 | CA3 |
| RRAS | 0.898519 | 0.000221 | 0.008607 | CA3 |
| CD151 | 0.723793 | 0.00022 | 0.008607 | CA3 |
| PPP2R3A | -0.58083 | 0.000222 | 0.008614 | CA3 |
| UQCRQ | 0.746286 | 0.000223 | 0.008614 | CA3 |
| IKBIP | -0.89796 | 0.000224 | 0.008614 | CA3 |
| ZNRD2 | 0.636021 | 0.000223 | 0.008614 | CA3 |
| AKIRIN2 | 0.617078 | 0.000227 | 0.008681 | CA3 |
| SLC1A4 | 0.666492 | 0.000229 | 0.008722 | CA3 |
| MAMLD1 | 0.642699 | 0.000235 | 0.008841 | CA3 |
| SCAMP3 | 0.620629 | 0.000235 | 0.008841 | CA3 |
| ENDOG | 0.80768 | 0.000235 | 0.008841 | CA3 |
| FAU | 0.716796 | 0.000236 | 0.008859 | CA3 |
| IDH3G | 0.738977 | 0.000241 | 0.008912 | CA3 |
| RBM48 | -0.83987 | 0.000242 | 0.008912 | CA3 |
| LOXL1 | 1.325664 | 0.00024 | 0.008912 | CA3 |
| CBR4 | -0.66143 | 0.000241 | 0.008912 | CA3 |
| NOP10 | 1.146899 | 0.00024 | 0.008912 | CA3 |
| RPS18 | 0.809126 | 0.000239 | 0.008912 | CA3 |
| NDUFB11 | 0.78986 | 0.000246 | 0.009019 | CA3 |
| CELF3 | 1.12233 | 0.000249 | 0.009095 | CA3 |
| CXorf21 | -1.05363 | 0.000254 | 0.009154 | CA3 |
| UBL5 | 0.972373 | 0.000254 | 0.009154 | CA3 |
| ARID4B | -0.59284 | 0.000255 | 0.009162 | CA3 |
| LRRC4B | 0.626542 | 0.000258 | 0.009231 | CA3 |
| SGO2 | -0.86019 | 0.00026 | 0.009238 | CA3 |
| FASN | 0.649693 | 0.000259 | 0.009238 | CA3 |
| TBC1D8B | -0.69407 | 0.000262 | 0.009317 | CA3 |
| DRAP1 | 0.670661 | 0.000268 | 0.00949 | CA3 |
| DKK3 | 0.806755 | 0.00027 | 0.009509 | CA3 |
| TRAK2 | -0.73708 | 0.000272 | 0.009509 | CA3 |
| ATP1B2 | 0.76435 | 0.000274 | 0.009523 | CA3 |
| CTSD | 0.828622 | 0.000278 | 0.00954 | CA3 |
| ATP8A1 | -0.59885 | 0.000276 | 0.00954 | CA3 |
| GYPC | 0.869926 | 0.000277 | 0.00954 | CA3 |
| TRMT10B | -0.63736 | 0.000277 | 0.00954 | CA3 |
| PRPF39 | -0.59026 | 0.000276 | 0.00954 | CA3 |
| CRB2 | 0.794453 | 0.000281 | 0.009624 | CA3 |
| KATNBL1 | -0.61066 | 0.000282 | 0.00963 | CA3 |
| RHBDD2 | 1.029732 | 0.000284 | 0.009671 | CA3 |
| ZNF708 | -0.73629 | 0.000288 | 0.009769 | CA3 |

| | | | | |
|---|---|---|---|---|
| ELL2 | -0.64893 | 0.000289 | 0.009798 | CA3 |
| RPP21 | 0.799716 | 0.000292 | 0.009868 | CA3 |
| ZNF423 | 0.726403 | 0.000294 | 0.009874 | CA3 |
| DBP | 0.583091 | 0.000294 | 0.009874 | CA3 |
| TICAM1 | 0.65734 | 0.000293 | 0.009874 | CA3 |
| NXPH3 | 0.759403 | 0.000295 | 0.009874 | CA3 |
| PRR12 | 0.580453 | 0.000297 | 0.009889 | CA3 |
| EPHB1 | 0.770191 | 0.000298 | 0.009889 | CA3 |
| LRRC63 | -1.33225 | 0.000296 | 0.009889 | CA3 |
| ZNF845 | -0.7958 | 0.0003 | 0.00993 | CA3 |
| ADAMTSL5 | 1.165131 | 0.000306 | 0.010023 | CA3 |
| S100A1 | 0.821423 | 0.000309 | 0.0101 | CA3 |
| TRAPPC5 | 1.158209 | 0.000314 | 0.01023 | CA3 |
| MT3 | 0.975892 | 0.000329 | 0.010562 | CA3 |
| MDK | 1.054431 | 0.000328 | 0.010562 | CA3 |
| BORA | -0.86342 | 0.000328 | 0.010562 | CA3 |
| TATDN3 | -0.61808 | 0.000329 | 0.010562 | CA3 |
| MRGPRF | 1.350075 | 0.000344 | 0.010881 | CA3 |
| AGT | 0.86523 | 0.000346 | 0.010921 | CA3 |
| NEK3 | -0.78703 | 0.000348 | 0.010922 | CA3 |
| C4orf33 | -0.67338 | 0.000348 | 0.010922 | CA3 |
| ADCK5 | 0.662604 | 0.000349 | 0.010922 | CA3 |
| CBX3 | -0.66279 | 0.000352 | 0.010987 | CA3 |
| CSDC2 | 0.74405 | 0.000355 | 0.011019 | CA3 |
| SIGMAR1 | 0.609244 | 0.000358 | 0.011032 | CA3 |
| NCKIPSD | 0.612415 | 0.00036 | 0.011046 | CA3 |
| MRPL34 | 0.911585 | 0.000363 | 0.01105 | CA3 |
| USP11 | 0.668115 | 0.000364 | 0.011055 | CA3 |
| ZNF654 | -0.59367 | 0.000375 | 0.011246 | CA3 |
| SP100 | -0.70166 | 0.000378 | 0.011263 | CA3 |
| MEX3D | 0.663073 | 0.000377 | 0.011263 | CA3 |
| TMEM132A | 0.732518 | 0.000387 | 0.01136 | CA3 |
| MRPS34 | 0.648371 | 0.000384 | 0.01136 | CA3 |
| WDR18 | 0.618321 | 0.000392 | 0.011435 | CA3 |
| ATG4C | -0.83987 | 0.0004 | 0.011625 | CA3 |
| PDLIM4 | 0.826883 | 0.000407 | 0.011693 | CA3 |
| TIMM29 | 0.713138 | 0.000407 | 0.011693 | CA3 |
| ZNF652 | -0.68024 | 0.000407 | 0.011693 | CA3 |
| TCF7 | 0.796506 | 0.000414 | 0.011715 | CA3 |
| ZNF600 | -0.73861 | 0.000413 | 0.011715 | CA3 |
| ZNF624 | -0.72626 | 0.000412 | 0.011715 | CA3 |
| TFAP2C | 1.02106 | 0.000416 | 0.011718 | CA3 |
| GRINA | 0.733284 | 0.000417 | 0.011718 | CA3 |
| H4C11 | 1.406612 | 0.000415 | 0.011718 | CA3 |

| | | | | |
|---|---|---|---|---|
| ABHD14A | 0.789737 | 0.000418 | 0.01174 | CA3 |
| NT5M | 0.629462 | 0.000426 | 0.011917 | CA3 |
| TLR1 | -0.76135 | 0.000427 | 0.011918 | CA3 |
| TRIP11 | -0.6284 | 0.000433 | 0.012014 | CA3 |
| GCFC2 | -0.63215 | 0.000436 | 0.012025 | CA3 |
| FGFRL1 | 0.825293 | 0.000436 | 0.012025 | CA3 |
| HCN2 | 0.791794 | 0.000442 | 0.012099 | CA3 |
| RPL36AL | 0.760413 | 0.000443 | 0.012099 | CA3 |
| MTLN | 1.096794 | 0.000442 | 0.012099 | CA3 |
| CDK6 | -0.77112 | 0.000446 | 0.012158 | CA3 |
| ZNF37A | -0.598 | 0.00045 | 0.012169 | CA3 |
| MARCHF7 | -0.61409 | 0.000451 | 0.012169 | CA3 |
| RPL8 | 0.621338 | 0.00045 | 0.012169 | CA3 |
| PRAG1 | 0.679421 | 0.000449 | 0.012169 | CA3 |
| TMEM184B | 0.71104 | 0.000455 | 0.012257 | CA3 |
| C1QTNF4 | 1.089749 | 0.000459 | 0.012332 | CA3 |
| FAM163B | 1.266317 | 0.000462 | 0.012369 | CA3 |
| RHOBTB2 | 0.588438 | 0.000467 | 0.012464 | CA3 |
| DAGLA | 0.785231 | 0.000468 | 0.012464 | CA3 |
| ZNF644 | -0.70393 | 0.000471 | 0.01248 | CA3 |
| DUSP15 | 0.708301 | 0.000474 | 0.012519 | CA3 |
| FBLN2 | 1.082839 | 0.000475 | 0.012525 | CA3 |
| ZDHHC20 | -0.80803 | 0.000476 | 0.012525 | CA3 |
| FAM43B | 0.639494 | 0.00048 | 0.012577 | CA3 |
| MTCH1 | 0.587129 | 0.000484 | 0.012649 | CA3 |
| POP7 | 0.720096 | 0.000485 | 0.012653 | CA3 |
| TAGLN | 1.357697 | 0.000491 | 0.012757 | CA3 |
| CHST5 | 0.928432 | 0.000496 | 0.012833 | CA3 |
| HSD17B10 | 0.654865 | 0.000505 | 0.012926 | CA3 |
| FAM83H | 0.69525 | 0.000506 | 0.012926 | CA3 |
| LSM10 | 0.591519 | 0.000505 | 0.012926 | CA3 |
| SMIM29 | 0.581622 | 0.000506 | 0.012926 | CA3 |
| FBLL1 | 1.149522 | 0.000507 | 0.012938 | CA3 |
| LRRC8A | 0.624917 | 0.00051 | 0.012971 | CA3 |
| SLC25A34 | 0.801775 | 0.000522 | 0.013032 | CA3 |
| NMB | 0.937447 | 0.00052 | 0.013032 | CA3 |
| MFSD3 | 0.816098 | 0.000524 | 0.013054 | CA3 |
| CEP97 | -0.65078 | 0.000524 | 0.013054 | CA3 |
| ZSCAN30 | -0.60447 | 0.000525 | 0.013058 | CA3 |
| CACNG7 | 0.637354 | 0.000528 | 0.013061 | CA3 |
| TBC1D10A | 0.638898 | 0.000532 | 0.013113 | CA3 |
| GREM1 | -1.32953 | 0.000531 | 0.013113 | CA3 |
| REEP6 | 0.757467 | 0.000538 | 0.013135 | CA3 |
| SNX6 | -0.64282 | 0.000537 | 0.013135 | CA3 |

| | | | | |
|---|---|---|---|---|
| RAPGEF5 | -0.84035 | 0.00054 | 0.013135 | CA3 |
| ZNF283 | -0.6068 | 0.000538 | 0.013135 | CA3 |
| RAC3 | 0.9093 | 0.000538 | 0.013135 | CA3 |
| DISP3 | 0.926782 | 0.000539 | 0.013135 | CA3 |
| POLR3G | -0.88515 | 0.000549 | 0.013292 | CA3 |
| AP2A1 | 0.586666 | 0.000557 | 0.013425 | CA3 |
| MESP1 | 1.042937 | 0.000559 | 0.013467 | CA3 |
| CFTR | -1.3417 | 0.00057 | 0.013558 | CA3 |
| MADCAM1 | 0.962243 | 0.00058 | 0.013558 | CA3 |
| TGFB1 | 0.722486 | 0.00057 | 0.013558 | CA3 |
| FBXO2 | 0.735235 | 0.000576 | 0.013558 | CA3 |
| APOE | 0.851032 | 0.000575 | 0.013558 | CA3 |
| TUBG1 | 0.679666 | 0.000571 | 0.013558 | CA3 |
| EVA1B | 1.037662 | 0.000582 | 0.013558 | CA3 |
| WNK2 | 0.67289 | 0.000574 | 0.013558 | CA3 |
| TMEM88 | 1.546053 | 0.000576 | 0.013558 | CA3 |
| FOXD2 | 2.099119 | 0.000581 | 0.013558 | CA3 |
| PSMB10 | 0.744141 | 0.000582 | 0.013558 | CA3 |
| FNDC10 | 0.921089 | 0.000579 | 0.013558 | CA3 |
| HELLS | -0.67332 | 0.000597 | 0.01374 | CA3 |
| LSM4 | 0.765762 | 0.000598 | 0.013743 | CA3 |
| EIF1AX | -0.67317 | 0.000603 | 0.013835 | CA3 |
| IKZF2 | -0.79088 | 0.000606 | 0.013835 | CA3 |
| RPS24 | 0.581515 | 0.000605 | 0.013835 | CA3 |
| ZNF891 | -0.69729 | 0.000607 | 0.013835 | CA3 |
| NDUFA7 | 0.834347 | 0.000605 | 0.013835 | CA3 |
| SLC39A3 | 0.594583 | 0.00061 | 0.013898 | CA3 |
| PEPD | 0.625227 | 0.000619 | 0.013961 | CA3 |
| F12 | 1.425288 | 0.000618 | 0.013961 | CA3 |
| ETNK2 | 0.653265 | 0.000619 | 0.013961 | CA3 |
| CCDC138 | -0.62308 | 0.00062 | 0.013961 | CA3 |
| COX6A1 | 0.716456 | 0.000623 | 0.013982 | CA3 |
| SHTN1 | -0.79497 | 0.00063 | 0.014072 | CA3 |
| AL662899.2 | 2.825926 | 0.000632 | 0.014086 | CA3 |
| ZNF574 | 0.885965 | 0.000642 | 0.014236 | CA3 |
| COX6B1 | 0.907986 | 0.000642 | 0.014236 | CA3 |
| CYB5R3 | 0.674319 | 0.000647 | 0.014289 | CA3 |
| WFIKKN1 | 0.786933 | 0.000648 | 0.014289 | CA3 |
| ZNHIT2 | 0.673653 | 0.00065 | 0.014316 | CA3 |
| RBM41 | -0.62333 | 0.000654 | 0.01432 | CA3 |
| NUMBL | 0.646153 | 0.000652 | 0.01432 | CA3 |
| CCDC7 | -0.68285 | 0.000651 | 0.01432 | CA3 |
| IFITM2 | 1.078926 | 0.000655 | 0.014334 | CA3 |
| DCAF15 | 0.669716 | 0.000656 | 0.014338 | CA3 |

| | | | | |
|---|---|---|---|---|
| PLP2 | 0.871957 | 0.000671 | 0.014597 | CA3 |
| DNPH1 | 0.775737 | 0.000672 | 0.014609 | CA3 |
| TP53I13 | 0.581281 | 0.000675 | 0.014639 | CA3 |
| FGFR3 | 0.763632 | 0.000681 | 0.014733 | CA3 |
| ZNF638 | -0.6148 | 0.000681 | 0.014733 | CA3 |
| GSTP1 | 0.6405 | 0.000684 | 0.014744 | CA3 |
| SLC25A1 | 0.622809 | 0.000687 | 0.014767 | CA3 |
| NECTIN2 | 0.72825 | 0.000694 | 0.014779 | CA3 |
| NDUFS5 | 0.836039 | 0.000692 | 0.014779 | CA3 |
| EIF6 | 0.653724 | 0.000691 | 0.014779 | CA3 |
| FAM207A | 0.67124 | 0.000706 | 0.014954 | CA3 |
| GPX1 | 0.960549 | 0.000706 | 0.014954 | CA3 |
| UQCR11 | 0.612728 | 0.000711 | 0.015036 | CA3 |
| CAPN15 | 0.601965 | 0.000717 | 0.015115 | CA3 |
| FBXW9 | 0.617392 | 0.000725 | 0.015229 | CA3 |
| PEX14 | 0.581126 | 0.000729 | 0.015297 | CA3 |
| EPB41L1 | 0.599414 | 0.000731 | 0.015311 | CA3 |
| ATP13A2 | 0.695941 | 0.000732 | 0.015315 | CA3 |
| CHST1 | 0.772433 | 0.000734 | 0.015337 | CA3 |
| PEF1 | 0.598354 | 0.000739 | 0.015376 | CA3 |
| MELTF | 0.741222 | 0.000751 | 0.015574 | CA3 |
| ZNF765 | -0.61466 | 0.000753 | 0.015574 | CA3 |
| BICRA | 0.58754 | 0.000769 | 0.015751 | CA3 |
| EYA2 | 0.685553 | 0.00077 | 0.015751 | CA3 |
| NTN3 | 0.947045 | 0.000771 | 0.015751 | CA3 |
| SNRNP48 | -0.60053 | 0.000771 | 0.015751 | CA3 |
| ZNF141 | -0.69913 | 0.000783 | 0.015939 | CA3 |
| H4C2 | 1.194314 | 0.000786 | 0.015975 | CA3 |
| TGFBR3L | 1.4693 | 0.000787 | 0.015975 | CA3 |
| METTL7B | 0.84635 | 0.000795 | 0.016111 | CA3 |
| RPAIN | -0.60816 | 0.000801 | 0.016171 | CA3 |
| NEUROG2 | 0.978989 | 0.00081 | 0.016336 | CA3 |
| PGM3 | -0.65554 | 0.000821 | 0.016387 | CA3 |
| SDSL | 0.985169 | 0.000822 | 0.016387 | CA3 |
| ZDHHC12 | 0.905942 | 0.000818 | 0.016387 | CA3 |
| CCDC14 | -0.62166 | 0.00082 | 0.016387 | CA3 |
| MUC4 | 2.60666 | 0.00083 | 0.016459 | CA3 |
| CHCHD6 | 0.845815 | 0.000839 | 0.01651 | CA3 |
| COPRS | 0.749874 | 0.000843 | 0.016557 | CA3 |
| NASP | -0.67125 | 0.000867 | 0.016735 | CA3 |
| RPLP1 | 0.60059 | 0.00087 | 0.016735 | CA3 |
| DMTN | 0.754629 | 0.000868 | 0.016735 | CA3 |
| SPINDOC | 0.594154 | 0.00086 | 0.016735 | CA3 |
| RNF187 | 0.66701 | 0.00087 | 0.016735 | CA3 |

| | | | | |
|---|---|---|---|---|
| MRPL53 | 0.666393 | 0.000874 | 0.01678 | CA3 |
| TIA1 | -0.58008 | 0.000877 | 0.016804 | CA3 |
| FOXN2 | -0.73838 | 0.000887 | 0.016946 | CA3 |
| NRROS | 0.639785 | 0.000894 | 0.017062 | CA3 |
| SLC5A5 | 2.709531 | 0.000897 | 0.01709 | CA3 |
| RTL8C | 0.743133 | 0.000901 | 0.017131 | CA3 |
| GAMT | 0.603097 | 0.00094 | 0.017711 | CA3 |
| RXRG | 0.648784 | 0.000953 | 0.017894 | CA3 |
| EFNB1 | 0.597744 | 0.000965 | 0.018028 | CA3 |
| ACTC1 | 2.032041 | 0.000966 | 0.018028 | CA3 |
| GAL3ST3 | 0.841514 | 0.000968 | 0.018034 | CA3 |
| TRPM4 | 0.727062 | 0.000974 | 0.018054 | CA3 |
| C15orf41 | -0.68747 | 0.000973 | 0.018054 | CA3 |
| MAP3K10 | 0.757501 | 0.000975 | 0.018058 | CA3 |
| CCN4 | 1.707198 | 0.000977 | 0.01806 | CA3 |
| CFL2 | -0.67083 | 0.000979 | 0.01806 | CA3 |
| PTRHD1 | 0.629799 | 0.000978 | 0.01806 | CA3 |
| SLC35B3 | -0.68577 | 0.000985 | 0.018124 | CA3 |
| CCDC106 | 0.681241 | 0.000988 | 0.018153 | CA3 |
| MPG | 0.633367 | 0.000997 | 0.01828 | CA3 |
| RPL13A | 0.627556 | 0.001001 | 0.018302 | CA3 |
| TPM2 | 1.006082 | 0.001006 | 0.018357 | CA3 |
| CBARP | 0.79386 | 0.001015 | 0.018461 | CA3 |
| SNTA1 | 0.61943 | 0.001026 | 0.01847 | CA3 |
| PRSS23 | 0.885948 | 0.001024 | 0.01847 | CA3 |
| PWWP2B | 0.625605 | 0.001018 | 0.01847 | CA3 |
| ELMOD2 | -0.62802 | 0.001021 | 0.01847 | CA3 |
| CLDND1 | -0.87081 | 0.00103 | 0.018471 | CA3 |
| SEPTIN7 | -0.72951 | 0.001033 | 0.018471 | CA3 |
| RPL38 | 0.589903 | 0.001031 | 0.018471 | CA3 |
| FKRP | 1.120025 | 0.001032 | 0.018471 | CA3 |
| ZNF138 | -0.69084 | 0.001034 | 0.018475 | CA3 |
| COX5A | 0.615482 | 0.001038 | 0.01853 | CA3 |
| ZGRF1 | -0.80426 | 0.001042 | 0.018561 | CA3 |
| FRYL | -0.75964 | 0.001047 | 0.01862 | CA3 |
| GPR68 | 1.111551 | 0.001059 | 0.018699 | CA3 |
| TOMM7 | 0.742112 | 0.001058 | 0.018699 | CA3 |
| AC004997.1 | 0.946358 | 0.001061 | 0.018712 | CA3 |
| PALM | 0.629272 | 0.001065 | 0.018754 | CA3 |
| FMO5 | -0.99425 | 0.001066 | 0.018754 | CA3 |
| C16orf89 | 0.785935 | 0.001093 | 0.019089 | CA3 |
| GET3 | 0.616252 | 0.001097 | 0.019097 | CA3 |
| PDXP | 1.272455 | 0.0011 | 0.019128 | CA3 |
| GJB5 | 1.599637 | 0.00111 | 0.019267 | CA3 |

| | | | | |
|---|---|---|---|---|
| SLFN5 | -0.59179 | 0.001117 | 0.019366 | CA3 |
| GIT1 | 0.599792 | 0.001119 | 0.01938 | CA3 |
| PNPT1 | -0.67591 | 0.001122 | 0.019393 | CA3 |
| ZNF503 | 0.709398 | 0.001122 | 0.019393 | CA3 |
| GPR82 | -1.9855 | 0.001125 | 0.01942 | CA3 |
| CISD3 | 0.602342 | 0.001131 | 0.019487 | CA3 |
| TIMM17B | 0.597869 | 0.001135 | 0.019493 | CA3 |
| SCRG1 | 0.641185 | 0.001153 | 0.019663 | CA3 |
| CCDC177 | 0.81373 | 0.001157 | 0.019712 | CA3 |
| TTYH3 | 0.656359 | 0.00116 | 0.019734 | CA3 |
| LGALS1 | 0.731608 | 0.001165 | 0.019751 | CA3 |
| ZNF326 | -0.626 | 0.001171 | 0.019785 | CA3 |
| FOXP2 | -0.7632 | 0.001188 | 0.0199 | CA3 |
| ACOT1 | 1.006551 | 0.001188 | 0.0199 | CA3 |
| RPUSD1 | 0.608999 | 0.001192 | 0.019913 | CA3 |
| NYAP1 | 0.645567 | 0.001193 | 0.019918 | CA3 |
| SLC8A2 | 1.046512 | 0.001212 | 0.020106 | CA3 |
| H1-10 | 0.60979 | 0.001214 | 0.020114 | CA3 |
| NR1D1 | 0.812275 | 0.001219 | 0.020162 | CA3 |
| CITED2 | 0.60309 | 0.001224 | 0.020162 | CA3 |
| ATPSCKMT | -0.63229 | 0.001229 | 0.020189 | CA3 |
| SNX16 | -0.60392 | 0.001243 | 0.020307 | CA3 |
| IGSF9B | 0.807422 | 0.001252 | 0.020316 | CA3 |
| GLI3 | 0.626718 | 0.001246 | 0.020316 | CA3 |
| CDH22 | 0.729965 | 0.001251 | 0.020316 | CA3 |
| LDOC1 | 0.777897 | 0.001248 | 0.020316 | CA3 |
| LINC00634 | 0.885653 | 0.00125 | 0.020316 | CA3 |
| FSTL4 | 1.151247 | 0.001264 | 0.020417 | CA3 |
| KRT19 | 2.09129 | 0.001263 | 0.020417 | CA3 |
| ZNF781 | -0.72923 | 0.001266 | 0.020417 | CA3 |
| SLC26A2 | -0.75743 | 0.001291 | 0.020645 | CA3 |
| ADORA1 | 0.711623 | 0.001289 | 0.020645 | CA3 |
| ELAPOR2 | -0.60474 | 0.001291 | 0.020645 | CA3 |
| PRDM16 | 0.808115 | 0.001299 | 0.020729 | CA3 |
| PLN | 3.007525 | 0.001304 | 0.020768 | CA3 |
| CLIP3 | 0.618175 | 0.00131 | 0.020795 | CA3 |
| SARDH | 0.795338 | 0.001308 | 0.020795 | CA3 |
| ARAP2 | -0.7314 | 0.001316 | 0.020851 | CA3 |
| PLD3 | 0.780492 | 0.001321 | 0.020886 | CA3 |
| CD320 | 0.722283 | 0.001339 | 0.021089 | CA3 |
| CCNE2 | -1.09187 | 0.00134 | 0.021089 | CA3 |
| LRFN1 | 0.794224 | 0.001344 | 0.021095 | CA3 |
| EZHIP | 1.071251 | 0.001343 | 0.021095 | CA3 |
| IL11 | 2.505599 | 0.001379 | 0.021422 | CA3 |

| | | | | |
|---|---|---|---|---|
| C1GALT1 | -0.81908 | 0.001405 | 0.021511 | CA3 |
| LHX2 | 0.691997 | 0.001407 | 0.021511 | CA3 |
| C1QL2 | 1.3529 | 0.001397 | 0.021511 | CA3 |
| COL1A1 | 1.076013 | 0.001426 | 0.021675 | CA3 |
| TMEM240 | 0.693861 | 0.001426 | 0.021675 | CA3 |
| ATP5F1D | 0.624169 | 0.001432 | 0.021699 | CA3 |
| FZD2 | 1.013129 | 0.001435 | 0.021705 | CA3 |
| TRPM6 | -0.868 | 0.001441 | 0.021776 | CA3 |
| ARHGAP5 | -0.98486 | 0.001481 | 0.022087 | CA3 |
| C4orf36 | -0.68579 | 0.00148 | 0.022087 | CA3 |
| TKT | 0.593794 | 0.00148 | 0.022087 | CA3 |
| CEP83 | -0.64026 | 0.001475 | 0.022087 | CA3 |
| REX1BD | 0.587305 | 0.001485 | 0.022107 | CA3 |
| PDLIM7 | 0.623729 | 0.001487 | 0.02212 | CA3 |
| MEIS3 | 1.029841 | 0.001506 | 0.022241 | CA3 |
| PIGZ | 0.687035 | 0.001505 | 0.022241 | CA3 |
| TSPAN33 | 0.58924 | 0.001503 | 0.022241 | CA3 |
| PABPC3 | 0.655239 | 0.001516 | 0.022361 | CA3 |
| CSRP1 | 0.657601 | 0.001521 | 0.022393 | CA3 |
| RSC1A1 | -2.41288 | 0.001524 | 0.022425 | CA3 |
| AGTPBP1 | -0.76034 | 0.001527 | 0.022441 | CA3 |
| LUC7L3 | -0.59363 | 0.001536 | 0.022517 | CA3 |
| WASHC4 | -0.5893 | 0.001541 | 0.022567 | CA3 |
| EPHB3 | 0.621959 | 0.001552 | 0.022651 | CA3 |
| H1-5 | -2.06543 | 0.001553 | 0.022651 | CA3 |
| ZNF579 | 0.646455 | 0.001551 | 0.022651 | CA3 |
| KRT8 | 1.536311 | 0.001562 | 0.022727 | CA3 |
| CITED4 | 0.779616 | 0.001575 | 0.022836 | CA3 |
| ESCO2 | -1.51755 | 0.001588 | 0.022959 | CA3 |
| BEGAIN | 0.725784 | 0.001595 | 0.02299 | CA3 |
| MSANTD2 | -0.6164 | 0.001598 | 0.023016 | CA3 |
| EGR4 | 1.326644 | 0.001601 | 0.023016 | CA3 |
| RPLP2 | 0.593946 | 0.001604 | 0.023016 | CA3 |
| RAD51AP1 | -1.16704 | 0.001606 | 0.023027 | CA3 |
| JPH2 | 1.418926 | 0.001616 | 0.023073 | CA3 |
| RBPMS2 | 1.017738 | 0.001614 | 0.023073 | CA3 |
| ECRG4 | 0.875507 | 0.001628 | 0.023194 | CA3 |
| TRMT13 | -0.74917 | 0.001629 | 0.023194 | CA3 |
| PIBF1 | -0.6321 | 0.001649 | 0.023369 | CA3 |
| FABP6 | 1.001696 | 0.001654 | 0.0234 | CA3 |
| FAM133B | -0.62967 | 0.001657 | 0.023433 | CA3 |
| PBXIP1 | 0.612935 | 0.001661 | 0.023462 | CA3 |
| C1QL1 | 0.862166 | 0.00167 | 0.023547 | CA3 |
| ARHGAP39 | 0.581306 | 0.001702 | 0.023815 | CA3 |

| | | | | |
|---|---|---|---|---|
| ADGRB2 | 0.663651 | 0.001712 | 0.023853 | CA3 |
| TRAM1L1 | -0.69851 | 0.001711 | 0.023853 | CA3 |
| ETHE1 | 0.714812 | 0.001714 | 0.023859 | CA3 |
| ARX | 0.766122 | 0.001721 | 0.023877 | CA3 |
| CHEK2 | -0.71545 | 0.001725 | 0.02388 | CA3 |
| GPR20 | 2.082717 | 0.001726 | 0.02388 | CA3 |
| AP001458.2 | 1.33973 | 0.001748 | 0.024087 | CA3 |
| SUSD2 | 1.115456 | 0.001772 | 0.024328 | CA3 |
| LYRM7 | -0.60227 | 0.001769 | 0.024328 | CA3 |
| TLR2 | -1.05837 | 0.001777 | 0.024387 | CA3 |
| SLC43A2 | 0.58114 | 0.001781 | 0.024396 | CA3 |
| FANCM | -0.64629 | 0.00178 | 0.024396 | CA3 |
| EMX2 | 0.73513 | 0.001786 | 0.024418 | CA3 |
| TESPA1 | -2.36139 | 0.001793 | 0.024481 | CA3 |
| DLGAP4 | 0.664241 | 0.001799 | 0.024523 | CA3 |
| HLA-DRB1 | 1.479243 | 0.001799 | 0.024523 | CA3 |
| TGFB1I1 | 0.975153 | 0.001817 | 0.024634 | CA3 |
| EFR3B | 0.649639 | 0.00182 | 0.024639 | CA3 |
| BAIAP2 | 0.624678 | 0.001819 | 0.024639 | CA3 |
| FRK | -0.7581 | 0.001837 | 0.024778 | CA3 |
| C1orf35 | 0.591006 | 0.001835 | 0.024778 | CA3 |
| TUBB | 0.633316 | 0.001838 | 0.024778 | CA3 |
| MLKL | -0.7524 | 0.00185 | 0.024895 | CA3 |
| LRP5 | 0.715138 | 0.001856 | 0.02496 | CA3 |
| CCDC88A | -0.6472 | 0.001862 | 0.024969 | CA3 |
| DYNC1LI2 | -0.71648 | 0.001858 | 0.024969 | CA3 |
| MRPL58 | 0.622201 | 0.001863 | 0.024969 | CA3 |
| SMKR1 | 1.247556 | 0.001864 | 0.024969 | CA3 |
| NOC4L | 0.59808 | 0.001871 | 0.025018 | CA3 |
| GP1BB | 1.184976 | 0.001877 | 0.025059 | CA3 |
| BLOC1S1 | 0.608245 | 0.001896 | 0.025208 | CA3 |
| H4C12 | 1.140043 | 0.001894 | 0.025208 | CA3 |
| LRRN4CL | 1.293595 | 0.001906 | 0.025278 | CA3 |
| SNRPN | 0.630504 | 0.001918 | 0.025324 | CA3 |
| MIS18BP1 | -0.69564 | 0.001915 | 0.025324 | CA3 |
| ZNF397 | -0.62996 | 0.001932 | 0.025462 | CA3 |
| SGK1 | -0.74285 | 0.001946 | 0.025567 | CA3 |
| FAM241A | -0.72468 | 0.001958 | 0.025675 | CA3 |
| FAM117A | 0.699341 | 0.001963 | 0.025695 | CA3 |
| ZNF696 | -1.04312 | 0.001977 | 0.025774 | CA3 |
| TTR | 2.547303 | 0.00199 | 0.025871 | CA3 |
| PTGIS | 1.244887 | 0.001992 | 0.025871 | CA3 |
| PHF11 | -0.63652 | 0.00199 | 0.025871 | CA3 |
| GPR6 | 2.382648 | 0.002018 | 0.026152 | CA3 |

| | | | | |
|---|---|---|---|---|
| FZD8 | 0.868657 | 0.00203 | 0.026232 | CA3 |
| PPP1R35 | 0.623142 | 0.002035 | 0.026256 | CA3 |
| GPC4 | 0.649433 | 0.002045 | 0.026299 | CA3 |
| HSPB1 | 1.184763 | 0.002049 | 0.026299 | CA3 |
| TMEM198 | 0.739413 | 0.002042 | 0.026299 | CA3 |
| ZFP62 | -0.64096 | 0.002049 | 0.026299 | CA3 |
| GLIS1 | 0.932464 | 0.002053 | 0.026324 | CA3 |
| WSB1 | -0.61079 | 0.002085 | 0.026532 | CA3 |
| RWDD1 | -0.58038 | 0.002085 | 0.026532 | CA3 |
| PDF | 0.624156 | 0.002085 | 0.026532 | CA3 |
| FIGNL2 | 1.277926 | 0.002097 | 0.026597 | CA3 |
| HSPB7 | 1.097554 | 0.002115 | 0.026736 | CA3 |
| PPP4R2 | -0.65499 | 0.002138 | 0.026818 | CA3 |
| PCDH9 | -0.6812 | 0.00214 | 0.026818 | CA3 |
| MRPL24 | 0.640532 | 0.002147 | 0.026851 | CA3 |
| LRRC1 | -0.79304 | 0.002167 | 0.026991 | CA3 |
| SLC52A2 | 0.583083 | 0.002183 | 0.027135 | CA3 |
| LAGE3 | 0.746197 | 0.0022 | 0.027218 | CA3 |
| ZNF540 | -0.66503 | 0.002205 | 0.027238 | CA3 |
| LRRCC1 | -0.59019 | 0.002208 | 0.027255 | CA3 |
| GRN | 0.685706 | 0.002222 | 0.027298 | CA3 |
| PTCH2 | 0.612584 | 0.002223 | 0.027298 | CA3 |
| CENPE | -1.08027 | 0.002213 | 0.027298 | CA3 |
| SLC16A11 | 0.811995 | 0.002215 | 0.027298 | CA3 |
| BRCA1 | -0.73035 | 0.002241 | 0.027437 | CA3 |
| SAMD10 | 0.79785 | 0.002263 | 0.027556 | CA3 |
| NOTCH1 | 0.602068 | 0.002265 | 0.027556 | CA3 |
| UQCC3 | 0.642005 | 0.002266 | 0.027556 | CA3 |
| SS18L2 | -0.6153 | 0.002273 | 0.027581 | CA3 |
| SURF2 | 0.632954 | 0.002277 | 0.027589 | CA3 |
| DIPK1B | 0.617114 | 0.002276 | 0.027589 | CA3 |
| LMTK3 | 0.774214 | 0.002293 | 0.027724 | CA3 |
| ZNF514 | -0.59129 | 0.002292 | 0.027724 | CA3 |
| ARHGAP21 | -0.62481 | 0.002302 | 0.027786 | CA3 |
| STK26 | -1.21775 | 0.002312 | 0.027883 | CA3 |
| LRRIQ3 | -1.34476 | 0.002313 | 0.027883 | CA3 |
| C1orf112 | -0.64862 | 0.002317 | 0.027883 | CA3 |
| MARCHF1 | -0.58253 | 0.002329 | 0.027995 | CA3 |
| TUBB4B | 0.628888 | 0.00234 | 0.028053 | CA3 |
| ADAM28 | -0.89978 | 0.002353 | 0.028108 | CA3 |
| NR4A2 | -0.82697 | 0.002355 | 0.028108 | CA3 |
| UBB | 0.65059 | 0.002388 | 0.028217 | CA3 |
| OTOF | 1.904602 | 0.002436 | 0.028537 | CA3 |
| SPTLC2 | -0.69139 | 0.002453 | 0.028675 | CA3 |

| | | | | |
|---|---|---|---|---|
| PPARA | -0.69969 | 0.002453 | 0.028675 | CA3 |
| NINJ1 | 0.611618 | 0.002466 | 0.028757 | CA3 |
| TCAP | 0.692371 | 0.002468 | 0.028769 | CA3 |
| ACTG2 | 3.325987 | 0.002471 | 0.028782 | CA3 |
| SFR1 | -0.80643 | 0.002481 | 0.028856 | CA3 |
| MGAT5B | 0.811394 | 0.002481 | 0.028856 | CA3 |
| PCDHGA4 | 0.685096 | 0.002488 | 0.028909 | CA3 |
| ICE2 | -0.58065 | 0.002501 | 0.029009 | CA3 |
| FZD9 | 0.864405 | 0.002509 | 0.029024 | CA3 |
| ZBTB45 | 0.603619 | 0.002516 | 0.029058 | CA3 |
| PCDH1 | 0.626482 | 0.00252 | 0.029082 | CA3 |
| PLD1 | -0.65087 | 0.002526 | 0.029122 | CA3 |
| IGFBP6 | 1.098846 | 0.00254 | 0.029125 | CA3 |
| MAP3K15 | 2.394102 | 0.002539 | 0.029125 | CA3 |
| DRD4 | 0.906408 | 0.002545 | 0.029157 | CA3 |
| DNLZ | 0.837386 | 0.002575 | 0.029359 | CA3 |
| KCNG1 | 0.684342 | 0.002579 | 0.029388 | CA3 |
| TIMP1 | 0.932878 | 0.002591 | 0.029401 | CA3 |
| REEP3 | -0.81394 | 0.002588 | 0.029401 | CA3 |
| MRPL12 | 0.673842 | 0.002592 | 0.029401 | CA3 |
| DLG1 | -0.58028 | 0.002596 | 0.029412 | CA3 |
| QPRT | 0.674388 | 0.00262 | 0.029622 | CA3 |
| IFI27L2 | 0.593533 | 0.00263 | 0.029691 | CA3 |
| TLR5 | -0.85039 | 0.002639 | 0.029773 | CA3 |
| ST8SIA4 | -0.74283 | 0.002661 | 0.029885 | CA3 |
| ANKRD36 | -0.62559 | 0.002679 | 0.029973 | CA3 |
| FOXC2 | 1.357115 | 0.002705 | 0.030148 | CA3 |
| MEIS1 | -0.82728 | 0.002712 | 0.03019 | CA3 |
| CD177 | 1.661805 | 0.002731 | 0.030295 | CA3 |
| ENPP4 | -0.70425 | 0.002757 | 0.030458 | CA3 |
| PAIP2B | -0.97486 | 0.002762 | 0.030469 | CA3 |
| HINT1 | 0.639523 | 0.002795 | 0.030638 | CA3 |
| ALDOA | 0.618786 | 0.002798 | 0.030658 | CA3 |
| EPHA8 | 1.466082 | 0.00282 | 0.03079 | CA3 |
| CRMP1 | 0.663246 | 0.002822 | 0.03079 | CA3 |
| KCNJ14 | 0.626288 | 0.002841 | 0.030913 | CA3 |
| C4B | 1.052424 | 0.002843 | 0.030924 | CA3 |
| SASS6 | -0.69709 | 0.002852 | 0.030974 | CA3 |
| PIK3R2 | 0.757762 | 0.00288 | 0.031095 | CA3 |
| PLPPR3 | 0.954993 | 0.00288 | 0.031095 | CA3 |
| SACS | -1.17797 | 0.002873 | 0.031095 | CA3 |
| RNASEK | 0.884787 | 0.002876 | 0.031095 | CA3 |
| PLPP3 | 0.638064 | 0.002901 | 0.031279 | CA3 |
| BLVRB | 0.66312 | 0.002948 | 0.031489 | CA3 |

| | | | | |
|---|---|---|---|---|
| RANBP17 | -0.88704 | 0.002949 | 0.031489 | CA3 |
| FTL | 0.605826 | 0.002959 | 0.03153 | CA3 |
| PARD6A | 0.622321 | 0.002964 | 0.031561 | CA3 |
| ERI2 | -0.61676 | 0.002967 | 0.031575 | CA3 |
| AQP7 | 1.629445 | 0.002975 | 0.031632 | CA3 |
| BGLAP | 0.937949 | 0.00299 | 0.031749 | CA3 |
| TRMT10A | -0.73876 | 0.003012 | 0.031845 | CA3 |
| AVPI1 | 0.643903 | 0.003018 | 0.031866 | CA3 |
| IL18 | -0.82926 | 0.003024 | 0.031882 | CA3 |
| ACKR3 | 0.711126 | 0.003043 | 0.032022 | CA3 |
| H2AC20 | 0.595879 | 0.003068 | 0.032164 | CA3 |
| SLC7A10 | 0.850651 | 0.003073 | 0.032176 | CA3 |
| H4C5 | 0.900324 | 0.003084 | 0.032241 | CA3 |
| RNF152 | -0.79769 | 0.003092 | 0.032287 | CA3 |
| CRIP1 | 1.295108 | 0.003092 | 0.032287 | CA3 |
| PSTPIP2 | -0.90383 | 0.0031 | 0.032314 | CA3 |
| RFLNA | 0.718585 | 0.00311 | 0.032326 | CA3 |
| NGB | 1.17596 | 0.003134 | 0.03247 | CA3 |
| DBN1 | 0.631332 | 0.003151 | 0.03251 | CA3 |
| CLU | 0.774084 | 0.003147 | 0.03251 | CA3 |
| IQGAP3 | -2.10271 | 0.003178 | 0.032604 | CA3 |
| AC069368.1 | 1.14552 | 0.003231 | 0.032999 | CA3 |
| BHLHA15 | 1.023578 | 0.003244 | 0.033081 | CA3 |
| TRIL | 0.743698 | 0.003262 | 0.033169 | CA3 |
| PCDHGB4 | 1.179655 | 0.003294 | 0.033451 | CA3 |
| ADRB1 | 0.733853 | 0.003303 | 0.033512 | CA3 |
| ISG15 | 1.057575 | 0.003304 | 0.033512 | CA3 |
| LACC1 | -0.8271 | 0.003347 | 0.033793 | CA3 |
| H2AC21 | 0.705766 | 0.003351 | 0.033801 | CA3 |
| KLHL4 | -0.77948 | 0.003356 | 0.033812 | CA3 |
| GCA | -0.71075 | 0.003358 | 0.033812 | CA3 |
| HCN4 | 0.901694 | 0.003373 | 0.03387 | CA3 |
| VWCE | 0.618864 | 0.003367 | 0.03387 | CA3 |
| ALDOC | 0.674913 | 0.003394 | 0.03406 | CA3 |
| FBXO44 | 0.74624 | 0.003416 | 0.034182 | CA3 |
| FABP7 | 0.587134 | 0.00343 | 0.034275 | CA3 |
| C3orf33 | -0.71527 | 0.003434 | 0.034275 | CA3 |
| ZNF253 | -0.90804 | 0.003431 | 0.034275 | CA3 |
| HCST | 1.389887 | 0.003437 | 0.034279 | CA3 |
| ATP6V0D1 | 0.653984 | 0.003443 | 0.034301 | CA3 |
| BCAN | 0.62839 | 0.003462 | 0.034444 | CA3 |
| CRTC1 | 0.691558 | 0.003514 | 0.034739 | CA3 |
| LINGO1 | 0.932356 | 0.003511 | 0.034739 | CA3 |
| AGMO | -1.08112 | 0.003508 | 0.034739 | CA3 |

| | | | | |
|---|---|---|---|---|
| BFSP1 | 0.918982 | 0.00353 | 0.034804 | CA3 |
| STEAP4 | -1.4053 | 0.003545 | 0.034892 | CA3 |
| GCHFR | 0.97063 | 0.003559 | 0.034906 | CA3 |
| CBFB | -0.59003 | 0.003567 | 0.034936 | CA3 |
| PTN | 0.586153 | 0.003572 | 0.034961 | CA3 |
| SPATA2L | 0.691304 | 0.003577 | 0.034975 | CA3 |
| CTAGE6 | -1.50362 | 0.003586 | 0.034995 | CA3 |
| TMEM147 | 0.644704 | 0.00362 | 0.035185 | CA3 |
| VPS26A | -0.62132 | 0.00362 | 0.035185 | CA3 |
| SH2B2 | 0.642804 | 0.003621 | 0.035185 | CA3 |
| PTP4A2 | -0.6983 | 0.003613 | 0.035185 | CA3 |
| P2RX2 | 1.767215 | 0.003626 | 0.035192 | CA3 |
| FRMD4B | -0.73283 | 0.003638 | 0.035237 | CA3 |
| FJX1 | 0.681932 | 0.003643 | 0.035237 | CA3 |
| TMEM200B | 0.868069 | 0.003642 | 0.035237 | CA3 |
| BTBD2 | 0.655818 | 0.003658 | 0.03524 | CA3 |
| FAM174C | 0.631008 | 0.003657 | 0.03524 | CA3 |
| TNC | 0.708509 | 0.003676 | 0.035337 | CA3 |
| CLDN10 | 0.698053 | 0.003677 | 0.035337 | CA3 |
| ZNF469 | 0.67741 | 0.003677 | 0.035337 | CA3 |
| UBE2V1 | -0.59717 | 0.003698 | 0.035513 | CA3 |
| ATP1A2 | 0.69443 | 0.003715 | 0.035635 | CA3 |
| RPS15 | 0.627543 | 0.003734 | 0.035795 | CA3 |
| HRH3 | 1.189629 | 0.003776 | 0.036028 | CA3 |
| HRC | 1.291702 | 0.003787 | 0.036087 | CA3 |
| MRPL30 | -0.7097 | 0.003803 | 0.03618 | CA3 |
| EPHB6 | 0.659596 | 0.003808 | 0.036188 | CA3 |
| ZFYVE16 | -0.70524 | 0.003853 | 0.036464 | CA3 |
| NFATC1 | 0.596248 | 0.003879 | 0.036649 | CA3 |
| RNF4 | -0.59534 | 0.003882 | 0.036658 | CA3 |
| FEZF2 | 0.830957 | 0.003888 | 0.036668 | CA3 |
| GLMN | -0.61325 | 0.003888 | 0.036668 | CA3 |
| ADGRA2 | 0.618379 | 0.003905 | 0.036772 | CA3 |
| ACER3 | -0.78536 | 0.00394 | 0.036955 | CA3 |
| KCNJ4 | 1.058894 | 0.003956 | 0.037042 | CA3 |
| LCN12 | 0.894396 | 0.003972 | 0.037091 | CA3 |
| ATP6V0C | 0.71944 | 0.004044 | 0.037485 | CA3 |
| RASSF7 | 0.716224 | 0.004077 | 0.037685 | CA3 |
| PYCARD | 1.005793 | 0.004093 | 0.037793 | CA3 |
| SLC29A4 | 0.743874 | 0.004119 | 0.037926 | CA3 |
| S100A14 | 1.683948 | 0.004122 | 0.037926 | CA3 |
| DDTL | 0.641966 | 0.004134 | 0.03795 | CA3 |
| DGCR6L | 0.594845 | 0.004215 | 0.038498 | CA3 |
| DLL3 | 0.684152 | 0.004236 | 0.038521 | CA3 |

| | | | | |
|---|---|---|---|---|
| RASD2 | 0.755654 | 0.004228 | 0.038521 | CA3 |
| SNAPC2 | 0.635297 | 0.004229 | 0.038521 | CA3 |
| CFL1 | 0.617653 | 0.004223 | 0.038521 | CA3 |
| PDYN | 1.340428 | 0.004258 | 0.038538 | CA3 |
| KIF20B | -0.5985 | 0.004255 | 0.038538 | CA3 |
| GABRD | 1.089465 | 0.004259 | 0.038538 | CA3 |
| ASPA | -0.8838 | 0.004272 | 0.03856 | CA3 |
| DUSP8 | 0.696617 | 0.004294 | 0.038643 | CA3 |
| SRXN1 | 0.589645 | 0.00429 | 0.038643 | CA3 |
| ANLN | -0.96929 | 0.004328 | 0.038809 | CA3 |
| FBLN5 | 0.654597 | 0.004328 | 0.038809 | CA3 |
| ST18 | -0.85909 | 0.00433 | 0.038809 | CA3 |
| RAMACL | -0.66334 | 0.004339 | 0.038848 | CA3 |
| PTPRC | -0.6639 | 0.004367 | 0.03898 | CA3 |
| ANKRD9 | 0.652893 | 0.004433 | 0.039476 | CA3 |
| RAB3IP | -0.58702 | 0.004491 | 0.039802 | CA3 |
| H2BC12 | 0.83485 | 0.004491 | 0.039802 | CA3 |
| ITGB4 | 0.671758 | 0.004538 | 0.04006 | CA3 |
| SIX5 | 0.710934 | 0.004551 | 0.040122 | CA3 |
| FBXL16 | 0.863581 | 0.004565 | 0.040146 | CA3 |
| LRRC3 | 0.764037 | 0.004576 | 0.04016 | CA3 |
| ACTB | 0.60152 | 0.004599 | 0.040198 | CA3 |
| PRR36 | 0.863085 | 0.004593 | 0.040198 | CA3 |
| CPNE6 | 0.74392 | 0.004651 | 0.0405 | CA3 |
| INSM1 | 0.838706 | 0.004654 | 0.040505 | CA3 |
| PSMB8 | 0.580976 | 0.004678 | 0.040586 | CA3 |
| ATP6V0E1 | 0.629774 | 0.004701 | 0.04068 | CA3 |
| TINAGL1 | 0.863785 | 0.004701 | 0.04068 | CA3 |
| TMEM144 | -0.99652 | 0.004706 | 0.04068 | CA3 |
| TLR9 | 1.008629 | 0.004704 | 0.04068 | CA3 |
| C15orf39 | 0.605537 | 0.00477 | 0.041121 | CA3 |
| EHD2 | 0.660462 | 0.00479 | 0.041248 | CA3 |
| KCNC4 | 0.642115 | 0.004846 | 0.041578 | CA3 |
| PLCD1 | 0.60142 | 0.004842 | 0.041578 | CA3 |
| CCDC82 | -0.59497 | 0.004894 | 0.041821 | CA3 |
| MT1E | 0.877045 | 0.004897 | 0.041821 | CA3 |
| SLC22A6 | 1.680971 | 0.004891 | 0.041821 | CA3 |
| SAMD14 | 0.72165 | 0.004911 | 0.041872 | CA3 |
| MOSPD2 | -0.68656 | 0.004933 | 0.042034 | CA3 |
| FOSL1 | 1.543557 | 0.004951 | 0.04215 | CA3 |
| C19orf81 | 1.026005 | 0.005006 | 0.042507 | CA3 |
| PPP1R1B | 0.6105 | 0.005015 | 0.04256 | CA3 |
| PRDM2 | -0.60548 | 0.005033 | 0.042601 | CA3 |
| TOX2 | 0.681005 | 0.005032 | 0.042601 | CA3 |

| | | | | |
|---|---|---|---|---|
| PLGLB1 | -0.6974 | 0.005039 | 0.042624 | CA3 |
| TOP2A | -2.57639 | 0.005058 | 0.042718 | CA3 |
| SLC4A3 | 0.582771 | 0.005085 | 0.042864 | CA3 |
| WFIKKN2 | 1.309116 | 0.005094 | 0.04289 | CA3 |
| SPDL1 | -0.62995 | 0.005105 | 0.042938 | CA3 |
| LSR | 0.89583 | 0.00512 | 0.043046 | CA3 |
| ATAD5 | -0.69401 | 0.005123 | 0.04305 | CA3 |
| TMEM59L | 0.822312 | 0.005133 | 0.043095 | CA3 |
| TRIM47 | 0.627489 | 0.005142 | 0.043121 | CA3 |
| KCNJ11 | 0.690049 | 0.005153 | 0.043156 | CA3 |
| POLQ | -1.21065 | 0.005171 | 0.043203 | CA3 |
| FXYD5 | 0.694097 | 0.005172 | 0.043203 | CA3 |
| LAMB2 | 0.593809 | 0.005173 | 0.043203 | CA3 |
| MYOT | -0.74691 | 0.005198 | 0.043276 | CA3 |
| GRIK5 | 0.678373 | 0.005237 | 0.043331 | CA3 |
| TMEM168 | -0.58093 | 0.005237 | 0.043331 | CA3 |
| PRRT3 | 0.604907 | 0.005225 | 0.043331 | CA3 |
| CNTF | -0.67025 | 0.005228 | 0.043331 | CA3 |
| UNC5A | 0.791598 | 0.005276 | 0.043524 | CA3 |
| DNAJC25-GNG10 | -0.90782 | 0.005282 | 0.043524 | CA3 |
| PDZD4 | 0.608918 | 0.005328 | 0.043788 | CA3 |
| ARHGAP11A | -0.77023 | 0.00533 | 0.043788 | CA3 |
| ID1 | 0.665548 | 0.005342 | 0.043834 | CA3 |
| DOCK5 | -0.75888 | 0.005343 | 0.043834 | CA3 |
| CCDC122 | -0.93036 | 0.005395 | 0.044122 | CA3 |
| TRPV4 | 1.171141 | 0.005415 | 0.044177 | CA3 |
| MOBP | -1.0229 | 0.005419 | 0.044177 | CA3 |
| FMO3 | -1.37463 | 0.005444 | 0.044304 | CA3 |
| METRNL | 0.626308 | 0.005444 | 0.044304 | CA3 |
| FOXG1 | 0.649123 | 0.005454 | 0.04434 | CA3 |
| RTL1 | 1.843504 | 0.005462 | 0.044362 | CA3 |
| FGFR4 | 0.78652 | 0.005468 | 0.044385 | CA3 |
| MFRP | 2.340507 | 0.005495 | 0.04456 | CA3 |
| COX7A1 | 0.957577 | 0.005541 | 0.044776 | CA3 |
| CALY | 1.006966 | 0.005557 | 0.044816 | CA3 |
| TCIM | -0.99478 | 0.005606 | 0.045075 | CA3 |
| PHLDA2 | 2.331613 | 0.005621 | 0.045106 | CA3 |
| AKAP9 | -0.63285 | 0.00563 | 0.045117 | CA3 |
| MOG | -0.84893 | 0.005628 | 0.045117 | CA3 |
| ORAI1 | 0.808291 | 0.005638 | 0.045138 | CA3 |
| BUB1B | -1.91008 | 0.005652 | 0.045222 | CA3 |
| RPL39L | 0.926166 | 0.005654 | 0.045222 | CA3 |
| PTMS | 0.584492 | 0.005669 | 0.045316 | CA3 |
| SCUBE3 | 1.592652 | 0.005688 | 0.045353 | CA3 |

| | | | | |
|---|---|---|---|---|
| BTBD19 | 0.730293 | 0.005765 | 0.045812 | CA3 |
| IFI6 | 0.873904 | 0.005888 | 0.046441 | CA3 |
| AQP3 | 1.336648 | 0.005887 | 0.046441 | CA3 |
| ERMN | -0.90421 | 0.005917 | 0.046546 | CA3 |
| FOXO6 | 0.648734 | 0.005935 | 0.046666 | CA3 |
| NCAN | 0.710964 | 0.005985 | 0.046792 | CA3 |
| CENPF | -0.80688 | 0.006017 | 0.046918 | CA3 |
| ZNF467 | 0.78209 | 0.006029 | 0.046973 | CA3 |
| ANG | 0.87244 | 0.006051 | 0.04704 | CA3 |
| SEMA3A | 0.72576 | 0.00606 | 0.047078 | CA3 |
| CASKIN1 | 0.638366 | 0.006086 | 0.047231 | CA3 |
| CHRNA4 | 0.644408 | 0.006126 | 0.047389 | CA3 |
| HYAL1 | 0.791441 | 0.00613 | 0.047389 | CA3 |
| URGCP-MRPS24 | 2.875652 | 0.006193 | 0.047811 | CA3 |
| CEND1 | 0.71983 | 0.00622 | 0.047908 | CA3 |
| GIN1 | -0.61128 | 0.006227 | 0.047919 | CA3 |
| ZNF594 | -0.62342 | 0.006225 | 0.047919 | CA3 |
| ADGRA1 | 0.614287 | 0.006265 | 0.048129 | CA3 |
| MMP28 | 0.735617 | 0.006288 | 0.048228 | CA3 |
| ARF1 | 0.891543 | 0.006306 | 0.048275 | CA3 |
| IL1RAPL1 | -0.81511 | 0.006321 | 0.048275 | CA3 |
| ZNF566 | -0.60441 | 0.006304 | 0.048275 | CA3 |
| PDE1C | -0.81545 | 0.006367 | 0.048514 | CA3 |
| HTR6 | 1.214793 | 0.006365 | 0.048514 | CA3 |
| KCNF1 | 0.726783 | 0.006393 | 0.048614 | CA3 |
| NCDN | 0.785649 | 0.006408 | 0.048706 | CA3 |
| SETD9 | -0.80045 | 0.006414 | 0.048735 | CA3 |
| CHTF8 | -1.12535 | 0.006455 | 0.048773 | CA3 |
| PDLIM3 | 0.624878 | 0.00648 | 0.048776 | CA3 |
| ZDHHC22 | 0.699752 | 0.006484 | 0.048776 | CA3 |
| COL13A1 | 1.365037 | 0.006464 | 0.048776 | CA3 |
| STX1A | 0.75255 | 0.006511 | 0.048877 | CA3 |
| LRRC69 | -0.79217 | 0.006532 | 0.048917 | CA3 |
| CTNNA3 | -0.94595 | 0.006563 | 0.049059 | CA3 |
| ALDH4A1 | 0.654479 | 0.006589 | 0.049145 | CA3 |
| TSEN15 | -0.74757 | 0.006608 | 0.049195 | CA3 |
| PCDHGA3 | 0.662313 | 0.006646 | 0.049363 | CA3 |
| ASPM | -1.50873 | 0.006702 | 0.049462 | CA3 |
| CEMIP | 1.085059 | 0.006715 | 0.049462 | CA3 |
| CRHR2 | 1.584263 | 0.006729 | 0.049462 | CA3 |
| NPM3 | 0.7611 | 0.00669 | 0.049462 | CA3 |
| SYT12 | 0.65613 | 0.006689 | 0.049462 | CA3 |
| ADGRG3 | 1.569486 | 0.006705 | 0.049462 | CA3 |
| MRPL54 | 0.585712 | 0.006718 | 0.049462 | CA3 |

| | | | | |
|---|---|---|---|---|
| HES5 | 1.225576 | 0.0067 | 0.049462 | CA3 |
| POMZP3 | 0.58284 | 0.00674 | 0.049479 | CA3 |
| FANCB | -0.85716 | 0.006775 | 0.049659 | CA3 |
| CTSW | 2.460629 | 0.001728 | 0.02389 | CA3 |
| | | | | |
| AL157935.2 | 3.23526 | 1.89E-12 | 3.07E-08 | CA4 |
| NDUFA3 | 1.281289 | 1.39E-11 | 1.13E-07 | CA4 |
| COX8A | 1.70178 | 6.69E-11 | 3.62E-07 | CA4 |
| MDK | 1.911259 | 1.74E-10 | 5.64E-07 | CA4 |
| C4orf48 | 2.329086 | 1.67E-10 | 5.64E-07 | CA4 |
| RPL27 | 1.57355 | 3.93E-10 | 1.06E-06 | CA4 |
| EDF1 | 1.291281 | 6.07E-10 | 1.27E-06 | CA4 |
| CHPF | 1.296015 | 6.25E-10 | 1.27E-06 | CA4 |
| NUBP2 | 0.761975 | 1.12E-09 | 1.66E-06 | CA4 |
| MRPL24 | 1.053658 | 9.86E-10 | 1.66E-06 | CA4 |
| PNPLA2 | 1.046974 | 1.08E-09 | 1.66E-06 | CA4 |
| ZNF408 | 1.027571 | 1.39E-09 | 1.88E-06 | CA4 |
| RABAC1 | 1.354859 | 1.53E-09 | 1.91E-06 | CA4 |
| TMSB10 | 1.569299 | 1.81E-09 | 2.09E-06 | CA4 |
| AIP | 0.868859 | 2.30E-09 | 2.49E-06 | CA4 |
| UBE2L6 | 0.924053 | 3.15E-09 | 3.20E-06 | CA4 |
| ARL2 | 0.914289 | 3.41E-09 | 3.26E-06 | CA4 |
| ALYREF | 1.040838 | 5.03E-09 | 4.53E-06 | CA4 |
| URGCP-MRPS24 | 5.636752 | 5.70E-09 | 4.87E-06 | CA4 |
| POLR2I | 1.131703 | 6.32E-09 | 5.12E-06 | CA4 |
| POLR2L | 1.330966 | 7.15E-09 | 5.53E-06 | CA4 |
| TOMM7 | 1.122597 | 7.81E-09 | 5.76E-06 | CA4 |
| CSTB | 0.953044 | 8.28E-09 | 5.84E-06 | CA4 |
| ROMO1 | 1.429622 | 1.34E-08 | 8.37E-06 | CA4 |
| TMEM115 | 0.696785 | 1.33E-08 | 8.37E-06 | CA4 |
| CIAO2B | 1.233341 | 1.33E-08 | 8.37E-06 | CA4 |
| NAA38 | 1.200634 | 1.63E-08 | 9.78E-06 | CA4 |
| ODF2L | -1.06948 | 1.70E-08 | 9.86E-06 | CA4 |
| PCSK1N | 1.629123 | 1.76E-08 | 9.87E-06 | CA4 |
| FASTKD1 | -0.72839 | 1.83E-08 | 9.91E-06 | CA4 |
| ZNF574 | 0.820465 | 2.40E-08 | 1.22E-05 | CA4 |
| USP34 | -0.59045 | 2.65E-08 | 1.30E-05 | CA4 |
| MAN2B2 | 0.660836 | 3.12E-08 | 1.41E-05 | CA4 |
| FRA10AC1 | -0.71752 | 3.03E-08 | 1.41E-05 | CA4 |
| SCAND1 | 1.328282 | 3.10E-08 | 1.41E-05 | CA4 |
| DRAP1 | 0.96153 | 3.54E-08 | 1.55E-05 | CA4 |
| CCDC66 | -0.84032 | 3.65E-08 | 1.56E-05 | CA4 |
| DDX54 | 0.733998 | 4.41E-08 | 1.79E-05 | CA4 |
| CEP57L1 | -0.91298 | 4.35E-08 | 1.79E-05 | CA4 |

| | | | | |
|---|---|---|---|---|
| NDUFS7 | 0.779026 | 4.81E-08 | 1.90E-05 | CA4 |
| ZNF326 | -0.87934 | 5.09E-08 | 1.97E-05 | CA4 |
| ZSWIM4 | 0.834253 | 5.72E-08 | 2.16E-05 | CA4 |
| UBL5 | 1.270873 | 6.05E-08 | 2.23E-05 | CA4 |
| NINJ1 | 1.063974 | 6.70E-08 | 2.41E-05 | CA4 |
| PRAF2 | 1.075108 | 6.91E-08 | 2.44E-05 | CA4 |
| MICOS13 | 0.783508 | 7.97E-08 | 2.75E-05 | CA4 |
| GPX4 | 0.692442 | 8.53E-08 | 2.82E-05 | CA4 |
| LONP1 | 0.709314 | 8.45E-08 | 2.82E-05 | CA4 |
| DYNC2H1 | -0.60334 | 9.23E-08 | 3.00E-05 | CA4 |
| C12orf57 | 1.01726 | 1.01E-07 | 3.14E-05 | CA4 |
| RAMP1 | 1.225686 | 1.00E-07 | 3.14E-05 | CA4 |
| GLUD2 | 0.697712 | 1.06E-07 | 3.23E-05 | CA4 |
| MZT2B | 1.065208 | 1.08E-07 | 3.25E-05 | CA4 |
| PPP1R13L | 1.154602 | 1.11E-07 | 3.28E-05 | CA4 |
| RPS11 | 1.170824 | 1.21E-07 | 3.47E-05 | CA4 |
| ZNF503 | 0.976981 | 1.22E-07 | 3.47E-05 | CA4 |
| TIGD7 | -0.63102 | 1.26E-07 | 3.52E-05 | CA4 |
| PEF1 | 0.762745 | 1.32E-07 | 3.64E-05 | CA4 |
| RBM41 | -0.8817 | 1.39E-07 | 3.64E-05 | CA4 |
| NFYB | -0.8959 | 1.38E-07 | 3.64E-05 | CA4 |
| PLXNB2 | 0.675536 | 1.38E-07 | 3.64E-05 | CA4 |
| LDB1 | 0.801733 | 1.43E-07 | 3.68E-05 | CA4 |
| PUF60 | 0.605647 | 1.49E-07 | 3.78E-05 | CA4 |
| G2E3 | -0.83411 | 1.77E-07 | 4.41E-05 | CA4 |
| AC007325.2 | 1.342717 | 1.79E-07 | 4.41E-05 | CA4 |
| C19orf53 | 1.262837 | 1.84E-07 | 4.46E-05 | CA4 |
| CAPS2 | -0.67957 | 1.89E-07 | 4.51E-05 | CA4 |
| RPL37A | 0.968722 | 1.93E-07 | 4.54E-05 | CA4 |
| NUDC | 0.680696 | 2.12E-07 | 4.69E-05 | CA4 |
| XRN1 | -0.6326 | 2.08E-07 | 4.69E-05 | CA4 |
| TBC1D8B | -0.99132 | 2.04E-07 | 4.69E-05 | CA4 |
| PAMR1 | 0.893606 | 2.11E-07 | 4.69E-05 | CA4 |
| TNIP1 | 0.634966 | 2.24E-07 | 4.79E-05 | CA4 |
| RPS18 | 1.114673 | 2.24E-07 | 4.79E-05 | CA4 |
| RICTOR | -0.73838 | 2.36E-07 | 4.91E-05 | CA4 |
| SF3A2 | 0.69671 | 2.44E-07 | 5.00E-05 | CA4 |
| ORC2 | -0.6812 | 2.56E-07 | 5.02E-05 | CA4 |
| CYSTM1 | 0.748603 | 2.65E-07 | 5.02E-05 | CA4 |
| LRP1 | 0.770213 | 2.55E-07 | 5.02E-05 | CA4 |
| MRPS12 | 0.963192 | 2.64E-07 | 5.02E-05 | CA4 |
| TP53I13 | 0.953235 | 2.61E-07 | 5.02E-05 | CA4 |
| PLEC | 0.88368 | 2.66E-07 | 5.02E-05 | CA4 |
| FNIP1 | -0.68103 | 2.51E-07 | 5.02E-05 | CA4 |

| | | | | |
|---|---|---|---|---|
| NDUFA11 | 0.912636 | 2.94E-07 | 5.48E-05 | CA4 |
| CHCHD10 | 1.127785 | 3.04E-07 | 5.61E-05 | CA4 |
| PRDX5 | 1.101091 | 3.11E-07 | 5.68E-05 | CA4 |
| RPL41 | 1.693554 | 3.41E-07 | 6.15E-05 | CA4 |
| PWWP2A | -0.80339 | 3.56E-07 | 6.31E-05 | CA4 |
| NDUFAF3 | 0.830523 | 3.58E-07 | 6.31E-05 | CA4 |
| BAX | 0.77217 | 3.94E-07 | 6.87E-05 | CA4 |
| PSMA7 | 0.856016 | 4.01E-07 | 6.92E-05 | CA4 |
| DYNLRB1 | 1.244669 | 4.10E-07 | 7.01E-05 | CA4 |
| PTPN4 | -0.76797 | 4.27E-07 | 7.14E-05 | CA4 |
| H2AX | 0.942274 | 4.26E-07 | 7.14E-05 | CA4 |
| ARID4B | -0.73464 | 4.32E-07 | 7.15E-05 | CA4 |
| AP1B1 | 0.666512 | 4.85E-07 | 7.86E-05 | CA4 |
| H4C3 | 1.232753 | 4.83E-07 | 7.86E-05 | CA4 |
| VAMP5 | 1.346441 | 4.97E-07 | 7.98E-05 | CA4 |
| ZNF846 | -0.71882 | 5.34E-07 | 8.50E-05 | CA4 |
| S100A1 | 1.03701 | 5.52E-07 | 8.70E-05 | CA4 |
| ANTKMT | 1.352854 | 5.60E-07 | 8.74E-05 | CA4 |
| AKAP7 | -0.92174 | 5.71E-07 | 8.82E-05 | CA4 |
| LZTFL1 | -0.70483 | 6.26E-07 | 9.49E-05 | CA4 |
| RAB11B | 0.721757 | 6.21E-07 | 9.49E-05 | CA4 |
| VPS51 | 0.6708 | 6.57E-07 | 9.69E-05 | CA4 |
| THAP11 | 0.986384 | 6.55E-07 | 9.69E-05 | CA4 |
| GRINA | 0.841341 | 6.54E-07 | 9.69E-05 | CA4 |
| TRPM7 | -0.61004 | 6.96E-07 | 9.90E-05 | CA4 |
| CCDC82 | -0.97082 | 6.96E-07 | 9.90E-05 | CA4 |
| FAU | 0.894549 | 6.95E-07 | 9.90E-05 | CA4 |
| ATP5ME | 1.114758 | 7.25E-07 | 0.000102 | CA4 |
| SF3B5 | 1.075237 | 7.34E-07 | 0.000103 | CA4 |
| SYVN1 | 0.64874 | 8.25E-07 | 0.000114 | CA4 |
| H4C11 | 1.755011 | 8.37E-07 | 0.000115 | CA4 |
| KCNH2 | 0.877059 | 9.24E-07 | 0.000121 | CA4 |
| EIF3G | 0.698961 | 9.18E-07 | 0.000121 | CA4 |
| RPLP1 | 0.83592 | 9.32E-07 | 0.000121 | CA4 |
| ADIRF | 1.196961 | 9.26E-07 | 0.000121 | CA4 |
| SRFBP1 | -0.68716 | 9.26E-07 | 0.000121 | CA4 |
| LAMTOR4 | 0.861337 | 9.34E-07 | 0.000121 | CA4 |
| CCDC124 | 0.789331 | 9.54E-07 | 0.000123 | CA4 |
| H2BC12 | 1.305423 | 9.62E-07 | 0.000123 | CA4 |
| TECR | 0.75301 | 9.95E-07 | 0.000125 | CA4 |
| VPS13C | -0.63434 | 1.12E-06 | 0.00014 | CA4 |
| XAB2 | 0.726638 | 1.14E-06 | 0.00014 | CA4 |
| MPHOSPH9 | -0.73532 | 1.22E-06 | 0.000146 | CA4 |
| BLOC1S1 | 0.876404 | 1.23E-06 | 0.000146 | CA4 |

| | | | | |
|---|---|---|---|---|
| EVA1B | 1.429798 | 1.24E-06 | 0.000146 | CA4 |
| CBR4 | -0.77283 | 1.24E-06 | 0.000146 | CA4 |
| CHMP6 | 0.734302 | 1.21E-06 | 0.000146 | CA4 |
| CLPTM1 | 0.685522 | 1.31E-06 | 0.000153 | CA4 |
| H1-10 | 0.689233 | 1.34E-06 | 0.000155 | CA4 |
| G6PD | 0.728019 | 1.37E-06 | 0.000158 | CA4 |
| ZNF644 | -0.79464 | 1.40E-06 | 0.00016 | CA4 |
| KIAA2013 | 0.677981 | 1.41E-06 | 0.00016 | CA4 |
| ATR | -0.67288 | 1.44E-06 | 0.000163 | CA4 |
| NDUFB7 | 1.151907 | 1.51E-06 | 0.000169 | CA4 |
| RAD18 | -0.74723 | 1.58E-06 | 0.000175 | CA4 |
| VASN | 1.318662 | 1.59E-06 | 0.000175 | CA4 |
| CCNC | -0.97213 | 1.65E-06 | 0.000181 | CA4 |
| TMEM205 | 0.919503 | 1.70E-06 | 0.000183 | CA4 |
| RNF187 | 0.783767 | 1.70E-06 | 0.000183 | CA4 |
| DUSP23 | 1.428033 | 1.76E-06 | 0.000187 | CA4 |
| PLEKHH3 | 0.754171 | 1.89E-06 | 0.0002 | CA4 |
| GSTP1 | 0.941696 | 1.89E-06 | 0.0002 | CA4 |
| TSPAN4 | 0.910884 | 1.94E-06 | 0.000203 | CA4 |
| RBM42 | 0.792728 | 1.97E-06 | 0.000204 | CA4 |
| CORO7 | 0.7311 | 1.96E-06 | 0.000204 | CA4 |
| LSM7 | 1.092129 | 2.05E-06 | 0.000209 | CA4 |
| CRTC2 | 0.609977 | 2.04E-06 | 0.000209 | CA4 |
| VPS54 | -0.62278 | 2.08E-06 | 0.000211 | CA4 |
| PPDPF | 1.165141 | 2.10E-06 | 0.000211 | CA4 |
| SRM | 0.824876 | 2.14E-06 | 0.000214 | CA4 |
| ATP6V1F | 0.882586 | 2.17E-06 | 0.000215 | CA4 |
| UQCRQ | 0.98471 | 2.17E-06 | 0.000215 | CA4 |
| AAMP | 0.580477 | 2.20E-06 | 0.000217 | CA4 |
| PHF23 | 0.599688 | 2.24E-06 | 0.000219 | CA4 |
| PIEZO1 | 0.773504 | 2.43E-06 | 0.000229 | CA4 |
| KMT5B | -0.62337 | 2.44E-06 | 0.000229 | CA4 |
| ADRM1 | 0.715245 | 2.39E-06 | 0.000229 | CA4 |
| SPAG16 | -0.70375 | 2.43E-06 | 0.000229 | CA4 |
| RPL38 | 0.851854 | 2.37E-06 | 0.000229 | CA4 |
| BRWD1 | -0.58875 | 2.43E-06 | 0.000229 | CA4 |
| PEPD | 0.852797 | 2.49E-06 | 0.000232 | CA4 |
| SP2 | 0.697524 | 2.57E-06 | 0.000239 | CA4 |
| ETFB | 0.826421 | 2.70E-06 | 0.000249 | CA4 |
| TNFSF14 | 3.386853 | 2.82E-06 | 0.000255 | CA4 |
| ANGEL2 | -0.84531 | 2.80E-06 | 0.000255 | CA4 |
| ZNF703 | 0.987218 | 2.86E-06 | 0.000255 | CA4 |
| NKAIN2 | -0.79218 | 2.85E-06 | 0.000255 | CA4 |
| H4C2 | 1.557972 | 2.83E-06 | 0.000255 | CA4 |

| | | | | |
|---|---|---|---|---|
| HLA-DRB1 | 2.258337 | 2.96E-06 | 0.000261 | CA4 |
| ZNF891 | -0.77647 | 2.94E-06 | 0.000261 | CA4 |
| RFX1 | 0.681349 | 2.98E-06 | 0.000261 | CA4 |
| LGALS1 | 1.063973 | 3.00E-06 | 0.000262 | CA4 |
| MIF | 1.115255 | 3.08E-06 | 0.000267 | CA4 |
| DAD1 | 0.745507 | 3.22E-06 | 0.000278 | CA4 |
| ZNF704 | -0.60742 | 3.25E-06 | 0.000279 | CA4 |
| NUCB1 | 0.609548 | 3.33E-06 | 0.000284 | CA4 |
| CLPP | 0.884136 | 3.35E-06 | 0.000285 | CA4 |
| METTL26 | 0.869911 | 3.37E-06 | 0.000285 | CA4 |
| RUVBL2 | 0.636676 | 3.51E-06 | 0.000294 | CA4 |
| BIVM | -0.60658 | 3.61E-06 | 0.000299 | CA4 |
| IRF2BP1 | 0.725648 | 3.64E-06 | 0.0003 | CA4 |
| VPS26A | -0.89666 | 3.71E-06 | 0.000304 | CA4 |
| CTSD | 1.04832 | 3.79E-06 | 0.000307 | CA4 |
| ZNF292 | -0.59962 | 3.77E-06 | 0.000307 | CA4 |
| ZNF580 | 0.676599 | 3.81E-06 | 0.000308 | CA4 |
| ZNF575 | 0.987659 | 3.85E-06 | 0.000309 | CA4 |
| ACTR6 | -0.76804 | 3.91E-06 | 0.000313 | CA4 |
| TESK1 | 0.675021 | 4.07E-06 | 0.000324 | CA4 |
| OTX1 | 1.171483 | 4.12E-06 | 0.000326 | CA4 |
| CFL1 | 0.921534 | 4.14E-06 | 0.000326 | CA4 |
| CCDC85B | 1.039587 | 4.23E-06 | 0.00033 | CA4 |
| GTPBP6 | 0.756101 | 4.22E-06 | 0.00033 | CA4 |
| CEP97 | -0.74151 | 4.28E-06 | 0.000333 | CA4 |
| TMEM147 | 0.948594 | 4.32E-06 | 0.000334 | CA4 |
| OGDH | 0.601249 | 4.35E-06 | 0.000335 | CA4 |
| CCM2 | 0.637798 | 4.41E-06 | 0.000337 | CA4 |
| USP12 | -0.80059 | 4.42E-06 | 0.000337 | CA4 |
| SHARPIN | 0.70314 | 4.46E-06 | 0.000338 | CA4 |
| VPS50 | -0.63785 | 4.52E-06 | 0.000341 | CA4 |
| MT3 | 1.198226 | 4.55E-06 | 0.000342 | CA4 |
| U2AF2 | 0.671336 | 4.58E-06 | 0.000342 | CA4 |
| FZD2 | 1.721378 | 4.71E-06 | 0.000351 | CA4 |
| ELOF1 | 0.82383 | 4.77E-06 | 0.000352 | CA4 |
| ARHGDIA | 0.642801 | 4.85E-06 | 0.000356 | CA4 |
| CITED4 | 1.21538 | 4.87E-06 | 0.000356 | CA4 |
| PCBP1 | 0.677449 | 5.10E-06 | 0.000371 | CA4 |
| PSMB10 | 0.963234 | 5.17E-06 | 0.000375 | CA4 |
| IFITM3 | 1.117564 | 5.32E-06 | 0.000384 | CA4 |
| MEA1 | 0.625886 | 5.44E-06 | 0.000389 | CA4 |
| TBC1D32 | -0.64502 | 5.43E-06 | 0.000389 | CA4 |
| PCNX4 | -0.66819 | 5.52E-06 | 0.000393 | CA4 |
| ECH1 | 0.725564 | 5.67E-06 | 0.000401 | CA4 |

| | | | | |
|---|---|---|---|---|
| CCDC186 | -0.72244 | 5.71E-06 | 0.000401 | CA4 |
| PRPF39 | -0.77133 | 5.75E-06 | 0.000402 | CA4 |
| RAD17 | -0.67701 | 6.00E-06 | 0.000418 | CA4 |
| NOTCH1 | 0.941858 | 6.14E-06 | 0.000426 | CA4 |
| IBTK | -0.62236 | 6.29E-06 | 0.000433 | CA4 |
| ABCA5 | -0.85755 | 6.37E-06 | 0.000436 | CA4 |
| RHBDD2 | 1.071981 | 6.49E-06 | 0.000442 | CA4 |
| UBA5 | -0.71872 | 6.68E-06 | 0.00045 | CA4 |
| FAM135A | -0.63412 | 6.67E-06 | 0.00045 | CA4 |
| PDZRN3 | 0.690094 | 6.65E-06 | 0.00045 | CA4 |
| BRI3 | 0.639514 | 6.89E-06 | 0.00046 | CA4 |
| NOP10 | 1.337566 | 6.88E-06 | 0.00046 | CA4 |
| DUSP15 | 1.062369 | 6.93E-06 | 0.000461 | CA4 |
| SMARCAD1 | -0.60445 | 6.98E-06 | 0.000462 | CA4 |
| RGS17 | -1.15173 | 7.11E-06 | 0.000467 | CA4 |
| ZFP14 | -0.64421 | 7.11E-06 | 0.000467 | CA4 |
| RAC3 | 1.210805 | 7.21E-06 | 0.000472 | CA4 |
| COX6B1 | 1.024253 | 7.30E-06 | 0.000476 | CA4 |
| MGA | -0.58003 | 7.40E-06 | 0.00048 | CA4 |
| ELFN1 | 1.19099 | 7.48E-06 | 0.000483 | CA4 |
| LAMTOR2 | 0.670949 | 7.76E-06 | 0.0005 | CA4 |
| DRD4 | 1.356162 | 7.84E-06 | 0.000503 | CA4 |
| TMEM232 | -0.80433 | 7.94E-06 | 0.000507 | CA4 |
| C15orf39 | 0.719796 | 8.02E-06 | 0.00051 | CA4 |
| IKZF2 | -0.75858 | 8.16E-06 | 0.000515 | CA4 |
| PUS7L | -0.83193 | 8.18E-06 | 0.000515 | CA4 |
| WDR18 | 0.787983 | 8.28E-06 | 0.000515 | CA4 |
| SLC22A17 | 0.85005 | 8.27E-06 | 0.000515 | CA4 |
| CCDC138 | -0.90859 | 8.23E-06 | 0.000515 | CA4 |
| SOS2 | -0.60462 | 8.50E-06 | 0.000526 | CA4 |
| WDR83OS | 0.770194 | 8.77E-06 | 0.000541 | CA4 |
| SNX13 | -0.6048 | 8.94E-06 | 0.000541 | CA4 |
| RTL8C | 0.846191 | 8.87E-06 | 0.000541 | CA4 |
| SCRN3 | -0.71564 | 8.84E-06 | 0.000541 | CA4 |
| TATDN1 | -0.76778 | 8.95E-06 | 0.000541 | CA4 |
| ACY1 | 0.795867 | 8.97E-06 | 0.000541 | CA4 |
| SMIM10L1 | -0.77231 | 8.89E-06 | 0.000541 | CA4 |
| WDR81 | 0.582452 | 9.13E-06 | 0.000549 | CA4 |
| GYPC | 1.182495 | 9.43E-06 | 0.000565 | CA4 |
| TMEM160 | 1.066708 | 9.59E-06 | 0.000566 | CA4 |
| IFITM2 | 1.363342 | 9.54E-06 | 0.000566 | CA4 |
| CEP78 | -0.65766 | 9.71E-06 | 0.000571 | CA4 |
| F8A3 | 0.803366 | 9.83E-06 | 0.000576 | CA4 |
| MYL6 | 0.62192 | 9.99E-06 | 0.000583 | CA4 |

| | | | | |
|---|---|---|---|---|
| GCFC2 | -0.68656 | 1.02E-05 | 0.00059 | CA4 |
| EIF6 | 0.733119 | 1.02E-05 | 0.00059 | CA4 |
| ATP8A1 | -0.65496 | 1.05E-05 | 0.0006 | CA4 |
| INTS8 | -0.59298 | 1.05E-05 | 0.0006 | CA4 |
| SLC10A3 | 0.767763 | 1.06E-05 | 0.0006 | CA4 |
| METTL25 | -0.8581 | 1.09E-05 | 0.000612 | CA4 |
| ZNF283 | -0.7259 | 1.09E-05 | 0.000612 | CA4 |
| DNLZ | 1.293872 | 1.09E-05 | 0.000612 | CA4 |
| NME3 | 0.729319 | 1.15E-05 | 0.000628 | CA4 |
| CHCHD2 | 0.720354 | 1.14E-05 | 0.000628 | CA4 |
| SLC27A1 | 0.656334 | 1.14E-05 | 0.000628 | CA4 |
| ZNF23 | -0.84394 | 1.13E-05 | 0.000628 | CA4 |
| LRRC24 | 0.927112 | 1.13E-05 | 0.000628 | CA4 |
| RIF1 | -0.63102 | 1.18E-05 | 0.000647 | CA4 |
| TRIP11 | -0.70936 | 1.27E-05 | 0.000678 | CA4 |
| FOXP4 | 0.668552 | 1.28E-05 | 0.000681 | CA4 |
| HCST | 1.94416 | 1.31E-05 | 0.000689 | CA4 |
| H3C13 | 1.928391 | 1.31E-05 | 0.000689 | CA4 |
| FAM107B | -1.08102 | 1.34E-05 | 0.000695 | CA4 |
| TAF6L | 0.595091 | 1.34E-05 | 0.000695 | CA4 |
| ZDHHC20 | -0.80151 | 1.34E-05 | 0.000695 | CA4 |
| PSMB8 | 0.905649 | 1.34E-05 | 0.000695 | CA4 |
| ECHDC1 | -0.67927 | 1.41E-05 | 0.000722 | CA4 |
| PC | 0.639383 | 1.40E-05 | 0.000722 | CA4 |
| ZNF277 | -0.63082 | 1.41E-05 | 0.000722 | CA4 |
| C1QL1 | 1.185157 | 1.45E-05 | 0.000736 | CA4 |
| LDOC1 | 0.913605 | 1.46E-05 | 0.000738 | CA4 |
| DDTL | 0.951086 | 1.48E-05 | 0.000745 | CA4 |
| EMX2 | 0.950499 | 1.48E-05 | 0.000745 | CA4 |
| TOX2 | 0.982938 | 1.49E-05 | 0.000747 | CA4 |
| PRICKLE3 | 0.871028 | 1.50E-05 | 0.000747 | CA4 |
| TTLL7 | -0.89926 | 1.53E-05 | 0.000756 | CA4 |
| ZGRF1 | -0.94019 | 1.53E-05 | 0.000756 | CA4 |
| SAP130 | 0.703759 | 1.55E-05 | 0.000764 | CA4 |
| MTLN | 1.401832 | 1.57E-05 | 0.000772 | CA4 |
| FBL | 0.715346 | 1.59E-05 | 0.000774 | CA4 |
| TUBG1 | 0.673323 | 1.60E-05 | 0.000776 | CA4 |
| MTCH1 | 0.654966 | 1.62E-05 | 0.000781 | CA4 |
| ZNF654 | -0.68822 | 1.62E-05 | 0.000781 | CA4 |
| MTX1 | 0.654869 | 1.64E-05 | 0.000785 | CA4 |
| PMS1 | -0.61527 | 1.69E-05 | 0.000806 | CA4 |
| MTRES1 | -0.65779 | 1.71E-05 | 0.000812 | CA4 |
| RPLP2 | 0.826811 | 1.80E-05 | 0.000851 | CA4 |
| DNAJC24 | -0.72682 | 1.91E-05 | 0.000892 | CA4 |

| | | | | |
|---|---|---|---|---|
| TRAK2 | -0.73777 | 1.94E-05 | 0.000896 | CA4 |
| H2AC19 | 1.066221 | 1.95E-05 | 0.000898 | CA4 |
| NHP2 | 0.644068 | 2.00E-05 | 0.000915 | CA4 |
| CDC40 | -0.75053 | 2.01E-05 | 0.000915 | CA4 |
| SSC5D | 1.093276 | 2.01E-05 | 0.000915 | CA4 |
| THAP4 | 0.667408 | 2.05E-05 | 0.000929 | CA4 |
| CCDC22 | 0.742961 | 2.07E-05 | 0.000932 | CA4 |
| PPP4C | 0.586903 | 2.10E-05 | 0.000943 | CA4 |
| MAP3K2 | -0.63828 | 2.10E-05 | 0.000943 | CA4 |
| NME2 | 0.617523 | 2.14E-05 | 0.000953 | CA4 |
| ATPSCKMT | -0.79294 | 2.18E-05 | 0.000962 | CA4 |
| SNRNP25 | 0.682604 | 2.19E-05 | 0.000962 | CA4 |
| SPSB3 | 0.580335 | 2.17E-05 | 0.000962 | CA4 |
| RING1 | 0.587572 | 2.18E-05 | 0.000962 | CA4 |
| CHST7 | 1.080248 | 2.21E-05 | 0.000971 | CA4 |
| CFAP69 | -0.89508 | 2.24E-05 | 0.000981 | CA4 |
| AURKAIP1 | 0.860424 | 2.27E-05 | 0.000987 | CA4 |
| CST3 | 1.010134 | 2.28E-05 | 0.000987 | CA4 |
| FSD1L | -0.71006 | 2.30E-05 | 0.000994 | CA4 |
| TMEM94 | 0.655401 | 2.31E-05 | 0.000997 | CA4 |
| ESF1 | -0.66841 | 2.34E-05 | 0.001 | CA4 |
| IDH3G | 0.865599 | 2.36E-05 | 0.001003 | CA4 |
| RASA2 | -0.80387 | 2.37E-05 | 0.001003 | CA4 |
| GLMN | -0.79332 | 2.37E-05 | 0.001003 | CA4 |
| CARD19 | 0.832826 | 2.39E-05 | 0.001008 | CA4 |
| HLA-DQA1 | 3.088755 | 2.40E-05 | 0.00101 | CA4 |
| TERF1 | -0.68128 | 2.41E-05 | 0.001012 | CA4 |
| KIAA0586 | -0.59909 | 2.44E-05 | 0.001023 | CA4 |
| ENDOG | 1.017927 | 2.57E-05 | 0.001076 | CA4 |
| ATM | -0.64167 | 2.59E-05 | 0.001077 | CA4 |
| JUP | 0.898083 | 2.69E-05 | 0.001113 | CA4 |
| NAA80 | 0.739473 | 2.73E-05 | 0.001123 | CA4 |
| TIMM17B | 0.63032 | 2.87E-05 | 0.001175 | CA4 |
| SOD3 | 1.438105 | 2.89E-05 | 0.001177 | CA4 |
| HSPB6 | 1.326041 | 2.94E-05 | 0.001184 | CA4 |
| FBXW5 | 0.671457 | 2.93E-05 | 0.001184 | CA4 |
| IRS2 | 0.636679 | 2.94E-05 | 0.001184 | CA4 |
| RPL36AL | 0.945463 | 3.02E-05 | 0.00121 | CA4 |
| RUFY2 | -0.63323 | 3.05E-05 | 0.001215 | CA4 |
| SLC15A3 | 0.937811 | 3.06E-05 | 0.001218 | CA4 |
| LINS1 | -0.74087 | 3.10E-05 | 0.001231 | CA4 |
| SNRPA | 0.700607 | 3.12E-05 | 0.001232 | CA4 |
| NDUFB11 | 0.801585 | 3.13E-05 | 0.001232 | CA4 |
| ADRA2C | 1.126624 | 3.16E-05 | 0.00124 | CA4 |

| | | | | |
|---|---|---|---|---|
| FBXO2 | 0.857742 | 3.18E-05 | 0.00124 | CA4 |
| NRM | 0.996304 | 3.19E-05 | 0.00124 | CA4 |
| UBB | 0.733116 | 3.19E-05 | 0.00124 | CA4 |
| ALKBH7 | 0.762469 | 3.21E-05 | 0.001246 | CA4 |
| POLK | -0.66932 | 3.24E-05 | 0.001256 | CA4 |
| GUCY1A2 | -0.89378 | 3.29E-05 | 0.001272 | CA4 |
| MRPS26 | 0.718809 | 3.32E-05 | 0.001278 | CA4 |
| TMEM109 | 0.833906 | 3.39E-05 | 0.001296 | CA4 |
| AC002996.1 | 0.947519 | 3.39E-05 | 0.001296 | CA4 |
| SLC3A2 | 0.652665 | 3.46E-05 | 0.001317 | CA4 |
| CORO1B | 0.754311 | 3.54E-05 | 0.001343 | CA4 |
| TVP23B | -0.73755 | 3.56E-05 | 0.001345 | CA4 |
| FXYD5 | 0.999998 | 3.61E-05 | 0.001362 | CA4 |
| APRT | 0.630293 | 3.65E-05 | 0.001373 | CA4 |
| WDR24 | 0.608863 | 3.72E-05 | 0.001395 | CA4 |
| ACTN4 | 0.665991 | 3.78E-05 | 0.001406 | CA4 |
| SLC52A2 | 0.881564 | 3.77E-05 | 0.001406 | CA4 |
| MRPL55 | 0.609454 | 3.85E-05 | 0.001429 | CA4 |
| TMUB1 | 0.812728 | 3.88E-05 | 0.001436 | CA4 |
| PEX14 | 0.633329 | 3.95E-05 | 0.001459 | CA4 |
| KIF20B | -0.91047 | 4.07E-05 | 0.00149 | CA4 |
| MICALL2 | 0.672795 | 4.08E-05 | 0.001492 | CA4 |
| EPHB3 | 0.882761 | 4.14E-05 | 0.001507 | CA4 |
| GTDC1 | -0.63224 | 4.19E-05 | 0.001519 | CA4 |
| TLNRD1 | 0.675026 | 4.25E-05 | 0.001528 | CA4 |
| PFKL | 0.590064 | 4.25E-05 | 0.001528 | CA4 |
| DENND11 | -0.58061 | 4.25E-05 | 0.001528 | CA4 |
| PEX16 | 0.589808 | 4.30E-05 | 0.001539 | CA4 |
| SCNM1 | 0.970426 | 4.31E-05 | 0.00154 | CA4 |
| TGFB1 | 0.849524 | 4.34E-05 | 0.001547 | CA4 |
| NUDCD1 | -0.73551 | 4.41E-05 | 0.001567 | CA4 |
| H4C12 | 1.411217 | 4.44E-05 | 0.001576 | CA4 |
| BLVRB | 0.807796 | 4.50E-05 | 0.001593 | CA4 |
| RAPGEF5 | -0.82716 | 4.58E-05 | 0.001601 | CA4 |
| NTSR2 | 1.125264 | 4.55E-05 | 0.001601 | CA4 |
| RASGRP4 | 1.294394 | 4.58E-05 | 0.001601 | CA4 |
| GLDC | 0.850672 | 4.57E-05 | 0.001601 | CA4 |
| PMM1 | 0.6076 | 4.66E-05 | 0.001623 | CA4 |
| ANKRD36 | -0.84755 | 4.74E-05 | 0.001633 | CA4 |
| NRROS | 0.872577 | 4.78E-05 | 0.001642 | CA4 |
| PLXND1 | 0.732156 | 4.85E-05 | 0.001662 | CA4 |
| RPL8 | 0.779413 | 4.88E-05 | 0.00167 | CA4 |
| HIGD2A | 0.646981 | 4.91E-05 | 0.001671 | CA4 |
| KIF7 | 0.703784 | 4.98E-05 | 0.001676 | CA4 |

| | | | | |
|---|---|---|---|---|
| MZT2A | 0.758946 | 4.97E-05 | 0.001676 | CA4 |
| MPEG1 | 1.566241 | 4.94E-05 | 0.001676 | CA4 |
| H2AC21 | 0.885241 | 5.01E-05 | 0.00168 | CA4 |
| ZBTB44 | -0.71502 | 5.01E-05 | 0.00168 | CA4 |
| STK17B | -0.93895 | 5.09E-05 | 0.001699 | CA4 |
| SCAMP3 | 0.64514 | 5.12E-05 | 0.001702 | CA4 |
| ARL14EP | -0.65987 | 5.17E-05 | 0.001712 | CA4 |
| ST3GAL6 | -0.67721 | 5.22E-05 | 0.001717 | CA4 |
| CYBA | 1.138981 | 5.25E-05 | 0.001722 | CA4 |
| TBCB | 0.748388 | 5.30E-05 | 0.00173 | CA4 |
| TTC26 | -0.80326 | 5.33E-05 | 0.00173 | CA4 |
| HINT3 | -0.75049 | 5.31E-05 | 0.00173 | CA4 |
| SAXO2 | -0.8767 | 5.32E-05 | 0.00173 | CA4 |
| CLIP2 | 0.606334 | 5.35E-05 | 0.001732 | CA4 |
| PURA | -0.73468 | 5.39E-05 | 0.001735 | CA4 |
| PIGT | 0.687955 | 5.43E-05 | 0.001741 | CA4 |
| IRF2BPL | 0.663084 | 5.48E-05 | 0.001751 | CA4 |
| AC024592.3 | 1.338886 | 5.48E-05 | 0.001751 | CA4 |
| GALNT13 | -0.70626 | 5.52E-05 | 0.001758 | CA4 |
| NLRX1 | 0.720619 | 5.64E-05 | 0.001791 | CA4 |
| CBX3 | -0.58742 | 5.67E-05 | 0.001796 | CA4 |
| DNAJC2 | -0.69247 | 5.78E-05 | 0.001824 | CA4 |
| LGI4 | 0.831077 | 5.83E-05 | 0.001838 | CA4 |
| DPM3 | 0.93878 | 5.89E-05 | 0.001844 | CA4 |
| ZNF784 | 0.857591 | 5.87E-05 | 0.001844 | CA4 |
| TUBB | 0.685021 | 5.92E-05 | 0.001844 | CA4 |
| ZNF777 | 0.584538 | 5.91E-05 | 0.001844 | CA4 |
| IFT80 | -0.70453 | 6.15E-05 | 0.001901 | CA4 |
| VPS36 | -0.72936 | 6.21E-05 | 0.001917 | CA4 |
| H2BC21 | 0.907922 | 6.25E-05 | 0.001922 | CA4 |
| HAUS3 | -0.8039 | 6.25E-05 | 0.001922 | CA4 |
| ERF | 0.642583 | 6.28E-05 | 0.001926 | CA4 |
| KATNBL1 | -0.67589 | 6.33E-05 | 0.001938 | CA4 |
| TRMT9B | -0.7925 | 6.36E-05 | 0.001939 | CA4 |
| SLC25A1 | 0.780213 | 6.40E-05 | 0.001939 | CA4 |
| GAMT | 0.776201 | 6.38E-05 | 0.001939 | CA4 |
| NR1H2 | 0.584186 | 6.39E-05 | 0.001939 | CA4 |
| MIGA1 | -0.65006 | 6.42E-05 | 0.001939 | CA4 |
| AGT | 0.973701 | 6.45E-05 | 0.001944 | CA4 |
| TICAM1 | 0.654777 | 6.56E-05 | 0.001972 | CA4 |
| SLF1 | -0.69831 | 6.56E-05 | 0.001972 | CA4 |
| RPS24 | 0.716485 | 6.58E-05 | 0.001974 | CA4 |
| IMP3 | 1.034716 | 6.63E-05 | 0.001982 | CA4 |
| WBP1 | 0.627168 | 6.67E-05 | 0.00199 | CA4 |

| | | | | |
|---|---|---|---|---|
| TRDMT1 | -0.76556 | 6.70E-05 | 0.001993 | CA4 |
| CPLANE1 | -0.66311 | 6.71E-05 | 0.001993 | CA4 |
| KIAA0825 | -0.6182 | 6.77E-05 | 0.002009 | CA4 |
| COX4I1 | 0.641046 | 6.79E-05 | 0.002011 | CA4 |
| SEC22A | -0.70644 | 6.81E-05 | 0.002013 | CA4 |
| DCAF15 | 0.8132 | 6.84E-05 | 0.002015 | CA4 |
| FLNC | 1.413701 | 6.91E-05 | 0.002031 | CA4 |
| UBE2S | 0.809655 | 6.96E-05 | 0.002035 | CA4 |
| RPL12 | 0.770908 | 6.96E-05 | 0.002035 | CA4 |
| SMC6 | -0.60385 | 6.99E-05 | 0.002038 | CA4 |
| SCAMP4 | 0.596242 | 7.36E-05 | 0.002129 | CA4 |
| CYB5R4 | -0.68532 | 7.49E-05 | 0.00215 | CA4 |
| RALY | 0.641687 | 7.51E-05 | 0.002153 | CA4 |
| APOE | 0.929211 | 7.52E-05 | 0.002153 | CA4 |
| EIF1AX | -0.7593 | 7.55E-05 | 0.002157 | CA4 |
| COX5A | 0.68008 | 7.62E-05 | 0.002173 | CA4 |
| TELO2 | 0.607464 | 7.65E-05 | 0.002176 | CA4 |
| MRPL34 | 0.919118 | 7.82E-05 | 0.002219 | CA4 |
| RRAD | 1.82703 | 7.81E-05 | 0.002219 | CA4 |
| C6orf226 | 1.231607 | 8.02E-05 | 0.002264 | CA4 |
| MMP16 | -0.91618 | 8.07E-05 | 0.002273 | CA4 |
| NDUFB2 | 0.72927 | 8.16E-05 | 0.002293 | CA4 |
| RNASEK-C17orf49 | 1.181045 | 8.26E-05 | 0.002314 | CA4 |
| CEP290 | -0.93941 | 8.27E-05 | 0.002314 | CA4 |
| RPL36 | 0.679827 | 8.35E-05 | 0.002333 | CA4 |
| TPD52L1 | 0.698831 | 8.46E-05 | 0.002355 | CA4 |
| ABCD1 | 0.742961 | 8.50E-05 | 0.002357 | CA4 |
| ZBTB6 | -0.6355 | 8.52E-05 | 0.00236 | CA4 |
| ZKSCAN7 | -0.7973 | 8.55E-05 | 0.002363 | CA4 |
| BPNT2 | -0.63287 | 8.60E-05 | 0.002368 | CA4 |
| UBE3D | -0.61452 | 8.60E-05 | 0.002368 | CA4 |
| NAA25 | -0.62408 | 8.69E-05 | 0.002381 | CA4 |
| GPAA1 | 0.58397 | 8.68E-05 | 0.002381 | CA4 |
| CDK6 | -1.03262 | 8.75E-05 | 0.002383 | CA4 |
| U2SURP | -0.63044 | 8.77E-05 | 0.002383 | CA4 |
| IFI27L2 | 0.771099 | 8.80E-05 | 0.002384 | CA4 |
| TLE5 | 0.624881 | 8.90E-05 | 0.002402 | CA4 |
| PFDN2 | 1.009557 | 8.96E-05 | 0.00241 | CA4 |
| COPS2 | -0.62084 | 9.12E-05 | 0.002442 | CA4 |
| NOC4L | 0.800254 | 9.11E-05 | 0.002442 | CA4 |
| RNF181 | 0.613341 | 9.14E-05 | 0.002442 | CA4 |
| CCDC102B | -0.67018 | 9.21E-05 | 0.002458 | CA4 |
| RNF166 | 0.588193 | 9.38E-05 | 0.00249 | CA4 |
| GDAP1 | -0.69033 | 9.54E-05 | 0.002508 | CA4 |

| | | | | |
|---|---|---|---|---|
| TFAM | -0.61412 | 9.49E-05 | 0.002508 | CA4 |
| KLHL5 | -0.59508 | 9.55E-05 | 0.002508 | CA4 |
| ANO7 | 1.000247 | 9.49E-05 | 0.002508 | CA4 |
| TAS2R14 | -1.00409 | 9.50E-05 | 0.002508 | CA4 |
| ARID4A | -0.64444 | 9.58E-05 | 0.002512 | CA4 |
| ANKRD36C | -0.71059 | 9.73E-05 | 0.002542 | CA4 |
| GK5 | -0.65001 | 9.85E-05 | 0.002556 | CA4 |
| CCDC30 | -0.73228 | 9.84E-05 | 0.002556 | CA4 |
| RTL8A | 0.667618 | 9.83E-05 | 0.002556 | CA4 |
| YBX1 | 0.708363 | 9.94E-05 | 0.002569 | CA4 |
| BLZF1 | -0.66179 | 9.94E-05 | 0.002569 | CA4 |
| YIF1A | 0.766936 | 9.93E-05 | 0.002569 | CA4 |
| GRN | 0.819778 | 0.0001 | 0.002574 | CA4 |
| CRAT | 0.634796 | 0.0001 | 0.002574 | CA4 |
| NECAB1 | -1.02319 | 0.000101 | 0.002574 | CA4 |
| DTX1 | 0.731004 | 0.0001 | 0.002574 | CA4 |
| COX5B | 0.652149 | 0.0001 | 0.002574 | CA4 |
| CEP83 | -0.74833 | 0.000101 | 0.002574 | CA4 |
| UQCR10 | 0.816185 | 0.000101 | 0.002574 | CA4 |
| AGRN | 0.709784 | 0.000101 | 0.002574 | CA4 |
| NECTIN2 | 0.882464 | 0.000102 | 0.002577 | CA4 |
| SHPRH | -0.6641 | 0.000102 | 0.002577 | CA4 |
| SOS1 | -0.58031 | 0.000102 | 0.002577 | CA4 |
| KRR1 | -0.73488 | 0.000102 | 0.002585 | CA4 |
| MICU2 | -0.63623 | 0.000102 | 0.002585 | CA4 |
| SHISA5 | 0.617345 | 0.000103 | 0.002591 | CA4 |
| UQCR11 | 0.683363 | 0.000103 | 0.002597 | CA4 |
| DPEP2 | 1.506605 | 0.000104 | 0.00261 | CA4 |
| ADPRS | 0.811345 | 0.000105 | 0.002627 | CA4 |
| CD320 | 0.815546 | 0.000105 | 0.002627 | CA4 |
| FBLN1 | 0.99123 | 0.000107 | 0.002655 | CA4 |
| BLOC1S4 | 0.783994 | 0.000107 | 0.002655 | CA4 |
| BLOC1S3 | 1.131313 | 0.000108 | 0.002668 | CA4 |
| OTOS | 1.934237 | 0.000108 | 0.00267 | CA4 |
| TIA1 | -0.59371 | 0.000108 | 0.002675 | CA4 |
| MAF1 | 0.671245 | 0.000109 | 0.002702 | CA4 |
| DENR | -0.5967 | 0.000111 | 0.002735 | CA4 |
| LSM10 | 0.663777 | 0.000113 | 0.002771 | CA4 |
| STXBP3 | -0.60123 | 0.000115 | 0.002812 | CA4 |
| IFT81 | -0.62174 | 0.000116 | 0.002812 | CA4 |
| CAPN5 | 0.60661 | 0.000116 | 0.002812 | CA4 |
| PIM3 | 0.930173 | 0.000116 | 0.002812 | CA4 |
| ZNF37A | -0.66712 | 0.000116 | 0.002816 | CA4 |
| ZZZ3 | -0.67795 | 0.000119 | 0.00284 | CA4 |

| | | | | |
|---|---|---|---|---|
| PSD4 | 0.589474 | 0.000119 | 0.00284 | CA4 |
| NDUFB5 | -0.60449 | 0.000118 | 0.00284 | CA4 |
| SH3BGRL3 | 0.601146 | 0.000119 | 0.00284 | CA4 |
| LRRC4C | -0.5937 | 0.000119 | 0.00284 | CA4 |
| GATD3A | 0.594297 | 0.000119 | 0.00284 | CA4 |
| SLC35E3 | -0.58067 | 0.000119 | 0.00284 | CA4 |
| ZNF518A | -0.86312 | 0.000118 | 0.00284 | CA4 |
| TTC3 | -0.65425 | 0.000118 | 0.00284 | CA4 |
| COMT | 0.69715 | 0.00012 | 0.00284 | CA4 |
| PPIB | 0.743716 | 0.00012 | 0.00284 | CA4 |
| PGLS | 0.664262 | 0.000121 | 0.002855 | CA4 |
| MGRN1 | 0.641228 | 0.000123 | 0.002892 | CA4 |
| FKBP3 | -0.58931 | 0.000124 | 0.002917 | CA4 |
| RFXANK | 0.653857 | 0.000125 | 0.002931 | CA4 |
| H4C5 | 1.168033 | 0.000126 | 0.002938 | CA4 |
| NDUFS5 | 1.045153 | 0.000127 | 0.002949 | CA4 |
| ANAPC4 | -0.63675 | 0.000127 | 0.002959 | CA4 |
| ANKRD26 | -0.91949 | 0.000127 | 0.002959 | CA4 |
| LRP5 | 0.949213 | 0.000128 | 0.002959 | CA4 |
| NDUFB9 | 0.785166 | 0.000128 | 0.002961 | CA4 |
| COPZ2 | 0.814748 | 0.000131 | 0.003013 | CA4 |
| PLCL1 | -0.77742 | 0.000131 | 0.003013 | CA4 |
| VKORC1 | 0.610471 | 0.000131 | 0.003013 | CA4 |
| IFITM1 | 1.530668 | 0.000131 | 0.003013 | CA4 |
| CDK11B | 0.63241 | 0.000131 | 0.003013 | CA4 |
| INAFM1 | 0.65868 | 0.000132 | 0.003015 | CA4 |
| NECAP2 | 0.677402 | 0.000133 | 0.003045 | CA4 |
| PLCD1 | 0.846182 | 0.000134 | 0.003048 | CA4 |
| NAALAD2 | -0.92759 | 0.000134 | 0.003054 | CA4 |
| ZNF345 | -0.71704 | 0.000137 | 0.003118 | CA4 |
| CRB2 | 1.019666 | 0.000138 | 0.003122 | CA4 |
| PHRF1 | 0.653542 | 0.000139 | 0.003137 | CA4 |
| ARMC5 | 0.673176 | 0.000139 | 0.003137 | CA4 |
| COX7A1 | 1.280554 | 0.000139 | 0.003137 | CA4 |
| MT2A | 1.242179 | 0.00014 | 0.003141 | CA4 |
| MAGOHB | -0.78148 | 0.000142 | 0.003177 | CA4 |
| COA3 | 0.58197 | 0.000142 | 0.00318 | CA4 |
| SCARF2 | 0.658411 | 0.000142 | 0.00318 | CA4 |
| TMEM54 | 1.053365 | 0.000144 | 0.003218 | CA4 |
| CASP8AP2 | -0.75953 | 0.000145 | 0.003231 | CA4 |
| SDF2L1 | 1.313069 | 0.000146 | 0.00325 | CA4 |
| CRIPT | -0.58776 | 0.000148 | 0.003272 | CA4 |
| PM20D2 | -0.82936 | 0.000148 | 0.003272 | CA4 |
| ZNF540 | -0.88478 | 0.000148 | 0.003279 | CA4 |

| | | | | |
|---|---|---|---|---|
| TMEM88 | 2.010143 | 0.00015 | 0.003318 | CA4 |
| MTERF1 | -0.98144 | 0.000154 | 0.00338 | CA4 |
| ERCC8 | -0.63849 | 0.000159 | 0.003467 | CA4 |
| CASD1 | -0.64017 | 0.000159 | 0.003467 | CA4 |
| NDUFA5 | -0.79403 | 0.00016 | 0.003471 | CA4 |
| TEX15 | -0.8964 | 0.00016 | 0.003471 | CA4 |
| GNG5 | 0.921035 | 0.00016 | 0.003471 | CA4 |
| MRPS18A | 0.604334 | 0.000161 | 0.003485 | CA4 |
| DPP7 | 0.596978 | 0.000162 | 0.003498 | CA4 |
| TUBB4B | 0.871941 | 0.000164 | 0.003535 | CA4 |
| COPRS | 0.735475 | 0.000166 | 0.003557 | CA4 |
| ACTB | 0.669793 | 0.000167 | 0.003571 | CA4 |
| ADCK5 | 0.714056 | 0.000167 | 0.003571 | CA4 |
| AC011462.1 | 3.465653 | 0.000167 | 0.003571 | CA4 |
| SART1 | 0.633023 | 0.000168 | 0.003584 | CA4 |
| SMC2 | -0.64662 | 0.000171 | 0.003637 | CA4 |
| FAM98B | -0.61814 | 0.000174 | 0.003678 | CA4 |
| TIMP1 | 1.090005 | 0.000175 | 0.003683 | CA4 |
| ZNF706 | -0.60379 | 0.000177 | 0.003728 | CA4 |
| VPS13A | -0.70204 | 0.000178 | 0.003754 | CA4 |
| NDUFS6 | 0.808759 | 0.000179 | 0.003757 | CA4 |
| SLC38A9 | -0.62257 | 0.000179 | 0.00376 | CA4 |
| CCDC152 | -0.89402 | 0.00018 | 0.00378 | CA4 |
| ECE1 | 0.658661 | 0.000181 | 0.00379 | CA4 |
| IGIP | -0.69098 | 0.000181 | 0.003791 | CA4 |
| RWDD4 | -0.65273 | 0.000183 | 0.003811 | CA4 |
| QPRT | 0.756925 | 0.000187 | 0.003893 | CA4 |
| GABARAPL1 | -0.93868 | 0.000188 | 0.003893 | CA4 |
| GET3 | 0.629689 | 0.000188 | 0.003893 | CA4 |
| ZNF708 | -0.73172 | 0.000189 | 0.003916 | CA4 |
| SCML2 | -0.66796 | 0.00019 | 0.003926 | CA4 |
| FASN | 0.601962 | 0.000191 | 0.003949 | CA4 |
| CBWD2 | -0.63399 | 0.000193 | 0.003984 | CA4 |
| MFSD10 | 0.608785 | 0.000195 | 0.004011 | CA4 |
| RNPEPL1 | 0.684712 | 0.000196 | 0.00402 | CA4 |
| ZNF678 | -1.35412 | 0.000196 | 0.00402 | CA4 |
| NDUFA1 | 0.947559 | 0.000197 | 0.004026 | CA4 |
| FAM133A | -0.76769 | 0.000199 | 0.004057 | CA4 |
| ARVCF | 0.659753 | 0.0002 | 0.004072 | CA4 |
| RPS27AP5 | 0.794825 | 0.000201 | 0.004087 | CA4 |
| ETHE1 | 0.68032 | 0.000202 | 0.004093 | CA4 |
| SMN1 | -0.7587 | 0.000204 | 0.004123 | CA4 |
| SMIM13 | -0.63383 | 0.000205 | 0.004132 | CA4 |
| CARNMT1 | -0.72062 | 0.000206 | 0.004142 | CA4 |

| | | | | |
|---|---|---|---|---|
| GPR180 | -0.6546 | 0.000209 | 0.004178 | CA4 |
| DCUN1D1 | -0.60926 | 0.000209 | 0.004179 | CA4 |
| RPS2 | 0.745168 | 0.00021 | 0.004195 | CA4 |
| N4BP2 | -0.81883 | 0.000213 | 0.00422 | CA4 |
| CATSPERE | -0.82945 | 0.000213 | 0.00422 | CA4 |
| NDUFA13 | 0.879321 | 0.000213 | 0.00422 | CA4 |
| TFPT | 0.615384 | 0.000215 | 0.004247 | CA4 |
| FBXL15 | 0.694378 | 0.000218 | 0.004289 | CA4 |
| GTPBP10 | -0.64613 | 0.00022 | 0.004333 | CA4 |
| CRLF1 | 1.490083 | 0.000225 | 0.004375 | CA4 |
| SECISBP2L | -0.71861 | 0.000225 | 0.004375 | CA4 |
| FAM126B | -0.62809 | 0.000223 | 0.004375 | CA4 |
| CMPK1 | -0.61179 | 0.000224 | 0.004375 | CA4 |
| MFSD3 | 0.86579 | 0.000225 | 0.004375 | CA4 |
| WDHD1 | -0.62214 | 0.000225 | 0.004375 | CA4 |
| VAMP8 | 1.423894 | 0.000227 | 0.00439 | CA4 |
| LAMB2 | 0.798287 | 0.000227 | 0.00439 | CA4 |
| ZFP62 | -0.87668 | 0.000227 | 0.00439 | CA4 |
| MED16 | 0.598731 | 0.000228 | 0.004392 | CA4 |
| ATP6V0B | 0.658463 | 0.00023 | 0.004413 | CA4 |
| ADGRF3 | 1.223782 | 0.000231 | 0.00443 | CA4 |
| ITGB4 | 0.911698 | 0.000232 | 0.004441 | CA4 |
| ACKR3 | 0.867622 | 0.000233 | 0.004441 | CA4 |
| UQCRC1 | 0.585168 | 0.000236 | 0.004485 | CA4 |
| RRAS | 0.922665 | 0.000235 | 0.004485 | CA4 |
| SQOR | 0.867652 | 0.000237 | 0.0045 | CA4 |
| BORCS6 | 0.651872 | 0.000237 | 0.0045 | CA4 |
| SMIM1 | 1.766401 | 0.000237 | 0.0045 | CA4 |
| ZNF483 | -0.67483 | 0.00024 | 0.004519 | CA4 |
| UQCRH | 0.779311 | 0.00024 | 0.004519 | CA4 |
| ILVBL | 0.583354 | 0.000242 | 0.004551 | CA4 |
| ZMYM5 | -0.66845 | 0.000242 | 0.004553 | CA4 |
| COL6A1 | 0.596864 | 0.000245 | 0.004582 | CA4 |
| BAG3 | 0.983216 | 0.000247 | 0.004626 | CA4 |
| PRKG2 | -0.77552 | 0.00025 | 0.004667 | CA4 |
| NR1D1 | 0.85545 | 0.000251 | 0.004684 | CA4 |
| ERGIC2 | -0.64994 | 0.000253 | 0.004704 | CA4 |
| MSANTD3-TMEFF1 | -1.16042 | 0.000254 | 0.004723 | CA4 |
| ANKAR | -0.61202 | 0.000255 | 0.004727 | CA4 |
| SLC6A15 | -0.75926 | 0.000258 | 0.004767 | CA4 |
| SPEF2 | -0.79221 | 0.000258 | 0.004767 | CA4 |
| KRT19 | 1.915663 | 0.00026 | 0.004794 | CA4 |
| MYZAP | 3.138901 | 0.000262 | 0.004805 | CA4 |
| ATG101 | 0.746768 | 0.000264 | 0.004835 | CA4 |

| | | | | |
|---|---|---|---|---|
| RTN4RL2 | 1.249152 | 0.000265 | 0.00485 | CA4 |
| NAP1L1 | -0.65145 | 0.000265 | 0.00485 | CA4 |
| CFD | 1.932312 | 0.000266 | 0.004856 | CA4 |
| EPM2AIP1 | -0.60378 | 0.00027 | 0.004902 | CA4 |
| CD74 | 1.315489 | 0.000271 | 0.004909 | CA4 |
| NAT14 | 0.836687 | 0.000271 | 0.004909 | CA4 |
| ATP1A4 | 1.439626 | 0.000274 | 0.004954 | CA4 |
| SNRNP48 | -0.62643 | 0.000277 | 0.004992 | CA4 |
| CCR10 | 1.035402 | 0.000277 | 0.004992 | CA4 |
| GNAI2 | 0.639806 | 0.00028 | 0.005035 | CA4 |
| C4orf33 | -0.79687 | 0.00028 | 0.005035 | CA4 |
| KIF27 | -0.70673 | 0.000284 | 0.005076 | CA4 |
| IRX1 | 1.35043 | 0.000283 | 0.005076 | CA4 |
| VN1R1 | -0.86394 | 0.000284 | 0.005076 | CA4 |
| CASTOR2 | 0.597829 | 0.000289 | 0.005139 | CA4 |
| SMIM14 | -0.58301 | 0.00029 | 0.005148 | CA4 |
| ATP13A2 | 0.596873 | 0.000291 | 0.005158 | CA4 |
| THAP5 | -0.75986 | 0.000291 | 0.005168 | CA4 |
| MPG | 0.705563 | 0.000295 | 0.005202 | CA4 |
| CNTLN | -0.59111 | 0.000296 | 0.005206 | CA4 |
| FANCM | -0.82996 | 0.000296 | 0.005206 | CA4 |
| CYB5R3 | 0.744835 | 0.000298 | 0.005214 | CA4 |
| CCP110 | -0.8244 | 0.000298 | 0.005214 | CA4 |
| RANGRF | 0.613643 | 0.000298 | 0.005214 | CA4 |
| PLAAT4 | 0.975035 | 0.000299 | 0.005214 | CA4 |
| TMEM151A | 0.771278 | 0.000298 | 0.005214 | CA4 |
| C16orf91 | 0.816416 | 0.0003 | 0.00522 | CA4 |
| S100A13 | 0.693214 | 0.000302 | 0.005248 | CA4 |
| TRPM4 | 0.763506 | 0.000304 | 0.005265 | CA4 |
| ACSF2 | 0.60362 | 0.000308 | 0.005326 | CA4 |
| UGGT2 | -0.63274 | 0.000309 | 0.005333 | CA4 |
| ZNF138 | -0.91333 | 0.000309 | 0.005333 | CA4 |
| CDH23 | 0.81483 | 0.000315 | 0.005411 | CA4 |
| PPP4R2 | -0.73601 | 0.000316 | 0.005414 | CA4 |
| ARHGAP21 | -0.69538 | 0.000319 | 0.00545 | CA4 |
| SLC49A3 | 0.869514 | 0.000319 | 0.005452 | CA4 |
| DAPK3 | 0.683045 | 0.000325 | 0.005539 | CA4 |
| EMP3 | 0.987303 | 0.000326 | 0.005555 | CA4 |
| PYCR2 | 0.645612 | 0.000328 | 0.005566 | CA4 |
| ZNF624 | -0.83383 | 0.000332 | 0.00561 | CA4 |
| CYLD | -0.69074 | 0.000336 | 0.005675 | CA4 |
| KLF2 | 1.363737 | 0.000336 | 0.005675 | CA4 |
| ZNF225 | -0.71592 | 0.000337 | 0.005685 | CA4 |
| HMG20B | 0.670862 | 0.000339 | 0.005701 | CA4 |

| | | | | |
|---|---|---|---|---|
| MRPL28 | 0.635698 | 0.000339 | 0.005701 | CA4 |
| SLC16A11 | 0.942803 | 0.00034 | 0.005706 | CA4 |
| SLC38A3 | 0.82835 | 0.000341 | 0.005721 | CA4 |
| PGAM1 | 0.618309 | 0.000343 | 0.005744 | CA4 |
| HCFC1R1 | 0.659588 | 0.000348 | 0.005804 | CA4 |
| INTS7 | -0.58718 | 0.000353 | 0.005869 | CA4 |
| DEDD2 | 0.657918 | 0.000355 | 0.005885 | CA4 |
| TPRKB | -0.62266 | 0.000356 | 0.00589 | CA4 |
| POM121 | 0.607044 | 0.000357 | 0.00589 | CA4 |
| CKB | 0.656711 | 0.000357 | 0.005891 | CA4 |
| LPAR5 | 0.887021 | 0.000359 | 0.005916 | CA4 |
| TKT | 0.656271 | 0.000362 | 0.005951 | CA4 |
| ITGB7 | 1.498813 | 0.000363 | 0.005951 | CA4 |
| IL1R2 | 3.216384 | 0.000364 | 0.005962 | CA4 |
| PHPT1 | 0.710874 | 0.000365 | 0.005967 | CA4 |
| FLNA | 0.888721 | 0.000365 | 0.005967 | CA4 |
| ZBTB45 | 0.613049 | 0.000365 | 0.005971 | CA4 |
| NFATC1 | 0.752188 | 0.000367 | 0.005986 | CA4 |
| EIF5B | -0.59444 | 0.000374 | 0.006067 | CA4 |
| LAGE3 | 0.857742 | 0.000372 | 0.006067 | CA4 |
| TAS2R19 | -1.04517 | 0.000373 | 0.006067 | CA4 |
| TWF2 | 0.632176 | 0.000374 | 0.006067 | CA4 |
| ZNF579 | 0.686108 | 0.000376 | 0.006085 | CA4 |
| HLA-DRB5 | 2.653059 | 0.000381 | 0.006159 | CA4 |
| LNPK | -0.6494 | 0.000382 | 0.00617 | CA4 |
| SIPA1 | 0.58533 | 0.000382 | 0.00617 | CA4 |
| ZNF587B | -0.64985 | 0.000383 | 0.006178 | CA4 |
| HSD17B10 | 0.765237 | 0.000384 | 0.006186 | CA4 |
| PGGT1B | -0.6122 | 0.000388 | 0.006242 | CA4 |
| YIPF4 | -0.65198 | 0.000392 | 0.006271 | CA4 |
| CENPC | -0.65079 | 0.000393 | 0.006288 | CA4 |
| MRPS24 | 0.77567 | 0.000395 | 0.006304 | CA4 |
| NAPG | -0.73087 | 0.000399 | 0.006352 | CA4 |
| NUDT16 | -1.61638 | 0.0004 | 0.006363 | CA4 |
| H4C8 | 0.704969 | 0.000403 | 0.006394 | CA4 |
| SEPTIN9 | 0.86753 | 0.000403 | 0.006394 | CA4 |
| ZNF511 | 0.664083 | 0.000409 | 0.006471 | CA4 |
| DLG1 | -0.67495 | 0.000413 | 0.006524 | CA4 |
| MRPL4 | 0.691739 | 0.000414 | 0.006532 | CA4 |
| SHISA4 | 0.697091 | 0.000414 | 0.006532 | CA4 |
| FGD3 | 0.849123 | 0.000423 | 0.006659 | CA4 |
| CFAP44 | -0.656 | 0.000429 | 0.006743 | CA4 |
| ZNF781 | -0.78314 | 0.000431 | 0.006759 | CA4 |
| YJU2 | 0.590917 | 0.000434 | 0.006797 | CA4 |

| | | | | |
|---|---|---|---|---|
| RAI2 | 0.727489 | 0.000437 | 0.006833 | CA4 |
| CLTB | 0.655921 | 0.000438 | 0.00684 | CA4 |
| TUBA1C | 0.712066 | 0.000441 | 0.006892 | CA4 |
| NAGLU | 0.763958 | 0.000443 | 0.006904 | CA4 |
| UFM1 | -0.59273 | 0.00045 | 0.006998 | CA4 |
| MT-ND6 | -1.26705 | 0.000453 | 0.007018 | CA4 |
| TUBA1B | 0.814943 | 0.000456 | 0.007063 | CA4 |
| LIPT1 | -0.93507 | 0.000461 | 0.007102 | CA4 |
| UBALD2 | 0.675349 | 0.000461 | 0.007102 | CA4 |
| ATP6V0C | 0.686787 | 0.000463 | 0.007117 | CA4 |
| APC2 | 0.627687 | 0.000469 | 0.007179 | CA4 |
| GNPDA2 | -0.63484 | 0.000469 | 0.007179 | CA4 |
| ATG4C | -0.82465 | 0.000473 | 0.007209 | CA4 |
| SMOX | 0.682556 | 0.000476 | 0.007234 | CA4 |
| NDUFA7 | 0.800383 | 0.000479 | 0.007277 | CA4 |
| SH2D3C | 0.654221 | 0.000481 | 0.007286 | CA4 |
| LLGL1 | 0.631122 | 0.000483 | 0.007297 | CA4 |
| METRN | 0.937612 | 0.000484 | 0.00731 | CA4 |
| LIN7B | 0.765966 | 0.000486 | 0.007335 | CA4 |
| FAM241A | -0.83623 | 0.000489 | 0.007357 | CA4 |
| ZNF638 | -0.67132 | 0.000491 | 0.007381 | CA4 |
| IGSF9B | 1.227174 | 0.000492 | 0.007386 | CA4 |
| SIGMAR1 | 0.589342 | 0.000493 | 0.007386 | CA4 |
| AC026954.2 | 2.856751 | 0.000494 | 0.007395 | CA4 |
| ST6GALNAC4 | 0.645396 | 0.000495 | 0.007403 | CA4 |
| EPN1 | 0.584866 | 0.000498 | 0.007417 | CA4 |
| RPS6KA4 | 0.635217 | 0.000499 | 0.007417 | CA4 |
| PLK3 | 1.272791 | 0.000499 | 0.007417 | CA4 |
| MRPL12 | 0.884114 | 0.000501 | 0.007434 | CA4 |
| CPNE6 | 0.680739 | 0.000502 | 0.007442 | CA4 |
| VWA1 | 0.996784 | 0.000504 | 0.007469 | CA4 |
| ERO1B | -0.6452 | 0.000508 | 0.007501 | CA4 |
| SLC66A2 | 0.660425 | 0.000507 | 0.007501 | CA4 |
| MID2 | 0.687269 | 0.00051 | 0.007523 | CA4 |
| RAB3IP | -0.73908 | 0.000511 | 0.007531 | CA4 |
| NOSIP | 0.623054 | 0.000512 | 0.007531 | CA4 |
| RAB4B-EGLN2 | 0.923548 | 0.000512 | 0.007531 | CA4 |
| H4C6 | 1.427041 | 0.000513 | 0.007535 | CA4 |
| RAC2 | 1.276907 | 0.000516 | 0.007542 | CA4 |
| NTN1 | 0.754358 | 0.000518 | 0.007546 | CA4 |
| PMVK | 0.633335 | 0.000517 | 0.007546 | CA4 |
| SCAI | -0.67236 | 0.000519 | 0.007557 | CA4 |
| PER2 | 0.679674 | 0.000526 | 0.007652 | CA4 |
| SLC25A39 | 0.60365 | 0.000527 | 0.007654 | CA4 |

| | | | | |
|---|---|---|---|---|
| CERS1 | 0.791166 | 0.000531 | 0.007681 | CA4 |
| TWF1 | -0.78072 | 0.000532 | 0.007685 | CA4 |
| BCKDHA | 0.661154 | 0.000532 | 0.007685 | CA4 |
| RGPD5 | -0.60511 | 0.000535 | 0.007719 | CA4 |
| SPAG7 | 0.623784 | 0.000537 | 0.007719 | CA4 |
| ZNF423 | 0.750319 | 0.000537 | 0.007719 | CA4 |
| HSPB1 | 1.277374 | 0.000537 | 0.007719 | CA4 |
| RPS21 | 0.685428 | 0.000536 | 0.007719 | CA4 |
| SHTN1 | -0.97588 | 0.000538 | 0.007722 | CA4 |
| AP5M1 | -0.5939 | 0.000539 | 0.007739 | CA4 |
| ATP6V0E1 | 0.765972 | 0.000542 | 0.007773 | CA4 |
| SCRIB | 0.580614 | 0.000544 | 0.007783 | CA4 |
| LRRC28 | -0.59573 | 0.000545 | 0.007788 | CA4 |
| EVC2 | 0.695142 | 0.000546 | 0.007788 | CA4 |
| FXYD1 | 0.814488 | 0.000545 | 0.007788 | CA4 |
| CTDSPL2 | -0.63044 | 0.000549 | 0.007825 | CA4 |
| LUC7L3 | -0.67599 | 0.000551 | 0.007842 | CA4 |
| ID3 | 0.76121 | 0.000555 | 0.007881 | CA4 |
| MICU3 | -0.76054 | 0.00056 | 0.007935 | CA4 |
| TMEM11 | 0.590986 | 0.000563 | 0.007958 | CA4 |
| ZNF33A | -0.80482 | 0.000564 | 0.007968 | CA4 |
| RWDD1 | -0.6802 | 0.000566 | 0.007973 | CA4 |
| AC073896.1 | 1.133921 | 0.000565 | 0.007973 | CA4 |
| MZT1 | -0.84548 | 0.000566 | 0.007973 | CA4 |
| SLC16A13 | 0.993088 | 0.00057 | 0.008011 | CA4 |
| SWT1 | -0.63037 | 0.000573 | 0.008042 | CA4 |
| C4orf3 | -0.78119 | 0.000575 | 0.008051 | CA4 |
| RGL3 | 0.672456 | 0.000575 | 0.008051 | CA4 |
| H2BC15 | 0.977208 | 0.000575 | 0.008051 | CA4 |
| KCNT2 | -0.7848 | 0.000576 | 0.00806 | CA4 |
| INTS1 | 0.759682 | 0.000577 | 0.008064 | CA4 |
| ZNF519 | -0.91367 | 0.00058 | 0.008067 | CA4 |
| BOLA1 | 0.839436 | 0.000578 | 0.008067 | CA4 |
| TCTE3 | -0.93385 | 0.00058 | 0.008067 | CA4 |
| CFAP54 | -0.86423 | 0.000579 | 0.008067 | CA4 |
| UQCC3 | 0.790111 | 0.000579 | 0.008067 | CA4 |
| CEP162 | -0.75762 | 0.000582 | 0.008078 | CA4 |
| MRPS2 | 0.841428 | 0.000586 | 0.008126 | CA4 |
| ROCK2 | -0.6264 | 0.000586 | 0.008126 | CA4 |
| PLIN4 | 0.881379 | 0.000589 | 0.008159 | CA4 |
| TMX3 | -0.6794 | 0.00059 | 0.008168 | CA4 |
| BCL6 | 0.793763 | 0.000592 | 0.008188 | CA4 |
| KCTD9 | -0.61192 | 0.000595 | 0.008194 | CA4 |
| FASTKD2 | -0.61493 | 0.000595 | 0.008194 | CA4 |

| | | | | |
|---|---|---|---|---|
| SEPTIN1 | 0.891883 | 0.00061 | 0.008347 | CA4 |
| ADCY7 | 0.672447 | 0.000615 | 0.008393 | CA4 |
| PHGDH | 0.766086 | 0.000615 | 0.008399 | CA4 |
| NR2F1 | 0.724788 | 0.000618 | 0.008428 | CA4 |
| PALM3 | 1.203275 | 0.00062 | 0.00844 | CA4 |
| TIMM29 | 0.605576 | 0.000629 | 0.008531 | CA4 |
| ZNF865 | 0.628852 | 0.000629 | 0.008531 | CA4 |
| VAMP4 | -0.68012 | 0.000637 | 0.008596 | CA4 |
| DDT | 0.632177 | 0.000643 | 0.008646 | CA4 |
| FHL3 | 0.688041 | 0.000642 | 0.008646 | CA4 |
| CTXN1 | 0.843323 | 0.000644 | 0.008651 | CA4 |
| SGCB | -0.66343 | 0.000649 | 0.008704 | CA4 |
| CTF1 | 0.998612 | 0.00066 | 0.008837 | CA4 |
| TPP1 | 0.662558 | 0.000663 | 0.008867 | CA4 |
| RPP21 | 0.817989 | 0.000663 | 0.008867 | CA4 |
| DCTPP1 | 0.630762 | 0.000671 | 0.008953 | CA4 |
| ELOB | 0.601721 | 0.000677 | 0.009022 | CA4 |
| LINGO1 | 0.940884 | 0.000679 | 0.009028 | CA4 |
| LINGO3 | 1.132581 | 0.000682 | 0.009052 | CA4 |
| NME5 | -0.81563 | 0.00069 | 0.009107 | CA4 |
| ISG15 | 1.098037 | 0.000692 | 0.009115 | CA4 |
| TAF1D | -0.58288 | 0.000696 | 0.009164 | CA4 |
| PYROXD2 | 0.591956 | 0.000702 | 0.009223 | CA4 |
| TCTEX1D2 | -0.62047 | 0.000709 | 0.009281 | CA4 |
| THUMPD2 | -0.59659 | 0.000711 | 0.00929 | CA4 |
| OSGIN1 | 1.209024 | 0.000711 | 0.00929 | CA4 |
| LIN7C | -0.8398 | 0.000714 | 0.009324 | CA4 |
| GSE1 | 0.618238 | 0.000719 | 0.009375 | CA4 |
| CPTP | 0.665403 | 0.000719 | 0.009375 | CA4 |
| PHOSPHO2 | -0.78936 | 0.00072 | 0.009381 | CA4 |
| SARDH | 0.775468 | 0.000725 | 0.009421 | CA4 |
| CEP126 | -0.64729 | 0.000729 | 0.009465 | CA4 |
| DTX2 | 0.643497 | 0.000732 | 0.009481 | CA4 |
| SH3GL3 | -0.61012 | 0.000736 | 0.009513 | CA4 |
| PLD1 | -0.65973 | 0.000737 | 0.009514 | CA4 |
| EPOR | 0.717192 | 0.000741 | 0.00956 | CA4 |
| ABHD13 | -0.64916 | 0.000753 | 0.009649 | CA4 |
| RPL11 | 0.634484 | 0.000758 | 0.009692 | CA4 |
| BBC3 | 0.666715 | 0.000759 | 0.009699 | CA4 |
| TCF7 | 0.777173 | 0.000762 | 0.009728 | CA4 |
| POP7 | 0.767631 | 0.000764 | 0.009743 | CA4 |
| ARHGDIB | 1.112624 | 0.000769 | 0.009791 | CA4 |
| FOXN2 | -0.70548 | 0.000772 | 0.009804 | CA4 |
| NXT1 | 0.800422 | 0.000773 | 0.009808 | CA4 |

| | | | | |
|---|---|---|---|---|
| RBM48 | -0.66072 | 0.000779 | 0.009855 | CA4 |
| SELPLG | 0.980085 | 0.00078 | 0.00987 | CA4 |
| PODNL1 | 1.060656 | 0.000783 | 0.009881 | CA4 |
| TIMM13 | 0.60721 | 0.000786 | 0.009904 | CA4 |
| ZDHHC21 | -0.60563 | 0.000787 | 0.009904 | CA4 |
| OMA1 | -0.60129 | 0.000793 | 0.009958 | CA4 |
| REEP4 | 0.859911 | 0.000795 | 0.009958 | CA4 |
| PLGLB1 | -0.98451 | 0.000795 | 0.009958 | CA4 |
| PLTP | 0.804374 | 0.000808 | 0.01004 | CA4 |
| RTN4R | 1.033412 | 0.000821 | 0.010151 | CA4 |
| MADCAM1 | 1.126776 | 0.000825 | 0.010171 | CA4 |
| PLXNA3 | 0.613505 | 0.000826 | 0.010171 | CA4 |
| SNAPC3 | -0.59376 | 0.000826 | 0.010171 | CA4 |
| HLA-DQB1 | 2.106509 | 0.000828 | 0.010187 | CA4 |
| MMP28 | 0.79248 | 0.00083 | 0.010205 | CA4 |
| AFTPH | -0.64176 | 0.000836 | 0.010266 | CA4 |
| DUS3L | 0.662792 | 0.000837 | 0.010268 | CA4 |
| LRWD1 | 0.663988 | 0.000838 | 0.010269 | CA4 |
| MSI1 | 0.647556 | 0.000845 | 0.010319 | CA4 |
| TDO2 | -1.37323 | 0.000844 | 0.010319 | CA4 |
| CD81 | 0.736673 | 0.000846 | 0.010322 | CA4 |
| ADRB2 | 0.698673 | 0.000847 | 0.010325 | CA4 |
| MFAP4 | 1.381295 | 0.000853 | 0.010369 | CA4 |
| WDR34 | 0.682546 | 0.000853 | 0.01037 | CA4 |
| XKR8 | 0.653193 | 0.000866 | 0.010457 | CA4 |
| TSPO | 0.932299 | 0.000867 | 0.010464 | CA4 |
| NXPE2 | -1.24764 | 0.000867 | 0.010464 | CA4 |
| RPL13A | 0.656853 | 0.00087 | 0.010479 | CA4 |
| ERBB2 | 0.796341 | 0.000872 | 0.010481 | CA4 |
| KBTBD3 | -0.67469 | 0.000872 | 0.010481 | CA4 |
| SNRPC | 0.61562 | 0.000876 | 0.010511 | CA4 |
| AC046185.1 | 2.409302 | 0.000888 | 0.010614 | CA4 |
| PYCARD | 1.236172 | 0.00089 | 0.010629 | CA4 |
| CDKN2D | 0.585313 | 0.000895 | 0.010649 | CA4 |
| H1-2 | 0.799532 | 0.000895 | 0.010649 | CA4 |
| NDUFA2 | 0.708681 | 0.000896 | 0.010654 | CA4 |
| ZNF112 | -0.69493 | 0.0009 | 0.010654 | CA4 |
| NIPSNAP3B | -0.78742 | 0.000898 | 0.010654 | CA4 |
| GPT | 0.838199 | 0.000898 | 0.010654 | CA4 |
| LOXL1 | 1.132632 | 0.000902 | 0.010667 | CA4 |
| ANKRD49 | -0.71449 | 0.000905 | 0.010683 | CA4 |
| FBXO6 | 0.618908 | 0.000906 | 0.010687 | CA4 |
| ITGAM | 0.80372 | 0.000906 | 0.010687 | CA4 |
| SAMD12 | -0.94962 | 0.000914 | 0.010724 | CA4 |

| | | | | |
|---|---|---|---|---|
| ERO1A | -0.67607 | 0.000925 | 0.010805 | CA4 |
| MYDGF | 0.593689 | 0.000939 | 0.010912 | CA4 |
| MEST | -0.69882 | 0.000946 | 0.010938 | CA4 |
| ZSWIM9 | 0.613578 | 0.000946 | 0.010938 | CA4 |
| ATP11B | -0.59786 | 0.000949 | 0.010947 | CA4 |
| H2AC18 | 0.850921 | 0.000951 | 0.010958 | CA4 |
| PCDH9 | -0.583 | 0.000953 | 0.010975 | CA4 |
| PSENEN | 0.637819 | 0.000956 | 0.010991 | CA4 |
| MAGEF1 | 0.703598 | 0.00097 | 0.011104 | CA4 |
| C11orf98 | 1.038994 | 0.000972 | 0.011104 | CA4 |
| POLR3GL | 0.614194 | 0.000986 | 0.011214 | CA4 |
| ZFYVE16 | -0.78228 | 0.000989 | 0.011233 | CA4 |
| POLI | -0.62337 | 0.000997 | 0.011304 | CA4 |
| AKAP9 | -0.74884 | 0.001003 | 0.011369 | CA4 |
| COMTD1 | 0.73379 | 0.001014 | 0.011464 | CA4 |
| C9orf16 | 0.763749 | 0.001023 | 0.011538 | CA4 |
| ZNF843 | 0.603406 | 0.001024 | 0.011551 | CA4 |
| TAS2R31 | -0.85513 | 0.001027 | 0.011563 | CA4 |
| RGS19 | 0.874587 | 0.001028 | 0.011571 | CA4 |
| FAM177A1 | -0.67668 | 0.001032 | 0.011592 | CA4 |
| CHST14 | 0.763263 | 0.001039 | 0.011666 | CA4 |
| FAM89B | 0.673002 | 0.001041 | 0.011675 | CA4 |
| ARAP2 | -0.67102 | 0.001048 | 0.011714 | CA4 |
| ZNF260 | -0.67228 | 0.001049 | 0.011717 | CA4 |
| LYRM7 | -0.60136 | 0.001068 | 0.011882 | CA4 |
| SURF2 | 0.644341 | 0.001074 | 0.011932 | CA4 |
| MIS18BP1 | -0.81556 | 0.001098 | 0.012144 | CA4 |
| TRAM1L1 | -0.68559 | 0.0011 | 0.012155 | CA4 |
| MDH2 | 0.593252 | 0.00111 | 0.012233 | CA4 |
| FGFR3 | 0.746927 | 0.001124 | 0.012346 | CA4 |
| NKIRAS1 | -0.62738 | 0.001126 | 0.012364 | CA4 |
| FAM171A2 | 0.751212 | 0.001131 | 0.012388 | CA4 |
| FOXD1 | 1.01035 | 0.001131 | 0.012388 | CA4 |
| RESF1 | -0.99298 | 0.001133 | 0.012389 | CA4 |
| KLHL25 | 0.647678 | 0.001133 | 0.012389 | CA4 |
| SVIP | -0.63973 | 0.001133 | 0.012389 | CA4 |
| CHIC1 | -0.68954 | 0.001137 | 0.012421 | CA4 |
| DOLK | 0.670677 | 0.001147 | 0.012495 | CA4 |
| RPUSD1 | 0.616917 | 0.00115 | 0.012508 | CA4 |
| ZNF493 | -0.69034 | 0.001155 | 0.012549 | CA4 |
| HNRNPLL | -0.58333 | 0.001162 | 0.012591 | CA4 |
| COX6A1 | 0.654026 | 0.001163 | 0.012594 | CA4 |
| JUND | 0.628216 | 0.00117 | 0.012626 | CA4 |
| MXRA8 | 0.734335 | 0.00117 | 0.012626 | CA4 |

| | | | | |
|---|---|---|---|---|
| MEX3D | 0.748816 | 0.001178 | 0.012686 | CA4 |
| DTWD1 | -0.84511 | 0.001179 | 0.012689 | CA4 |
| ID1 | 0.81857 | 0.001193 | 0.012795 | CA4 |
| ANKRD36B | -0.72482 | 0.001193 | 0.012795 | CA4 |
| CDK2AP2 | 0.77754 | 0.001206 | 0.012909 | CA4 |
| FASTKD3 | -0.73552 | 0.001208 | 0.012913 | CA4 |
| SOX2 | 0.654613 | 0.001208 | 0.012913 | CA4 |
| TRADD | 0.639953 | 0.001211 | 0.012939 | CA4 |
| METRNL | 0.739718 | 0.001217 | 0.012987 | CA4 |
| ARX | 0.768057 | 0.001226 | 0.013056 | CA4 |
| BGN | 0.695381 | 0.001228 | 0.013065 | CA4 |
| ACY3 | 1.847095 | 0.001241 | 0.013168 | CA4 |
| SGSH | 0.632872 | 0.001247 | 0.013209 | CA4 |
| CCDC88A | -0.64577 | 0.001248 | 0.013209 | CA4 |
| DYNC1LI2 | -0.82595 | 0.001249 | 0.013211 | CA4 |
| RAMP2 | 1.084403 | 0.001254 | 0.013257 | CA4 |
| GPSM1 | 0.642103 | 0.001261 | 0.013312 | CA4 |
| RPGRIP1L | -0.58388 | 0.001265 | 0.013325 | CA4 |
| VSIR | 0.712684 | 0.001266 | 0.013332 | CA4 |
| YIPF6 | -1.11815 | 0.001287 | 0.013485 | CA4 |
| INHBB | 0.73575 | 0.001292 | 0.013505 | CA4 |
| C5orf22 | -0.60986 | 0.001297 | 0.013538 | CA4 |
| DHRS4L2 | 0.77529 | 0.001305 | 0.013622 | CA4 |
| CENPJ | -0.6371 | 0.00132 | 0.013725 | CA4 |
| S100A6 | 0.643839 | 0.00132 | 0.013725 | CA4 |
| TAS2R46 | -1.2722 | 0.001322 | 0.013736 | CA4 |
| DIAPH2 | -0.78363 | 0.001328 | 0.013785 | CA4 |
| SKA2 | -0.684 | 0.001342 | 0.013903 | CA4 |
| TMEM117 | -0.62438 | 0.001358 | 0.014007 | CA4 |
| C4B | 1.254769 | 0.001361 | 0.014032 | CA4 |
| HLA-E | 0.779185 | 0.001372 | 0.014125 | CA4 |
| SLC39A1 | 0.678814 | 0.001377 | 0.014155 | CA4 |
| RHBDF1 | 0.629189 | 0.001386 | 0.014211 | CA4 |
| ZNF578 | -0.71137 | 0.001386 | 0.014211 | CA4 |
| PSAP | 0.635914 | 0.001392 | 0.014244 | CA4 |
| DDX11 | 1.191469 | 0.001402 | 0.014313 | CA4 |
| DNAJB14 | -0.62491 | 0.001406 | 0.014327 | CA4 |
| AC008758.1 | 4.792066 | 0.001405 | 0.014327 | CA4 |
| CPZ | 2.793697 | 0.00141 | 0.01435 | CA4 |
| H3-2 | 1.485879 | 0.001432 | 0.014519 | CA4 |
| ADGRA2 | 0.586094 | 0.001439 | 0.014574 | CA4 |
| TMEM170B | -0.68531 | 0.001458 | 0.014722 | CA4 |
| PLD3 | 0.697624 | 0.001465 | 0.014766 | CA4 |
| CASKIN2 | 0.618486 | 0.001464 | 0.014766 | CA4 |

| | | | | |
|---|---|---|---|---|
| GLI3 | 0.625906 | 0.001468 | 0.014783 | CA4 |
| PLEKHA1 | -0.64456 | 0.001471 | 0.014783 | CA4 |
| FBXO44 | 0.679695 | 0.001476 | 0.014819 | CA4 |
| FSIP1 | -0.67354 | 0.001476 | 0.014819 | CA4 |
| MAP2K3 | 0.61791 | 0.001483 | 0.014882 | CA4 |
| EEF1AKMT2 | -0.66312 | 0.001486 | 0.014901 | CA4 |
| TRMT13 | -0.76219 | 0.001496 | 0.014953 | CA4 |
| RPAP2 | -0.62639 | 0.001497 | 0.014953 | CA4 |
| PLD4 | 1.043981 | 0.00152 | 0.015148 | CA4 |
| GIN1 | -0.59299 | 0.001531 | 0.015231 | CA4 |
| C1orf35 | 0.761199 | 0.001534 | 0.015239 | CA4 |
| AXL | 0.608622 | 0.001536 | 0.015254 | CA4 |
| TCF20 | -1.03087 | 0.001541 | 0.015271 | CA4 |
| IGSF9 | 0.955834 | 0.00155 | 0.015327 | CA4 |
| TRIB3 | 0.81722 | 0.001555 | 0.015359 | CA4 |
| EXOSC4 | 0.653904 | 0.001558 | 0.01538 | CA4 |
| ZP3 | 0.872163 | 0.001574 | 0.015484 | CA4 |
| MYLK | 0.634788 | 0.001595 | 0.015654 | CA4 |
| EFCAB7 | -0.64907 | 0.001599 | 0.015684 | CA4 |
| RPL35 | 0.582947 | 0.001606 | 0.015702 | CA4 |
| ENPP4 | -0.78901 | 0.00162 | 0.015791 | CA4 |
| MRPS34 | 0.651219 | 0.001626 | 0.015834 | CA4 |
| UCKL1 | 0.605321 | 0.001629 | 0.015853 | CA4 |
| ZNHIT2 | 0.722618 | 0.00163 | 0.015858 | CA4 |
| SLC27A3 | 0.698986 | 0.001651 | 0.016029 | CA4 |
| RPUSD2 | 0.597492 | 0.001654 | 0.016043 | CA4 |
| AP2S1 | 0.677207 | 0.001655 | 0.016045 | CA4 |
| MELTF | 0.693158 | 0.001659 | 0.01605 | CA4 |
| BTBD19 | 1.221944 | 0.00166 | 0.016051 | CA4 |
| ELAPOR2 | -0.63809 | 0.001662 | 0.016065 | CA4 |
| PRXL2C | -0.68164 | 0.001666 | 0.016078 | CA4 |
| CCL5 | 2.130606 | 0.001666 | 0.016078 | CA4 |
| IRAK4 | -0.75635 | 0.001668 | 0.016087 | CA4 |
| PRIM2 | -0.79396 | 0.001674 | 0.016137 | CA4 |
| HSPB2 | 0.700091 | 0.001682 | 0.016192 | CA4 |
| COPS9 | 0.62089 | 0.001685 | 0.016192 | CA4 |
| ZNF317 | 0.591372 | 0.001692 | 0.016243 | CA4 |
| TAS1R3 | 1.357505 | 0.001696 | 0.016273 | CA4 |
| SMC4 | -0.63055 | 0.001716 | 0.016403 | CA4 |
| SYCP2 | -0.67142 | 0.001715 | 0.016403 | CA4 |
| TSGA10 | -0.69513 | 0.001719 | 0.01643 | CA4 |
| PSMB9 | 0.809342 | 0.001726 | 0.016466 | CA4 |
| PRELP | 0.772878 | 0.001736 | 0.016542 | CA4 |
| SLC39A4 | 0.881496 | 0.001738 | 0.01655 | CA4 |

| | | | | |
|---|---|---|---|---|
| BTBD8 | -0.66773 | 0.00175 | 0.016613 | CA4 |
| EFNB1 | 0.626085 | 0.001764 | 0.016724 | CA4 |
| SPCS3 | -0.62886 | 0.001772 | 0.016762 | CA4 |
| FGFR4 | 0.867428 | 0.001774 | 0.016767 | CA4 |
| CREB1 | -0.83246 | 0.001779 | 0.016789 | CA4 |
| RPF2 | -0.64122 | 0.001782 | 0.016796 | CA4 |
| ZNF322 | -0.84561 | 0.001785 | 0.016822 | CA4 |
| MMP11 | 0.958688 | 0.001793 | 0.016855 | CA4 |
| SFRP2 | 0.939328 | 0.001795 | 0.016855 | CA4 |
| PANK1 | -0.74577 | 0.001824 | 0.017045 | CA4 |
| ACTG1 | 0.670748 | 0.001826 | 0.01705 | CA4 |
| REX1BD | 0.617312 | 0.001833 | 0.017089 | CA4 |
| ZFP37 | -0.68127 | 0.001852 | 0.01723 | CA4 |
| LRRC3 | 0.739322 | 0.001874 | 0.017404 | CA4 |
| RAB3IL1 | 0.603693 | 0.001878 | 0.017409 | CA4 |
| ACP5 | 1.389751 | 0.00188 | 0.017421 | CA4 |
| RPS6KA6 | -0.71052 | 0.001884 | 0.01743 | CA4 |
| HLA-B | 0.944391 | 0.001906 | 0.017614 | CA4 |
| CLU | 0.859064 | 0.001908 | 0.017617 | CA4 |
| ARPC1B | 0.630515 | 0.001912 | 0.017646 | CA4 |
| MIPOL1 | -0.62389 | 0.001923 | 0.01767 | CA4 |
| SLC43A2 | 0.771319 | 0.001922 | 0.01767 | CA4 |
| RHOG | 0.813973 | 0.001928 | 0.01767 | CA4 |
| MCF2 | -0.86075 | 0.001941 | 0.017761 | CA4 |
| SNX16 | -0.61648 | 0.001941 | 0.017761 | CA4 |
| PTP4A2 | -0.77401 | 0.001939 | 0.017761 | CA4 |
| FBXL7 | 0.62574 | 0.00196 | 0.017887 | CA4 |
| THAP8 | 0.627186 | 0.001963 | 0.017903 | CA4 |
| AC233723.1 | 1.564317 | 0.001964 | 0.017903 | CA4 |
| PTRHD1 | 0.626441 | 0.001969 | 0.01794 | CA4 |
| THAP9 | -0.95005 | 0.001975 | 0.017981 | CA4 |
| LRRC8A | 0.601199 | 0.001976 | 0.017983 | CA4 |
| AC011005.1 | 0.953306 | 0.001984 | 0.018021 | CA4 |
| TMEM265 | -1.61418 | 0.002016 | 0.018296 | CA4 |
| SPI1 | 0.945063 | 0.002038 | 0.018422 | CA4 |
| FBXO4 | -0.60513 | 0.002037 | 0.018422 | CA4 |
| FZD9 | 0.895518 | 0.002056 | 0.01853 | CA4 |
| ASTN2 | 0.613431 | 0.00206 | 0.01855 | CA4 |
| RAD51AP2 | -2.0109 | 0.00206 | 0.01855 | CA4 |
| CENPE | -1.04899 | 0.002093 | 0.018738 | CA4 |
| PRKAA2 | -0.6011 | 0.002111 | 0.01885 | CA4 |
| CRTAC1 | 0.908663 | 0.002113 | 0.018854 | CA4 |
| B3GALT2 | -0.69748 | 0.00212 | 0.01887 | CA4 |
| ZNF141 | -0.81477 | 0.002122 | 0.01888 | CA4 |

| | | | | |
|---|---|---|---|---|
| VAMP7 | -0.68979 | 0.002129 | 0.018915 | CA4 |
| FGFRL1 | 0.849305 | 0.002129 | 0.018915 | CA4 |
| DNAH6 | -0.74524 | 0.002144 | 0.01898 | CA4 |
| MTSS2 | 0.684149 | 0.002145 | 0.01898 | CA4 |
| H2AC20 | 0.652394 | 0.002147 | 0.01898 | CA4 |
| TMEM256 | 0.855838 | 0.002146 | 0.01898 | CA4 |
| CDC42EP1 | 0.860546 | 0.002152 | 0.018995 | CA4 |
| EIF4E | -0.85163 | 0.002152 | 0.018995 | CA4 |
| GAS2L1 | 0.68787 | 0.002173 | 0.019133 | CA4 |
| AC069368.1 | 1.368153 | 0.002179 | 0.019169 | CA4 |
| CCDC169 | -0.78128 | 0.002192 | 0.01922 | CA4 |
| TSR3 | 0.591157 | 0.002199 | 0.019242 | CA4 |
| BMT2 | -0.6982 | 0.002199 | 0.019242 | CA4 |
| FMNL1 | 0.719766 | 0.002196 | 0.019242 | CA4 |
| ZMYND15 | 0.89162 | 0.002208 | 0.019247 | CA4 |
| P2RY2 | 1.036577 | 0.002204 | 0.019247 | CA4 |
| TGFBR3L | 1.208723 | 0.002205 | 0.019247 | CA4 |
| IL2RB | 2.153526 | 0.002222 | 0.019329 | CA4 |
| RPL29 | 0.640221 | 0.002257 | 0.019496 | CA4 |
| ADPRM | -0.60583 | 0.002259 | 0.01951 | CA4 |
| TMEM30A | -0.59795 | 0.002262 | 0.019518 | CA4 |
| QPCTL | 0.580678 | 0.002267 | 0.019531 | CA4 |
| EEF1G | 0.805007 | 0.002268 | 0.019531 | CA4 |
| CD68 | 1.369098 | 0.002272 | 0.019546 | CA4 |
| PLAG1 | -0.61169 | 0.002273 | 0.019546 | CA4 |
| RAB9B | -0.7227 | 0.002291 | 0.019647 | CA4 |
| SOX15 | 1.120572 | 0.002295 | 0.019661 | CA4 |
| BBIP1 | -0.67 | 0.002321 | 0.019854 | CA4 |
| MED11 | 0.666399 | 0.002325 | 0.019865 | CA4 |
| SCAMP1 | -0.69224 | 0.002327 | 0.019872 | CA4 |
| SPDL1 | -0.82361 | 0.002336 | 0.019916 | CA4 |
| FOSL1 | 1.897815 | 0.002335 | 0.019916 | CA4 |
| FAM174C | 0.733172 | 0.002343 | 0.019953 | CA4 |
| CCDC7 | -0.68336 | 0.002361 | 0.020055 | CA4 |
| HLA-A | 0.799316 | 0.002371 | 0.020099 | CA4 |
| CFAP206 | -0.91973 | 0.002394 | 0.02026 | CA4 |
| TAS2R13 | -0.81483 | 0.002406 | 0.020344 | CA4 |
| NPM3 | 0.997707 | 0.00241 | 0.020353 | CA4 |
| GPR22 | -1.03057 | 0.002415 | 0.02039 | CA4 |
| FAM110A | 0.832225 | 0.002426 | 0.020463 | CA4 |
| MAPK8IP3 | 0.593743 | 0.002427 | 0.020463 | CA4 |
| TACO1 | 0.640717 | 0.002432 | 0.020466 | CA4 |
| D2HGDH | 0.591659 | 0.002437 | 0.020487 | CA4 |
| OPLAH | 0.715362 | 0.002443 | 0.020531 | CA4 |

| | | | | |
|---|---|---|---|---|
| CEP295 | -0.73151 | 0.002452 | 0.020583 | CA4 |
| DBF4 | -0.71258 | 0.00247 | 0.0207 | CA4 |
| G0S2 | 1.419364 | 0.002478 | 0.020742 | CA4 |
| RELT | 0.636408 | 0.00248 | 0.020754 | CA4 |
| FBXL14 | 0.706587 | 0.002511 | 0.020949 | CA4 |
| UNC93B1 | 0.591354 | 0.002515 | 0.020961 | CA4 |
| TTC23L | -0.82829 | 0.002515 | 0.020961 | CA4 |
| ABHD14A | 0.734906 | 0.002523 | 0.021018 | CA4 |
| SUSD3 | 1.183964 | 0.002534 | 0.021081 | CA4 |
| MSLN | 1.375284 | 0.002539 | 0.021114 | CA4 |
| GPX3 | 1.030037 | 0.002544 | 0.021122 | CA4 |
| MTBP | -0.86018 | 0.002565 | 0.021238 | CA4 |
| MYSM1 | -0.61017 | 0.002579 | 0.021316 | CA4 |
| WDR17 | -0.59794 | 0.002583 | 0.021337 | CA4 |
| NDUFAF5 | -0.62572 | 0.002587 | 0.021345 | CA4 |
| UBE2W | -0.77077 | 0.002587 | 0.021345 | CA4 |
| SERINC2 | 0.996152 | 0.002597 | 0.021398 | CA4 |
| PAX6 | 0.661867 | 0.002643 | 0.021651 | CA4 |
| PPP1R1B | 0.690323 | 0.002643 | 0.021651 | CA4 |
| FCGR3B | 2.050586 | 0.002648 | 0.021667 | CA4 |
| CAVIN4 | -0.71195 | 0.002656 | 0.021699 | CA4 |
| ZNF566 | -0.60195 | 0.002696 | 0.021915 | CA4 |
| HAGHL | 0.748462 | 0.002698 | 0.021924 | CA4 |
| NGFR | 1.439768 | 0.002701 | 0.021931 | CA4 |
| SLC22A6 | 1.512002 | 0.002722 | 0.022046 | CA4 |
| LYPLA1 | -0.62378 | 0.002756 | 0.022255 | CA4 |
| SH2B2 | 0.643061 | 0.002764 | 0.022288 | CA4 |
| SNX6 | -0.63823 | 0.002781 | 0.022417 | CA4 |
| LPIN3 | 0.749654 | 0.002801 | 0.022491 | CA4 |
| PGAM2 | 1.036572 | 0.00281 | 0.022536 | CA4 |
| ABCE1 | -0.61544 | 0.002811 | 0.022537 | CA4 |
| ZNF286A | -0.67475 | 0.002819 | 0.022552 | CA4 |
| BMP8A | 0.648319 | 0.002834 | 0.022641 | CA4 |
| VGLL3 | -1.21315 | 0.002833 | 0.022641 | CA4 |
| BDP1 | -0.61716 | 0.002847 | 0.022729 | CA4 |
| MARCHF7 | -0.59332 | 0.002857 | 0.022754 | CA4 |
| SLC26A1 | 0.855356 | 0.00286 | 0.022754 | CA4 |
| ODR4 | -0.5996 | 0.002861 | 0.022754 | CA4 |
| NEK5 | -0.9291 | 0.002855 | 0.022754 | CA4 |
| EEF1AKMT3 | -0.83801 | 0.002875 | 0.02282 | CA4 |
| PUS10 | -0.69543 | 0.002877 | 0.02282 | CA4 |
| PFKFB3 | 0.659491 | 0.002879 | 0.02282 | CA4 |
| H2AC4 | 1.380247 | 0.002885 | 0.022841 | CA4 |
| INKA1 | 0.888123 | 0.002901 | 0.022951 | CA4 |

| | | | | |
|---|---|---|---|---|
| JARID2 | 0.788952 | 0.002906 | 0.022979 | CA4 |
| CCDC181 | -0.69241 | 0.002922 | 0.02307 | CA4 |
| CRYAB | 0.916703 | 0.00295 | 0.023245 | CA4 |
| OPRL1 | 0.677834 | 0.002955 | 0.023249 | CA4 |
| RPL39L | 0.937112 | 0.002973 | 0.02331 | CA4 |
| IFI6 | 0.821668 | 0.002984 | 0.023359 | CA4 |
| MESP1 | 0.851198 | 0.002992 | 0.023399 | CA4 |
| KIAA0408 | -0.60825 | 0.002991 | 0.023399 | CA4 |
| CXADR | -0.74458 | 0.002996 | 0.023414 | CA4 |
| CADM4 | 0.586493 | 0.003027 | 0.023612 | CA4 |
| IL1RAPL1 | -0.72452 | 0.003036 | 0.02367 | CA4 |
| TTC14 | -0.58951 | 0.003046 | 0.023723 | CA4 |
| SCN4A | 1.092965 | 0.003048 | 0.023727 | CA4 |
| C16orf96 | 0.764993 | 0.003049 | 0.023727 | CA4 |
| DNPH1 | 0.625929 | 0.003055 | 0.023761 | CA4 |
| ZNF714 | -0.74354 | 0.003065 | 0.023817 | CA4 |
| KIF5B | -0.66611 | 0.003064 | 0.023817 | CA4 |
| C14orf28 | -0.6244 | 0.003077 | 0.023851 | CA4 |
| MPV17L2 | 0.681229 | 0.003081 | 0.02386 | CA4 |
| ASAH2B | -0.75308 | 0.003085 | 0.023862 | CA4 |
| DBI | 0.670863 | 0.003092 | 0.023903 | CA4 |
| LRRC34 | -0.75488 | 0.003101 | 0.023959 | CA4 |
| ZNF761 | -0.73751 | 0.003103 | 0.023961 | CA4 |
| GCK | 0.932908 | 0.00312 | 0.024001 | CA4 |
| RMDN2 | -0.66299 | 0.003144 | 0.024118 | CA4 |
| MAP9 | -0.71882 | 0.003143 | 0.024118 | CA4 |
| RNF208 | 0.69987 | 0.003143 | 0.024118 | CA4 |
| AKR7A3 | 0.594065 | 0.003155 | 0.024178 | CA4 |
| NDUFA6 | 0.593915 | 0.003161 | 0.024194 | CA4 |
| MOSPD3 | 0.590124 | 0.003174 | 0.024257 | CA4 |
| PLP2 | 0.872215 | 0.003177 | 0.024271 | CA4 |
| AGBL3 | -0.62222 | 0.00319 | 0.02433 | CA4 |
| SLC16A7 | -1.07214 | 0.003192 | 0.024336 | CA4 |
| ARF1 | 0.941097 | 0.003193 | 0.024336 | CA4 |
| CYP21A2 | 1.130812 | 0.003217 | 0.024479 | CA4 |
| ALPL | 0.840831 | 0.00323 | 0.024527 | CA4 |
| H3C4 | 0.794985 | 0.003243 | 0.024578 | CA4 |
| TEX45 | 1.120044 | 0.003243 | 0.024578 | CA4 |
| YWHAB | -0.67832 | 0.003249 | 0.024611 | CA4 |
| AC004997.1 | 0.869078 | 0.003273 | 0.024757 | CA4 |
| MBLAC1 | 0.758703 | 0.003274 | 0.024757 | CA4 |
| NME9 | -0.60715 | 0.003277 | 0.024767 | CA4 |
| DNAH12 | -1.02256 | 0.003285 | 0.024797 | CA4 |
| NDUFA12 | 0.678481 | 0.003295 | 0.024797 | CA4 |

| | | | | |
|---|---|---|---|---|
| C19orf38 | 0.869772 | 0.003295 | 0.024797 | CA4 |
| ORAI1 | 0.979491 | 0.003284 | 0.024797 | CA4 |
| C3orf33 | -0.64003 | 0.003301 | 0.024832 | CA4 |
| ZNF226 | -0.70046 | 0.003321 | 0.02491 | CA4 |
| TAS2R10 | -1.11279 | 0.00333 | 0.02497 | CA4 |
| RNF145 | -0.66287 | 0.003342 | 0.025023 | CA4 |
| SLC25A28 | 0.705413 | 0.003351 | 0.025067 | CA4 |
| TMEM184B | 0.608269 | 0.003357 | 0.025089 | CA4 |
| RBP1 | 0.697788 | 0.003362 | 0.0251 | CA4 |
| MAPK11 | 0.580303 | 0.003361 | 0.0251 | CA4 |
| PYROXD1 | -0.71805 | 0.003377 | 0.025177 | CA4 |
| PTRH1 | 0.5845 | 0.003404 | 0.025309 | CA4 |
| TAMALIN | 0.84638 | 0.00341 | 0.025331 | CA4 |
| NT5C3A | -0.64901 | 0.003416 | 0.025367 | CA4 |
| PDLIM4 | 0.714438 | 0.003427 | 0.025432 | CA4 |
| CD177 | 1.672451 | 0.003454 | 0.025569 | CA4 |
| CCDC175 | -0.93251 | 0.003475 | 0.025671 | CA4 |
| CCDC112 | -0.59099 | 0.003539 | 0.026075 | CA4 |
| PCMTD1 | -0.7589 | 0.003539 | 0.026075 | CA4 |
| FMOD | 1.345098 | 0.003542 | 0.026085 | CA4 |
| FAT2 | 2.34411 | 0.003577 | 0.026269 | CA4 |
| CCDC110 | -0.89695 | 0.003578 | 0.026269 | CA4 |
| TOM1L2 | 0.632771 | 0.003598 | 0.026389 | CA4 |
| LSP1 | 1.186699 | 0.003635 | 0.026599 | CA4 |
| FKRP | 1.048685 | 0.003633 | 0.026599 | CA4 |
| ZNF814 | -0.6155 | 0.00364 | 0.026616 | CA4 |
| THOC1 | -0.62103 | 0.003644 | 0.026618 | CA4 |
| CALY | 0.914119 | 0.003647 | 0.026624 | CA4 |
| FANCB | -0.97274 | 0.003681 | 0.026793 | CA4 |
| DNASE2 | 0.66178 | 0.003727 | 0.027067 | CA4 |
| PTPN6 | 0.890022 | 0.003823 | 0.02758 | CA4 |
| S100A10 | 0.956126 | 0.003866 | 0.027817 | CA4 |
| LMNA | 0.593395 | 0.00387 | 0.027834 | CA4 |
| TMEM187 | 0.669628 | 0.003873 | 0.027841 | CA4 |
| SLC22A18 | 1.088459 | 0.003915 | 0.028091 | CA4 |
| WDR62 | 0.866467 | 0.003951 | 0.028277 | CA4 |
| WTIP | 0.718567 | 0.003967 | 0.028346 | CA4 |
| VIM | 0.897237 | 0.003985 | 0.028395 | CA4 |
| ARL6 | -0.65726 | 0.003985 | 0.028395 | CA4 |
| ZNF672 | 1.02503 | 0.003991 | 0.028426 | CA4 |
| CBS | 0.726696 | 0.00401 | 0.028486 | CA4 |
| IMPG2 | -0.72241 | 0.004019 | 0.028509 | CA4 |
| KCTD16 | -0.91514 | 0.004018 | 0.028509 | CA4 |
| SRP19 | -0.61525 | 0.004025 | 0.028542 | CA4 |

| | | | | |
|---|---|---|---|---|
| BAIAP2L2 | 0.766644 | 0.004037 | 0.028578 | CA4 |
| TBC1D10C | 1.107855 | 0.004043 | 0.028606 | CA4 |
| SCRG1 | 0.631813 | 0.004054 | 0.028658 | CA4 |
| CEP135 | -0.59918 | 0.004059 | 0.028664 | CA4 |
| FAM47E | -0.66927 | 0.00406 | 0.028664 | CA4 |
| MCOLN3 | -1.11564 | 0.004083 | 0.02874 | CA4 |
| IGSF21 | 0.594998 | 0.004088 | 0.02874 | CA4 |
| PLSCR3 | 0.65687 | 0.004088 | 0.02874 | CA4 |
| CEND1 | 0.601826 | 0.004107 | 0.02882 | CA4 |
| PLEKHF1 | 0.743486 | 0.004137 | 0.028935 | CA4 |
| C4A | 1.022469 | 0.004145 | 0.028972 | CA4 |
| PATL2 | 1.214515 | 0.00416 | 0.029053 | CA4 |
| EGF | 0.862145 | 0.004194 | 0.029228 | CA4 |
| FAM227B | -0.67338 | 0.004218 | 0.029257 | CA4 |
| FAM83H | 0.583066 | 0.004214 | 0.029257 | CA4 |
| DPY19L2 | -0.70009 | 0.004248 | 0.029431 | CA4 |
| AEBP1 | 0.864903 | 0.004259 | 0.029493 | CA4 |
| GPRC5C | 0.618727 | 0.00427 | 0.029557 | CA4 |
| PAPLN | 0.748941 | 0.004276 | 0.029587 | CA4 |
| ABHD17B | -0.60223 | 0.004308 | 0.029715 | CA4 |
| TST | 0.66155 | 0.004321 | 0.029785 | CA4 |
| MALT1 | -0.63456 | 0.004323 | 0.029787 | CA4 |
| GOLT1B | -0.7133 | 0.004347 | 0.02991 | CA4 |
| ARL4D | 1.059905 | 0.004365 | 0.030008 | CA4 |
| HLA-DPA1 | 1.056571 | 0.004415 | 0.030261 | CA4 |
| ALDH1L1 | 0.601092 | 0.004443 | 0.030403 | CA4 |
| HLA-C | 0.739323 | 0.004463 | 0.030516 | CA4 |
| NDUFAF4 | -0.5874 | 0.004472 | 0.030556 | CA4 |
| IKBKE | 0.675752 | 0.004473 | 0.030556 | CA4 |
| PLPPR3 | 0.719204 | 0.004481 | 0.030572 | CA4 |
| LGALS9B | 1.398485 | 0.0045 | 0.03068 | CA4 |
| CDH4 | 0.589358 | 0.004534 | 0.030881 | CA4 |
| SORBS3 | 0.594367 | 0.004548 | 0.030952 | CA4 |
| MVP | 0.59133 | 0.004603 | 0.031156 | CA4 |
| DNAH5 | -0.69293 | 0.004606 | 0.031156 | CA4 |
| ACOT1 | 0.980337 | 0.004613 | 0.031187 | CA4 |
| RPL26L1 | 0.734068 | 0.004699 | 0.031621 | CA4 |
| FLT4 | 0.710366 | 0.00471 | 0.031659 | CA4 |
| SUSD2 | 1.057992 | 0.004712 | 0.031659 | CA4 |
| OAF | 0.716869 | 0.004715 | 0.031663 | CA4 |
| CORO1A | 0.581411 | 0.004728 | 0.031738 | CA4 |
| ZNF577 | -0.63221 | 0.004735 | 0.031757 | CA4 |
| CD37 | 0.651909 | 0.00475 | 0.031835 | CA4 |
| CBLN3 | 0.65337 | 0.004765 | 0.031893 | CA4 |

| | | | | |
|---|---|---|---|---|
| CSF1R | 0.718059 | 0.00477 | 0.031912 | CA4 |
| ZNF670 | -0.64964 | 0.004787 | 0.032 | CA4 |
| LINC00634 | 0.684604 | 0.004832 | 0.032244 | CA4 |
| ZNF527 | -0.78368 | 0.004839 | 0.032269 | CA4 |
| SPSB2 | 0.792121 | 0.004873 | 0.032427 | CA4 |
| GADD45GIP1 | 0.595234 | 0.004895 | 0.032547 | CA4 |
| FGF14 | -0.66484 | 0.004904 | 0.032565 | CA4 |
| ZNF35 | -0.68213 | 0.004905 | 0.032565 | CA4 |
| FAT4 | -0.59777 | 0.00491 | 0.032565 | CA4 |
| SPATA2L | 0.58535 | 0.004922 | 0.032594 | CA4 |
| TMEM132E | 0.689395 | 0.00492 | 0.032594 | CA4 |
| S100A4 | 1.345198 | 0.004938 | 0.032672 | CA4 |
| LCN12 | 0.889812 | 0.004981 | 0.032906 | CA4 |
| SOX18 | 1.353404 | 0.00501 | 0.033056 | CA4 |
| CLCA2 | -1.96058 | 0.005038 | 0.033162 | CA4 |
| MN1 | 0.601325 | 0.005036 | 0.033162 | CA4 |
| ATP1A2 | 0.637106 | 0.005047 | 0.033204 | CA4 |
| FLT3LG | 0.641758 | 0.005061 | 0.033261 | CA4 |
| NUDT1 | 0.713202 | 0.005109 | 0.033401 | CA4 |
| CATSPER2 | -0.78505 | 0.005103 | 0.033401 | CA4 |
| KIAA1586 | -0.80933 | 0.005138 | 0.033465 | CA4 |
| NPIPB4 | -0.88614 | 0.005149 | 0.033527 | CA4 |
| GPSM2 | -0.58366 | 0.005158 | 0.033545 | CA4 |
| CAMK4 | -0.66503 | 0.005157 | 0.033545 | CA4 |
| CLDND1 | -0.91677 | 0.005175 | 0.033628 | CA4 |
| CTSZ | 0.638829 | 0.005181 | 0.033652 | CA4 |
| MGAM | 0.918909 | 0.005183 | 0.033652 | CA4 |
| MIDN | 0.725912 | 0.005215 | 0.033843 | CA4 |
| PHF10 | -0.71044 | 0.005232 | 0.033929 | CA4 |
| SASS6 | -0.67332 | 0.005261 | 0.034064 | CA4 |
| S100A11 | 0.97966 | 0.005278 | 0.034148 | CA4 |
| PBXIP1 | 0.602791 | 0.005286 | 0.034167 | CA4 |
| BTF3L4 | -0.7122 | 0.005292 | 0.034184 | CA4 |
| C3orf14 | -0.60462 | 0.00534 | 0.03438 | CA4 |
| STAT5A | 0.58096 | 0.005339 | 0.03438 | CA4 |
| SMKR1 | 1.12168 | 0.005334 | 0.03438 | CA4 |
| CD72 | 0.896253 | 0.005353 | 0.034409 | CA4 |
| MED22 | 0.663262 | 0.005364 | 0.034436 | CA4 |
| FEZF2 | 0.766493 | 0.005377 | 0.034484 | CA4 |
| HTR1F | -1.13417 | 0.005428 | 0.034742 | CA4 |
| PIBF1 | -0.61961 | 0.005498 | 0.035122 | CA4 |
| DNAH8 | -1.1053 | 0.005503 | 0.03513 | CA4 |
| PANX2 | 0.633649 | 0.00553 | 0.035231 | CA4 |
| ALDH16A1 | 0.59275 | 0.005531 | 0.035231 | CA4 |

| | | | | |
|---|---|---|---|---|
| POLR2G | 0.610351 | 0.005542 | 0.035277 | CA4 |
| PTGER3 | -1.59151 | 0.00557 | 0.035376 | CA4 |
| PLCD3 | 0.627163 | 0.005571 | 0.035376 | CA4 |
| SLC9A3R2 | 0.615463 | 0.00566 | 0.035791 | CA4 |
| EFR3A | -0.60911 | 0.00566 | 0.035791 | CA4 |
| C1QTNF4 | 0.758674 | 0.005666 | 0.035791 | CA4 |
| FBLL1 | 0.714021 | 0.005669 | 0.035791 | CA4 |
| ZNF92 | -0.6048 | 0.005695 | 0.035908 | CA4 |
| SEPTIN7 | -0.6452 | 0.005704 | 0.035932 | CA4 |
| RPP25 | 0.598247 | 0.005705 | 0.035932 | CA4 |
| LHX2 | 0.584419 | 0.00571 | 0.035942 | CA4 |
| C16orf86 | 0.653374 | 0.005752 | 0.036101 | CA4 |
| SPHK1 | 0.769669 | 0.00575 | 0.036101 | CA4 |
| AHI1 | -0.58848 | 0.005764 | 0.036149 | CA4 |
| TAGLN2 | 0.757482 | 0.005782 | 0.036208 | CA4 |
| ARHGAP8 | 0.882802 | 0.005817 | 0.036379 | CA4 |
| PTPRQ | -0.95808 | 0.005826 | 0.036414 | CA4 |
| GRIN2D | 0.714773 | 0.005831 | 0.03643 | CA4 |
| H2BS1 | 1.515071 | 0.005836 | 0.036433 | CA4 |
| CCDC117 | -0.63778 | 0.005848 | 0.036452 | CA4 |
| SPOCK3 | -0.68646 | 0.005847 | 0.036452 | CA4 |
| PIK3R2 | 0.642393 | 0.005921 | 0.036814 | CA4 |
| TUBB2A | 0.669013 | 0.005922 | 0.036814 | CA4 |
| LSR | 0.94833 | 0.005932 | 0.036841 | CA4 |
| TNFAIP8L2 | 1.013397 | 0.005946 | 0.036851 | CA4 |
| EMILIN1 | 0.632229 | 0.005987 | 0.037033 | CA4 |
| B4GALT6 | -0.58281 | 0.006009 | 0.037126 | CA4 |
| GBP4 | 1.005853 | 0.00602 | 0.037178 | CA4 |
| PSTPIP1 | 0.594617 | 0.006025 | 0.037198 | CA4 |
| C1QB | 1.26252 | 0.006089 | 0.037537 | CA4 |
| EID2 | 0.637364 | 0.006089 | 0.037537 | CA4 |
| VAX2 | 0.842378 | 0.006179 | 0.037902 | CA4 |
| PTGDS | 0.590457 | 0.006199 | 0.037982 | CA4 |
| ATP2A3 | 0.828069 | 0.006236 | 0.038142 | CA4 |
| C4orf19 | 0.60216 | 0.006264 | 0.038238 | CA4 |
| REP15 | -1.00539 | 0.006279 | 0.038297 | CA4 |
| SPN | 0.973667 | 0.006281 | 0.038298 | CA4 |
| TNC | 0.698967 | 0.006297 | 0.038301 | CA4 |
| CLVS1 | -0.63054 | 0.006299 | 0.038301 | CA4 |
| LCAT | 0.625766 | 0.006293 | 0.038301 | CA4 |
| TP53I11 | 0.737305 | 0.006314 | 0.038311 | CA4 |
| FRRS1L | -0.65976 | 0.006338 | 0.038429 | CA4 |
| TGFB1I1 | 0.796478 | 0.006375 | 0.038621 | CA4 |
| OPALIN | -1.29778 | 0.006374 | 0.038621 | CA4 |

| | | | | |
|---|---|---|---|---|
| STX1A | 0.692002 | 0.006409 | 0.038768 | CA4 |
| RASD1 | 0.776635 | 0.006447 | 0.038899 | CA4 |
| AC098850.3 | -1.29106 | 0.006449 | 0.0389 | CA4 |
| H4C9 | 1.154444 | 0.006454 | 0.038911 | CA4 |
| PAQR4 | 0.590896 | 0.006485 | 0.039045 | CA4 |
| C8orf34 | -0.67275 | 0.006571 | 0.03945 | CA4 |
| HEXIM2 | 0.62222 | 0.006572 | 0.03945 | CA4 |
| BCAT1 | -0.62204 | 0.006583 | 0.039459 | CA4 |
| KRT222 | -1.04179 | 0.006602 | 0.039526 | CA4 |
| TCEA3 | 0.601985 | 0.00662 | 0.039563 | CA4 |
| LDHD | 0.659619 | 0.006687 | 0.039847 | CA4 |
| AC093525.1 | 1.331622 | 0.006715 | 0.039966 | CA4 |
| BCAN | 0.620916 | 0.006761 | 0.040168 | CA4 |
| LRRCC1 | -0.61889 | 0.006785 | 0.040264 | CA4 |
| STAC2 | 0.741223 | 0.006807 | 0.040384 | CA4 |
| SNCG | 0.773854 | 0.006822 | 0.040395 | CA4 |
| TNN | 1.680872 | 0.006869 | 0.040648 | CA4 |
| PBX4 | 0.716182 | 0.006885 | 0.04069 | CA4 |
| ZNF605 | -0.6522 | 0.006913 | 0.040744 | CA4 |
| BANF1 | 0.603795 | 0.006958 | 0.040953 | CA4 |
| SYDE2 | -0.62232 | 0.006981 | 0.041039 | CA4 |
| TEX9 | -0.78062 | 0.006984 | 0.041042 | CA4 |
| LBH | 0.915082 | 0.006995 | 0.041094 | CA4 |
| HSD17B8 | 0.691823 | 0.007023 | 0.041192 | CA4 |
| RFX2 | 0.602399 | 0.007043 | 0.041222 | CA4 |
| ALOX5AP | 1.321202 | 0.007077 | 0.041361 | CA4 |
| ZWILCH | -0.76316 | 0.007108 | 0.04147 | CA4 |
| RASGEF1C | 0.626784 | 0.00712 | 0.041495 | CA4 |
| EFCAB10 | -1.06105 | 0.007119 | 0.041495 | CA4 |
| ZNF691 | 0.660669 | 0.007132 | 0.041529 | CA4 |
| DHFR | -0.75946 | 0.007136 | 0.041529 | CA4 |
| GPX1 | 0.882745 | 0.007135 | 0.041529 | CA4 |
| H1-4 | 0.598184 | 0.007159 | 0.04162 | CA4 |
| MNS1 | -0.82868 | 0.007209 | 0.041835 | CA4 |
| SCX | 0.958393 | 0.007223 | 0.041902 | CA4 |
| RBM12 | -0.70089 | 0.007244 | 0.041974 | CA4 |
| CD47 | -0.62323 | 0.007249 | 0.041991 | CA4 |
| UBA52 | 0.588572 | 0.007264 | 0.042045 | CA4 |
| ADAD2 | 1.468097 | 0.007325 | 0.042312 | CA4 |
| GNG8 | 1.424868 | 0.007342 | 0.042394 | CA4 |
| CNTN3 | -0.67848 | 0.00735 | 0.042405 | CA4 |
| H2AC8 | 0.898244 | 0.007353 | 0.042405 | CA4 |
| LRRC37A | -0.83502 | 0.00737 | 0.042432 | CA4 |
| GDPD2 | 0.76152 | 0.00741 | 0.04262 | CA4 |

| | | | | |
|---|---|---|---|---|
| CBWD3 | -0.7571 | 0.007441 | 0.042754 | CA4 |
| OTOG | 1.055902 | 0.007488 | 0.0429 | CA4 |
| CLEC3B | 0.96269 | 0.007495 | 0.042927 | CA4 |
| RSC1A1 | -2.14092 | 0.007524 | 0.043018 | CA4 |
| LHPP | 0.607123 | 0.007616 | 0.043385 | CA4 |
| CLDN15 | 0.730946 | 0.007625 | 0.043427 | CA4 |
| ST18 | -0.71937 | 0.007638 | 0.043464 | CA4 |
| PCDH15 | -0.67413 | 0.007642 | 0.043464 | CA4 |
| C11orf91 | 1.381648 | 0.007643 | 0.043464 | CA4 |
| ZNF189 | -0.59681 | 0.007718 | 0.043826 | CA4 |
| ETAA1 | -0.59905 | 0.00772 | 0.043826 | CA4 |
| ZNF485 | -0.78029 | 0.007723 | 0.043826 | CA4 |
| GATA2 | 0.799157 | 0.007738 | 0.043884 | CA4 |
| ZNF100 | -0.63657 | 0.007766 | 0.044025 | CA4 |
| SERTAD1 | 0.853315 | 0.007785 | 0.044105 | CA4 |
| MFSD2A | 0.76284 | 0.0078 | 0.044157 | CA4 |
| CFAP43 | -0.77441 | 0.00783 | 0.044292 | CA4 |
| CTNNA3 | -1.09676 | 0.007848 | 0.04434 | CA4 |
| ARMCX5 | -0.75116 | 0.007857 | 0.044372 | CA4 |
| DYNLT3 | -0.59014 | 0.00787 | 0.044401 | CA4 |
| PEG10 | 0.744522 | 0.0079 | 0.044505 | CA4 |
| PIR | 0.653864 | 0.007911 | 0.044519 | CA4 |
| COL6A2 | 0.938141 | 0.007912 | 0.044519 | CA4 |
| ZNF660 | -0.7487 | 0.007913 | 0.044519 | CA4 |
| SYNGR2 | 0.628456 | 0.007938 | 0.044628 | CA4 |
| SDSL | 0.86595 | 0.00795 | 0.044646 | CA4 |
| FOLR2 | 1.114874 | 0.007952 | 0.044647 | CA4 |
| ZNF836 | -0.71021 | 0.007989 | 0.044822 | CA4 |
| SLITRK4 | -0.73264 | 0.007998 | 0.044853 | CA4 |
| FAM181A | 0.68722 | 0.008033 | 0.045023 | CA4 |
| PABPC5 | -0.71214 | 0.008057 | 0.045125 | CA4 |
| ZNF404 | -0.61743 | 0.008106 | 0.045306 | CA4 |
| ZFP36L1 | 0.586697 | 0.008108 | 0.045306 | CA4 |
| PROB1 | 0.686119 | 0.008121 | 0.045341 | CA4 |
| CAPS | 0.886708 | 0.008152 | 0.045499 | CA4 |
| SAMD5 | -0.76981 | 0.008161 | 0.045518 | CA4 |
| STX11 | 1.1559 | 0.008164 | 0.045521 | CA4 |
| CLVS2 | -0.63739 | 0.008181 | 0.045596 | CA4 |
| NCF2 | 1.00508 | 0.008253 | 0.045889 | CA4 |
| AC120114.4 | 1.432827 | 0.008274 | 0.045961 | CA4 |
| TMEM204 | 0.696483 | 0.008281 | 0.045981 | CA4 |
| ALDH3B1 | 0.884363 | 0.008287 | 0.045994 | CA4 |
| AMDHD2 | 0.627787 | 0.008292 | 0.045994 | CA4 |
| MOBP | -1.08244 | 0.00829 | 0.045994 | CA4 |

| | | | | |
|---|---|---|---|---|
| SLC44A5 | -0.5992 | 0.008309 | 0.046073 | CA4 |
| F10 | 1.649641 | 0.008319 | 0.046115 | CA4 |
| TNFRSF13C | 0.843872 | 0.008339 | 0.046207 | CA4 |
| CCDC88B | 0.61191 | 0.008345 | 0.046228 | CA4 |
| TUBE1 | -0.59093 | 0.008377 | 0.046341 | CA4 |
| FZD8 | 0.710534 | 0.008441 | 0.046615 | CA4 |
| SLC25A22 | 0.664456 | 0.008441 | 0.046615 | CA4 |
| SIX5 | 0.710653 | 0.008445 | 0.046621 | CA4 |
| AC026464.3 | -1.93251 | 0.008507 | 0.046871 | CA4 |
| KIF11 | -1.02704 | 0.008562 | 0.047117 | CA4 |
| GNG11 | 0.753815 | 0.008579 | 0.047171 | CA4 |
| FSCN2 | 1.022975 | 0.008668 | 0.047574 | CA4 |
| TDRD6 | -0.59778 | 0.008677 | 0.047595 | CA4 |
| MCM5 | 0.584161 | 0.008731 | 0.047829 | CA4 |
| GSN | 0.614513 | 0.008809 | 0.048124 | CA4 |
| NNAT | 0.755572 | 0.008855 | 0.048314 | CA4 |
| TMEM198 | 0.65667 | 0.00895 | 0.048761 | CA4 |
| ACAP1 | 0.871421 | 0.008957 | 0.048772 | CA4 |
| SGCA | 1.848602 | 0.008962 | 0.048772 | CA4 |
| ICAM1 | 0.995768 | 0.009037 | 0.049056 | CA4 |
| C5orf49 | 0.703831 | 0.009067 | 0.049168 | CA4 |
| FOXL2 | 1.202062 | 0.00911 | 0.049354 | CA4 |
| TNFSF4 | -0.63317 | 0.009147 | 0.049501 | CA4 |
| SPRED3 | 0.672366 | 0.009182 | 0.049628 | CA4 |
| CARTPT | 1.373157 | 0.009189 | 0.049645 | CA4 |
| GLRA3 | -0.88117 | 0.009217 | 0.049766 | CA4 |
| LACC1 | -0.92303 | 0.009265 | 0.049921 | CA4 |
| EPHX3 | 2.752902 | 7.17E-05 | 0.002082 | CA4 |
| PADI4 | 2.346112 | 0.003544 | 0.026093 | CA4 |
| PROK2 | 2.565942 | 0.001924 | 0.01767 | CA4 |
| FAM47C | -1.85732 | 0.00526 | 0.034064 | CA4 |
| UBD | 4.052033 | 0.000203 | 0.004104 | CA4 |
| CD8B2 | 2.277851 | 0.007235 | 0.041952 | CA4 |
| PPIAL4F | -1.65521 | 0.008156 | 0.045506 | CA4 |
| | | | | |
| PNPLA2 | 1.267583 | 4.94E-11 | 8.07E-07 | EC |
| CHPF | 1.315567 | 6.42E-10 | 5.25E-06 | EC |
| RPP21 | 1.023741 | 1.70E-09 | 9.27E-06 | EC |
| POLR2L | 1.212422 | 4.26E-09 | 1.74E-05 | EC |
| RPL27 | 1.393329 | 1.14E-08 | 3.72E-05 | EC |
| ASTN2 | 0.673812 | 1.44E-08 | 3.92E-05 | EC |
| EDF1 | 1.111977 | 2.12E-08 | 4.94E-05 | EC |
| SCAMP4 | 0.807675 | 2.58E-08 | 5.27E-05 | EC |
| PMS1 | -0.84849 | 3.18E-08 | 5.34E-05 | EC |

| | | | | |
|---|---|---|---|---|
| KIAA2013 | 0.821862 | 3.86E-08 | 5.34E-05 | EC |
| SMARCAD1 | -0.63315 | 3.92E-08 | 5.34E-05 | EC |
| SP2 | 0.782656 | 3.41E-08 | 5.34E-05 | EC |
| TMSB10 | 1.364676 | 4.99E-08 | 5.82E-05 | EC |
| WDR83OS | 0.950896 | 4.88E-08 | 5.82E-05 | EC |
| TESK1 | 0.706914 | 6.80E-08 | 6.30E-05 | EC |
| PUS7L | -0.88187 | 6.46E-08 | 6.30E-05 | EC |
| DUSP23 | 1.424016 | 6.90E-08 | 6.30E-05 | EC |
| VPS50 | -0.70286 | 7.55E-08 | 6.40E-05 | EC |
| LONP1 | 0.72484 | 7.83E-08 | 6.40E-05 | EC |
| ZNF574 | 0.925874 | 1.04E-07 | 8.13E-05 | EC |
| CSTB | 0.830206 | 1.36E-07 | 9.67E-05 | EC |
| MEX3D | 1.010521 | 1.34E-07 | 9.67E-05 | EC |
| EIF6 | 0.79039 | 1.42E-07 | 9.67E-05 | EC |
| REP15 | -1.33619 | 1.61E-07 | 9.94E-05 | EC |
| ZNF579 | 0.882544 | 1.64E-07 | 9.94E-05 | EC |
| COX8A | 1.406855 | 1.80E-07 | 0.000105 | EC |
| ADRM1 | 0.718904 | 1.97E-07 | 0.000105 | EC |
| CRB2 | 1.246638 | 2.01E-07 | 0.000105 | EC |
| CRTC2 | 0.715636 | 1.94E-07 | 0.000105 | EC |
| SCAND1 | 1.187551 | 2.06E-07 | 0.000105 | EC |
| SF3B5 | 0.981456 | 2.29E-07 | 0.000113 | EC |
| KCNH2 | 0.851254 | 2.99E-07 | 0.000116 | EC |
| XAB2 | 0.731112 | 2.87E-07 | 0.000116 | EC |
| C19orf53 | 1.161725 | 2.95E-07 | 0.000116 | EC |
| ILVBL | 0.732436 | 3.15E-07 | 0.000116 | EC |
| MRPS2 | 1.222898 | 2.67E-07 | 0.000116 | EC |
| MEA1 | 0.693093 | 3.34E-07 | 0.000116 | EC |
| ROMO1 | 1.34208 | 3.20E-07 | 0.000116 | EC |
| ARMC5 | 0.884584 | 3.19E-07 | 0.000116 | EC |
| NOSIP | 0.763525 | 3.30E-07 | 0.000116 | EC |
| RRAD | 2.661489 | 2.42E-07 | 0.000116 | EC |
| GRINA | 0.880541 | 2.67E-07 | 0.000116 | EC |
| POLR2A | 0.663081 | 2.99E-07 | 0.000116 | EC |
| NAA38 | 1.128898 | 2.54E-07 | 0.000116 | EC |
| EFCAB7 | -0.78599 | 2.75E-07 | 0.000116 | EC |
| RPUSD1 | 0.856373 | 3.89E-07 | 0.000119 | EC |
| ERO1B | -0.66875 | 3.97E-07 | 0.000119 | EC |
| CLPTM1 | 0.73872 | 3.85E-07 | 0.000119 | EC |
| MAGOHB | -0.76131 | 3.99E-07 | 0.000119 | EC |
| ZNF142 | 0.586838 | 3.83E-07 | 0.000119 | EC |
| DYNLRB1 | 1.102649 | 3.87E-07 | 0.000119 | EC |
| LRFN3 | 0.78328 | 3.50E-07 | 0.000119 | EC |
| AK9 | -0.71671 | 3.77E-07 | 0.000119 | EC |

| | | | | |
|---|---|---|---|---|
| PTPRF | 0.594014 | 4.15E-07 | 0.000119 | EC |
| RAB11B | 0.731226 | 4.15E-07 | 0.000119 | EC |
| MT2A | 1.339531 | 4.30E-07 | 0.00012 | EC |
| INAFM1 | 0.812215 | 4.33E-07 | 0.00012 | EC |
| ZFPL1 | 0.650106 | 4.62E-07 | 0.000126 | EC |
| BCL9L | 0.82391 | 4.83E-07 | 0.000129 | EC |
| ABHD12 | 0.699245 | 5.04E-07 | 0.000131 | EC |
| PRDX5 | 1.040238 | 5.10E-07 | 0.000131 | EC |
| SF3B4 | 0.742172 | 5.13E-07 | 0.000131 | EC |
| HSPB6 | 1.373063 | 5.30E-07 | 0.000133 | EC |
| BAHD1 | 0.700709 | 5.39E-07 | 0.000134 | EC |
| CACTIN | 0.633617 | 5.66E-07 | 0.000136 | EC |
| MED16 | 0.828039 | 5.81E-07 | 0.000138 | EC |
| ANTKMT | 1.166143 | 6.68E-07 | 0.000154 | EC |
| PSMB10 | 0.989682 | 6.65E-07 | 0.000154 | EC |
| TRIP11 | -0.81255 | 7.40E-07 | 0.000163 | EC |
| ATG4D | 0.58507 | 7.73E-07 | 0.000168 | EC |
| NDUFA11 | 0.865492 | 7.81E-07 | 0.000168 | EC |
| TECR | 0.84185 | 8.07E-07 | 0.000171 | EC |
| EFNB1 | 0.955377 | 8.50E-07 | 0.000174 | EC |
| GUCY1A2 | -0.84651 | 8.42E-07 | 0.000174 | EC |
| NOP10 | 1.326681 | 8.41E-07 | 0.000174 | EC |
| SHARPIN | 0.737682 | 9.30E-07 | 0.000185 | EC |
| DNLZ | 0.946661 | 9.29E-07 | 0.000185 | EC |
| VASN | 1.349425 | 9.46E-07 | 0.000186 | EC |
| ARHGDIA | 0.643852 | 9.71E-07 | 0.000189 | EC |
| EXOSC4 | 0.706613 | 1.03E-06 | 0.000198 | EC |
| ATN1 | 0.645563 | 1.04E-06 | 0.000198 | EC |
| ODF2L | -0.96775 | 1.06E-06 | 0.000199 | EC |
| SCNM1 | 0.670371 | 1.09E-06 | 0.000201 | EC |
| AC106886.6 | 0.947701 | 1.09E-06 | 0.000201 | EC |
| SLC39A3 | 0.683813 | 1.11E-06 | 0.000201 | EC |
| DPM2 | 0.686308 | 1.15E-06 | 0.000204 | EC |
| VPS51 | 0.776599 | 1.14E-06 | 0.000204 | EC |
| U2AF2 | 0.749377 | 1.21E-06 | 0.000208 | EC |
| PFDN2 | 1.058765 | 1.20E-06 | 0.000208 | EC |
| ARHGEF10L | 0.610562 | 1.29E-06 | 0.000212 | EC |
| RBM41 | -0.84583 | 1.26E-06 | 0.000212 | EC |
| PUF60 | 0.666895 | 1.29E-06 | 0.000212 | EC |
| UBL5 | 1.034742 | 1.30E-06 | 0.000212 | EC |
| RPS11 | 0.977393 | 1.33E-06 | 0.000215 | EC |
| CIAO2B | 1.083959 | 1.37E-06 | 0.000218 | EC |
| PLD5 | -0.6487 | 1.37E-06 | 0.000218 | EC |
| RABAC1 | 1.0653 | 1.41E-06 | 0.00022 | EC |

| | | | | |
|---|---|---|---|---|
| ERF | 0.791926 | 1.40E-06 | 0.00022 | EC |
| ARAF | 0.676769 | 1.45E-06 | 0.000223 | EC |
| ELOF1 | 0.928994 | 1.48E-06 | 0.000223 | EC |
| TMEM232 | -0.66825 | 1.48E-06 | 0.000223 | EC |
| ZNF777 | 0.692041 | 1.49E-06 | 0.000223 | EC |
| H4C11 | 1.600381 | 1.51E-06 | 0.000224 | EC |
| ZNF846 | -0.69287 | 1.52E-06 | 0.000224 | EC |
| FLYWCH1 | 0.662085 | 1.68E-06 | 0.000231 | EC |
| GSTP1 | 0.906905 | 1.68E-06 | 0.000231 | EC |
| POLR2I | 0.918161 | 1.59E-06 | 0.000231 | EC |
| PPDPF | 1.138134 | 1.67E-06 | 0.000231 | EC |
| RBM42 | 0.76878 | 1.60E-06 | 0.000231 | EC |
| SHISA5 | 0.652974 | 1.67E-06 | 0.000231 | EC |
| LSM10 | 0.699244 | 1.68E-06 | 0.000231 | EC |
| RNF208 | 0.934642 | 1.66E-06 | 0.000231 | EC |
| SNRPC | 0.771968 | 1.77E-06 | 0.000241 | EC |
| MIF | 0.975736 | 1.79E-06 | 0.000242 | EC |
| RASA2 | -0.68912 | 1.97E-06 | 0.000262 | EC |
| AP2A1 | 0.592587 | 1.99E-06 | 0.000262 | EC |
| ARL2 | 0.778799 | 2.06E-06 | 0.00027 | EC |
| PRIMPOL | -0.70438 | 2.09E-06 | 0.000271 | EC |
| SLC10A3 | 0.887343 | 2.13E-06 | 0.000274 | EC |
| TCF4 | -0.62139 | 2.16E-06 | 0.000276 | EC |
| MZT2B | 0.936386 | 2.23E-06 | 0.000283 | EC |
| CEP97 | -0.72538 | 2.30E-06 | 0.000289 | EC |
| TMEM115 | 0.640597 | 2.32E-06 | 0.00029 | EC |
| DUSP15 | 0.99143 | 2.36E-06 | 0.000292 | EC |
| MZT2A | 0.88902 | 2.46E-06 | 0.000298 | EC |
| ZNHIT2 | 0.927441 | 2.45E-06 | 0.000298 | EC |
| NBN | -0.61833 | 2.57E-06 | 0.000298 | EC |
| OSGIN1 | 1.328906 | 2.58E-06 | 0.000298 | EC |
| UBE2L6 | 0.794111 | 2.52E-06 | 0.000298 | EC |
| SYVN1 | 0.612564 | 2.61E-06 | 0.000298 | EC |
| CCDC186 | -0.838 | 2.59E-06 | 0.000298 | EC |
| SAMD5 | -1.07035 | 2.61E-06 | 0.000298 | EC |
| AAMP | 0.610473 | 2.69E-06 | 0.000299 | EC |
| FASTKD1 | -0.69757 | 2.68E-06 | 0.000299 | EC |
| VKORC1 | 0.753627 | 2.65E-06 | 0.000299 | EC |
| RAB4B-EGLN2 | 1.181835 | 2.69E-06 | 0.000299 | EC |
| PKN2 | -0.63956 | 2.78E-06 | 0.0003 | EC |
| GNPTG | 0.685747 | 2.76E-06 | 0.0003 | EC |
| C12orf57 | 1.025777 | 2.79E-06 | 0.0003 | EC |
| NDUFS7 | 0.762609 | 2.74E-06 | 0.0003 | EC |
| ZYX | 0.713275 | 2.77E-06 | 0.0003 | EC |

| | | | | |
|---|---|---|---|---|
| TMEM151A | 0.926701 | 2.84E-06 | 0.000303 | EC |
| PDCD2L | 0.870291 | 2.89E-06 | 0.000304 | EC |
| IRF2BP1 | 0.682329 | 2.90E-06 | 0.000304 | EC |
| IRS2 | 0.622243 | 2.88E-06 | 0.000304 | EC |
| CCDC124 | 0.673365 | 2.95E-06 | 0.000307 | EC |
| SLC22A17 | 0.966091 | 2.97E-06 | 0.000308 | EC |
| ERCC6L2 | -0.61442 | 3.00E-06 | 0.000309 | EC |
| SQSTM1 | 0.685757 | 3.10E-06 | 0.000313 | EC |
| ZNF408 | 0.658328 | 3.14E-06 | 0.000313 | EC |
| CTSD | 1.101588 | 3.17E-06 | 0.000314 | EC |
| LRP1 | 0.789332 | 3.19E-06 | 0.000314 | EC |
| LAMP1 | 0.648754 | 3.22E-06 | 0.000315 | EC |
| TLE5 | 0.64298 | 3.54E-06 | 0.000338 | EC |
| FBXL15 | 0.722089 | 3.56E-06 | 0.000338 | EC |
| SOS1 | -0.6306 | 3.57E-06 | 0.000338 | EC |
| NBEAL1 | -0.67385 | 3.51E-06 | 0.000338 | EC |
| NDUFB9 | 0.756849 | 3.56E-06 | 0.000338 | EC |
| IFI27L2 | 0.925844 | 3.67E-06 | 0.000341 | EC |
| PLEC | 0.838229 | 3.67E-06 | 0.000341 | EC |
| CEP162 | -0.85815 | 3.83E-06 | 0.000353 | EC |
| AHDC1 | 0.636584 | 3.87E-06 | 0.000355 | EC |
| AP1B1 | 0.603157 | 3.95E-06 | 0.000356 | EC |
| LDB1 | 0.765098 | 3.96E-06 | 0.000356 | EC |
| HAUS3 | -0.80007 | 3.92E-06 | 0.000356 | EC |
| VPS13C | -0.60201 | 4.07E-06 | 0.000364 | EC |
| PRKCSH | 0.610167 | 4.14E-06 | 0.000367 | EC |
| ZNF784 | 0.859734 | 4.27E-06 | 0.000375 | EC |
| PSMA7 | 0.754565 | 4.31E-06 | 0.000377 | EC |
| TSEN34 | 0.663999 | 4.40E-06 | 0.000382 | EC |
| CCDC82 | -0.87622 | 4.45E-06 | 0.000385 | EC |
| PLD3 | 0.778041 | 4.55E-06 | 0.000391 | EC |
| C6orf226 | 1.306181 | 4.56E-06 | 0.000391 | EC |
| RAMP1 | 1.218533 | 4.75E-06 | 0.000402 | EC |
| CHMP6 | 0.666237 | 4.96E-06 | 0.000415 | EC |
| ATRX | -0.65258 | 5.00E-06 | 0.000416 | EC |
| SOD3 | 1.469698 | 5.02E-06 | 0.000416 | EC |
| ATP5MC2 | 0.643844 | 5.09E-06 | 0.00042 | EC |
| NDUFS6 | 0.7639 | 5.17E-06 | 0.000423 | EC |
| NFKBIL1 | 0.626784 | 5.16E-06 | 0.000423 | EC |
| PRH1 | -0.75473 | 5.42E-06 | 0.000438 | EC |
| LDOC1 | 0.945261 | 5.54E-06 | 0.000443 | EC |
| DDX54 | 0.59348 | 5.61E-06 | 0.000446 | EC |
| TRAPPC5 | 1.485924 | 5.62E-06 | 0.000446 | EC |
| EIF3G | 0.592398 | 5.66E-06 | 0.000447 | EC |

| | | | | |
|---|---|---|---|---|
| MCOLN3 | -2.61467 | 5.84E-06 | 0.00045 | EC |
| BICRA | 0.755996 | 5.75E-06 | 0.00045 | EC |
| PLPPR2 | 0.770994 | 5.85E-06 | 0.00045 | EC |
| FBXW5 | 0.709933 | 5.74E-06 | 0.00045 | EC |
| CFL1 | 0.830914 | 5.91E-06 | 0.00045 | EC |
| BAX | 0.770431 | 6.06E-06 | 0.000454 | EC |
| TOMM7 | 0.896631 | 6.18E-06 | 0.000461 | EC |
| SENP7 | -0.63607 | 6.21E-06 | 0.000461 | EC |
| AKIRIN2 | 0.657421 | 6.25E-06 | 0.000462 | EC |
| FURIN | 0.612719 | 6.30E-06 | 0.000464 | EC |
| CEP44 | -0.66073 | 6.52E-06 | 0.000477 | EC |
| GPX4 | 0.598233 | 6.61E-06 | 0.000482 | EC |
| INTS5 | 0.801881 | 6.64E-06 | 0.000482 | EC |
| SLC45A1 | 0.615778 | 6.78E-06 | 0.00049 | EC |
| ZFP14 | -0.63392 | 6.97E-06 | 0.000499 | EC |
| MRPL24 | 0.841895 | 7.02E-06 | 0.000499 | EC |
| THAP4 | 0.680977 | 6.98E-06 | 0.000499 | EC |
| RFX1 | 0.724359 | 7.11E-06 | 0.000499 | EC |
| PNPT1 | -0.58813 | 7.06E-06 | 0.000499 | EC |
| NLRX1 | 0.722182 | 7.15E-06 | 0.000499 | EC |
| GK5 | -0.68676 | 7.27E-06 | 0.000506 | EC |
| RNF187 | 0.72952 | 7.36E-06 | 0.00051 | EC |
| PAK4 | 0.699854 | 7.47E-06 | 0.000512 | EC |
| ZUP1 | -0.73131 | 7.52E-06 | 0.000512 | EC |
| GATD3A | 0.613939 | 7.52E-06 | 0.000512 | EC |
| ZNF781 | -0.93873 | 7.49E-06 | 0.000512 | EC |
| H2AX | 0.764986 | 7.63E-06 | 0.000513 | EC |
| TIMP1 | 1.551228 | 7.68E-06 | 0.000514 | EC |
| TBCB | 0.727789 | 7.77E-06 | 0.000516 | EC |
| G6PD | 0.731212 | 7.74E-06 | 0.000516 | EC |
| PCSK1N | 1.349672 | 8.12E-06 | 0.000529 | EC |
| PEPD | 0.866672 | 8.08E-06 | 0.000529 | EC |
| ATP6V1F | 0.785459 | 8.12E-06 | 0.000529 | EC |
| FAM174C | 0.837573 | 8.03E-06 | 0.000529 | EC |
| BLVRB | 0.886126 | 8.19E-06 | 0.00053 | EC |
| GRB14 | -1.47435 | 8.27E-06 | 0.000532 | EC |
| ZFPM1 | 0.932466 | 8.32E-06 | 0.000533 | EC |
| PEX14 | 0.618657 | 8.46E-06 | 0.00054 | EC |
| YJU2 | 0.692771 | 8.72E-06 | 0.00055 | EC |
| UBXN11 | 0.595433 | 8.71E-06 | 0.00055 | EC |
| DPY19L2 | -0.82375 | 8.86E-06 | 0.000557 | EC |
| NDUFB2 | 0.740623 | 9.25E-06 | 0.000562 | EC |
| RGS17 | -1.03794 | 9.12E-06 | 0.000562 | EC |
| CDADC1 | -0.60385 | 9.03E-06 | 0.000562 | EC |

| | | | | |
|---|---|---|---|---|
| SF3A2 | 0.729251 | 9.15E-06 | 0.000562 | EC |
| CLPP | 0.886674 | 9.22E-06 | 0.000562 | EC |
| PLAAT4 | 1.032148 | 9.11E-06 | 0.000562 | EC |
| DAGLA | 0.722227 | 9.31E-06 | 0.000562 | EC |
| PEF1 | 0.658505 | 9.31E-06 | 0.000562 | EC |
| LGI3 | 0.920338 | 9.24E-06 | 0.000562 | EC |
| ZNF566 | -0.78852 | 9.33E-06 | 0.000562 | EC |
| FEZF2 | 1.088874 | 9.37E-06 | 0.000563 | EC |
| TBC1D25 | 0.74096 | 9.69E-06 | 0.000576 | EC |
| AKAP7 | -0.72678 | 9.66E-06 | 0.000576 | EC |
| COX6B1 | 1.074555 | 9.65E-06 | 0.000576 | EC |
| TICAM1 | 0.670941 | 9.88E-06 | 0.000585 | EC |
| CRIP2 | 0.602082 | 9.99E-06 | 0.00059 | EC |
| ZC3H3 | 0.662239 | 1.01E-05 | 0.000591 | EC |
| SLC2A8 | 0.584499 | 1.01E-05 | 0.000591 | EC |
| EPN1 | 0.654828 | 1.04E-05 | 0.000595 | EC |
| RFXANK | 0.663196 | 1.04E-05 | 0.000595 | EC |
| ABCC3 | 2.122587 | 1.03E-05 | 0.000595 | EC |
| MRPS26 | 0.628784 | 1.04E-05 | 0.000595 | EC |
| ATR | -0.62612 | 1.04E-05 | 0.000595 | EC |
| ZNF624 | -0.87373 | 1.04E-05 | 0.000595 | EC |
| H2AC19 | 1.111719 | 1.03E-05 | 0.000595 | EC |
| PTPRU | 0.695561 | 1.06E-05 | 0.000598 | EC |
| C4orf48 | 1.544118 | 1.06E-05 | 0.000599 | EC |
| BLOC1S1 | 0.96469 | 1.09E-05 | 0.000612 | EC |
| SAP130 | 0.711031 | 1.12E-05 | 0.000624 | EC |
| IFT81 | -0.65724 | 1.15E-05 | 0.000635 | EC |
| EXOC6 | -0.64352 | 1.16E-05 | 0.000635 | EC |
| SH3BGRL3 | 0.622259 | 1.15E-05 | 0.000635 | EC |
| CCDC74B | 0.640626 | 1.15E-05 | 0.000635 | EC |
| LTBP3 | 0.659872 | 1.15E-05 | 0.000635 | EC |
| PLEKHH3 | 0.619381 | 1.17E-05 | 0.000637 | EC |
| SNX13 | -0.67772 | 1.17E-05 | 0.000637 | EC |
| PUS10 | -0.72207 | 1.17E-05 | 0.000637 | EC |
| UQCRQ | 0.727772 | 1.22E-05 | 0.000654 | EC |
| GAMT | 0.82891 | 1.23E-05 | 0.000659 | EC |
| FBXW9 | 0.93985 | 1.24E-05 | 0.00066 | EC |
| ADPRS | 0.726394 | 1.27E-05 | 0.000673 | EC |
| ATP2A3 | 1.100659 | 1.28E-05 | 0.000676 | EC |
| BCLAF3 | -0.69198 | 1.29E-05 | 0.000678 | EC |
| C1orf35 | 0.80285 | 1.31E-05 | 0.000682 | EC |
| CCDC138 | -0.61724 | 1.32E-05 | 0.000683 | EC |
| UBA6 | -0.65303 | 1.34E-05 | 0.000685 | EC |
| POLRMT | 0.664109 | 1.33E-05 | 0.000685 | EC |

| | | | | |
|---|---|---|---|---|
| ALYREF | 0.817528 | 1.34E-05 | 0.000685 | EC |
| H4C12 | 1.435086 | 1.33E-05 | 0.000685 | EC |
| AC007325.2 | 1.22056 | 1.35E-05 | 0.000686 | EC |
| ZNF626 | -0.61699 | 1.41E-05 | 0.000712 | EC |
| RFX2 | 0.78604 | 1.45E-05 | 0.000725 | EC |
| PFKL | 0.607345 | 1.45E-05 | 0.000725 | EC |
| C11orf68 | 0.666111 | 1.46E-05 | 0.00073 | EC |
| ZGRF1 | -0.89476 | 1.47E-05 | 0.000731 | EC |
| CCDC148 | -0.66448 | 1.48E-05 | 0.000734 | EC |
| CASP8AP2 | -0.81299 | 1.50E-05 | 0.00074 | EC |
| TUBG1 | 0.727141 | 1.50E-05 | 0.00074 | EC |
| SERPING1 | 0.921196 | 1.52E-05 | 0.000744 | EC |
| IDH3G | 0.760389 | 1.53E-05 | 0.000745 | EC |
| POMGNT2 | 0.672897 | 1.53E-05 | 0.000745 | EC |
| OCIAD1 | -0.65124 | 1.54E-05 | 0.000745 | EC |
| LCORL | -0.6846 | 1.56E-05 | 0.000751 | EC |
| PCDH1 | 0.618448 | 1.58E-05 | 0.000757 | EC |
| ZNF680 | -0.66562 | 1.58E-05 | 0.000757 | EC |
| LGALS1 | 0.947207 | 1.59E-05 | 0.000758 | EC |
| DISP3 | 1.26664 | 1.61E-05 | 0.000767 | EC |
| BCAR1 | 0.661276 | 1.62E-05 | 0.000768 | EC |
| H4C2 | 1.54513 | 1.63E-05 | 0.000768 | EC |
| GCFC2 | -0.6514 | 1.64E-05 | 0.00077 | EC |
| AC024592.3 | 1.138092 | 1.64E-05 | 0.00077 | EC |
| TM9SF4 | 0.594121 | 1.68E-05 | 0.000778 | EC |
| SLC27A1 | 0.658813 | 1.68E-05 | 0.000778 | EC |
| RMDN2 | -0.69524 | 1.69E-05 | 0.000781 | EC |
| MEPCE | 0.621558 | 1.70E-05 | 0.000781 | EC |
| NXF2B | -3.11767 | 1.70E-05 | 0.000781 | EC |
| TATDN1 | -0.7549 | 1.71E-05 | 0.000781 | EC |
| PER2 | 0.765318 | 1.73E-05 | 0.000789 | EC |
| U2SURP | -0.59358 | 1.74E-05 | 0.000789 | EC |
| LRWD1 | 0.682014 | 1.75E-05 | 0.000791 | EC |
| SMC6 | -0.62385 | 1.75E-05 | 0.000791 | EC |
| ANAPC4 | -0.62849 | 1.80E-05 | 0.000809 | EC |
| TIMM13 | 0.657804 | 1.81E-05 | 0.000811 | EC |
| NUDC | 0.636192 | 1.88E-05 | 0.000838 | EC |
| PTPN4 | -0.70099 | 1.90E-05 | 0.000839 | EC |
| FOXP4 | 0.592175 | 1.90E-05 | 0.000839 | EC |
| NTSR2 | 1.172471 | 1.90E-05 | 0.000839 | EC |
| SCAMP3 | 0.690207 | 1.94E-05 | 0.000854 | EC |
| EMP3 | 1.156482 | 1.96E-05 | 0.000858 | EC |
| RAD18 | -0.61307 | 2.01E-05 | 0.000874 | EC |
| MPHOSPH9 | -0.67967 | 2.03E-05 | 0.00088 | EC |

| | | | | |
|---|---|---|---|---|
| SCN9A | -0.89199 | 2.10E-05 | 0.000906 | EC |
| IFT80 | -0.78156 | 2.15E-05 | 0.000914 | EC |
| UGGT2 | -0.68974 | 2.15E-05 | 0.000914 | EC |
| CAPS | 1.191753 | 2.15E-05 | 0.000914 | EC |
| NDUFA3 | 0.823673 | 2.15E-05 | 0.000914 | EC |
| RHOG | 0.993865 | 2.14E-05 | 0.000914 | EC |
| TAS2R14 | -1.17003 | 2.15E-05 | 0.000914 | EC |
| METRN | 1.110839 | 2.23E-05 | 0.000938 | EC |
| RAVER1 | 0.644579 | 2.22E-05 | 0.000938 | EC |
| CPTP | 0.654505 | 2.22E-05 | 0.000938 | EC |
| FAM207A | 0.717809 | 2.26E-05 | 0.000946 | EC |
| ZBTB8OS | -0.67163 | 2.27E-05 | 0.000948 | EC |
| DOCK11 | -0.62211 | 2.28E-05 | 0.000949 | EC |
| AMIGO3 | 2.281847 | 2.28E-05 | 0.000949 | EC |
| ZNF865 | 0.742086 | 2.33E-05 | 0.000962 | EC |
| MAF1 | 0.689741 | 2.34E-05 | 0.000965 | EC |
| POLI | -0.67098 | 2.37E-05 | 0.000972 | EC |
| TBC1D31 | -0.64882 | 2.41E-05 | 0.000982 | EC |
| FAM204A | -0.58784 | 2.40E-05 | 0.000982 | EC |
| DRD4 | 1.377043 | 2.45E-05 | 0.000993 | EC |
| NUDCD1 | -0.70474 | 2.45E-05 | 0.000993 | EC |
| PLXNB2 | 0.656995 | 2.48E-05 | 0.001003 | EC |
| PROSER3 | 0.594742 | 2.50E-05 | 0.001006 | EC |
| ECE1 | 0.729205 | 2.54E-05 | 0.001011 | EC |
| POSTN | -1.91199 | 2.52E-05 | 0.001011 | EC |
| LGI4 | 0.776818 | 2.54E-05 | 0.001011 | EC |
| CLN3 | 0.607507 | 2.53E-05 | 0.001011 | EC |
| ZNF282 | 0.694401 | 2.56E-05 | 0.001015 | EC |
| TMEM205 | 0.743632 | 2.59E-05 | 0.001024 | EC |
| LIN28B | -1.30313 | 2.64E-05 | 0.001039 | EC |
| PMM1 | 0.608746 | 2.67E-05 | 0.00104 | EC |
| ETFB | 0.659157 | 2.66E-05 | 0.00104 | EC |
| AVPI1 | 0.764706 | 2.66E-05 | 0.00104 | EC |
| SAC3D1 | 0.942496 | 2.67E-05 | 0.00104 | EC |
| MADCAM1 | 1.081442 | 2.69E-05 | 0.001047 | EC |
| ARID4A | -0.6394 | 2.71E-05 | 0.00105 | EC |
| MMP16 | -0.89064 | 2.78E-05 | 0.001064 | EC |
| SPATA2L | 0.783346 | 2.78E-05 | 0.001064 | EC |
| CCDC66 | -0.63719 | 2.83E-05 | 0.001075 | EC |
| DNALI1 | 0.731615 | 2.84E-05 | 0.001077 | EC |
| IPO13 | 0.587223 | 2.89E-05 | 0.001093 | EC |
| PIGT | 0.745127 | 2.90E-05 | 0.001093 | EC |
| F8A3 | 0.771348 | 2.95E-05 | 0.001109 | EC |
| PPP4C | 0.582706 | 2.99E-05 | 0.00112 | EC |

| | | | | |
|---|---|---|---|---|
| ZNF431 | -0.64473 | 2.99E-05 | 0.00112 | EC |
| FBXL14 | 0.673267 | 3.05E-05 | 0.001138 | EC |
| ISLR | 1.077004 | 3.07E-05 | 0.001144 | EC |
| SART1 | 0.63178 | 3.13E-05 | 0.001162 | EC |
| NOTCH1 | 0.959262 | 3.14E-05 | 0.001162 | EC |
| GDPD5 | 0.64705 | 3.19E-05 | 0.001177 | EC |
| ZNF681 | -0.77334 | 3.20E-05 | 0.001177 | EC |
| NFIC | 0.603008 | 3.30E-05 | 0.001207 | EC |
| SIGMAR1 | 0.598014 | 3.29E-05 | 0.001207 | EC |
| CPLANE1 | -0.68651 | 3.30E-05 | 0.001207 | EC |
| ACTN4 | 0.615204 | 3.36E-05 | 0.001219 | EC |
| JMJD8 | 0.584782 | 3.35E-05 | 0.001219 | EC |
| MDM4 | -0.75244 | 3.36E-05 | 0.001219 | EC |
| SENP6 | -0.61707 | 3.42E-05 | 0.001238 | EC |
| TBC1D32 | -0.61419 | 3.43E-05 | 0.00124 | EC |
| NME3 | 0.672326 | 3.46E-05 | 0.001246 | EC |
| SLC6A8 | 0.612987 | 3.48E-05 | 0.001252 | EC |
| GLIS2 | 0.912563 | 3.49E-05 | 0.001255 | EC |
| BLZF1 | -0.59194 | 3.51E-05 | 0.001256 | EC |
| PPIB | 0.606692 | 3.51E-05 | 0.001256 | EC |
| CHD9 | -0.62098 | 3.58E-05 | 0.001274 | EC |
| PRAF2 | 0.798502 | 3.58E-05 | 0.001274 | EC |
| SLC2A6 | 0.750143 | 3.59E-05 | 0.001274 | EC |
| METTL26 | 0.684828 | 3.68E-05 | 0.001297 | EC |
| JUND | 0.725074 | 3.75E-05 | 0.001318 | EC |
| FAU | 0.803752 | 3.77E-05 | 0.001318 | EC |
| CARNMT1 | -0.71305 | 3.79E-05 | 0.001322 | EC |
| ARHGAP33 | 0.656844 | 3.86E-05 | 0.001344 | EC |
| TIMM29 | 0.781445 | 3.88E-05 | 0.001346 | EC |
| TRMT61A | 0.670506 | 4.05E-05 | 0.001402 | EC |
| EVA1B | 1.475568 | 4.06E-05 | 0.001404 | EC |
| CRYAB | 0.964443 | 4.14E-05 | 0.001419 | EC |
| TAF1D | -0.63727 | 4.14E-05 | 0.001419 | EC |
| DDTL | 0.884693 | 4.17E-05 | 0.001423 | EC |
| RTN4RL2 | 1.280512 | 4.18E-05 | 0.001423 | EC |
| COMT | 0.790893 | 4.25E-05 | 0.001439 | EC |
| ZNF112 | -0.78317 | 4.34E-05 | 0.001452 | EC |
| RANGRF | 0.646716 | 4.33E-05 | 0.001452 | EC |
| DPM3 | 0.922033 | 4.32E-05 | 0.001452 | EC |
| CEP78 | -0.62072 | 4.36E-05 | 0.001456 | EC |
| LZTFL1 | -0.62751 | 4.37E-05 | 0.001457 | EC |
| RPS18 | 0.900506 | 4.41E-05 | 0.001466 | EC |
| PRKG2 | -0.7736 | 4.44E-05 | 0.00147 | EC |
| PCDH15 | -0.76791 | 4.45E-05 | 0.00147 | EC |

| | | | | |
|---|---|---|---|---|
| ATP13A2 | 0.614476 | 4.45E-05 | 0.00147 | EC |
| TMEM184B | 0.821425 | 4.44E-05 | 0.00147 | EC |
| NRXN2 | 0.602488 | 4.48E-05 | 0.001475 | EC |
| POLR3G | -0.8624 | 4.50E-05 | 0.001481 | EC |
| GPR20 | 2.291226 | 4.55E-05 | 0.001493 | EC |
| NDUFAF3 | 0.656352 | 4.61E-05 | 0.00151 | EC |
| SSB | -0.64114 | 4.68E-05 | 0.001522 | EC |
| SLC66A2 | 0.671618 | 4.70E-05 | 0.001528 | EC |
| CEP290 | -0.99313 | 4.74E-05 | 0.001537 | EC |
| NINJ1 | 0.84766 | 4.76E-05 | 0.001541 | EC |
| SNX16 | -0.72831 | 4.82E-05 | 0.001552 | EC |
| CORO7 | 0.796894 | 4.90E-05 | 0.001575 | EC |
| ETHE1 | 0.766718 | 4.95E-05 | 0.001582 | EC |
| SLC16A7 | -1.25379 | 4.95E-05 | 0.001582 | EC |
| RNPEPL1 | 0.763027 | 4.96E-05 | 0.001584 | EC |
| BCKDK | 0.608291 | 5.03E-05 | 0.0016 | EC |
| TMX3 | -0.60967 | 5.10E-05 | 0.001615 | EC |
| CXCL10 | 3.164955 | 5.09E-05 | 0.001615 | EC |
| FAM89B | 0.707346 | 5.13E-05 | 0.001618 | EC |
| ETAA1 | -0.68121 | 5.16E-05 | 0.001624 | EC |
| ZSWIM4 | 0.599099 | 5.46E-05 | 0.001699 | EC |
| ZNF891 | -0.63402 | 5.46E-05 | 0.001699 | EC |
| C2CD4D | 1.532242 | 5.42E-05 | 0.001699 | EC |
| POMZP3 | 0.749676 | 5.55E-05 | 0.001723 | EC |
| RPRM | 1.277396 | 5.57E-05 | 0.001724 | EC |
| FLRT1 | 0.614899 | 5.61E-05 | 0.001733 | EC |
| THAP11 | 0.824636 | 5.62E-05 | 0.001733 | EC |
| WDR18 | 0.695814 | 5.74E-05 | 0.001747 | EC |
| CDK6 | -1.02388 | 5.78E-05 | 0.001747 | EC |
| BET1 | -0.64773 | 5.80E-05 | 0.001747 | EC |
| AKAP9 | -0.87946 | 5.78E-05 | 0.001747 | EC |
| MTCH1 | 0.646159 | 5.77E-05 | 0.001747 | EC |
| CCDC102B | -0.76571 | 5.76E-05 | 0.001747 | EC |
| DPP7 | 0.674215 | 5.75E-05 | 0.001747 | EC |
| ZNF138 | -0.84494 | 5.78E-05 | 0.001747 | EC |
| EPHB3 | 0.607972 | 5.88E-05 | 0.001764 | EC |
| APLP1 | 0.791668 | 5.90E-05 | 0.001766 | EC |
| METTL14 | -0.64732 | 6.04E-05 | 0.001799 | EC |
| LARP4 | -0.58804 | 6.18E-05 | 0.001825 | EC |
| NDUFA13 | 0.929478 | 6.17E-05 | 0.001825 | EC |
| CYB5R3 | 0.768343 | 6.31E-05 | 0.001852 | EC |
| NUDT1 | 1.027743 | 6.33E-05 | 0.001852 | EC |
| ARHGEF40 | 0.669176 | 6.52E-05 | 0.001904 | EC |
| ADGRG3 | 2.028568 | 6.55E-05 | 0.001907 | EC |

| | | | | |
|---|---|---|---|---|
| IFI6 | 1.197568 | 6.59E-05 | 0.001907 | EC |
| SOX11 | -0.74867 | 6.58E-05 | 0.001907 | EC |
| PER1 | 0.591753 | 6.59E-05 | 0.001907 | EC |
| GNAI2 | 0.682723 | 6.61E-05 | 0.001908 | EC |
| AC006030.1 | 1.058133 | 6.79E-05 | 0.001951 | EC |
| RAB33A | 0.976292 | 6.85E-05 | 0.001963 | EC |
| ZNF761 | -0.76498 | 7.00E-05 | 0.001992 | EC |
| RPLP1 | 0.712006 | 7.04E-05 | 0.001997 | EC |
| PCBP1 | 0.585117 | 7.06E-05 | 0.002 | EC |
| CNNM3 | 0.582991 | 7.18E-05 | 0.002019 | EC |
| SASS6 | -0.62116 | 7.20E-05 | 0.002021 | EC |
| MAPK3 | 0.596438 | 7.39E-05 | 0.002062 | EC |
| SHISA4 | 0.644395 | 7.52E-05 | 0.00208 | EC |
| ZNF180 | -0.79913 | 7.58E-05 | 0.002086 | EC |
| RRBP1 | 0.600303 | 7.67E-05 | 0.002095 | EC |
| NCKIPSD | 0.594595 | 7.71E-05 | 0.002095 | EC |
| CERS1 | 0.97421 | 7.70E-05 | 0.002095 | EC |
| H1-10 | 0.584729 | 7.78E-05 | 0.002108 | EC |
| OTX1 | 1.006094 | 7.85E-05 | 0.002122 | EC |
| TAS2R4 | -0.91153 | 7.89E-05 | 0.002126 | EC |
| HLA-DRB1 | 1.929612 | 7.90E-05 | 0.002126 | EC |
| C3orf33 | -0.68802 | 8.01E-05 | 0.002148 | EC |
| NPTX2 | 2.15149 | 8.08E-05 | 0.002161 | EC |
| CSNK1G3 | -0.62043 | 8.08E-05 | 0.002161 | EC |
| VWA1 | 0.974698 | 8.13E-05 | 0.002168 | EC |
| ZNF703 | 0.789968 | 8.27E-05 | 0.002193 | EC |
| C5orf49 | 0.843822 | 8.28E-05 | 0.002193 | EC |
| UBALD2 | 0.752028 | 8.33E-05 | 0.002203 | EC |
| FHL3 | 0.64545 | 8.37E-05 | 0.002205 | EC |
| MAP2K3 | 0.690632 | 8.54E-05 | 0.002215 | EC |
| AIP | 0.654205 | 8.49E-05 | 0.002215 | EC |
| UQCC3 | 0.676612 | 8.49E-05 | 0.002215 | EC |
| DTX2 | 0.598138 | 8.57E-05 | 0.00222 | EC |
| MEGF8 | 0.660616 | 8.62E-05 | 0.002228 | EC |
| TMEM54 | 0.92143 | 8.69E-05 | 0.002239 | EC |
| ABCD1 | 0.860233 | 8.75E-05 | 0.002241 | EC |
| DCAF15 | 0.76135 | 8.73E-05 | 0.002241 | EC |
| UBD | 3.784245 | 8.83E-05 | 0.002257 | EC |
| EMX2 | 0.70846 | 8.86E-05 | 0.002262 | EC |
| PC | 0.644278 | 8.88E-05 | 0.002263 | EC |
| CHML | -0.74706 | 8.89E-05 | 0.002263 | EC |
| CASD1 | -0.67242 | 8.93E-05 | 0.002267 | EC |
| GTPBP6 | 0.632662 | 8.98E-05 | 0.002272 | EC |
| CEP57L1 | -0.75882 | 9.07E-05 | 0.00228 | EC |

| | | | | |
|---|---|---|---|---|
| S100A16 | 0.807492 | 9.06E-05 | 0.00228 | EC |
| PRPF39 | -0.58854 | 9.22E-05 | 0.002303 | EC |
| TMEM109 | 0.757771 | 9.24E-05 | 0.002305 | EC |
| AP2S1 | 0.815426 | 9.26E-05 | 0.002306 | EC |
| FXYD5 | 0.998281 | 9.30E-05 | 0.002308 | EC |
| KPNA5 | -0.69482 | 9.34E-05 | 0.002312 | EC |
| KIAA1328 | -0.58694 | 9.39E-05 | 0.002321 | EC |
| MICU2 | -0.59587 | 9.52E-05 | 0.002343 | EC |
| WDR17 | -0.66614 | 9.78E-05 | 0.002397 | EC |
| DNAJC25-GNG10 | -1.30246 | 9.83E-05 | 0.002404 | EC |
| ZZZ3 | -0.66561 | 9.86E-05 | 0.002408 | EC |
| DTX1 | 0.701709 | 9.90E-05 | 0.002411 | EC |
| C9orf24 | 0.920316 | 9.90E-05 | 0.002411 | EC |
| INHBA | 2.238262 | 0.0001 | 0.002435 | EC |
| RHBDD2 | 0.958249 | 0.000102 | 0.002479 | EC |
| ADORA1 | 0.825146 | 0.000104 | 0.002517 | EC |
| CCDC14 | -0.62615 | 0.000105 | 0.002517 | EC |
| HRH3 | 0.770504 | 0.000106 | 0.002525 | EC |
| WDR34 | 0.685495 | 0.000106 | 0.002525 | EC |
| PPIP5K2 | -0.60752 | 0.000105 | 0.002525 | EC |
| ATP5ME | 0.833086 | 0.000106 | 0.002525 | EC |
| AC012184.2 | 1.370426 | 0.000106 | 0.002525 | EC |
| ROCK2 | -0.6132 | 0.000107 | 0.00254 | EC |
| ABHD13 | -0.69252 | 0.000108 | 0.002544 | EC |
| TNFAIP2 | 1.010113 | 0.000108 | 0.002557 | EC |
| RTN4R | 1.078402 | 0.000109 | 0.002569 | EC |
| TUBB4A | 0.776444 | 0.00011 | 0.002577 | EC |
| KCNT2 | -0.67947 | 0.00011 | 0.002577 | EC |
| ZMIZ1 | 0.584655 | 0.000111 | 0.002597 | EC |
| POLR2J | 0.655256 | 0.000113 | 0.002612 | EC |
| MFSD9 | -0.68921 | 0.000112 | 0.002612 | EC |
| GYPC | 1.024979 | 0.000112 | 0.002612 | EC |
| MSTN | -0.88127 | 0.000112 | 0.002612 | EC |
| C20orf27 | 0.660845 | 0.000114 | 0.002637 | EC |
| CRLF1 | 1.297147 | 0.000115 | 0.002666 | EC |
| PLGLB1 | -0.92974 | 0.000117 | 0.002697 | EC |
| STK17B | -0.87229 | 0.000117 | 0.0027 | EC |
| PHPT1 | 0.701447 | 0.000118 | 0.002708 | EC |
| OSBPL8 | -0.58407 | 0.000118 | 0.002708 | EC |
| NFYB | -0.75208 | 0.000119 | 0.002718 | EC |
| ZFYVE16 | -0.77191 | 0.000119 | 0.002723 | EC |
| PM20D2 | -0.7501 | 0.000119 | 0.002723 | EC |
| TBK1 | -0.59078 | 0.00012 | 0.002727 | EC |
| CD7 | 1.913041 | 0.00012 | 0.002735 | EC |

| | | | | |
|---|---|---|---|---|
| NR2F1 | 0.649233 | 0.00012 | 0.002738 | EC |
| ABCA5 | -0.77211 | 0.000122 | 0.002757 | EC |
| VPS13A | -0.681 | 0.000123 | 0.00278 | EC |
| ABHD14A | 0.716002 | 0.000125 | 0.002805 | EC |
| KXD1 | 0.595341 | 0.000126 | 0.002819 | EC |
| PLTP | 0.908615 | 0.000127 | 0.002833 | EC |
| ZDHHC1 | 0.611349 | 0.000127 | 0.002833 | EC |
| TST | 0.810597 | 0.000127 | 0.002836 | EC |
| TRDMT1 | -0.66155 | 0.000129 | 0.002869 | EC |
| ZNF708 | -0.69684 | 0.00013 | 0.002869 | EC |
| CHCHD2 | 0.63221 | 0.000131 | 0.002885 | EC |
| TMEM198 | 0.789675 | 0.000131 | 0.002887 | EC |
| RNF181 | 0.589708 | 0.000132 | 0.002898 | EC |
| UBB | 0.634089 | 0.000132 | 0.002898 | EC |
| PTRH1 | 0.749442 | 0.000132 | 0.002898 | EC |
| TRAPPC6B | -0.58288 | 0.000133 | 0.002904 | EC |
| IFITM2 | 1.221141 | 0.000134 | 0.002917 | EC |
| ROGDI | 0.659719 | 0.000134 | 0.002928 | EC |
| CHCHD10 | 0.825351 | 0.000134 | 0.002928 | EC |
| CHST7 | 1.060592 | 0.000135 | 0.002942 | EC |
| KATNBL1 | -0.61222 | 0.000136 | 0.002946 | EC |
| IRF2BPL | 0.586922 | 0.000136 | 0.002947 | EC |
| EFR3A | -0.6601 | 0.000138 | 0.002981 | EC |
| ZMYND15 | 1.064257 | 0.000138 | 0.002981 | EC |
| HCN2 | 0.714955 | 0.000139 | 0.002985 | EC |
| NDUFA1 | 0.850375 | 0.000139 | 0.002985 | EC |
| SCAI | -0.72584 | 0.000139 | 0.002985 | EC |
| DIAPH2 | -0.76837 | 0.000141 | 0.002996 | EC |
| AGL | -0.59843 | 0.00014 | 0.002996 | EC |
| HTR1F | -1.02031 | 0.00014 | 0.002996 | EC |
| LRRC69 | -1.04187 | 0.000141 | 0.002996 | EC |
| VWC2 | -0.69782 | 0.000142 | 0.003009 | EC |
| H4C8 | 0.751711 | 0.000142 | 0.003011 | EC |
| FGF7 | -2.19293 | 0.000142 | 0.003014 | EC |
| TSEN15 | -0.99128 | 0.000143 | 0.00302 | EC |
| ANKRD36 | -0.75703 | 0.000143 | 0.003024 | EC |
| CITED4 | 0.871854 | 0.000143 | 0.003024 | EC |
| RELB | 1.258741 | 0.000144 | 0.003029 | EC |
| TTC26 | -0.59068 | 0.000146 | 0.003036 | EC |
| KIF20B | -0.85127 | 0.000146 | 0.003036 | EC |
| COX7A1 | 1.019766 | 0.000145 | 0.003036 | EC |
| WDR86 | 1.042661 | 0.000146 | 0.003036 | EC |
| ADAT2 | -0.79839 | 0.000146 | 0.003036 | EC |
| CD177 | 1.940966 | 0.000148 | 0.003054 | EC |

| | | | | |
|---|---|---|---|---|
| NME5 | -0.74422 | 0.000148 | 0.003056 | EC |
| LLGL1 | 0.734877 | 0.00015 | 0.003075 | EC |
| B3GNT9 | 0.725662 | 0.000152 | 0.003109 | EC |
| NXT1 | 0.753784 | 0.000153 | 0.003117 | EC |
| GLMN | -0.74521 | 0.000153 | 0.003117 | EC |
| GAL3ST1 | 0.876114 | 0.000153 | 0.00312 | EC |
| NDUFB11 | 0.715454 | 0.000153 | 0.003121 | EC |
| THOC1 | -0.69728 | 0.000155 | 0.003129 | EC |
| FBLL1 | 0.848667 | 0.000155 | 0.003134 | EC |
| LAMB3 | 1.430301 | 0.000156 | 0.003139 | EC |
| NECAB1 | -0.91498 | 0.000158 | 0.003181 | EC |
| MYH9 | 0.615361 | 0.000159 | 0.00319 | EC |
| LAMTOR4 | 0.640715 | 0.000159 | 0.003196 | EC |
| MRPL53 | 0.808218 | 0.00016 | 0.003199 | EC |
| PPP1R13L | 0.785959 | 0.00016 | 0.003201 | EC |
| MEGF6 | 0.889317 | 0.000161 | 0.003209 | EC |
| NDUFA2 | 0.7449 | 0.000162 | 0.00323 | EC |
| ECH1 | 0.653801 | 0.000163 | 0.003235 | EC |
| ANKRD26 | -0.88545 | 0.000163 | 0.003235 | EC |
| CREBRF | -0.58366 | 0.000163 | 0.003235 | EC |
| PHOSPHO2 | -0.85753 | 0.000164 | 0.003241 | EC |
| RWDD4 | -0.65853 | 0.000165 | 0.003241 | EC |
| SMIM10L1 | -0.73506 | 0.000164 | 0.003241 | EC |
| SSX2IP | -0.7135 | 0.000166 | 0.003251 | EC |
| TRMT112 | 0.638026 | 0.000167 | 0.003264 | EC |
| MIS18BP1 | -0.79527 | 0.000168 | 0.003283 | EC |
| ZNF133 | -0.59051 | 0.000169 | 0.00329 | EC |
| FAM133A | -0.83827 | 0.000169 | 0.00329 | EC |
| KIF7 | 0.647123 | 0.00017 | 0.003295 | EC |
| DST | -0.62606 | 0.00017 | 0.0033 | EC |
| SPOPL | -0.81285 | 0.000173 | 0.003339 | EC |
| NDUFB7 | 0.897959 | 0.000174 | 0.003343 | EC |
| BTBD8 | -0.63667 | 0.000174 | 0.003343 | EC |
| VIPR2 | -0.72514 | 0.000175 | 0.003356 | EC |
| CRYBG3 | -0.65033 | 0.000176 | 0.003362 | EC |
| CHST1 | 0.653346 | 0.000178 | 0.003404 | EC |
| RMI1 | -0.6665 | 0.00018 | 0.00343 | EC |
| H4C5 | 1.016011 | 0.00018 | 0.00343 | EC |
| ITGB4 | 1.02835 | 0.000182 | 0.003458 | EC |
| TMEM50B | -0.65507 | 0.000189 | 0.003569 | EC |
| H2AC4 | 1.467603 | 0.000189 | 0.003569 | EC |
| DNAJC2 | -0.62491 | 0.000191 | 0.003601 | EC |
| KRR1 | -0.62406 | 0.000192 | 0.003601 | EC |
| GRIK5 | 0.590725 | 0.000193 | 0.003617 | EC |

| | | | | |
|---|---|---|---|---|
| TSGA10 | -0.73207 | 0.000194 | 0.003627 | EC |
| FBLN5 | 0.864817 | 0.000194 | 0.003627 | EC |
| PHRF1 | 0.651472 | 0.000195 | 0.003634 | EC |
| MOSPD3 | 0.645664 | 0.000196 | 0.003642 | EC |
| FAM117A | 0.654375 | 0.000198 | 0.003686 | EC |
| H2BC12 | 1.059654 | 0.000199 | 0.003689 | EC |
| H3C10 | 1.60592 | 0.000199 | 0.003689 | EC |
| GPAA1 | 0.605417 | 0.0002 | 0.003695 | EC |
| ZNF423 | 0.833747 | 0.0002 | 0.003696 | EC |
| CNTFR | 0.86072 | 0.000201 | 0.003714 | EC |
| SOX15 | 1.173756 | 0.000202 | 0.003716 | EC |
| TCF7L1 | 0.74156 | 0.000202 | 0.003721 | EC |
| LGR6 | 1.409053 | 0.000204 | 0.003743 | EC |
| CEP135 | -0.68243 | 0.000205 | 0.003747 | EC |
| ACTG1 | 0.747336 | 0.000205 | 0.003754 | EC |
| ZNF577 | -0.64321 | 0.000209 | 0.003804 | EC |
| AZI2 | -0.60286 | 0.000213 | 0.003867 | EC |
| TTC14 | -0.64747 | 0.000215 | 0.003885 | EC |
| VAMP5 | 0.972293 | 0.000215 | 0.003885 | EC |
| CPNE6 | 0.66562 | 0.000217 | 0.00391 | EC |
| FBXL7 | 0.669135 | 0.00022 | 0.003928 | EC |
| EEF1G | 0.873392 | 0.000221 | 0.003928 | EC |
| RPF2 | -0.72105 | 0.000222 | 0.003945 | EC |
| LGALS8 | -0.68097 | 0.000224 | 0.00397 | EC |
| GIN1 | -0.73394 | 0.000224 | 0.00397 | EC |
| C4B | 1.446947 | 0.000224 | 0.00397 | EC |
| ANO7 | 1.075923 | 0.000226 | 0.003995 | EC |
| FXYD1 | 0.853743 | 0.000226 | 0.003995 | EC |
| ZNF503 | 0.746071 | 0.000227 | 0.004003 | EC |
| TP53I13 | 0.68067 | 0.000229 | 0.00403 | EC |
| GRN | 0.85832 | 0.000229 | 0.004031 | EC |
| ACY3 | 2.20294 | 0.00023 | 0.004032 | EC |
| RAB5C | 0.58727 | 0.000232 | 0.004052 | EC |
| TTYH3 | 0.632638 | 0.000232 | 0.004052 | EC |
| ALDOA | 0.628 | 0.000233 | 0.004063 | EC |
| MICU3 | -0.70729 | 0.000234 | 0.004075 | EC |
| DUSP2 | 0.979692 | 0.000234 | 0.004076 | EC |
| ARID4B | -0.64731 | 0.000238 | 0.004125 | EC |
| REEP4 | 0.713734 | 0.000242 | 0.004179 | EC |
| HSPB2 | 0.906365 | 0.000244 | 0.004195 | EC |
| ENDOG | 0.824333 | 0.000246 | 0.004208 | EC |
| FLNC | 1.144712 | 0.00025 | 0.004241 | EC |
| C1QL1 | 0.895201 | 0.00025 | 0.004241 | EC |
| CCDC7 | -0.72064 | 0.00025 | 0.004241 | EC |

| | | | | |
|---|---|---|---|---|
| UCHL5 | -0.66793 | 0.000252 | 0.004264 | EC |
| MRPS24 | 0.707539 | 0.000254 | 0.004274 | EC |
| MDK | 1.168178 | 0.000254 | 0.004274 | EC |
| TAS2R13 | -0.85509 | 0.000254 | 0.004274 | EC |
| YBX1 | 0.618449 | 0.000255 | 0.004287 | EC |
| FAM126A | -0.69535 | 0.000261 | 0.004366 | EC |
| MAP9 | -0.78134 | 0.000264 | 0.0044 | EC |
| THAP5 | -0.76147 | 0.000265 | 0.004406 | EC |
| CAMK4 | -0.73839 | 0.000267 | 0.004422 | EC |
| PIBF1 | -0.68979 | 0.00027 | 0.004455 | EC |
| PCGF1 | 0.626788 | 0.000271 | 0.004455 | EC |
| TMUB1 | 0.623744 | 0.000271 | 0.004455 | EC |
| CARD19 | 0.68575 | 0.000271 | 0.004455 | EC |
| MLST8 | 0.636867 | 0.000271 | 0.004455 | EC |
| URGCP-MRPS24 | 2.961798 | 0.000272 | 0.004467 | EC |
| GAL3ST3 | 0.751224 | 0.000274 | 0.004486 | EC |
| PADI2 | 0.691217 | 0.000276 | 0.004508 | EC |
| ANKRD36C | -0.62982 | 0.000276 | 0.004508 | EC |
| CLU | 0.884162 | 0.000278 | 0.004527 | EC |
| CRTAC1 | 0.816558 | 0.000281 | 0.004543 | EC |
| OPRL1 | 0.683356 | 0.000283 | 0.004572 | EC |
| IPMK | -0.58695 | 0.000284 | 0.004581 | EC |
| ICAM1 | 1.595257 | 0.000285 | 0.004587 | EC |
| SLC52A2 | 0.650243 | 0.000287 | 0.004613 | EC |
| GADD45G | 1.007919 | 0.00029 | 0.004645 | EC |
| MRPS34 | 0.595564 | 0.000291 | 0.004656 | EC |
| PUSL1 | 0.615431 | 0.000292 | 0.004663 | EC |
| TUBB4B | 0.697234 | 0.000292 | 0.004663 | EC |
| PLAG1 | -0.66909 | 0.000293 | 0.00468 | EC |
| RPS2 | 0.692299 | 0.000294 | 0.004687 | EC |
| N4BP2 | -0.63658 | 0.000295 | 0.004699 | EC |
| GET3 | 0.676907 | 0.000296 | 0.004707 | EC |
| APH1A | 0.680551 | 0.000299 | 0.004721 | EC |
| TMEM256 | 0.715708 | 0.0003 | 0.004732 | EC |
| RTL8A | 0.709836 | 0.000302 | 0.004757 | EC |
| GPC4 | 0.60242 | 0.000304 | 0.004787 | EC |
| NKAIN2 | -0.73621 | 0.000306 | 0.004792 | EC |
| RARA | 0.589614 | 0.000307 | 0.004795 | EC |
| UQCR10 | 0.628083 | 0.000307 | 0.004795 | EC |
| SIKE1 | -0.58733 | 0.000309 | 0.004809 | EC |
| SLC25A1 | 0.636117 | 0.000313 | 0.004851 | EC |
| PPIG | -0.6118 | 0.000313 | 0.004851 | EC |
| AMY2B | -0.59433 | 0.000313 | 0.004851 | EC |
| PGAP6 | 0.581142 | 0.000314 | 0.004852 | EC |

| | | | | |
|---|---|---|---|---|
| CCDC88A | -0.62691 | 0.000315 | 0.004857 | EC |
| CST3 | 0.869764 | 0.000317 | 0.00488 | EC |
| MARCHF7 | -0.64971 | 0.00032 | 0.004913 | EC |
| FAM171A2 | 0.807293 | 0.000321 | 0.004919 | EC |
| FSTL3 | 1.412095 | 0.000324 | 0.004951 | EC |
| AMN1 | -0.5855 | 0.000326 | 0.00498 | EC |
| FOXJ1 | 1.700272 | 0.000328 | 0.005001 | EC |
| TVP23B | -0.74071 | 0.000331 | 0.005016 | EC |
| ZNF141 | -0.7593 | 0.000333 | 0.005037 | EC |
| PYCR2 | 0.599227 | 0.000335 | 0.005045 | EC |
| SRM | 0.631731 | 0.000336 | 0.005051 | EC |
| CBR4 | -0.63662 | 0.000336 | 0.005051 | EC |
| H4C3 | 0.82966 | 0.000336 | 0.005051 | EC |
| AC069503.2 | -0.91041 | 0.000337 | 0.005058 | EC |
| REV3L | -0.62013 | 0.000339 | 0.005082 | EC |
| ZBTB1 | -0.61873 | 0.000341 | 0.005095 | EC |
| PRKAA2 | -0.70505 | 0.000341 | 0.005095 | EC |
| ZNF107 | -0.77425 | 0.000341 | 0.005095 | EC |
| MXRA8 | 0.981808 | 0.000342 | 0.0051 | EC |
| PCDHA10 | -0.58812 | 0.000345 | 0.005124 | EC |
| LMO1 | 1.062041 | 0.000346 | 0.005132 | EC |
| HSPB1 | 1.367199 | 0.000349 | 0.005166 | EC |
| PGGT1B | -0.60916 | 0.000354 | 0.005206 | EC |
| FSCN1 | 0.615449 | 0.000355 | 0.005217 | EC |
| HS3ST2 | 1.68633 | 0.000356 | 0.005217 | EC |
| C4orf33 | -0.66602 | 0.000356 | 0.005217 | EC |
| CEP83 | -0.74774 | 0.000355 | 0.005217 | EC |
| ESCO1 | -0.67519 | 0.000357 | 0.005221 | EC |
| TMEM161A | 0.644427 | 0.00036 | 0.005258 | EC |
| ICE2 | -0.60671 | 0.000361 | 0.00527 | EC |
| AGO3 | -0.62097 | 0.000361 | 0.005271 | EC |
| SLC16A11 | 0.875202 | 0.000363 | 0.005288 | EC |
| GZMM | 2.001653 | 0.000364 | 0.00529 | EC |
| VPS37D | 0.726978 | 0.000365 | 0.005295 | EC |
| GPATCH3 | 0.582411 | 0.000365 | 0.005297 | EC |
| ATP6V0C | 0.625791 | 0.000367 | 0.005314 | EC |
| C1QTNF4 | 0.926055 | 0.000368 | 0.005317 | EC |
| SLC44A5 | -0.71469 | 0.000369 | 0.005329 | EC |
| NDUFS5 | 0.82674 | 0.000371 | 0.005335 | EC |
| LRRTM4 | -0.8358 | 0.000372 | 0.005348 | EC |
| PCMTD2 | -0.59808 | 0.000373 | 0.005363 | EC |
| RAB7B | 1.045519 | 0.000374 | 0.005368 | EC |
| ZNF92 | -0.63447 | 0.000375 | 0.005374 | EC |
| ZNF326 | -0.72974 | 0.000378 | 0.005379 | EC |

| | | | | |
|---|---|---|---|---|
| IFITM10 | 0.71674 | 0.000378 | 0.005379 | EC |
| SSC5D | 0.919727 | 0.00038 | 0.005406 | EC |
| TRAK2 | -0.74936 | 0.000381 | 0.005413 | EC |
| ATP6V0B | 0.624508 | 0.000382 | 0.005419 | EC |
| FGF9 | -1.15172 | 0.000383 | 0.005421 | EC |
| NPB | 1.071123 | 0.000385 | 0.005432 | EC |
| CNTLN | -0.61444 | 0.000386 | 0.005435 | EC |
| LMNA | 0.668942 | 0.000388 | 0.005457 | EC |
| DNAJB14 | -0.64545 | 0.000389 | 0.005466 | EC |
| ZDBF2 | -0.65909 | 0.000394 | 0.005518 | EC |
| AGFG1 | 0.616516 | 0.000396 | 0.005529 | EC |
| ZNF638 | -0.61889 | 0.000399 | 0.005554 | EC |
| ESF1 | -0.63032 | 0.000399 | 0.005554 | EC |
| SWT1 | -0.66669 | 0.0004 | 0.005566 | EC |
| RPL36AL | 0.670656 | 0.000401 | 0.005571 | EC |
| ZNF37A | -0.63025 | 0.000403 | 0.005578 | EC |
| HAGHL | 0.680628 | 0.000403 | 0.005578 | EC |
| HMGN5 | -0.71481 | 0.000404 | 0.005586 | EC |
| SLC35E4 | 0.678765 | 0.000407 | 0.005606 | EC |
| CBR3 | 0.841585 | 0.000412 | 0.005662 | EC |
| FGF14 | -0.71047 | 0.000415 | 0.005669 | EC |
| NR1D1 | 0.865709 | 0.000414 | 0.005669 | EC |
| NAT8L | 0.603967 | 0.000413 | 0.005669 | EC |
| MPV17L2 | 0.806202 | 0.000413 | 0.005669 | EC |
| WFIKKN1 | 0.983856 | 0.000419 | 0.005704 | EC |
| POTEG | -2.41164 | 0.00042 | 0.005718 | EC |
| REX1BD | 0.706054 | 0.000423 | 0.005732 | EC |
| INTS1 | 0.642458 | 0.000423 | 0.005732 | EC |
| ELFN1 | 0.793205 | 0.00043 | 0.005813 | EC |
| ST3GAL6 | -0.65318 | 0.000432 | 0.005824 | EC |
| ZNF654 | -0.64757 | 0.000431 | 0.005824 | EC |
| ZBTB41 | -0.61108 | 0.000432 | 0.005824 | EC |
| MZT1 | -0.86547 | 0.000436 | 0.005851 | EC |
| SORCS2 | 0.63382 | 0.000438 | 0.005866 | EC |
| ACTB | 0.635349 | 0.000439 | 0.005876 | EC |
| ZNF605 | -0.63275 | 0.00044 | 0.005879 | EC |
| PGD | 0.588047 | 0.000443 | 0.005912 | EC |
| FGFRL1 | 0.950534 | 0.000444 | 0.005912 | EC |
| PSAP | 0.739056 | 0.000447 | 0.00595 | EC |
| MT3 | 0.929061 | 0.000449 | 0.00597 | EC |
| ABHD17A | 0.644183 | 0.00045 | 0.005972 | EC |
| TNFSF14 | 3.011339 | 0.000452 | 0.005991 | EC |
| ZNF514 | -0.67778 | 0.000454 | 0.006018 | EC |
| PHGDH | 0.743137 | 0.000455 | 0.00602 | EC |

| | | | | |
|---|---|---|---|---|
| NAALAD2 | -0.81661 | 0.000462 | 0.006087 | EC |
| NR2C1 | -0.58886 | 0.000461 | 0.006087 | EC |
| RPLP2 | 0.668471 | 0.000464 | 0.006087 | EC |
| PSMB8 | 0.781645 | 0.000463 | 0.006087 | EC |
| VAMP4 | -0.77412 | 0.000466 | 0.006102 | EC |
| MDH2 | 0.61576 | 0.000466 | 0.006102 | EC |
| MAPK8IP3 | 0.58265 | 0.000467 | 0.006113 | EC |
| HERC4 | -0.60535 | 0.000468 | 0.006115 | EC |
| CDC42EP1 | 0.925533 | 0.000469 | 0.006116 | EC |
| PLIN1 | 1.113319 | 0.000471 | 0.006133 | EC |
| ZNF644 | -0.6796 | 0.000477 | 0.006186 | EC |
| DOHH | 0.63182 | 0.000476 | 0.006186 | EC |
| DIO2 | -1.06968 | 0.000481 | 0.006234 | EC |
| RPL41 | 1.212627 | 0.000482 | 0.006238 | EC |
| LTBP4 | 0.671762 | 0.000484 | 0.006244 | EC |
| LNPK | -0.63192 | 0.000484 | 0.006244 | EC |
| CXADR | -0.62611 | 0.000484 | 0.006244 | EC |
| LYPLA1 | -0.6741 | 0.000488 | 0.006276 | EC |
| TMEM160 | 0.828506 | 0.000488 | 0.006276 | EC |
| ADAMTS8 | 0.667428 | 0.000489 | 0.006286 | EC |
| PLP2 | 0.956742 | 0.00049 | 0.006293 | EC |
| SREK1IP1 | -0.63249 | 0.000493 | 0.00631 | EC |
| METRNL | 0.651239 | 0.000494 | 0.006319 | EC |
| DNPH1 | 0.691971 | 0.000496 | 0.006336 | EC |
| ABCA10 | -0.65755 | 0.000501 | 0.006376 | EC |
| APC2 | 0.593055 | 0.000503 | 0.006379 | EC |
| ERO1A | -0.65539 | 0.000504 | 0.006379 | EC |
| TSPAN4 | 0.715338 | 0.000504 | 0.006382 | EC |
| TXNL1 | -0.63524 | 0.000515 | 0.006478 | EC |
| CLSTN1 | 0.598166 | 0.000523 | 0.006554 | EC |
| FTL | 0.76381 | 0.000524 | 0.006561 | EC |
| GLRA3 | -1.08088 | 0.000525 | 0.006572 | EC |
| NPC2 | 0.798837 | 0.000526 | 0.006579 | EC |
| P2RY11 | 0.845681 | 0.000529 | 0.006609 | EC |
| C9orf85 | -0.64323 | 0.000531 | 0.006623 | EC |
| SP7 | 1.389373 | 0.000532 | 0.006632 | EC |
| RNF145 | -0.71427 | 0.000533 | 0.006641 | EC |
| SYNGR2 | 0.779893 | 0.000534 | 0.006641 | EC |
| LUC7L3 | -0.61397 | 0.000534 | 0.006645 | EC |
| ERGIC2 | -0.64311 | 0.000535 | 0.006653 | EC |
| ZAP70 | 1.07951 | 0.000537 | 0.006664 | EC |
| MTLN | 0.983915 | 0.000537 | 0.006664 | EC |
| RPL38 | 0.600026 | 0.000538 | 0.006666 | EC |
| LRRD1 | -1.19583 | 0.000545 | 0.006752 | EC |

| | | | | |
|---|---|---|---|---|
| GSN | 0.701137 | 0.000548 | 0.006772 | EC |
| APOE | 0.825799 | 0.000556 | 0.006837 | EC |
| DNAH14 | -0.75347 | 0.000558 | 0.006849 | EC |
| ADRA2C | 0.768355 | 0.000561 | 0.006864 | EC |
| ZNF33A | -0.72131 | 0.000561 | 0.006864 | EC |
| PGAP1 | -0.72699 | 0.000563 | 0.006878 | EC |
| C16orf91 | 0.776987 | 0.000564 | 0.006878 | EC |
| LPAR6 | -1.14677 | 0.000565 | 0.006883 | EC |
| MTERF1 | -0.75791 | 0.000569 | 0.006912 | EC |
| PDLIM4 | 0.961829 | 0.000571 | 0.006917 | EC |
| MIGA1 | -0.61234 | 0.000571 | 0.006917 | EC |
| AC093525.2 | 0.671202 | 0.00057 | 0.006917 | EC |
| CISD2 | -0.65167 | 0.000572 | 0.006926 | EC |
| MFSD10 | 0.659668 | 0.000579 | 0.006988 | EC |
| SLC38A3 | 0.905948 | 0.00058 | 0.006994 | EC |
| TTLL7 | -0.70336 | 0.000583 | 0.007021 | EC |
| LINC00634 | 0.748753 | 0.000586 | 0.007036 | EC |
| KDM6B | 0.796539 | 0.000587 | 0.00704 | EC |
| JUP | 0.799959 | 0.000591 | 0.007083 | EC |
| FUT9 | -0.70367 | 0.000595 | 0.007114 | EC |
| BTBD19 | 0.809049 | 0.000596 | 0.00712 | EC |
| PPP4R2 | -0.71197 | 0.000598 | 0.007134 | EC |
| FBXO44 | 0.64794 | 0.0006 | 0.007147 | EC |
| TMEM106B | -0.69332 | 0.000604 | 0.007178 | EC |
| BCOR | 0.654802 | 0.000605 | 0.007185 | EC |
| TMEM147 | 0.763924 | 0.00061 | 0.007204 | EC |
| C1QL2 | 1.098035 | 0.000608 | 0.007204 | EC |
| CDH23 | 0.794283 | 0.000611 | 0.007208 | EC |
| SKA2 | -0.7839 | 0.000611 | 0.007208 | EC |
| ARHGAP39 | 0.601438 | 0.000613 | 0.007213 | EC |
| AC002996.1 | 0.753451 | 0.000613 | 0.007213 | EC |
| COLCA2 | 0.735865 | 0.000615 | 0.007226 | EC |
| EIF4EBP3 | 1.244229 | 0.00062 | 0.007262 | EC |
| CFAP126 | 1.829713 | 0.000624 | 0.007302 | EC |
| ARHGAP5 | -0.94508 | 0.000627 | 0.007313 | EC |
| LGALS3BP | 0.760559 | 0.000634 | 0.007361 | EC |
| PURA | -0.75531 | 0.000635 | 0.007361 | EC |
| B4GALT6 | -0.6609 | 0.000636 | 0.007375 | EC |
| SLC25A28 | 0.827259 | 0.000638 | 0.007375 | EC |
| HR | 0.752916 | 0.000638 | 0.007375 | EC |
| NXF2 | -1.84135 | 0.000646 | 0.007445 | EC |
| LUZP2 | -0.74164 | 0.000651 | 0.007489 | EC |
| VPS26A | -0.91499 | 0.000653 | 0.007503 | EC |
| CEND1 | 0.643667 | 0.000655 | 0.00752 | EC |

| | | | | |
|---|---|---|---|---|
| ADPRHL1 | 1.24768 | 0.000656 | 0.007524 | EC |
| SERTAD1 | 1.047599 | 0.000657 | 0.007524 | EC |
| ERCC8 | -0.61664 | 0.000657 | 0.007524 | EC |
| TEX15 | -0.83588 | 0.000658 | 0.007524 | EC |
| ZNF181 | -0.62554 | 0.000659 | 0.007524 | EC |
| NOC4L | 0.682358 | 0.000661 | 0.00754 | EC |
| NGFR | 1.292283 | 0.000673 | 0.007651 | EC |
| ZNF182 | -0.68596 | 0.000679 | 0.007694 | EC |
| AURKAIP1 | 0.602636 | 0.000679 | 0.007694 | EC |
| ORAI1 | 1.114213 | 0.000679 | 0.007694 | EC |
| SNRNP48 | -0.61203 | 0.00068 | 0.007701 | EC |
| CENPJ | -0.70261 | 0.000682 | 0.007707 | EC |
| SDR16C5 | -0.93932 | 0.000682 | 0.007707 | EC |
| ZNF519 | -0.68529 | 0.000682 | 0.007707 | EC |
| NUDT12 | -0.6129 | 0.000684 | 0.007723 | EC |
| KRTCAP2 | 0.584279 | 0.000685 | 0.007731 | EC |
| GCNT2 | -0.67413 | 0.000687 | 0.007735 | EC |
| MYLK | 0.611509 | 0.000688 | 0.007739 | EC |
| HSD17B10 | 0.651437 | 0.000688 | 0.007739 | EC |
| IFITM1 | 1.211059 | 0.000691 | 0.007763 | EC |
| TMEM132A | 0.733118 | 0.000692 | 0.007771 | EC |
| BAG3 | 0.891553 | 0.000697 | 0.007809 | EC |
| DCC | -0.60847 | 0.000698 | 0.007813 | EC |
| RAB9B | -0.69651 | 0.000702 | 0.007849 | EC |
| DAPK3 | 0.744564 | 0.000703 | 0.007849 | EC |
| ULK1 | 0.582337 | 0.000708 | 0.007893 | EC |
| PPP1R1B | 0.683175 | 0.000716 | 0.007947 | EC |
| ENTPD2 | 1.047046 | 0.000736 | 0.008102 | EC |
| TBC1D8B | -0.74098 | 0.000736 | 0.008102 | EC |
| SPNS1 | 0.590568 | 0.000735 | 0.008102 | EC |
| ZSCAN22 | 0.601477 | 0.000735 | 0.008102 | EC |
| GJC2 | 0.933041 | 0.000736 | 0.008102 | EC |
| TAX1BP3 | 0.727382 | 0.000737 | 0.008102 | EC |
| EGR1 | 1.156147 | 0.000743 | 0.008165 | EC |
| ADIRF | 0.964464 | 0.000744 | 0.008167 | EC |
| ATPSCKMT | -0.63047 | 0.000756 | 0.008237 | EC |
| PRIM2 | -0.59282 | 0.000765 | 0.008321 | EC |
| SEPTIN1 | 0.89959 | 0.000766 | 0.008323 | EC |
| ELOB | 0.612047 | 0.000769 | 0.008341 | EC |
| MT-ND6 | -1.0628 | 0.000772 | 0.008371 | EC |
| HMG20B | 0.583661 | 0.000775 | 0.008386 | EC |
| VIM | 0.964916 | 0.000777 | 0.00839 | EC |
| MFSD3 | 0.753514 | 0.000778 | 0.008394 | EC |
| TKT | 0.58792 | 0.000779 | 0.008401 | EC |

| | | | | |
|---|---|---|---|---|
| UNC93B1 | 0.693904 | 0.000783 | 0.008424 | EC |
| CD68 | 1.225134 | 0.000788 | 0.00846 | EC |
| FOXN2 | -0.71137 | 0.000788 | 0.00846 | EC |
| ABHD4 | 0.677835 | 0.000794 | 0.008494 | EC |
| RHPN1 | 0.595455 | 0.000795 | 0.008497 | EC |
| SLC22A6 | 2.047352 | 0.000799 | 0.008533 | EC |
| MRPL12 | 0.615375 | 0.000799 | 0.008533 | EC |
| AC008073.3 | -0.77336 | 0.000802 | 0.008554 | EC |
| ZNF100 | -0.72227 | 0.000805 | 0.00858 | EC |
| SLC12A4 | 0.689686 | 0.00081 | 0.008599 | EC |
| C5orf63 | -0.79532 | 0.000809 | 0.008599 | EC |
| YIF1A | 0.626641 | 0.00081 | 0.008599 | EC |
| KLHL25 | 0.613594 | 0.00081 | 0.008599 | EC |
| CCDC168 | -1.22133 | 0.000819 | 0.008664 | EC |
| LCN12 | 1.07869 | 0.000828 | 0.00874 | EC |
| SYCP2 | -0.70943 | 0.000828 | 0.00874 | EC |
| RPL37A | 0.676899 | 0.000828 | 0.00874 | EC |
| RAMACL | -0.72304 | 0.000832 | 0.008762 | EC |
| ACTC1 | 1.831215 | 0.000844 | 0.008872 | EC |
| COL22A1 | 1.630891 | 0.000844 | 0.008874 | EC |
| SGTB | -0.67721 | 0.00085 | 0.008922 | EC |
| TOMM34 | 0.691108 | 0.000851 | 0.008925 | EC |
| CTF1 | 0.843997 | 0.000853 | 0.008933 | EC |
| PCK2 | 0.613545 | 0.000856 | 0.008952 | EC |
| LMCD1 | 0.843937 | 0.000857 | 0.008963 | EC |
| IL1R2 | 2.538855 | 0.000859 | 0.008977 | EC |
| DHFR | -0.77473 | 0.00086 | 0.008982 | EC |
| NDUFA6 | 0.593873 | 0.000864 | 0.009006 | EC |
| ZNF483 | -0.62369 | 0.000866 | 0.00901 | EC |
| AP5M1 | -0.60925 | 0.00087 | 0.009048 | EC |
| PLS1 | -0.75029 | 0.000876 | 0.009091 | EC |
| SUN2 | 0.620695 | 0.000882 | 0.009095 | EC |
| PIEZO1 | 0.631761 | 0.000883 | 0.009095 | EC |
| FSD1L | -0.69128 | 0.000881 | 0.009095 | EC |
| TOX2 | 0.705264 | 0.000879 | 0.009095 | EC |
| APOD | 0.929537 | 0.000878 | 0.009095 | EC |
| NCOR2 | 0.602902 | 0.00088 | 0.009095 | EC |
| ZFP62 | -0.63788 | 0.000878 | 0.009095 | EC |
| AC079447.1 | -0.72866 | 0.000882 | 0.009095 | EC |
| MSC | 0.939255 | 0.000885 | 0.009109 | EC |
| APLF | -0.75576 | 0.000891 | 0.009157 | EC |
| PAMR1 | 0.668507 | 0.000892 | 0.00916 | EC |
| ANO2 | -0.69163 | 0.000903 | 0.009262 | EC |
| ARHGAP18 | -0.70804 | 0.000912 | 0.009332 | EC |

| | | | | |
|---|---|---|---|---|
| CD320 | 0.689153 | 0.000917 | 0.009363 | EC |
| HINT3 | -0.69814 | 0.000923 | 0.009396 | EC |
| PLCD3 | 0.764912 | 0.000923 | 0.009396 | EC |
| FBLN2 | 0.900361 | 0.000923 | 0.009396 | EC |
| UBFD1 | 0.838464 | 0.000934 | 0.009469 | EC |
| ATP6AP1 | 0.599072 | 0.000936 | 0.009487 | EC |
| C4A | 1.111584 | 0.00094 | 0.009511 | EC |
| NRROS | 0.801035 | 0.000948 | 0.009565 | EC |
| TMEM168 | -0.62505 | 0.000951 | 0.009591 | EC |
| NECTIN2 | 0.719522 | 0.000956 | 0.009628 | EC |
| AL928654.3 | 1.249669 | 0.000959 | 0.009645 | EC |
| SORBS3 | 0.59393 | 0.000977 | 0.009798 | EC |
| XIAP | -1.13292 | 0.000984 | 0.009856 | EC |
| SHTN1 | -0.96082 | 0.000989 | 0.009889 | EC |
| NIPAL2 | -0.75991 | 0.000997 | 0.009946 | EC |
| RAC3 | 0.728752 | 0.000999 | 0.009954 | EC |
| TRIM6-TRIM34 | -3.96725 | 0.001012 | 0.010039 | EC |
| ICAM5 | 0.944705 | 0.00102 | 0.010103 | EC |
| TRMT13 | -0.63277 | 0.001021 | 0.010103 | EC |
| TRPC6 | -0.80333 | 0.001022 | 0.010103 | EC |
| DUS3L | 0.658552 | 0.001023 | 0.010103 | EC |
| FANCM | -0.69293 | 0.001023 | 0.010103 | EC |
| POP7 | 0.684343 | 0.001027 | 0.010124 | EC |
| FOXO3B | 0.58989 | 0.001027 | 0.010124 | EC |
| ISG15 | 0.895295 | 0.00103 | 0.010142 | EC |
| NKIRAS1 | -0.6099 | 0.001041 | 0.010234 | EC |
| DEGS2 | 0.845801 | 0.001044 | 0.010249 | EC |
| CLEC11A | 1.152973 | 0.001049 | 0.010266 | EC |
| VAT1 | 0.593983 | 0.00105 | 0.010266 | EC |
| AGT | 0.874037 | 0.001049 | 0.010266 | EC |
| H3C4 | 0.828934 | 0.001052 | 0.010273 | EC |
| CEP126 | -0.8126 | 0.001062 | 0.010364 | EC |
| SP100 | -0.63292 | 0.001066 | 0.010372 | EC |
| STAT5A | 0.622477 | 0.001073 | 0.010398 | EC |
| CAPNS2 | -1.17774 | 0.001072 | 0.010398 | EC |
| CHST9 | -0.94882 | 0.001085 | 0.010456 | EC |
| SLCO2A1 | 1.184799 | 0.001085 | 0.010456 | EC |
| CCDC30 | -0.63563 | 0.00109 | 0.010485 | EC |
| SAMD12 | -1.03499 | 0.001092 | 0.010505 | EC |
| HLA-B | 0.879897 | 0.001097 | 0.010544 | EC |
| BCAT1 | -0.63243 | 0.001103 | 0.010587 | EC |
| ZNF678 | -1.00959 | 0.001114 | 0.010666 | EC |
| B3GALT4 | 0.606568 | 0.001114 | 0.010667 | EC |
| PMP22 | 0.660216 | 0.001116 | 0.010682 | EC |

| | | | | |
|---|---|---|---|---|
| FZD2 | 1.241183 | 0.00112 | 0.010704 | EC |
| YTHDF3 | -0.88367 | 0.001123 | 0.010715 | EC |
| NFKB2 | 0.978103 | 0.001125 | 0.010728 | EC |
| CD81 | 0.736747 | 0.001129 | 0.010747 | EC |
| GNG5 | 0.737742 | 0.001129 | 0.010747 | EC |
| CCDC85B | 0.639914 | 0.00113 | 0.010747 | EC |
| ZNF254 | -0.81381 | 0.00113 | 0.010747 | EC |
| BTF3L4 | -0.70081 | 0.00115 | 0.010873 | EC |
| TCP11X1 | -2.21012 | 0.00115 | 0.010873 | EC |
| SLC2A4RG | 0.636236 | 0.001154 | 0.01088 | EC |
| ABCA2 | 0.763167 | 0.001183 | 0.011064 | EC |
| ZNF711 | -0.62242 | 0.0012 | 0.011177 | EC |
| RNASEK | 0.810009 | 0.001204 | 0.0112 | EC |
| F12 | 1.10813 | 0.00121 | 0.011238 | EC |
| TMEM41B | -0.67514 | 0.001209 | 0.011238 | EC |
| IFITM3 | 0.896994 | 0.001211 | 0.011243 | EC |
| C1QTNF5 | 1.082073 | 0.001213 | 0.011249 | EC |
| ANKRD18A | -0.98964 | 0.001217 | 0.011272 | EC |
| MDGA1 | 0.586214 | 0.001219 | 0.011276 | EC |
| CLP1 | 0.590315 | 0.001221 | 0.011292 | EC |
| SARDH | 0.69331 | 0.001224 | 0.011312 | EC |
| TRIQK | -0.60669 | 0.001225 | 0.011312 | EC |
| TUBA1C | 0.613964 | 0.001229 | 0.011334 | EC |
| JOSD2 | 0.632529 | 0.001236 | 0.011368 | EC |
| BORCS8-MEF2B | 1.116692 | 0.001238 | 0.011387 | EC |
| PIGB | -0.58388 | 0.001245 | 0.011426 | EC |
| SLC27A3 | 0.714186 | 0.001249 | 0.011457 | EC |
| CCDC126 | -0.70713 | 0.001251 | 0.011465 | EC |
| NR1D2 | -0.6073 | 0.001254 | 0.011469 | EC |
| FRRS1L | -0.64807 | 0.001257 | 0.011488 | EC |
| SPDL1 | -0.77464 | 0.001263 | 0.011528 | EC |
| CDC7 | -0.61078 | 0.001263 | 0.011528 | EC |
| RSPO1 | -3.61973 | 0.001273 | 0.011584 | EC |
| PATE2 | -0.94973 | 0.001273 | 0.011584 | EC |
| MMP9 | 3.25121 | 0.001275 | 0.011592 | EC |
| PHLDA3 | 0.627934 | 0.001287 | 0.011636 | EC |
| RPL8 | 0.611992 | 0.001292 | 0.011661 | EC |
| MRGPRF | 1.118242 | 0.001297 | 0.011696 | EC |
| MEST | -0.61814 | 0.001306 | 0.011761 | EC |
| ATP2C2 | -0.68602 | 0.001308 | 0.011772 | EC |
| SMIM1 | 1.270855 | 0.001313 | 0.011805 | EC |
| CHST5 | 1.157436 | 0.001313 | 0.011805 | EC |
| VWC2L | -0.87178 | 0.001318 | 0.011835 | EC |
| AC007192.1 | 3.234309 | 0.00132 | 0.01184 | EC |

| | | | | |
|---|---|---|---|---|
| C1GALT1 | -0.64185 | 0.001326 | 0.01187 | EC |
| COX6A1 | 0.59745 | 0.001333 | 0.01192 | EC |
| LHPP | 0.622969 | 0.001343 | 0.011968 | EC |
| AC022400.5 | 0.745801 | 0.00135 | 0.011987 | EC |
| MSANTD3-TMEFF1 | -0.97254 | 0.001353 | 0.012003 | EC |
| NDUFA7 | 0.824204 | 0.001357 | 0.012021 | EC |
| FLNA | 0.742184 | 0.001366 | 0.012089 | EC |
| COL4A4 | -0.78244 | 0.001367 | 0.012095 | EC |
| RWDD1 | -0.72441 | 0.001374 | 0.012134 | EC |
| PGAM2 | 1.061756 | 0.001373 | 0.012134 | EC |
| EMC10 | 0.583181 | 0.001375 | 0.012136 | EC |
| CBS | 0.658075 | 0.001389 | 0.012228 | EC |
| NAGLU | 0.701423 | 0.001411 | 0.012352 | EC |
| CYCS | -0.61884 | 0.00141 | 0.012352 | EC |
| SAXO2 | -0.73724 | 0.001414 | 0.012373 | EC |
| SEPTIN7 | -0.71906 | 0.001418 | 0.012391 | EC |
| QPRT | 0.593978 | 0.001442 | 0.012538 | EC |
| RAB3IP | -0.74502 | 0.00144 | 0.012538 | EC |
| ZNF385D | -0.74406 | 0.001442 | 0.012538 | EC |
| LSM2 | 0.612607 | 0.001442 | 0.012538 | EC |
| HLA-A | 0.807944 | 0.001442 | 0.012538 | EC |
| TMEM265 | -1.30776 | 0.001444 | 0.012544 | EC |
| ZNF527 | -0.74945 | 0.001449 | 0.01258 | EC |
| ELMOD2 | -0.65305 | 0.001452 | 0.01259 | EC |
| VN1R1 | -0.72025 | 0.001465 | 0.012677 | EC |
| ITPRID1 | -1.38551 | 0.001478 | 0.012769 | EC |
| TAMALIN | 0.842094 | 0.001481 | 0.012774 | EC |
| C1orf194 | 1.45833 | 0.00148 | 0.012774 | EC |
| CYP4F3 | 1.327023 | 0.001482 | 0.012775 | EC |
| FAM102A | 0.694003 | 0.001484 | 0.012783 | EC |
| FAM186B | 1.926428 | 0.001489 | 0.012809 | EC |
| TMEM170B | -0.67527 | 0.001495 | 0.012829 | EC |
| PGLS | 0.624238 | 0.001511 | 0.012921 | EC |
| PCDHGB5 | -0.74928 | 0.001513 | 0.012927 | EC |
| PTGDS | 0.645796 | 0.001521 | 0.012973 | EC |
| IMP3 | 0.657618 | 0.001537 | 0.013082 | EC |
| AQP3 | 1.500433 | 0.001547 | 0.013141 | EC |
| D2HGDH | 0.601172 | 0.001551 | 0.013162 | EC |
| PPP1R1A | 0.654029 | 0.001561 | 0.013223 | EC |
| CHCHD6 | 0.59098 | 0.001566 | 0.013254 | EC |
| TMEM259 | 0.584021 | 0.001567 | 0.013254 | EC |
| LYRM7 | -0.67209 | 0.001572 | 0.013287 | EC |
| PIM3 | 0.708953 | 0.001573 | 0.013287 | EC |
| TRADD | 0.638365 | 0.001576 | 0.013299 | EC |

| | | | | |
|---|---|---|---|---|
| RTL8C | 0.595127 | 0.001578 | 0.013299 | EC |
| GABRB2 | -0.80262 | 0.001579 | 0.013299 | EC |
| NRAP | 1.521792 | 0.001576 | 0.013299 | EC |
| FBXO2 | 0.674768 | 0.001583 | 0.013312 | EC |
| LRRC37A | -0.80298 | 0.001598 | 0.013393 | EC |
| DCX | -0.65222 | 0.0016 | 0.013397 | EC |
| TCF7 | 0.808071 | 0.001656 | 0.01371 | EC |
| CCNC | -0.62345 | 0.001653 | 0.01371 | EC |
| WTIP | 0.631602 | 0.001654 | 0.01371 | EC |
| KCTD11 | 0.656551 | 0.001657 | 0.01371 | EC |
| LIPE | 0.908685 | 0.00166 | 0.013725 | EC |
| TMEM271 | -0.84263 | 0.001664 | 0.013746 | EC |
| ADAMTS9 | -1.07995 | 0.001688 | 0.013932 | EC |
| SESN2 | 0.587721 | 0.001694 | 0.013954 | EC |
| MGAT1 | 0.724213 | 0.001705 | 0.014011 | EC |
| MFSD5 | 0.586436 | 0.001704 | 0.014011 | EC |
| S100A1 | 0.654321 | 0.001713 | 0.014048 | EC |
| AP1AR | -0.58411 | 0.001729 | 0.014159 | EC |
| UBE2S | 0.620694 | 0.001743 | 0.014245 | EC |
| SYCE1L | 1.472628 | 0.001744 | 0.014247 | EC |
| IGF1 | -0.94641 | 0.00176 | 0.014344 | EC |
| SLC44A2 | 0.588648 | 0.001759 | 0.014344 | EC |
| GXYLT1 | -0.64718 | 0.001781 | 0.014483 | EC |
| AJM1 | 0.692351 | 0.001784 | 0.014489 | EC |
| SCARF2 | 0.629484 | 0.001808 | 0.014628 | EC |
| MC1R | 0.757442 | 0.001824 | 0.014692 | EC |
| CBR1 | 0.6964 | 0.001843 | 0.014805 | EC |
| CD52 | 1.753145 | 0.001865 | 0.014923 | EC |
| DAZAP2 | 0.755096 | 0.001881 | 0.015016 | EC |
| DNAJB5 | 0.772524 | 0.001888 | 0.015054 | EC |
| PLIN3 | 0.586474 | 0.001896 | 0.015091 | EC |
| AC011005.1 | 0.923876 | 0.001907 | 0.015174 | EC |
| RBM48 | -0.66433 | 0.00192 | 0.015234 | EC |
| DYNC1LI2 | -0.82708 | 0.001919 | 0.015234 | EC |
| IGFBP6 | 0.703374 | 0.00192 | 0.015234 | EC |
| FBLN1 | 0.850456 | 0.001924 | 0.015244 | EC |
| HSPB8 | 0.633241 | 0.001934 | 0.015307 | EC |
| EPHB2 | 0.632902 | 0.001952 | 0.015382 | EC |
| GDPD2 | 0.774796 | 0.001954 | 0.015383 | EC |
| HLA-DRB5 | 2.353341 | 0.001954 | 0.015383 | EC |
| CCDC171 | -0.62462 | 0.001962 | 0.015427 | EC |
| SLC43A2 | 0.70352 | 0.001976 | 0.015495 | EC |
| MMP2 | 0.986076 | 0.00198 | 0.015509 | EC |
| RPS6KA6 | -0.67105 | 0.00199 | 0.015535 | EC |

| | | | | |
|---|---|---|---|---|
| TMEM88 | 1.46491 | 0.001992 | 0.015535 | EC |
| KLRC4-KLRK1 | -1.49068 | 0.001991 | 0.015535 | EC |
| ZNF813 | -0.69052 | 0.001995 | 0.015548 | EC |
| ADAM32 | -0.61208 | 0.001997 | 0.015557 | EC |
| IGIP | -0.68954 | 0.002006 | 0.015621 | EC |
| NDUFA5 | -0.76008 | 0.002009 | 0.015632 | EC |
| RPL12 | 0.59177 | 0.00201 | 0.015635 | EC |
| PRKAR1A | -0.59951 | 0.002012 | 0.015638 | EC |
| HCST | 1.309453 | 0.002021 | 0.015662 | EC |
| NEK7 | -0.80802 | 0.002021 | 0.015662 | EC |
| RAB8B | -0.83909 | 0.00202 | 0.015662 | EC |
| ELOVL4 | -0.73251 | 0.002045 | 0.015774 | EC |
| KIRREL3 | 0.753233 | 0.002046 | 0.015774 | EC |
| ADAT3 | 1.16624 | 0.002041 | 0.015774 | EC |
| HCN4 | 0.805572 | 0.002057 | 0.015826 | EC |
| CRIP1 | 1.077925 | 0.002065 | 0.015876 | EC |
| VDR | 1.283961 | 0.002093 | 0.016028 | EC |
| ACTR6 | -0.59173 | 0.002106 | 0.016101 | EC |
| GPR22 | -0.92318 | 0.002107 | 0.016102 | EC |
| TPBGL | 0.96294 | 0.00212 | 0.01618 | EC |
| CDK2AP2 | 0.766168 | 0.002128 | 0.016208 | EC |
| DHRS4L2 | 0.749627 | 0.00214 | 0.016268 | EC |
| HFM1 | -0.68142 | 0.002153 | 0.016333 | EC |
| UCHL3 | -0.66088 | 0.002167 | 0.016415 | EC |
| ELOVL1 | 0.659447 | 0.002177 | 0.016465 | EC |
| MAP2K6 | -0.61182 | 0.002176 | 0.016465 | EC |
| ZNF286A | -0.65677 | 0.002185 | 0.016508 | EC |
| DEPDC4 | -0.87712 | 0.0022 | 0.016594 | EC |
| GABRA4 | -0.75687 | 0.002212 | 0.016653 | EC |
| NEK5 | -0.70136 | 0.002211 | 0.016653 | EC |
| YWHAB | -0.81996 | 0.002226 | 0.016712 | EC |
| MARVELD1 | 0.58215 | 0.002232 | 0.016728 | EC |
| RPL29 | 0.681657 | 0.00223 | 0.016728 | EC |
| CLDND2 | 1.547024 | 0.00224 | 0.016775 | EC |
| ERBB2 | 0.646647 | 0.002251 | 0.016819 | EC |
| ATG4C | -0.76958 | 0.002253 | 0.016819 | EC |
| LAMB2 | 0.745435 | 0.002289 | 0.017002 | EC |
| SUMO4 | -0.65378 | 0.002286 | 0.017002 | EC |
| CD72 | 0.806211 | 0.002321 | 0.017164 | EC |
| TAS2R8 | -1.40139 | 0.002324 | 0.017172 | EC |
| H2BC21 | 0.651718 | 0.002344 | 0.017262 | EC |
| CDC20 | 2.073531 | 0.00235 | 0.017284 | EC |
| CCDC167 | 0.774966 | 0.002352 | 0.01729 | EC |
| RAB20 | 0.789441 | 0.002355 | 0.01729 | EC |

| | | | | |
|---|---|---|---|---|
| EHD2 | 0.706174 | 0.002369 | 0.017344 | EC |
| DNAJC28 | -0.7033 | 0.002384 | 0.017408 | EC |
| GDAP1L1 | 0.629024 | 0.002399 | 0.017466 | EC |
| CAMK2N2 | 0.6925 | 0.0024 | 0.017469 | EC |
| GGT7 | 0.653125 | 0.002405 | 0.017492 | EC |
| C2orf88 | -1.03175 | 0.00241 | 0.017514 | EC |
| RRAS | 0.723302 | 0.002447 | 0.017679 | EC |
| ZNF804A | -0.73141 | 0.002456 | 0.01771 | EC |
| CELSR1 | 1.673806 | 0.002461 | 0.017737 | EC |
| CDKN2A | 0.914195 | 0.00247 | 0.017786 | EC |
| C1R | 0.745322 | 0.002483 | 0.017844 | EC |
| ISLR2 | 1.132586 | 0.002486 | 0.01786 | EC |
| RFK | -0.63519 | 0.002493 | 0.01789 | EC |
| BBC3 | 0.580228 | 0.002507 | 0.01796 | EC |
| RPS5 | 0.639317 | 0.002509 | 0.017963 | EC |
| BDP1 | -0.64935 | 0.002513 | 0.017971 | EC |
| ACOT1 | 0.937762 | 0.002514 | 0.017971 | EC |
| TNC | 1.345063 | 0.002518 | 0.017985 | EC |
| PHLDA2 | 2.392129 | 0.002518 | 0.017985 | EC |
| MFAP4 | 1.237884 | 0.002523 | 0.017994 | EC |
| MT1E | 0.797549 | 0.002522 | 0.017994 | EC |
| CNGA3 | 1.263894 | 0.002543 | 0.01808 | EC |
| H2AC21 | 0.658078 | 0.002551 | 0.018122 | EC |
| RPL13A | 0.622219 | 0.002564 | 0.018194 | EC |
| TWF1 | -0.63292 | 0.002573 | 0.01821 | EC |
| GAS2L1 | 0.641452 | 0.002572 | 0.01821 | EC |
| FGF12 | -0.63814 | 0.002587 | 0.018277 | EC |
| HVCN1 | 0.839009 | 0.002585 | 0.018277 | EC |
| ANKRD49 | -0.59418 | 0.002588 | 0.018277 | EC |
| FAM241A | -0.67904 | 0.002587 | 0.018277 | EC |
| VIP | -0.92572 | 0.002607 | 0.018337 | EC |
| CD74 | 0.947248 | 0.002614 | 0.018382 | EC |
| KIF5B | -0.74437 | 0.002636 | 0.018496 | EC |
| ARPP19 | -0.63921 | 0.002641 | 0.018514 | EC |
| CLCF1 | 1.979386 | 0.002652 | 0.018573 | EC |
| IL33 | -0.74673 | 0.002665 | 0.018662 | EC |
| TCTE3 | -0.77413 | 0.002672 | 0.018692 | EC |
| GLIS1 | 0.703513 | 0.002689 | 0.018778 | EC |
| FASTKD3 | -0.61836 | 0.002698 | 0.018819 | EC |
| FNDC10 | 0.698395 | 0.002704 | 0.018839 | EC |
| CMPK1 | -0.69019 | 0.002713 | 0.018858 | EC |
| CHI3L2 | 2.024149 | 0.002753 | 0.019065 | EC |
| RGR | 1.144552 | 0.002792 | 0.019282 | EC |
| H3C13 | 1.040601 | 0.002802 | 0.019339 | EC |

| | | | | |
|---|---|---|---|---|
| SMOX | 0.641182 | 0.002804 | 0.01934 | EC |
| PAIP2B | -1.16874 | 0.002807 | 0.019348 | EC |
| NUAK2 | 0.709196 | 0.002832 | 0.019455 | EC |
| EGR4 | 1.603664 | 0.002841 | 0.019488 | EC |
| UBA52 | 0.633848 | 0.002843 | 0.019488 | EC |
| EIF4E | -0.87425 | 0.002903 | 0.019783 | EC |
| ZNF808 | -0.75866 | 0.002909 | 0.019797 | EC |
| SETD6 | 0.665536 | 0.002914 | 0.019813 | EC |
| TPP1 | 0.603628 | 0.002915 | 0.019813 | EC |
| S100A4 | 1.489998 | 0.00295 | 0.020013 | EC |
| FOXS1 | 1.34497 | 0.002956 | 0.020036 | EC |
| SCN7A | -0.68737 | 0.002976 | 0.020134 | EC |
| SIGLEC1 | 1.11748 | 0.002996 | 0.020214 | EC |
| RPP40 | -0.74719 | 0.002991 | 0.020214 | EC |
| CDC42EP2 | 0.684215 | 0.002996 | 0.020214 | EC |
| AC069368.1 | 1.032845 | 0.003021 | 0.020327 | EC |
| DYNLT3 | -0.65021 | 0.003033 | 0.020371 | EC |
| GTDC1 | -0.59486 | 0.003044 | 0.020392 | EC |
| NFATC1 | 0.630727 | 0.00305 | 0.020417 | EC |
| MT-ATP6 | 1.365647 | 0.003087 | 0.020543 | EC |
| C6orf163 | -0.75096 | 0.003083 | 0.020543 | EC |
| COL6A2 | 0.883777 | 0.003097 | 0.020596 | EC |
| SLC15A3 | 0.654613 | 0.00311 | 0.020634 | EC |
| RPL39L | 0.834993 | 0.003125 | 0.020719 | EC |
| H3C1 | 0.822793 | 0.003143 | 0.02081 | EC |
| CD151 | 0.676099 | 0.003163 | 0.020884 | EC |
| BCL3 | 0.818804 | 0.003166 | 0.020887 | EC |
| CHADL | 0.94043 | 0.003204 | 0.021077 | EC |
| ZNF300 | -0.73059 | 0.003203 | 0.021077 | EC |
| KIAA0513 | 0.61737 | 0.00325 | 0.021317 | EC |
| SOX10 | 0.765699 | 0.003252 | 0.021322 | EC |
| H1-4 | 0.636173 | 0.003253 | 0.021322 | EC |
| PLXNA1 | 0.597592 | 0.003259 | 0.021322 | EC |
| SCAMP1 | -0.61698 | 0.003268 | 0.021358 | EC |
| H2AW | 0.72964 | 0.00328 | 0.021419 | EC |
| HLA-DQA1 | 2.0339 | 0.003302 | 0.021532 | EC |
| SLC8A2 | 0.637289 | 0.003308 | 0.021564 | EC |
| BSG | 0.603082 | 0.003315 | 0.021575 | EC |
| LDHD | 0.593544 | 0.003336 | 0.021678 | EC |
| SAMD9 | -0.65012 | 0.003364 | 0.021829 | EC |
| CCNJL | 0.609946 | 0.003369 | 0.021834 | EC |
| B3GALT2 | -0.7138 | 0.00337 | 0.021834 | EC |
| EIF1AX | -0.6539 | 0.003381 | 0.02188 | EC |
| LTBP2 | 1.031924 | 0.003385 | 0.021898 | EC |

| | | | | |
|---|---|---|---|---|
| LINGO1 | 0.726405 | 0.003411 | 0.022014 | EC |
| FPGT | -0.77236 | 0.003444 | 0.0222 | EC |
| LSM7 | 0.648027 | 0.003481 | 0.022393 | EC |
| IL17RE | 0.912887 | 0.003496 | 0.022476 | EC |
| LILRB2 | 0.889302 | 0.003504 | 0.022514 | EC |
| CEP295 | -0.70745 | 0.003509 | 0.022529 | EC |
| ANKRD36B | -0.62988 | 0.003518 | 0.022581 | EC |
| SLC26A4 | -0.76152 | 0.003526 | 0.022622 | EC |
| TGFB1 | 0.64426 | 0.003532 | 0.022648 | EC |
| EFHD1 | 0.638617 | 0.003539 | 0.022655 | EC |
| TMEM132E | 0.69187 | 0.003541 | 0.022655 | EC |
| CALCRL | -0.60924 | 0.003551 | 0.022703 | EC |
| TSPAN14 | 0.799933 | 0.003557 | 0.022726 | EC |
| LRRC34 | -0.58059 | 0.003569 | 0.022789 | EC |
| SGO2 | -0.61073 | 0.003577 | 0.022833 | EC |
| ARL6 | -0.62995 | 0.003603 | 0.022948 | EC |
| CLEC2D | -0.58049 | 0.00361 | 0.022979 | EC |
| BLOC1S3 | 0.898043 | 0.003616 | 0.023004 | EC |
| APOM | 0.996762 | 0.00363 | 0.023056 | EC |
| CD48 | 2.128383 | 0.003651 | 0.023153 | EC |
| STAP2 | 0.59276 | 0.003671 | 0.023251 | EC |
| FUOM | 0.762317 | 0.003676 | 0.023266 | EC |
| CYP4X1 | -0.63254 | 0.003682 | 0.023285 | EC |
| SEMA3B | 0.902594 | 0.003702 | 0.023329 | EC |
| BCL2L12 | 0.752145 | 0.003712 | 0.023347 | EC |
| AC040162.1 | 0.689664 | 0.003716 | 0.023357 | EC |
| TLR10 | -0.98489 | 0.003724 | 0.023386 | EC |
| CTXN1 | 0.705 | 0.003733 | 0.023423 | EC |
| PLXNB3 | 0.783576 | 0.003742 | 0.023455 | EC |
| MACROD2 | -0.5853 | 0.003744 | 0.023458 | EC |
| IMPG2 | -0.6291 | 0.003758 | 0.023504 | EC |
| ZNF264 | -1.31828 | 0.003754 | 0.023504 | EC |
| CNTN5 | -0.81556 | 0.003757 | 0.023504 | EC |
| B9D2 | 0.657306 | 0.003763 | 0.023516 | EC |
| CCR10 | 0.796396 | 0.003781 | 0.023598 | EC |
| OAF | 0.678269 | 0.003804 | 0.02367 | EC |
| MAP1LC3B2 | 0.707955 | 0.003807 | 0.023681 | EC |
| PRR7 | 0.625336 | 0.003819 | 0.023722 | EC |
| LRRCC1 | -0.61797 | 0.00383 | 0.023746 | EC |
| ATP1A2 | 0.833881 | 0.003854 | 0.023847 | EC |
| USP12 | -0.68278 | 0.003878 | 0.023948 | EC |
| NMB | 0.91738 | 0.003942 | 0.024231 | EC |
| ZBTB42 | 0.721679 | 0.003969 | 0.024348 | EC |
| EPOR | 0.634653 | 0.003992 | 0.024457 | EC |

| | | | | |
|---|---|---|---|---|
| ABCD2 | -0.62206 | 0.004028 | 0.024613 | EC |
| COL6A3 | 1.133933 | 0.00404 | 0.024633 | EC |
| COMTD1 | 0.711973 | 0.004038 | 0.024633 | EC |
| CAMKK1 | 0.703053 | 0.004062 | 0.024703 | EC |
| DNER | 0.60754 | 0.004058 | 0.024703 | EC |
| UFM1 | -0.58659 | 0.004065 | 0.024709 | EC |
| PLEKHH2 | -0.6073 | 0.004093 | 0.024834 | EC |
| L1TD1 | -1.59399 | 0.00411 | 0.024918 | EC |
| TTC9B | 0.600067 | 0.004138 | 0.025015 | EC |
| BRCA1 | -0.59053 | 0.004152 | 0.025082 | EC |
| STX11 | 0.994453 | 0.004168 | 0.025132 | EC |
| RBMS1 | -0.58128 | 0.00418 | 0.025168 | EC |
| ZNF860 | -1.14466 | 0.00418 | 0.025168 | EC |
| GLYCTK | 0.61888 | 0.004182 | 0.025169 | EC |
| ARC | 1.371055 | 0.004192 | 0.025212 | EC |
| PRRT1 | 0.626838 | 0.004204 | 0.025264 | EC |
| TRPC4 | -0.6062 | 0.004265 | 0.025491 | EC |
| CSRNP1 | 0.686825 | 0.004309 | 0.025651 | EC |
| GABRD | 0.797481 | 0.004326 | 0.025741 | EC |
| ACTA1 | 1.38577 | 0.004349 | 0.025861 | EC |
| SLC6A9 | 0.628346 | 0.00435 | 0.025861 | EC |
| SIX5 | 0.633858 | 0.004355 | 0.025865 | EC |
| ZNF714 | -0.72291 | 0.004365 | 0.025907 | EC |
| GPR3 | 1.146084 | 0.004372 | 0.025934 | EC |
| UNC5A | 0.669612 | 0.004386 | 0.025975 | EC |
| TGFBR3L | 0.921835 | 0.004387 | 0.025975 | EC |
| H4C6 | 1.010771 | 0.004412 | 0.026069 | EC |
| KCNC3 | 0.780258 | 0.004416 | 0.026082 | EC |
| VIT | 1.38389 | 0.004445 | 0.02623 | EC |
| G0S2 | 1.217392 | 0.004453 | 0.026232 | EC |
| YIPF6 | -1.21473 | 0.004452 | 0.026232 | EC |
| AC099489.1 | 0.738372 | 0.004451 | 0.026232 | EC |
| SNAPC2 | 0.644239 | 0.004476 | 0.026319 | EC |
| WNK4 | 1.402953 | 0.004491 | 0.0264 | EC |
| CCDC18 | -0.68117 | 0.0045 | 0.026433 | EC |
| SLC25A22 | 0.585782 | 0.00451 | 0.026463 | EC |
| EIF5A2 | -0.60111 | 0.004515 | 0.02648 | EC |
| LEPROTL1 | -0.60972 | 0.004534 | 0.026529 | EC |
| H2AC13 | 1.56782 | 0.004534 | 0.026529 | EC |
| STARD4 | -0.63295 | 0.004551 | 0.026604 | EC |
| IGSF9B | 1.166366 | 0.004583 | 0.026735 | EC |
| AEBP1 | 0.973676 | 0.004592 | 0.026772 | EC |
| UFSP1 | 0.911991 | 0.004598 | 0.026788 | EC |
| LRP5 | 0.720449 | 0.004602 | 0.026797 | EC |

| | | | | |
|---|---|---|---|---|
| GINS3 | 0.991395 | 0.004606 | 0.026802 | EC |
| ARHGAP29 | -0.65588 | 0.004621 | 0.026845 | EC |
| KISS1R | 1.721918 | 0.004636 | 0.026889 | EC |
| ZP3 | 0.703762 | 0.004635 | 0.026889 | EC |
| S100A10 | 0.876181 | 0.004637 | 0.026889 | EC |
| HLA-DQB1 | 1.784003 | 0.004663 | 0.026983 | EC |
| ZDHHC20 | -0.63667 | 0.004665 | 0.026983 | EC |
| EXOG | -0.62359 | 0.004675 | 0.027021 | EC |
| BAG2 | -0.6681 | 0.004685 | 0.027048 | EC |
| PRR36 | 0.710813 | 0.004771 | 0.027502 | EC |
| MSMO1 | -0.76362 | 0.004796 | 0.027622 | EC |
| LAGE3 | 0.669081 | 0.004826 | 0.027731 | EC |
| FA2H | 0.892201 | 0.004852 | 0.027821 | EC |
| C9orf16 | 0.59525 | 0.00486 | 0.027837 | EC |
| HAPLN1 | -1.04575 | 0.004863 | 0.027845 | EC |
| NAP1L1 | -0.62374 | 0.004869 | 0.027853 | EC |
| SLC22A4 | 0.693381 | 0.004876 | 0.027881 | EC |
| LOXL2 | 0.598638 | 0.004912 | 0.028058 | EC |
| EYA2 | 0.636394 | 0.004924 | 0.028106 | EC |
| WDR90 | 0.582368 | 0.004924 | 0.028106 | EC |
| GCC2 | -0.60893 | 0.004935 | 0.028124 | EC |
| CBWD6 | -0.62253 | 0.004938 | 0.028124 | EC |
| TNFSF9 | 1.0531 | 0.00496 | 0.028211 | EC |
| AATK | 0.695317 | 0.004972 | 0.02825 | EC |
| PPP1R35 | 0.607499 | 0.004983 | 0.028305 | EC |
| TET3 | 0.716394 | 0.005009 | 0.028421 | EC |
| SV2C | 1.446763 | 0.005024 | 0.028495 | EC |
| AC023055.1 | 1.955132 | 0.005043 | 0.028559 | EC |
| LYPD6B | -0.81747 | 0.005061 | 0.028625 | EC |
| HEPACAM | 0.599507 | 0.00511 | 0.028854 | EC |
| NPIPB4 | -0.78802 | 0.005108 | 0.028854 | EC |
| TTC23L | -0.6854 | 0.005144 | 0.028997 | EC |
| LACC1 | -0.95576 | 0.005179 | 0.029162 | EC |
| AP001458.2 | 0.961867 | 0.005257 | 0.029491 | EC |
| SNTA1 | 0.592673 | 0.005269 | 0.029525 | EC |
| SNX6 | -0.5897 | 0.005275 | 0.029541 | EC |
| LCK | 2.12336 | 0.005282 | 0.02957 | EC |
| BGLAP | 0.904499 | 0.005292 | 0.029613 | EC |
| SPI1 | 0.76379 | 0.005309 | 0.029664 | EC |
| RPL26L1 | 0.594986 | 0.005397 | 0.029981 | EC |
| BST2 | 0.71778 | 0.005459 | 0.03022 | EC |
| TMEM156 | -0.89377 | 0.005497 | 0.030348 | EC |
| PCOLCE | 0.683027 | 0.00551 | 0.030372 | EC |
| ZNF391 | -0.72153 | 0.005525 | 0.030403 | EC |

| | | | | |
|---|---|---|---|---|
| SZRD1 | 0.746556 | 0.005547 | 0.030473 | EC |
| TAS2R19 | -0.70661 | 0.005543 | 0.030473 | EC |
| COL4A3 | -0.69872 | 0.005604 | 0.03061 | EC |
| TAF13 | -0.63226 | 0.005637 | 0.030723 | EC |
| TCAP | 0.646135 | 0.005647 | 0.030752 | EC |
| ZNF214 | -0.58248 | 0.005663 | 0.030784 | EC |
| COL13A1 | 1.485216 | 0.005682 | 0.030844 | EC |
| ADGRE5 | 0.72456 | 0.005688 | 0.03086 | EC |
| METTL7B | 0.871011 | 0.005697 | 0.030871 | EC |
| UTS2R | 1.97157 | 0.005698 | 0.030871 | EC |
| FCER1G | 1.047877 | 0.005738 | 0.03103 | EC |
| ABCA12 | -1.36762 | 0.005765 | 0.03113 | EC |
| IQCJ | -0.8745 | 0.005872 | 0.031637 | EC |
| SCN5A | 1.045683 | 0.005902 | 0.031765 | EC |
| RAMP2 | 1.193086 | 0.005935 | 0.031915 | EC |
| HLA-E | 0.772245 | 0.005958 | 0.032013 | EC |
| EPHA8 | 0.681209 | 0.005982 | 0.032105 | EC |
| SERTM2 | -2.73015 | 0.006016 | 0.032229 | EC |
| SLC49A3 | 0.655446 | 0.006045 | 0.032336 | EC |
| SYNGR3 | 0.587211 | 0.006049 | 0.032344 | EC |
| HRC | 0.922957 | 0.006056 | 0.032362 | EC |
| MCMDC2 | -0.80766 | 0.006055 | 0.032362 | EC |
| RAB27B | -0.78737 | 0.00613 | 0.032691 | EC |
| ADM5 | 1.402511 | 0.006155 | 0.032762 | EC |
| TIGD4 | -0.62678 | 0.006179 | 0.032867 | EC |
| CXCL3 | 1.871568 | 0.006206 | 0.032993 | EC |
| KLRC4 | -1.33949 | 0.006309 | 0.033342 | EC |
| TMEM88B | 1.136529 | 0.006308 | 0.033342 | EC |
| TTN | -0.85114 | 0.006313 | 0.033347 | EC |
| PAX2 | 1.826163 | 0.006341 | 0.033409 | EC |
| TNFRSF18 | 1.301921 | 0.006372 | 0.033502 | EC |
| MCM2 | 0.638127 | 0.006379 | 0.033528 | EC |
| GPR37L1 | 0.685462 | 0.006419 | 0.033674 | EC |
| FAM222A | 0.692041 | 0.006452 | 0.033781 | EC |
| HES6 | 0.645946 | 0.006452 | 0.033781 | EC |
| METTL7A | -0.71395 | 0.006478 | 0.033898 | EC |
| PCDHGA4 | -0.69384 | 0.006525 | 0.034056 | EC |
| KRT86 | 1.580656 | 0.006559 | 0.034178 | EC |
| SNX10 | -0.71456 | 0.006584 | 0.034222 | EC |
| NPY | 1.198134 | 0.006608 | 0.034305 | EC |
| FAM181B | 0.598123 | 0.00662 | 0.034333 | EC |
| IQCF3 | -1.32827 | 0.006626 | 0.034352 | EC |
| ADAMTS2 | 0.871632 | 0.006658 | 0.034449 | EC |
| NKG7 | 1.841808 | 0.006651 | 0.034449 | EC |

| | | | | |
|---|---|---|---|---|
| ZNF233 | -0.62094 | 0.006689 | 0.03459 | EC |
| SNCG | 0.600916 | 0.006693 | 0.0346 | EC |
| CD3E | 2.124699 | 0.00674 | 0.034801 | EC |
| NINJ2 | 0.931328 | 0.006752 | 0.03483 | EC |
| FANCB | -0.96892 | 0.00675 | 0.03483 | EC |
| TNFRSF12A | 1.083949 | 0.006764 | 0.034875 | EC |
| C19orf71 | 0.713597 | 0.006773 | 0.034897 | EC |
| TSPO | 0.762111 | 0.006782 | 0.034917 | EC |
| PCDH11X | -0.61831 | 0.006795 | 0.034951 | EC |
| TFPI | -0.81134 | 0.006807 | 0.034967 | EC |
| KCNJ4 | 0.649608 | 0.006813 | 0.034989 | EC |
| USP18 | 0.630338 | 0.006819 | 0.035009 | EC |
| ARL8B | -0.74701 | 0.006827 | 0.035027 | EC |
| KLF2 | 1.196554 | 0.00685 | 0.035111 | EC |
| RNF152 | -0.72054 | 0.006952 | 0.035487 | EC |
| SLC7A10 | 0.681778 | 0.00698 | 0.035553 | EC |
| KIF14 | -1.30025 | 0.006982 | 0.035556 | EC |
| PLLP | 0.790446 | 0.007101 | 0.036026 | EC |
| HPN | 0.652443 | 0.007128 | 0.036094 | EC |
| H2AC12 | 1.26 | 0.007127 | 0.036094 | EC |
| SPR | 0.715722 | 0.007142 | 0.036154 | EC |
| KLHL5 | -0.5854 | 0.007154 | 0.036203 | EC |
| METTL25 | -0.59568 | 0.007192 | 0.036329 | EC |
| ANKRD20A1 | -0.85787 | 0.0072 | 0.036347 | EC |
| PLEKHF1 | 0.628029 | 0.007226 | 0.03642 | EC |
| TMEM238 | 1.254389 | 0.007271 | 0.036571 | EC |
| DLK2 | 0.752872 | 0.007276 | 0.036577 | EC |
| PODNL1 | 0.843577 | 0.007281 | 0.036588 | EC |
| H1-2 | 0.621575 | 0.007295 | 0.036643 | EC |
| ZNF449 | -0.61267 | 0.007314 | 0.036694 | EC |
| CGB7 | -2.16169 | 0.007344 | 0.036748 | EC |
| CCDC54 | -1.20929 | 0.007375 | 0.036864 | EC |
| LRGUK | -0.61936 | 0.007477 | 0.037233 | EC |
| SLC22A18 | 0.724558 | 0.007508 | 0.03735 | EC |
| PATL2 | 1.005368 | 0.007523 | 0.037402 | EC |
| MT1F | 0.911793 | 0.007545 | 0.037475 | EC |
| CCKBR | 0.724655 | 0.007578 | 0.037618 | EC |
| SYT5 | 0.714278 | 0.007595 | 0.037688 | EC |
| PCMTD1 | -0.66843 | 0.007613 | 0.037731 | EC |
| CDH12 | -0.74849 | 0.007644 | 0.037859 | EC |
| TEN1 | -0.99212 | 0.007672 | 0.037919 | EC |
| MMP25 | 1.224933 | 0.007698 | 0.038003 | EC |
| CTNNA3 | -1.0882 | 0.00771 | 0.038041 | EC |
| TMEM59L | 0.609742 | 0.007742 | 0.038152 | EC |

| | | | | |
|---|---|---|---|---|
| CCDC68 | -0.73624 | 0.007767 | 0.038216 | EC |
| AGTPBP1 | -0.69479 | 0.007776 | 0.038248 | EC |
| CCDC181 | -0.66679 | 0.007856 | 0.038528 | EC |
| TMEM125 | 1.090748 | 0.007861 | 0.038539 | EC |
| TCN2 | 0.631737 | 0.007877 | 0.038597 | EC |
| MAG | 0.907676 | 0.007894 | 0.038647 | EC |
| MNS1 | -0.74928 | 0.007918 | 0.03872 | EC |
| VWCE | 0.614989 | 0.007919 | 0.03872 | EC |
| PLCD1 | 0.602305 | 0.007935 | 0.038742 | EC |
| S100A11 | 0.879269 | 0.007948 | 0.038793 | EC |
| C2CD4B | 1.887692 | 0.007966 | 0.038869 | EC |
| GPR68 | 0.996472 | 0.008011 | 0.038977 | EC |
| BCAN | 0.580059 | 0.008033 | 0.039057 | EC |
| TMEM169 | -0.67281 | 0.008038 | 0.039068 | EC |
| SCRT2 | 1.072331 | 0.008045 | 0.039093 | EC |
| PAQR4 | 0.602063 | 0.008049 | 0.039099 | EC |
| MSX1 | 0.818834 | 0.008065 | 0.039167 | EC |
| SELPLG | 0.733196 | 0.008098 | 0.039266 | EC |
| H2BC11 | 0.825733 | 0.008122 | 0.039347 | EC |
| CLEC3B | 0.904983 | 0.008218 | 0.039693 | EC |
| KLHL31 | -0.86042 | 0.008275 | 0.039896 | EC |
| CYP21A2 | 1.058216 | 0.008277 | 0.039896 | EC |
| F10 | 1.331422 | 0.008287 | 0.039934 | EC |
| RASSF7 | 0.726726 | 0.008296 | 0.039968 | EC |
| TTPA | -0.82143 | 0.008307 | 0.040007 | EC |
| CCDC175 | -0.77021 | 0.008324 | 0.040046 | EC |
| NRGN | 0.744944 | 0.008334 | 0.040046 | EC |
| CRCP | -0.68634 | 0.008334 | 0.040046 | EC |
| LOXL1 | 0.711994 | 0.008359 | 0.040129 | EC |
| CACNA1G | 0.8041 | 0.008362 | 0.04013 | EC |
| MMEL1 | 0.884224 | 0.008367 | 0.04013 | EC |
| H2AC18 | 0.689495 | 0.00837 | 0.040134 | EC |
| AC093155.3 | -1.23429 | 0.008385 | 0.040168 | EC |
| ACAN | 0.721954 | 0.008408 | 0.040254 | EC |
| PNMT | 0.707404 | 0.008483 | 0.040511 | EC |
| NIPAL4 | 0.927357 | 0.008574 | 0.040827 | EC |
| DBNDD2 | 0.719665 | 0.008575 | 0.040827 | EC |
| STAT4 | -0.72878 | 0.008621 | 0.040962 | EC |
| COPZ2 | 0.600406 | 0.008746 | 0.041402 | EC |
| OASL | 1.325597 | 0.008754 | 0.041428 | EC |
| GCK | 0.605309 | 0.008794 | 0.041551 | EC |
| HPGD | -1.0228 | 0.008795 | 0.041551 | EC |
| CHRM4 | 0.730305 | 0.008845 | 0.041725 | EC |
| ZDHHC12 | 0.726021 | 0.00891 | 0.041957 | EC |

| | | | | |
|---|---|---|---|---|
| SYT16 | -0.61801 | 0.008941 | 0.04201 | EC |
| PEBP4 | 0.890062 | 0.008979 | 0.042151 | EC |
| UBE2W | -0.67283 | 0.009025 | 0.04232 | EC |
| PRSS27 | 0.652756 | 0.009061 | 0.042464 | EC |
| DUSP3 | -0.82588 | 0.009071 | 0.042484 | EC |
| AC073896.1 | 0.637934 | 0.009124 | 0.042678 | EC |
| ERFL | 1.094323 | 0.009131 | 0.042678 | EC |
| GOLGA8J | 1.209164 | 0.009256 | 0.043176 | EC |
| HBQ1 | 1.326808 | 0.009262 | 0.043194 | EC |
| AC022384.1 | 1.583823 | 0.009287 | 0.043271 | EC |
| TMEM176B | 0.609858 | 0.009316 | 0.043336 | EC |
| RNASE1 | 0.79545 | 0.009326 | 0.043356 | EC |
| RORA | -0.60572 | 0.00935 | 0.043407 | EC |
| FAM187A | 2.079292 | 0.009373 | 0.043487 | EC |
| CSRNP3 | -0.66757 | 0.009522 | 0.044015 | EC |
| KCNG2 | 1.034874 | 0.009531 | 0.044035 | EC |
| CHODL | 1.038037 | 0.009634 | 0.044366 | EC |
| SLC38A11 | -0.74155 | 0.009678 | 0.0445 | EC |
| PRRT4 | 0.6843 | 0.009712 | 0.044616 | EC |
| ANKRD20A3P | -0.62005 | 0.009722 | 0.044651 | EC |
| EGR3 | 0.905689 | 0.009743 | 0.044721 | EC |
| SDSL | 0.82015 | 0.009769 | 0.044802 | EC |
| PET117 | -0.62035 | 0.009817 | 0.044986 | EC |
| GNG8 | 1.149288 | 0.009833 | 0.04502 | EC |
| IKBKE | 0.622868 | 0.009963 | 0.045374 | EC |
| TONSL | 0.598187 | 0.010041 | 0.045627 | EC |
| RNF133 | -0.7965 | 0.010043 | 0.045627 | EC |
| NLRP12 | 1.151714 | 0.010053 | 0.045643 | EC |
| SERPINH1 | 0.897371 | 0.010096 | 0.045753 | EC |
| DEPDC1 | -1.03608 | 0.010109 | 0.045776 | EC |
| TLR2 | -0.77762 | 0.010146 | 0.045875 | EC |
| LAMC3 | 0.657549 | 0.010192 | 0.046042 | EC |
| LTC4S | 1.192655 | 0.01022 | 0.046119 | EC |
| S1PR4 | 1.794466 | 0.010255 | 0.046203 | EC |
| VSTM2L | 0.712445 | 0.010258 | 0.046203 | EC |
| AHNAK2 | 0.618659 | 0.010342 | 0.046437 | EC |
| FGF20 | -1.2367 | 0.010367 | 0.046491 | EC |
| GPR6 | 1.310446 | 0.010366 | 0.046491 | EC |
| EFNA3 | 0.595388 | 0.01041 | 0.046671 | EC |
| H2BC7 | 0.669132 | 0.010448 | 0.046752 | EC |
| WFS1 | 0.695443 | 0.01048 | 0.046808 | EC |
| PI16 | 0.849099 | 0.010492 | 0.046832 | EC |
| ASAH2B | -0.63095 | 0.010517 | 0.046928 | EC |
| SLC25A35 | 0.590799 | 0.010554 | 0.047059 | EC |

| | | | | |
|---|---|---|---|---|
| TMED6 | -0.77388 | 0.010584 | 0.047164 | EC |
| PSORS1C1 | 1.003483 | 0.010603 | 0.04721 | EC |
| GPR37 | 0.784776 | 0.010628 | 0.047272 | EC |
| DOT1L | 0.580749 | 0.010765 | 0.047829 | EC |
| RAB17 | 1.187319 | 0.010876 | 0.048178 | EC |
| C4orf3 | -0.64727 | 0.011059 | 0.04879 | EC |
| LIPT1 | -0.75753 | 0.011151 | 0.049104 | EC |
| HCN1 | -0.58741 | 0.011149 | 0.049104 | EC |
| RIMBP3 | 1.006341 | 0.011163 | 0.04914 | EC |
| NTN3 | 0.746046 | 0.011205 | 0.049194 | EC |
| BMP3 | -1.09604 | 0.011261 | 0.049332 | EC |
| TAS2R7 | -1.06148 | 0.011276 | 0.049364 | EC |
| JRK | -0.93312 | 0.011286 | 0.049378 | EC |
| HRCT1 | 0.757625 | 0.01144 | 0.04989 | EC |
