# Supplementary material for: Integrative Transcriptomic Analyses of Hippocampal–Entorhinal System Subfields Identify Key Regulators in Alzheimer's Disease
Source: Adv Sci (Weinh). 2023 May 26;10(22):2300876. doi: 10.1002/advs.202300876 (PMC10401097; doi:10.1002/advs.202300876)
Supplement: Supplementary file 4 — Supplemental Table 3 [file ADVS-10-2300876-s007.pdf]

ADVANCED SCIENCE
Open Access

Supporting Information

for *Adv. Sci.*, DOI 10.1002/advs.202300876

Integrative Transcriptomic Analyses of Hippocampal–Entorhinal System Subfields Identify Key Regulators in Alzheimer's Disease

Dan Luo, Jingying Li, Hanyou Liu, Jiayu Wang, Yu Xia, Wenying Qiu, Naili Wang, Xue Wang, Xia Wang, Chao Ma* and Wei Ge**

Table S3. Summary of significant TWAS hits for Hippocampal analyses.

gene	geneid	chr	start_pos	end_pos	padj
NDUFS2	ENSG00000158864.12	1	161197104	161214395	6.52E-06
CR2	ENSG00000117322.17	1	207454230	207489895	2.53E-07
CR1	ENSG00000203710.10	1	207496147	207640647	3.47E-06
HLA-DRB6	ENSG00000229391.7	6	32552713	32560022	8.97E-07
HLA-DRB1	ENSG00000196126.11	6	32578769	32589848	2.85E-09
HLA-DQA1	ENSG00000196735.11	6	32628179	32643652	7.82E-06
HLA-DQA2	ENSG00000237541.3	6	32741342	32747215	2.37E-09
BRD2	ENSG00000204256.12	6	32968660	32981505	1.72E-06
ZKSCAN1	ENSG00000106261.16	7	100015572	100041689	7.83E-09
AP4M1	ENSG00000221838.9	7	100101941	100110345	5.96E-07
CNPY4	ENSG00000166997.7	7	100119613	100125511	5.20E-07
C7orf43	ENSG00000146826.16	7	100154420	100158715	5.47E-08
AP000640.10	ENSG00000254477.1	11	59753015	59754975	1.15E-07
MRPL16	ENSG00000166902.4	11	59806135	59810872	1.32E-07
MS4A6A	ENSG00000110077.14	11	60172014	60184666	8.23E-06
MS4A1	ENSG00000156738.17	11	60455752	60470760	8.19E-09
RP11-320L11.2	ENSG00000254783.1	11	86283927	86284668	2.82E-06
GOLGA5	ENSG00000066455.12	14	92794231	92839963	1.74E-08
CHGA	ENSG00000100604.12	14	92923080	92935293	6.95E-07
SLC7A5P1	ENSG00000260727.1	16	29613104	29613640	4.28E-06
INO80E	ENSG00000169592.14	16	29995294	30005508	6.75E-06

RP11-455F5.3	ENSG00000250616.2	16	30096430	30104116	8.01E-06
RP11-455F5.5	ENSG00000261416.1	16	30183726	30184563	1.27E-06
CD2BP2	ENSG00000169217.8	16	30350766	30355361	3.04E-06
RP11-146F11.1	ENSG00000261840.2	16	30697707	30699058	3.91E-07
ZNF629	ENSG00000102870.5	16	30778449	30787202	1.59E-06
CTF1	ENSG00000150281.6	16	30896607	30903560	5.77E-06
FBXL19	ENSG00000099364.16	16	30924097	30948783	1.59E-07
KAT8	ENSG00000103510.19	16	31115754	31131393	1.32E-08
RP11-196G11.4	ENSG00000262766.1	16	31118078	31118747	6.60E-06
PRSS36	ENSG00000178226.10	16	31138925	31150094	1.85E-06
SCIMP	ENSG00000161929.14	17	5208961	5234860	3.94E-06
ZNF180	ENSG00000167384.10	19	44474428	44500524	1.17E-08
CTB-171A8.1	ENSG00000266903.1	19	44632199	44718759	2.43E-74
TOMM40	ENSG00000130204.12	19	44890569	44903689	4.18E-21
APOE	ENSG00000130203.9	19	44905754	44909393	9.40E-10
CTB-129P6.11	ENSG00000267114.1	19	44950044	44954007	1.02E-11
CLPTM1	ENSG00000104853.15	19	44954591	44993341	1.99E-75
ZNF296	ENSG00000170684.8	19	45071500	45076509	1.62E-18
GEMIN7	ENSG00000142252.10	19	45079195	45091524	2.45E-06
MARK4	ENSG00000007047.14	19	45251258	45305283	1.19E-19
PPP1R37	ENSG00000104866.10	19	45091792	45147285	1.18E-31
EXOC3L2	ENSG00000283632.2	19	45212621	45245431	6.75E-23
CKM	ENSG00000104879.4	19	45306414	45322977	7.39E-0

					6
CD3EAP	ENSG00000117877.10	19	45406209	45410766	4.97E-19
OPA3	ENSG00000125741.4	19	45527427	45602212	1.98E-12
AC074212.6	ENSG00000267395.5	19	45767796	45772504	9.20E-06
