# Supplementary material for: Integrative Transcriptomic Analyses of Hippocampal–Entorhinal System Subfields Identify Key Regulators in Alzheimer's Disease
Source: Adv Sci (Weinh). 2023 May 26;10(22):2300876. doi: 10.1002/advs.202300876 (PMC10401097; doi:10.1002/advs.202300876)
Supplement: Supplementary file 5 — Supplemental Table 4 [file ADVS-10-2300876-s006.pdf]

ADVANCED SCIENCE
Open Access

Supporting Information

for *Adv. Sci.*, DOI 10.1002/advs.202300876

Integrative Transcriptomic Analyses of Hippocampal–Entorhinal System Subfields Identify Key Regulators in Alzheimer's Disease

Dan Luo, Jingying Li, Hanyou Liu, Jiayu Wang, Yu Xia, Wenying Qiu, Naili Wang, Xue Wang, Xia Wang, Chao Ma* and Wei Ge**

Table S4. Summary of significant TWAS hits for cortical analyses

gene	geneid	chr	start_pos	end_pos	padj
NIT1	ENSG00000158793.13	1	161118112	161125445	6.79E-07
CR1	ENSG00000203710.10	1	207496147	207640647	3.61E-06
HLA-DRB1	ENSG00000196126.11	6	32578769	32589848	1.25E-07
HLA-DQA2	ENSG00000237541.3	6	32741342	32747215	7.84E-08
AZGP1	ENSG00000160862.12	7	99966720	99976157	6.48E-09
ZKSCAN1	ENSG00000106261.16	7	100015572	100041689	3.19E-07
PCOLCE	ENSG00000106333.12	7	100602177	100608175	2.12E-07
TFR2	ENSG00000106327.12	7	100620416	100642721	4.86E-09
GNB2	ENSG00000172354.9	7	100673531	100679174	1.44E-07
INO80E	ENSG00000169592.14	16	29995294	30005508	1.93E-06
ZNF48	ENSG00000180035.12	16	30378106	30400108	6.57E-06
BCL7C	ENSG00000099385.11	16	30833626	30894547	1.21E-06
ORAI3	ENSG00000175938.6	16	30949066	30956461	2.24E-06
ZNF646	ENSG00000167395.10	16	31074422	31084118	2.16E-07
KAT8	ENSG00000103510.19	16	31115754	31131393	2.33E-09
RP11-196G11.4	ENSG00000262766.1	16	31118078	31118747	6.79E-06
PRSS8	ENSG00000052344.15	16	31131605	31135762	7.31E-06
PRSS36	ENSG00000178226.10	16	31138925	31150094	7.28E-07
FUS	ENSG00000089280.18	16	31180110	31194871	2.76E-07
CHRNE	ENSG00000108556.7	17	4898062	4903074	1.44E-07
ACE	ENSG00000159640.15	17	63477061	63498380	1.10E-06
CTB-171A8.1	ENSG00000266903.1	19	44632199	44718759	7.49E-83
CEACAM19	ENSG00000186567.12	19	44666958	44684359	2.20E-31
CBLC	ENSG00000142273.10	19	44777869	44800634	8.36E-22
APOE	ENSG00000130203.9	19	44905754	44909393	3.11E-42
CTB-129P6.11	ENSG00000267114.1	19	44950044	44954007	1.33E-22
CLASRP	ENSG00000104859.14	19	45039040	45070956	2.15E-25
ZNF296	ENSG00000170684.8	19	45071500	45076509	1.05E-10
PPP1R37	ENSG00000104866.10	19	45091792	45147285	1.47E-17
TRAPPC6A	ENSG00000007255.10	19	45162928	45178237	4.84E-40
KLC3	ENSG00000104892.16	19	45333434	45349602	1.18E-09
CD3EAP	ENSG00000117877.10	19	45406209	45410766	1.36E-14
