# Supplementary material for: Integrative Transcriptomic Analyses of Hippocampal–Entorhinal System Subfields Identify Key Regulators in Alzheimer's Disease
Source: Adv Sci (Weinh). 2023 May 26;10(22):2300876. doi: 10.1002/advs.202300876 (PMC10401097; doi:10.1002/advs.202300876)
Supplement: Supplementary file 9 — Supplemental Table 8 [file ADVS-10-2300876-s008.pdf]

ADVANCED SCIENCE
Open Access

Supporting Information

for *Adv. Sci.*, DOI 10.1002/advs.202300876

Integrative Transcriptomic Analyses of Hippocampal–Entorhinal System Subfields Identify Key Regulators in Alzheimer's Disease

Dan Luo, Jingying Li, Hanyou Liu, Jiayu Wang, Yu Xia, Wenying Qiu, Naili Wang, Xue Wang, Xia Wang, Chao Ma* and Wei Ge**

Table S8. Rat primer sequences.

Gene	Forward	Reverse
Amigo2	GTTCGCCACAACAACATCAC	GTTTCTGCAAGTGGGAGAGC
Axl	GAAGCCACCTTGAACAGTC	CACCTTATGCCGATCTACCA
β-Actin	AAGTCCCTCACCCTCCCAAAAG	AAGCAATGCTGTCACCTTCCC
B3gnt5	TGCTCCTGGATGAAAGGTCC	ACATGCTTGATCCGTGTGGT
C3	ACAGGAGAAACTTAAGGTAAGGG	TAGTACCGCTTCTTGGCAG
Cd109	GTCGCTCACAGGTACCTCAA	CTGTGAAGTTGAGCGTTGGC
Cd44	CACTCAAGTGGGAATCAAGAC	GATAAGCCACTCTGGAATCTG
Clcf1	GGACCTACCTGAACTACCTG	CACTTCCAAGTTGACCGTG
Cp	GATGTTTCCCCAAACGCCTG	GTAGCTCTGAGACGATGCTTGA
Emp1	ACCATTGCCAACGTCTGGAT	TGGAACACGAAGACCACGAG
Fbln5	ATGGTTCTTTCATCTGCCG	TGCACTCATCCATATCACTG
Fkbp5	GAACCCAATGCTGAGCTTATG	ATGTACTTGCCTCCCTTGAAG
Gas6	GATATCAAGAGTGCATGAGGA	GTGCCCTTCTTATCACAGG
Gbp2	GGAGATTGAAGTGGAACGG	TCAAACTTCTCCTGCTTCTC
Gfap	AAATCTGTGTCAGAAGGCCA	CTCCTTAATGACCTCGCCA
Ggta1	TCTCAGGATCTGGGAGTTGGA	GAGTTCTATGGAGCTCCCGC
Gpc4	TGGACCGACTGGTTACTGATG	CCCTGGTTGGCTAATCCGTT
Gpc6	TTTCGACCCTACAACCCGGA	GTCTGTGACACTGTGCTGCAT
H2-T23	ATTGGAGCTGTTGTGAGGAGG	CCACGAGGCAACTGTCTTTTC
Hspb1	AAATACACGCTCCCTCCAG	GTGATCTCCGCTGATTGTG
Lcn2	GGAATATTCACAGCTACCCTC	ATACCATGGCAAACTGGTC
Megf10	TACCGCCATGGGGAGAAAAC	TTATCAGCGCAGTGAGGGAC
Mertk	CTGCTTCTGCGGGTTTGTTC	GGCTTTGCAAGGTAAGCTCG
Psap	CACCAAGGAGGAGATCCTG	AATTCATCACACTGCTTCTGG
Psmb8	CAGGAAGTTACATTGCTACCA	CACAACCAGACATGGTTCC
Ptgs2	CCAACCTCTCCTACTACACC	CCTTATTTCCTTTCACACCCA
Ptx3	CAATGGACTTCATCCCACC	GATGAACAGCTTGTCCCAC
S100a10	ATGGAAAGGGAGTTCCCTG	ATAGAAAGCTCTGGAAGCCC
S100b	AAGCACAAGCTGAAGAAGTC	CTGCTCTTTGATTTCCTCCA
S1pr3	ACTGTTGAGCTTCATCGTC	TTGACCTTGTAGGCTATGC
Serping1	TGGCTCAGAGGCTAACTGGC	GAATCTGAGAAGGCTCTATCCCCA
Sparc	GAGACAGGGTTACCTGTGG	CTCGACAGTTTCCTCTGCA
Sparcl1	CAGTCCCGACAACGTTTCTCT	CTGTCGACTGTTCATGGGCT
Sphk1	GAACTACTATGCTGGGCAC	GATTCATGGGTGACAGCTG
Srgn	GTTCAAGGTTATCCTGCTCGGA	AAACAGGATCGGTCATCGGG
Steap4	CAAACGCCGAGTACCTTGCT	CAGACAAACACCTGCCGACT
Tgm1	CACATAATCCTCTTCCTGAACC	CTCAGGATTGTTTCCGATGAG
Thbs1	AGAATGTGAGGTTTGTCTTTGG	GAAGGACGTTGGTAGAACTG
Timp1	TGATAGCTTCCAGTAAAGCC	CCCTTATAACCAGGTCCGA
Tm4sf1	CAGAAGGACCAAAGTGTAGC	AATCCAGAAGGTACTGTCCC
Vim	TCACTTCCTCTGGTTGACAC	ATTGATCACCTGTCCGTCTC
